# *In Vitro* Whole Genome DNA Binding Analysis of the Bacterial Replication Initiator and Transcription Factor DnaA

**DOI:** 10.1371/journal.pgen.1005258

**Published:** 2015-05-28

**Authors:** Janet L. Smith, Alan D. Grossman

**Affiliations:** Department of Biology, Massachusetts Institute of Technology, Cambridge, Massachusetts, United States of America; University of California, San Francisco, UNITED STATES

## Abstract

DnaA, the replication initiation protein in bacteria, is an AAA+ ATPase that binds and hydrolyzes ATP and exists in a heterogeneous population of ATP-DnaA and ADP-DnaA. DnaA binds cooperatively to the origin of replication and several other chromosomal regions, and functions as a transcription factor at some of these regions. We determined the binding properties of *Bacillus subtilis* DnaA to genomic DNA *in vitro* at single nucleotide resolution using *in vitro* DNA affinity purification and deep sequencing (IDAP-Seq). We used these data to identify 269 binding regions, refine the consensus sequence of the DnaA binding site, and compare the relative affinity of binding regions for ATP-DnaA and ADP-DnaA. Most sites had a slightly higher affinity for ATP-DnaA than ADP-DnaA, but a few had a strong preference for binding ATP-DnaA. Of the 269 sites, only the eight strongest binding ones have been observed to bind DnaA *in vivo*, suggesting that other cellular factors or the amount of available DnaA *in vivo* restricts DnaA binding to these additional sites. Conversely, we found several chromosomal regions that were bound by DnaA *in vivo* but not *in vitro*, and that the nucleoid-associated protein Rok was required for binding *in vivo*. Our *in vitro* characterization of the inherent ability of DnaA to bind the genome at single nucleotide resolution provides a backdrop for interpreting data on *in vivo* binding and regulation of DnaA, and is an approach that should be adaptable to many other DNA binding proteins.

## Author Summary

DNA binding proteins are involved in many cellular processes. The ability of these proteins to bind DNA is often modulated, either directly or indirectly. We determined the binding properties of *Bacillus subtilis* DnaA to genomic DNA at single nucleotide resolution using *in vitro* DNA affinity purification and deep sequencing. DnaA is the replication initiator and transcription factor and a AAA+ ATPase found in virtually all bacteria. Like other AAA+ proteins, DnaA binds ATP or ADP, and the identity of the nucleotide influences protein activity. We found that most DNA binding regions had a slightly higher affinity for ATP-DnaA than ADP-DnaA, but that a few regions had a strong preference for binding ATP-DnaA. Although some chromosomal regions were bound by DnaA both *in vitro* and *in vivo*, we observed many differences. Notably, we found regions that were bound *in vivo* that were not detectably bound *in vitro*. Binding to these regions *in vivo* required the nucleoid associated protein Rok. Our findings highlight the importance of other factors in the cell that modify association of DnaA with specific chromosomal regions. The general approach, to date used with only a couple of proteins, should be readily adaptable to many other DNA binding proteins.

## Introduction

DnaA is the highly conserved replication initiation protein found in virtually all bacteria [reviewed in 1, 2–6]. DnaA binds to multiple 9 bp "DnaA boxes" (DnaA binding sites; consensus sequence 5'-TTATNCACA) in the origin of chromosomal replication (*oriC*). Once properly bound to sites in *oriC*, DnaA causes melting of an AT-rich region and recruitment of the replication machinery. DnaA also binds to DnaA boxes at other chromosomal regions, and in some cases functions as a transcription factor, activating some genes and repressing others. DnaA directly activates transcription of *sda* [7–11], and appears to directly repress transcription of *ywlC*, *vpr*, and the *yyzF-yydD*, *trmE-noc*, and *ywcI-sacT* operons [8, 9, 12, 13].

DnaA is a AAA+ ATPase that is present in cells as both ATP-DnaA and ADP-DnaA. Although both ATP-DnaA and ADP-DnaA bind DNA, where analyzed, ATP-DnaA is required for replication initiation [3, 14–19]. For *E*. *coli* DnaA, there are some sites that appear to bind ATP-DnaA and ADP-DnaA equally well, and others that have a preference for ATP-DnaA [reviewed in 3]. The activity of *E*. *coli* DnaA is largely controlled by regulation of its ATPase activity and nucleotide exchange [reviewed in 1, 3, 20, 21]. In contrast, the activity of *Bacillus subtilis* DnaA appears to be largely regulated by several proteins, all of which affect its ability to bind cooperatively to DNA [22–24].

We set out to analyze the binding properties of *B*. *subtilis* DnaA to target chromosomal sites, on a genomic scale, *in vitro*, in the absence of other proteins. We used *in vitro* DNA affinity purification and sequencing (IDAP-Seq), an approach that is an *in vitro* analog of chromatin immunoprecipitation or affinity purification followed by deep sequencing, ChIP-Seq or ChAP-Seq, respectively. In IDAP-Seq, purified his-tagged protein (DnaA-his) is mixed with genomic DNA, the DNA bound to protein is isolated by affinity purification, and the bound DNA is analyzed by high throughput DNA sequencing. This approach has been used to analyze DNA binding by the transcriptional regulator CodY [25–27].

Using IDAP-Seq, we defined individual DnaA binding sites throughout the genome, and compared their affinity for ATP-DnaA and ADP-DnaA over a range of DnaA concentrations. We generated a position specific scoring matrix (PSSM) that can be used to predict DnaA binding sites with improved accuracy compared to a simple consensus sequence. The vast majority of sites bound *in vitro* have not been observed *in vivo*, suggesting that the amount of DnaA *in vivo* is limiting, or that other factors prevent binding at these sites under normal growth conditions. Conversely, we found some sites that were bound by DnaA *in vivo*, but that were not bound *in vitro*, indicating that at least one additional factor was involved in binding *in vivo*. We found that the nucleoid associated protein Rok was required for DnaA to bind to these chromosomal regions *in vivo*. Our results demonstrate that IDAP-Seq data can be used to understand and compare inherent binding properties of different forms of a given protein under defined *in vitro* conditions. In addition, comparison of IDAP-Seq data with data from *in vivo* analyses can provide insights into binding regions that require additional factors *in vivo*.

## Results and Discussion

### Overview of *in vitro* DNA affinity purification and sequencing (IDAP-Seq)

The overall goal of our experiments was to identify all regions in the *B*. *subtilis* genome capable of binding DnaA, and to compare the binding properties of ATP-DnaA and ADP-DnaA to these regions to gain a better understanding of how DnaA binding is regulated. We incubated various concentrations of purified functional C-terminal hexa-histidine tagged DnaA (DnaA-his) with either ATP or ADP, and mixed each nucleotide-bound form of DnaA-his with purified sheared genomic DNA from *B*. *subtilis*. The ATPase activity of DnaA was very low under these conditions, hydrolyzing 2.2 ± 0.6 moles of ATP per mole of DnaA per hr (n = 6). This rate of hydrolysis would cause a small (≤0.2%) decrease in the concentration of ATP over the course of the incubations. Given the rapid rate of exchange between ATP and ADP [23], we expect that after addition of ATP most of the DnaA will be in the ATP-bound form. Genomic DNA with a uniform copy number along the chromosome and nearly random shearing pattern was used in these experiments, facilitating quantitative analysis of the data (see S1 Text). We isolated DnaA-his along with any bound DNA fragments, without crosslinking, using affinity resin for the hexa-histidine tag. We analyzed binding over a range of DnaA concentrations to compare binding at different chromosomal regions and to estimate apparent binding constants. The identity and amounts of genomic regions bound by DnaA were determined by deep sequencing (IDAP-Seq).

### Binding of ATP-DnaA-his to genomic DNA *in vitro*

**Fig 1:**
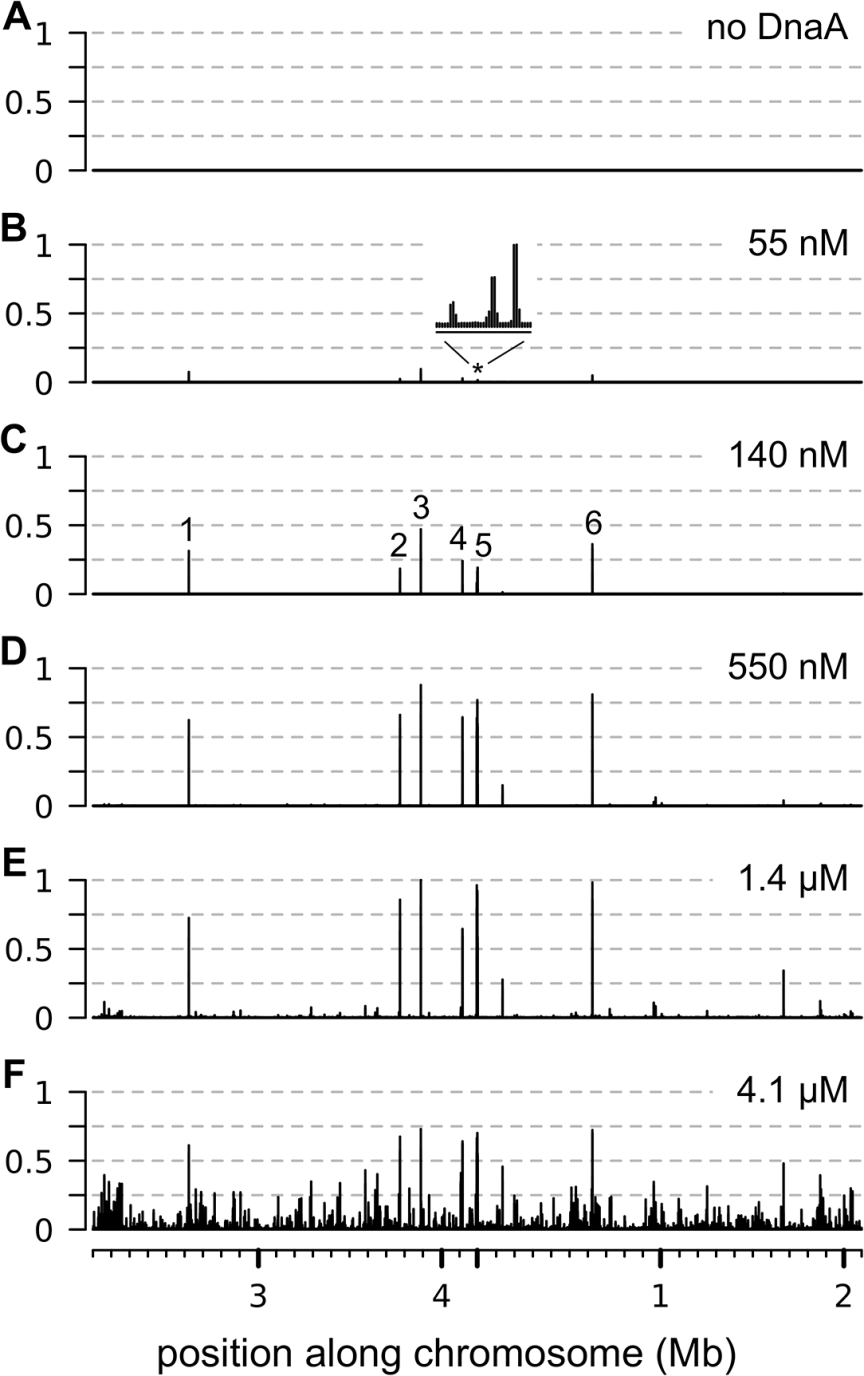
Binding of ATP-DnaA-his to genomic DNA *in vitro*. The relative amount of binding by ATP-DnaA-his is plotted on the y-axis (normalized so that maximum binding has an amplitude of 1) versus the position along the chromosome on the x-axis. The amount of binding was determined by sequence analysis of the DNA recovered in each binding reaction. Binding data is presented in 200 nucleotide bins, with the maximum binding amplitude in each bin drawn. The 4.2 mb circular chromosome is depicted linearly such that the origin of replication is near the middle of the x-axis. The concentration of ATP-DnaA-his in each binding reaction was *(A)* no DnaA; *(B)* 55 nM; *(C)* 140 nM; *(D)* 550 nM; *(E)* 1.4 μM; *(F)* 4.1 μM. The major peaks are numbered *(C)*, and correspond to the following nearby loci: (1) *sda*; (2) *ywlC*; (3) *ywc*I; (4) *yydA*; (5) consists of 3 adjacent peaks (*trmE*, *dnaA*, and between *dnaA* and *dnaN*) that are not resolved at this scale; (6) *gcp*/*ydiF*. The inset in panel B above the asterisk corresponds to a 7 kb region that contains the *trmE*, *dnaA*, and *dnaA/N* binding regions.

Using IDAP-seq, we identified the chromosomal regions that had increased binding by DnaA within the range of 55 nM to 4.1 μM DnaA. We found that the number of chromosomal regions bound and the amount of binding to individual regions increased with increasing concentrations of ATP-DnaA-his (Fig 1). There were no specific chromosomal regions recovered in control reactions with no added DnaA, as assessed by the distribution of sequencing reads over the genome (Fig 1A). In contrast, there were eight chromosomal regions predominantly associated with 55 nM ATP-DnaA-his following affinity purification (Fig 1B). These regions were the same as the major DnaA binding regions previously defined *in vivo* [8, 9, 12, 13, 28]. They have a greater number of DnaA boxes than the other regions detected *in vitro* that required higher concentrations of DnaA for binding. As the concentration of ATP-DnaA-his was increased (55 nM; 140 nM; 550 nM; 1.4 μM; 4.1 μM), binding to the eight predominant regions increased and appeared to become saturated (Fig 1B–1F and S1 Fig, panels 1–8). In addition, binding to many other regions was detected and increased with increasing concentrations of ATP-DnaA-his. Confirmation that binding was mediated by the DnaA-binding domain of DnaA was obtained for six of the regions, spanning a wide range of affinities, by performing a parallel assay with a mutant DnaA (DnaA∆C-his) that is missing the DNA binding domain (S2 Fig).

We identified 269 chromosomal regions that were bound by 1.4 μM ATP-DnaA-his (S1 Fig and S1 Table). This list includes all the regions that were bound at lower concentrations of DnaA, and also those that had increased binding at 4.1 μM DnaA. There was an approximately 300-fold difference in the amount of DNA detected from the weakest bound regions compared to the strongest sites at 1.4 μM ATP-DnaA-his, the second highest DnaA concentration tested. There were many additional regions bound at 4.1 μM ATP-DnaA-his, the highest concentration tested, that were not detected at the lower concentrations (Fig 1F). Because the amount of binding at these regions was low and was not detected at other concentrations of DnaA, they were not included in the list of binding regions (S1 Table).

The number of binding regions (269) determined *in vitro* is significantly greater than the previously known binding regions (eight) determined *in vivo*. Because several different analyses of *in vivo* binding did not detect these additional regions [8, 9, 12, 13, 28], it seems unlikely that all, or even most, of these 269 regions are occupied by DnaA *in vivo*. The much larger number of regions bound by DnaA *in vitro* could be due to a combination factors, including the much higher sensitivity of the *in vitro* system, the possibility that the amount of DnaA *in vivo* is limiting, and the fact that DnaA binding is regulated *in vivo*.

### Analysis of binding regions at single nucleotide resolution

**Fig 2:**
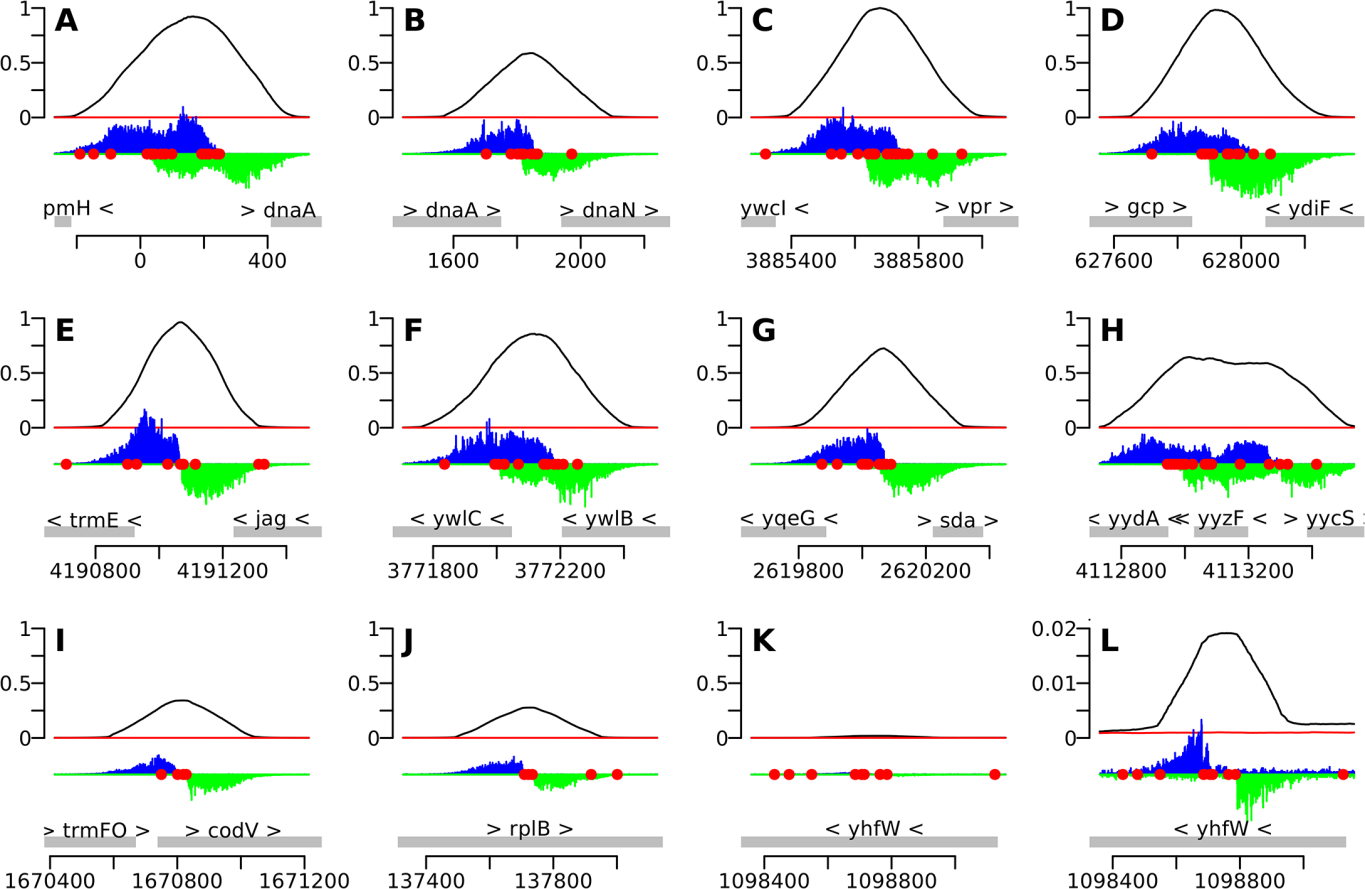
IDAP-Seq data for individual regions bound by DnaA. The 10 strongest binding regions *(A-J)*, plus one weaker binding region *(K*, *L)*, are displayed, using binding data at 1.4 μM ATP-DnaA-his. The bottom section of each panel shows the genomic coordinates (using the AG1839 genomic sequence), the positions of each gene (gray rectangles), and the gene names. Arrowheads indicate the direction of transcription. The middle section of each panel is a histogram of the number of sequence reads that start at each nucleotide (blue, above the line, for sequence reads mapping to the top strand; green, below the line, for sequence reads mapping to the bottom strand). The red circles indicate DnaA boxes, predicted using the PSSM described in this paper. The top section of each panel is a plot of the amount of DNA recovered (as inferred from the sequence data) versus genome position, using 1.4 μM ATP-DnaA-his (black line) or no DnaA (red lines). The amount of DNA recovered is scaled to a global maximum of 1, as described for Fig 1. *A-J*. The 10 strongest binding regions, corresponding to peaks #1–10 in Table 1, S1 Table, and S1 Fig. *(A)* upstream of *dnaA* (part of *oriC*); *(B)* between *dnaA* and *dnaN*, containing the DNA unwinding element (also part of *oriC*); *(C)* upstream of *ywcI* and *vpr*; *(D)* downstream of *gcp* and *ydiF*; *(E)* upstream of *trmE* and downstream of *jag*; *(F)* upstream of *ywlC* and downstream of *ywlB*; *(G)* upstream of *yqeG* and *sda*; *(H)* upstream of *yydA*, spanning *yyzF*, and upstream of *yycS*; *(I)* within *codV*; *(J)* within *rplB*. *K-L*. A representative weaker binding region, (peak #49 in S1 Table; S1 Fig). *(K)* binding scaled to 1 to be comparable to previous panels; *(L)* the same region rescaled so that the binding pattern is visible.

**Fig 3:**
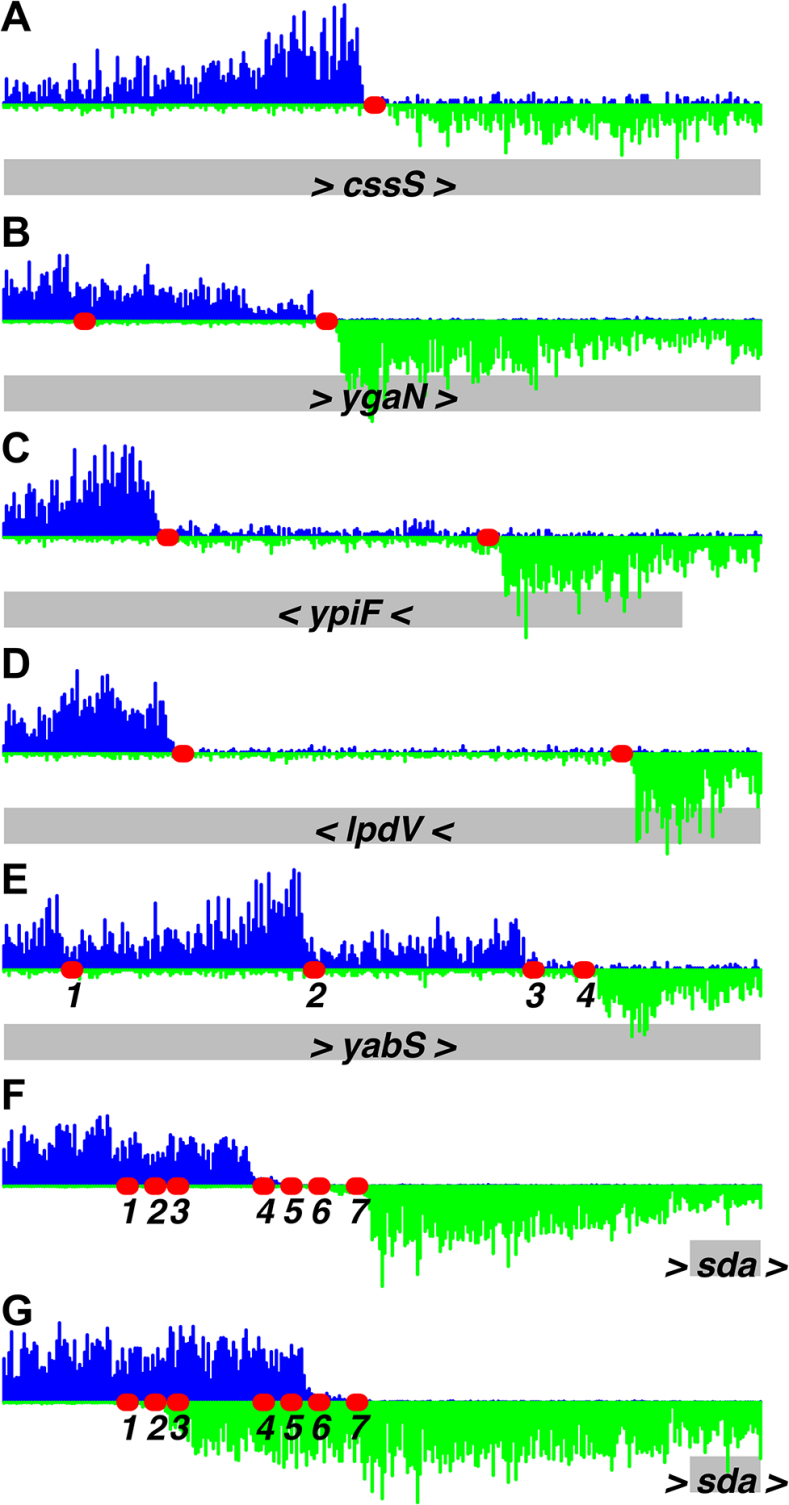
Identification of DnaA binding sites at single nucleotide resolution. Histograms of the number of sequence reads that start at each nucleotide are plotted as in Fig 2 (blue, above the line, for sequence reads mapping to the top strand; green, below the line, for sequence reads mapping to the bottom strand). Data are from a binding reaction containing 4.1 μM ATP-DnaA-his, except for panel *F*, where data are from a reaction containing 55 nM ATP-DnaA-his. For each panel, a 300 bp portion of the genome is presented, and the y-axis is scaled so that the data fills the space. Predicted DnaA boxes are represented by red ovals, and are numbered from left to right for regions with more than three DnaA boxes. The gray rectangles below the histograms depict nearby genes, with arrowheads indicating the direction of transcription. The regions presented are: *(A)* inside *cssS*; *(B)* inside *ygaN*; *(C)* inside *ypiF*; *(D)* inside *lpdV*; *(E)* inside *yabS*; *(F)* upstream of *sda* (55 nM ATP-DnaA-his data); *(G)* upstream of *sda* (4.1 μM ATP-DnaA-his data). Panel B corresponds to peak #140 (S1 Table and S1 Fig) and panels F and G correspond to peak #7. The other regions did not show sufficient binding at 1.4 μM DnaA to be included in the peak catalog.

We used the IDAP-Seq data to visualize binding by DnaA at single nucleotide resolution (Figs 2 and 3 and S1). In these analyses, the number of sequence reads starting at a specific nucleotide position was determined, and the reads were extended and summed to generate a curve indicative of total binding. If a specific DNA sequence is required for binding, then no sequence reads should start in that region. At 1.4 μM ATP-DnaA-his, the binding patterns for the strongest binding regions with multiple DnaA boxes were complex (Fig 2A–2H), often having plateaus and multiple peaks of read start sites. In contrast, other regions with fewer DnaA boxes typically had a single, well-defined peak on each strand (Figs 2I–2L and 3A–3D).

**Table 1:** Top 20 DnaA binding regions *in vitro*.

				Kd (μM)^4^	
Region^1^	nearby gene(s)^1^	summit^2^	sites^3^	ATP	ADP
1	upstream of *rpmH*; upstream of *dnaA*	150	12	0.21	0.33
2	downstream of *dnaA*; upstream of *dnaN*	1841	8	0.16	0.30
3	upstream of *ywcI*; upstream of *vpr*	3885674	11	0.13	0.17
4	downstream of *gcp*; downstream of *ydiF*	627955	10	0.15	0.33
5	upstream of *trmE*; downstream of *jag*	4191071	4	0.33	0.43
6	upstream of *ywlC*; downstream of *ywlB*	3772105	9	0.25	0.45
7	upstream of *yqeG*; upstream of *sda*	2620051	8	0.15	0.51
8	upstream of *yydA*; downstream of *yyzF*	4113012	7	0.16	0.33
9	inside *codV*	1670814	4	1.1	1.9
10	inside *rplB*	137727	2	1.4	1.2
11	downstream of *ynaI*; upstream of *xynP*	1870622	3	2.0	3.0
12	inside *yhcA*	962763	3	3.6	2.8
13	inside *yorF*	2158839	4	2.0	3.0
14	upstream of *yvmC*; upstream of *yvmB*	3582878	2	2.2	4.4
15	inside *yhcN*	972751	3	19.4	6.9
16	inside *yutJ*	3287547	5	2.5	2.9
17	inside *yydH*	4104262	3	2.4	4.6
18	inside *ggaA*	3648588	4	2.7	4.8
19	inside *yopH*	2184371	2	4.9	5.0
20	inside *rlmCD*	722497	3	7.5	6.7

^1^The two binding regions in *oriC* are listed first, and subsequent peaks are ordered by the amounts of DNA recovered at 1.4 μM ATP-DnaA-his. For peaks in intergenic regions, both flanking genes are indicated. For peaks located within genes, only that gene is indicated.

^2^The numbers presented are the genome coordinates for the summit of each peak. The genome sequence used was from lab strain AG1839 [29] with 1 being the nucleotide 409 bp upstream of the *dnaA* open reading frame.

^3^The number of putative DnaA binding sites (DnaA boxes) in a 300 bp window centered on the peak summit, as determined using the PSSM (Materials and Methods).

^4^Apparent K_d_’s were determined as described (Materials and Methods) and are indicated for ATP-DnaA-his (ATP) and ADP-DnaA-his (ADP).

Analyses and visualization of the binding regions at single nucleotide resolution provided insights into the requirements and contributions of individual DnaA boxes. A characteristic almost symmetric pattern of sequence reads beginning on either side of an otherwise "bare" region was indicative of a simple binding region containing a single DnaA box (Fig 3A and 3B). This footprint-like region defines the binding site and can be used to determine binding sites for uncharacterized proteins. Similarly, a larger bare region flanked by sequence reads on opposite strands was indicative of two binding sites, both of which appear to be required for DnaA to bind the region (Fig 3C and 3D). In more complex regions (e.g., Fig 3E), some DnaA boxes (numbered 1 and 2) appeared to make partial contributions to binding, as evidenced by an abrupt decrease in, but not a complete elimination of, reads at the junctions of the DnaA boxes. In contrast, DnaA boxes 3 and 4 appeared to be required for binding, since no sequence reads started in or between them.

The strongest binding regions contain arrays of DnaA boxes, and had complex binding patterns (Fig 2A–2H and 3F and 3G). In addition to DNA fragments that contained the complete array of DnaA boxes, fragments were also efficiently recovered that had one end within the array and therefore contained only a subset of the DnaA boxes. The requirement for specific DnaA boxes varied with the DnaA concentration. For example, in the *sda* promoter region, DnaA boxes 4, 5, 6, and 7 (Fig 3F) were required for binding at the lowest concentration (55 nM) of ATP-DnaA-his tested. However, at the highest ATP-DnaA-his concentration (4.1 μM), fragments were efficiently recovered as long as they contained either DnaA boxes 1 and 2 or DnaA boxes 6 and 7 (Fig 3G). The finding that DnaA boxes 1 and 2 contribute to binding is consistent with *in vivo* results showing that these sites are important for full activation of transcription of *sda* by DnaA, and that a mutation in either of these individual DnaA boxes causes a reduction in *sda* expression [7].

The single nucleotide resolution afforded by IDAP-seq is somewhat reminiscent of the resolution obtained with DNA footprinting. Published footprinting data for DnaA binding to *B*. *subtilis* DNA is available for two sites: the *dnaA* promoter region, and the region upstream of the DUE [30]. About half of the DnaA boxes observed by footprinting of these regions were directly supported by our IDAP-seq data. For the remaining footprinted sites, it was not possible to determine whether or not they were bound in our assay. This is because the IDAP-seq method is more analogous to a single nucleotide truncation analysis than footprinting, and in regions that contain arrays of DnaA boxes (including the *dnaA* promoter and the DUE), removal of one DnaA box from the end will not always give a robust change in DNA recovery if its contribution is small compared the remaining DnaA boxes.

### Position-specific scoring matrix for DnaA binding sites

We used a subset of the binding data to establish a position-specific scoring matrix (PSSM) that predicted DnaA binding sites more effectively than a simple consensus sequence. When we searched for potential DnaA binding sites based on the consensus sequence 5'-TTATNCACA-3' [31] we found that restricting the search to one mismatch was overly stringent, whereas allowing two mismatches from the consensus resulted in an excessive number of predicted DnaA boxes throughout the genome. A PSSM was developed to more accurately predict the binding sites observed in our experiments.

**Fig 4:**
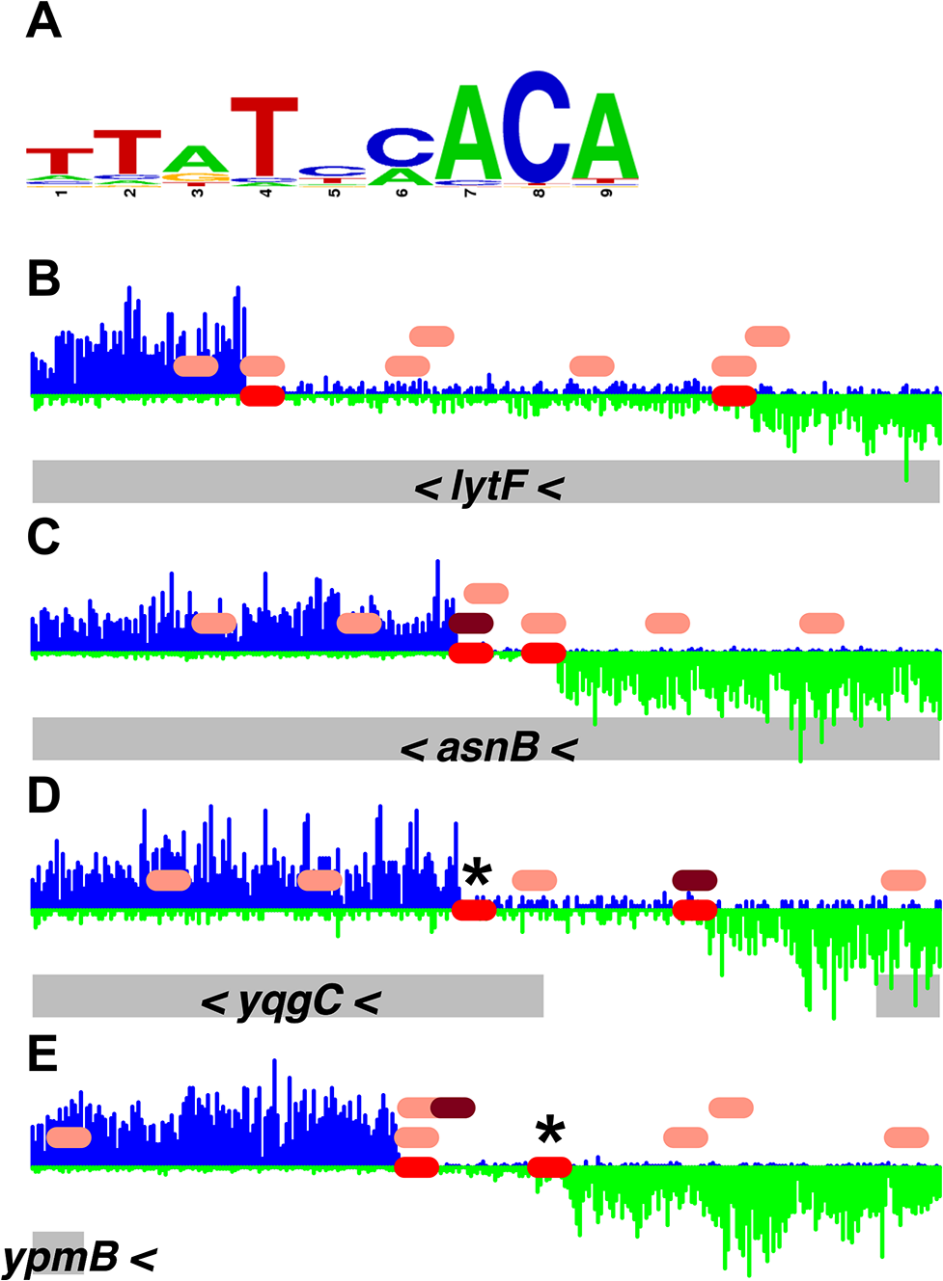
Comparison of DnaA boxes from a consensus sequence with DnaA boxes from the PSSM. *(A)* A logo, drawn using WebLogo [32], of the DnaA boxes used to construct the DnaA box PSSM is shown. *(B-E)* Histograms of the number of sequence reads that start at each nucleotide are plotted (blue for sequence reads mapping to the top strand; green for sequence reads mapping to the bottom strand). Data are from the binding reaction containing 4.1 μM ATP-DnaA-his. For each panel, a 300 bp portion of the genome is presented, and the y-axes are scaled so that the data fills the space. The red ovals on the horizontal axis indicate the position of DnaA binding sites predicted using the PSSM described here. The pink ovals above the horizontal axis indicate DnaA boxes with 2 mismatches from the TTATNCACA consensus sequence, and the maroon ovals have 1 mismatch from the TTATNCACA consensus. (No perfect matches to the consensus are found in these regions.) The gray rectangles below the histograms indicate nearby genes, with arrowheads indicating the direction of transcription.

We derived a PSSM from 150 DnaA box sequences (S2 Table) that were observed to bind DnaA in our IDAP-Seq data. Whereas the PSSM (Fig 4A and S3 Table) was consistent with the previously determined consensus sequence, it provided a more sensitive measure of the extent to which mismatches are tolerated at each position. We predicted a total of 11,353 DnaA boxes over the whole genome using the PSSM, and in general these putative DnaA boxes were more closely correlated with binding than boxes predicted using the consensus sequence with up to two mismatches (e.g., Fig 4B–4E). In many instances, the PSSM identified functional DnaA boxes that had three mismatches from the consensus (asterisks in Fig 4D and 4E). In contrast, using the consensus sequence and allowing three mismatches predicted one DnaA box every 17 bp of genomic sequence. The vast majority of the 269 regions bound by ATP-DnaA-his contained at least one DnaA box centered at the peak summit or two DnaA boxes flanking the peak summit (S1 Fig and S4 Table). A total of 784 predicted DnaA boxes were found within 150 bp of the summits of these 269 binding regions. A Pearson’s correlation coefficient of 0.74 was observed between the amount of binding observed at each region and how well the DnaA boxes in that region matched the PSSM (S4 Fig).

Many additional predicted DnaA boxes were bound at 4.1 μM ATP-DnaA-his, and others might require yet higher DnaA concentrations to bind. In addition, there are almost certainly some predicted DnaA boxes that are not functional, either because of limitations in the PSSM itself, or due to that fact that the PSSM approach does not take into consideration other factors that could influence binding, including flanking sequences and the number, orientation, and spacing of nearby DnaA boxes.

### Apparent binding constants for several regions throughout the genome

**Fig 5:**
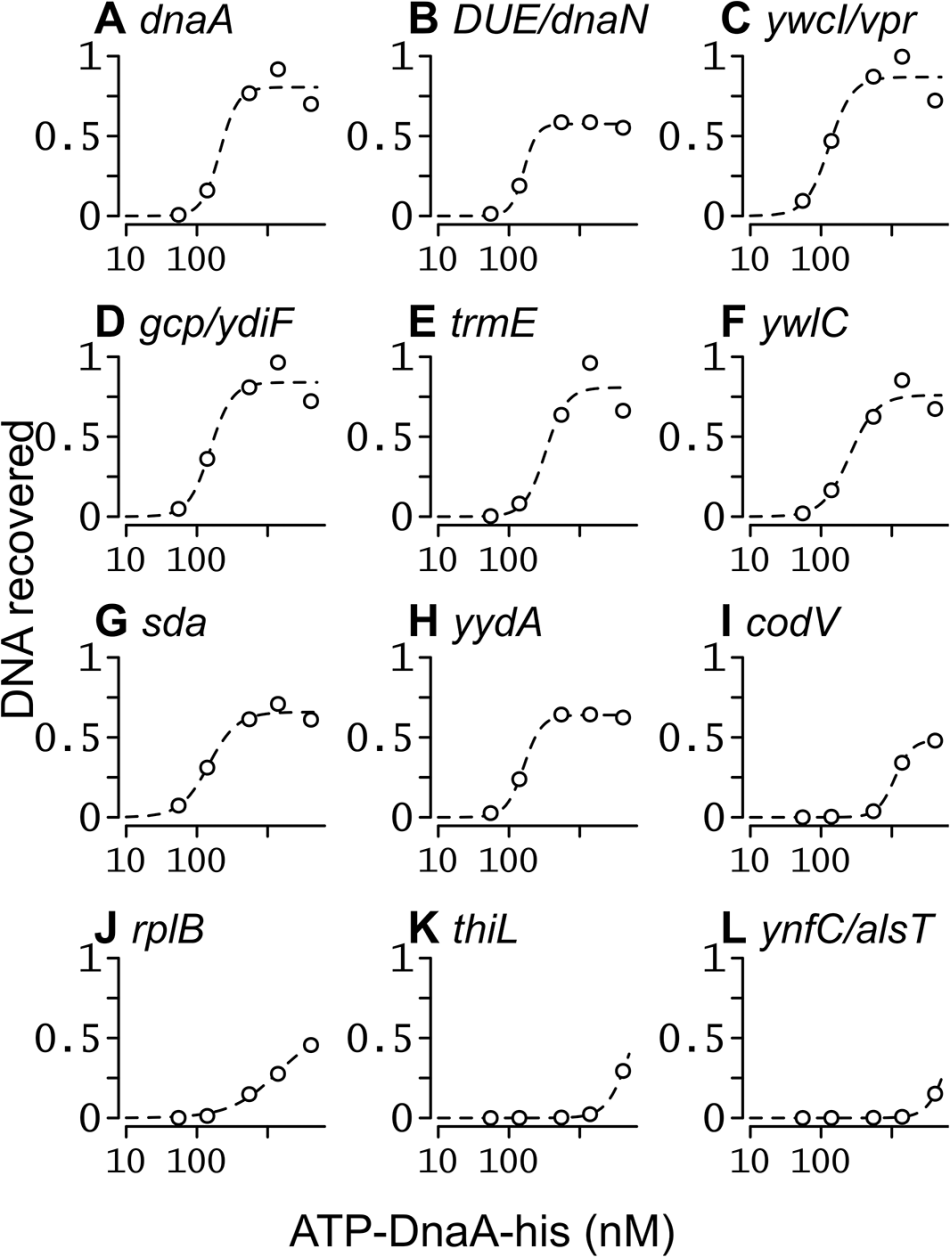
Binding curves for ATP-DnaA-his for selected chromosomal regions. The relative amount of DNA recovered from different chromosomal regions is plotted on the y-axis, versus the ATP-DnaA-his concentration on the x-axis. The curves were obtained from fitting the data to the Hill Equation. Chromosomal locations (nucleotide position in the sequence of AG1839, peak number in S1 Table, and nearby genes) include: *(A)* 150, #1, upstream of *rpmH* and *dnaA*; *(B)* 1841, #2, downstream of *dnaA* and upstream of the DUE and *dnaN*; *(C)* 3885674, #5, upstream of *ywcI* and *vpr*; *(D)* 627955, #3, downstream of *gcp* and *ydiF*; *(E)* 4191071, #4, upstream of *trmE* and downstream of *jag*; *(F)* 3772105, #6, upstream of *ywlC* and downstream of *ywlB*; *(G)* 2620059, #7, upstream of *yqeG* and *sda*; *(H)* 4113012, #8, upstream of *yydA* and downstream of *yyzF*; *(I)* 1670814, #9, inside *codV*; *(J)* 137727, #10, inside *rplB*; *(K)* 624773, #40, inside *thiL*; *(L)* 1922157, #127, upstream of *ynfC* and *alsT*.

We used the IDAP-Seq data to estimate apparent dissociation constants for several of the 269 regions bound at 1.4 μM ATP-DnaA-his. We plotted the amount of DNA recovered as a function of ATP-DnaA-his concentration for each of the 269 binding regions (Figs 5 and S1). For each region the nucleotide position with the maximum amount of DNA (peak summit) was identified, and the relative number of DNA fragments that spanned that position was determined at each DnaA concentration (Materials and Methods; S3 Fig). Genomic DNA (~300 μM of base pairs) was used in the binding reactions, providing an excess of non-specific DNA for competition in binding to DnaA.

For regions with high affinity, the amount of DNA recovered became saturated as the ATP-DnaA-his concentration was increased (Fig 5A–5H). For regions with intermediate affinity, the amount of DNA recovered appeared to begin to saturate at the highest concentration of ATP-DnaA-his (Fig 5I and 5J). For the remaining regions, the amount of DNA recovered increased with the concentration of ATP-DnaA-his, but did not reach saturation (e.g., Fig 5K and 5L). Evaluating relative binding for the weaker binding regions can be done by comparing the relative amount of DNA recovered at 1.4 or 4.1 μM DnaA (S1 Table). DnaA binding to almost all regions was cooperative, consistent with the positive cooperative binding previously observed for *B*. *subtilis* DnaA [22–24, 30].

The apparent binding constants determined by IDAP-seq for the eight high affinity binding sites ranged from 0.13–0.33 μM (Table 1, S1 Table), and were several-fold greater than those determined previously by gel mobility shift assays [22, 23]. Although IDAP-seq measurements have inherently higher error owing to the complex experimental protocol, the higher K_d_’s can be attributed, in large part, to washing the bound complexes in the IDAP experiments and the absence of caging effects from gel electrophoresis. In gel mobility shift assays, the complexes are loaded directly on a gel, where caging effects of the gel matrix stabilize binding. Other factors, including the heterogeneous nature of the DNA template for binding, the presence of multiple binding regions with similar affinities, and the excess of competitor DNA in the IDAP-Seq experiments, likely also contribute to the higher apparent K_d_’s determined with IDAP-Seq compared to those determined by gel shift assays. These differences between IDAP-Seq and gel mobility shift assays likely also affect estimates of cooperativity.

### Comparisons of binding by ATP-DnaA-his to that of ADP-DnaA-his

**Fig 6:**
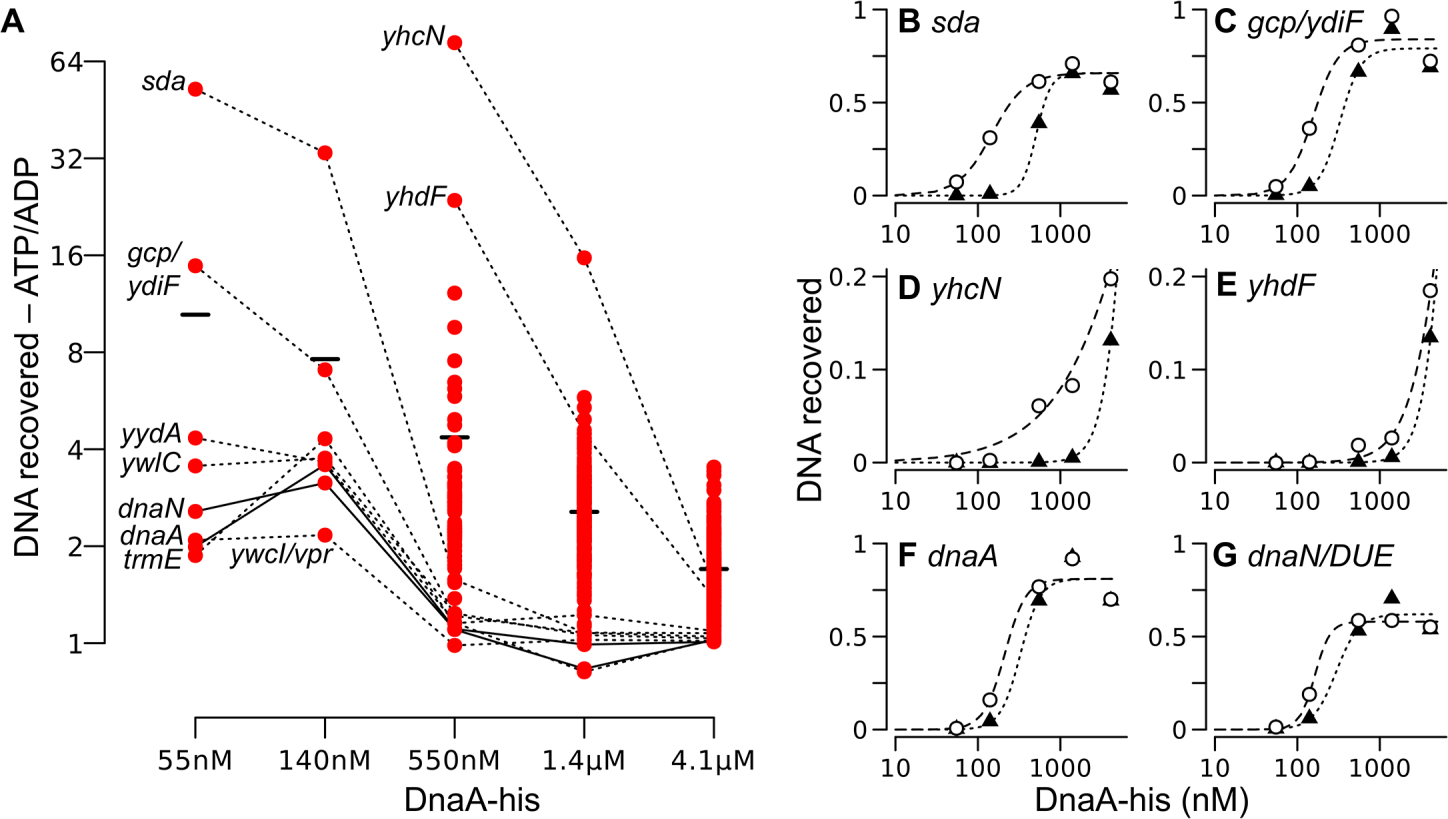
Relative DNA binding by ATP-DnaA-his compared to ADP-DnaA-his. *(A)* The ratio of the amount of DNA recovered bound to ATP-DnaA-his vs. ADP-DnaA-his is plotted versus the DnaA concentration. Background binding was subtracted prior to calculating the ratio. All 269 peaks detected at 1.4 μM were analyzed at each concentration, but the ratio of ATP/ADP binding is shown only if the binding was 1.5-times greater than background for both the ATP and the ADP binding reactions at that DnaA-his concentration. For many weaker peaks, these criteria were met only at the highest concentration of DnaA-his. Solid and dotted lines connect points for the indicated region across concentrations. The black bars indicate the average ratio (ATP-DnaA-his/ADP-DnaA-his) at each DnaA-his concentration. The two data points at 1.4 μM DnaA-his that are slightly less than 1 correspond to the *yydA* and *dnaN* regions. *(B-G)* The relative amount of DNA recovered from different chromosomal regions is plotted on the y-axis, versus the DnaA-his concentration (log scale) on the x-axis. ATP-DnaA-his, open circles and dashed lines (data are the same as shown in parts of Fig 5); ADP-DnaA-his, filled triangles and dotted lines. Genomic coordinates (AG1839 genome), and nearby genes: *(B)* 2620051, upstream of *sda* and *yqeG*; *(C)* 627955, downstream of *gcp* and *ydiF*; *(D)* 972751, inside *yhcN*; *(E)* 1006129, inside *yhdF*; *(F)* 150, *oriC* upstream of *dnaA*; *(G)* 1841, *oriC* upstream of the DUE and *dnaN*.

We found that the overall binding patterns for ADP-DnaA-his (S5 Fig) were similar to those for ATP-DnaA-his (Fig 1). At the lowest concentration of DnaA tested, the prominent binding regions were the same eight regions that were bound by ATP-DnaA-his, and the number of bound regions increased at higher concentrations of ADP-DnaA-his (S5 Fig). The amount of binding to any specific region at a given concentration of DnaA was almost always greater with ATP-DnaA-his than with ADP-DnaA-his. This is seen by comparing the ratio of binding (amount of DNA recovered) by ATP-DnaA-his to that by ADP-DnaA-his (Fig 6A). Two regions (the *yydA* promoter region and the region in *oriC* between *dnaA* and *dnaN*) appeared to have a small preference for ADP-DnaA-his over ATP-DnaA-his, but only at 1.4 μM DnaA (Fig 6A and 6G).

A few regions had a strong preference for ATP-DnaA-his (Fig 6A). Among the eight high affinity regions, the most dramatic differences between ATP-DnaA-his and ADP-DnaA-his were observed in the *sda* promoter region and the region between the 3’ ends of *gcp* and *ydiF* (Fig 6A and 6B and 6C). Approximately 50-fold more DNA from the *sda* promoter region was recovered with 55 nM ATP-DnaA-his than with 55 nM ADP-DnaA-his. For the region between *gcp* and *ydiF*, this difference was 16-fold. The differences between ATP- and ADP-DnaA-his diminished at higher DnaA concentrations as binding became saturated.

Large differences between binding by ATP-DnaA-his and ADP-DnaA-his were also observed for weaker binding regions. For example, there was detectable binding to *yhcN* by ATP-DnaA-his at a concentration of 140 nM, whereas binding by ADP-DnaA-his was not detected until 550 nM (Fig 6A and 6D). At 550 nM DnaA, there was 73-fold more *yhcN* DNA bound to ATP-DnaA-his compared to ADP-DnaA-his. Similarly, there was 24-fold more *yhdF* bound to 550 nM ATP-DnaA-his compared to ADP-DnaA-his (Fig 6A and 6E). Although we cannot be certain that homogeneous DnaA-ATP or DnaA-ADP was present in the respective reactions, if heterogeneity did exist, it would cause an underestimate of the differences between DnaA-ATP and DnaA-ADP.

The basis for some DnaA sites exhibiting much higher affinity for ATP-DnaA than ADP-DnaA is almost certainly due to a combination of factors, including the sequence, orientation and spacing of the DnaA boxes, and the sequences flanking the DnaA boxes. There are not enough regions to define the features that contribute to the large differences.

#### The *dnaA-dnaN oriC* region

The *oriC* region includes two clusters of DnaA binding sites: one in the *dnaA* promoter region (Fig 2A), and the other between *dnaA* and *dnaN* (Fig 2B), just upstream of the DNA unwinding element (DUE). The difference between the nucleotide bound forms for these *oriC* binding regions are fairly modest—a maximal difference is seen at 140 nM DnaA-his, where 3–4 times more DNA is bound with ATP compared to ADP (Fig 6A and 6F and 6G). It is likely that *in vivo*, DnaA is bound to the *oriC* sites regardless of whether DnaA is in the ATP or ADP bound form. Thus far, none of the known regulators of replication initiation in *B*. *subtilis* affect nucleotide binding, exchange, or hydrolysis by DnaA. Rather, the four characterized regulators of replication initiation in *B*. *subtilis*, YabA [22, 33, 34], Soj [24], SirA [35–37], and DnaD [23, 34], all affect the ability of DnaA to bind DNA. Our findings that DnaA binding to the *oriC* region is not particularly sensitive to the nucleotide bound state of DnaA are consistent with the emerging view that regulation of nucleotide hydrolysis and exchange may not play a predominant role in the regulation of replication initiation in *B*. *subtilis*, in contrast to the regulation in *E*. *coli* [6, 38].

#### sda

Whereas the activity of DnaA during replication initiation appears to be regulated primarily at the level of oligomerization (rather than nucleotide binding) this is not necessarily the case when DnaA functions as a transcription factor. Our finding that ATP-bound DnaA binds to the *sda* promoter region much more strongly than the ADP-bound form may point to an important role for bound nucleotide in transcriptional regulation at this locus, and perhaps other loci where DnaA functions as a transcriptional regulator.

The *sda* gene product inhibits activation of Spo0A [7, 10, 11], a transcription factor that functions during stationary phase and sporulation. DnaA activates transcription of *sda in vivo*, thereby inhibiting entry into sporulation. Because the Sda protein is highly unstable [39], changes in transcription of *sda* will result in rapid changes in Sda protein levels.

Our findings indicate that transcription from the *sda* promoter may be highly sensitive to the relative amounts of ATP- and ADP-DnaA. These changes could occur due to modulation of the nucleotide hydrolysis and exchange activities of DnaA, or due to changes in the cellular ratio of ATP and ADP. Further studies will be needed to determine whether these potential mechanisms are important *in vivo* for DnaA/Sda-mediated activation of stationary phase and sporulation gene expression pathways.

### Comparisons of DnaA binding *in vitro* and *in vivo*

Our *in vitro* data on DnaA binding provides a framework for interpreting *in vivo* DnaA ChIP results, and *vice versa*. We anticipated three general types of findings when comparing *in vitro* to *in vivo* binding by DnaA: 1) binding is detected both *in vitro* and *in vivo*; 2) binding is detected *in vitro* but not *in vivo*; and 3) binding is detected *in vivo*, but is not detected *in vitro*.

Of the 269 binding regions identified *in vitro* at 1.4 μM ATP-DnaA-his, only the eight strongest binding regions have been readily detected *in vivo* [8, 9, 12, 13]. The next strongest binding regions *in vitro* were within the open reading frames of *codV* (encoding a homologue of the tyrosine recombinase XerC), and *rplB* (encoding ribosomal protein L2) (Table 1). We estimated that the *in vivo* concentration of DnaA is ~1–2.5 μM in cells growing exponentially in minimal glucose medium at 30°C. The amount of binding at *rplB in vitro* at the 1.4 and 4.1 μM ATP-DnaA-his concentrations is 28–46% that observed for the eight sites that are readily observed *in vivo*. If no other factors affect binding, then this indicates that DnaA could bind *rplB* in a significant fraction of cells. Instead we found no detectable DnaA binding to *rplB in vivo* using ChIP-PCR.

We suspect that there are factors *in vivo* that prevent DnaA from binding to the site within *rplB*. For example, since the binding site is within the *rplB* open reading frame, it is possible that transcription prevents stable association of DnaA with the site. Alternatively, the concentration of available DnaA might be limited by titration due to efficient binding at other regions [e.g., 40]. It is also possible that there is some binding *in vivo*, but that it is below the limit of detection of the ChIP-PCR assay, or that binding occurs under biological conditions that we have not assayed.

### DnaA depends on Rok *in vivo* to bind to some chromosomal regions

We used ChIP-PCR to measure DnaA binding *in vivo* at four regions that we observed to bind DnaA in preliminary *in vivo* ChIP-seq experiments. These regions had not been identified in previously reported *in vivo* ChIP experiments with DnaA [8, 9, 12, 13], perhaps due to lower sensitivity of the detection methods. These are all intergenic regions (between: *ywiB*-*sboA*, *yuzB*-*yutJ*, *yjcM*-*yjcN*, and *icsS*-*braB*) and contain promoters in one or both directions [41]. We found that DnaA was consistently associated with these regions in ChIP-PCR experiments (n = 6; Table 2), with mean fold enrichment values ranging from 4.5 (*iscS*) to 12.8 (s*boA*), compared to 83-fold for the *dnaA* promoter, a control site that is readily detected *in vivo* [8, 9, 12, 13, 28].

**Table 2:** Effects of Rok on binding by DnaA in vivo^1^.

region	*rok+*	*rok-*	P
*iscS*	4.5 ± 1.5	1.2 ± 0.3	2.8E-04
*yuzB*	5.2 ± 2.1	1.9 ± 0.6	3.1E-03
*sboA*	12.8 ± 5.6	1.7 ± 0.8	7.1E-04
*yjcM*	11.0 ± 4.2	1.7 ± 0.7	3.5E-04
*dnaA*	83 ± 29	53 ± 26	5.5E-02

^1^ Wild type (*rok*+; AG174) and a *rok* null mutant (*rok*-; HM57) were grown to mid-exponential phase in LB medium. Crosslinking, immunoprecipitation with anti-DnaA antibodies, and PCR with primers to the indicated chromosomal regions were done as described (Materials and Methods). Numbers indicate the fold enrichment of the indicated chromosomal regions in the immunoprecipitate compared to a control chromosomal region ± standard deviation from six independent cultures. P values are from a one-sided Student’s t-Test. The effects of *rok* on DnaA binding are significant at all loci indicated, except for *dnaA*.

Interestingly, none of these four regions bound DnaA *in vitro* in our IDAP-seq experiments, even at the highest concentration of DnaA tested. Furthermore, only the *sboA* region has a recognizable DnaA box near the *in vivo* binding site. The simple interpretation of these results is that there is a factor needed for binding *in vivo* that is not present in the purified *in vitro* binding reactions. Because three of these four regions bound by DnaA *in vivo* but not *in vitro* were previously found to be bound *in vivo* by the nucleoid-associated protein Rok [42], we tested whether Rok might be required for DnaA binding at these regions *in vivo*. In a *rok* null mutant, there was much less association of DnaA with these regions compared to wild type cells (Table 2). DnaA protein levels are not substantially different in *rok* null mutant cells (S6 Fig), indicating that the loss of binding was not due to a decreased amount of DnaA. Consistent with this, binding at the *dnaA* promoter, which has 12 DnaA boxes and binds DnaA robustly *in vitro*, was not significantly different between wild type and *rok* mutant cells (Table 2). These results indicate that Rok is required for association of DnaA with these chromosomal regions *in vivo*.

### General utility of IDAP-Seq approach

The IDAP-Seq approach used here to study DNA binding by DnaA should be a useful for determining the inherent binding properties of many different proteins, and the effects of various ligands, such as the ATP and ADP comparison presented here. It should also be possible to determine the effects of mutations, phosphorylation, acetylation, and other modifications on DNA binding. The effects of regulatory proteins that modulate binding could also be evaluated, provided the two proteins do not have the same tag. In all cases, apparent binding constants can be determined and compared for sites throughout the entire genome.

Experiments to date with DnaA and CodY [25–27] used a his-tagged version of the DNA binding protein, with DNA-protein complexes recovered by metal affinity chromatography. Other tags could potentially be used for purification of complexes. In preliminary experiments, we found that filter binding, which does not require a tag, can also be used to recover DNA-protein complexes. Antibodies could also be used to recover DNA-protein complexes with or without a tag. Our experiments were performed without crosslinking, but for weaker binding proteins, where initially bound fragments might be lost in wash steps, crosslinking protein to DNA could be used in a more standard ChIP-type experiment, either with immunoprecipitation, affinity purification, or filter binding of crosslinked protein-DNA complexes. Comparing *in vitro* studies of genomic binding using the IDAP-Seq method with more traditional *in vivo* ChIP experiments should provide valuable clues about how the activities of DNA binding proteins are modulated in cells.

## Materials and Methods

Purification of hexa-histidine-tagged DnaA, preparation of sheared genomic DNA, refinement of the catalog of DnaA binding regions, and development of the DnaA box PSSM are described in S1 Text.

### DnaA-his

*B*. *subtilis* DnaA with the amino acids AAALEHHHHHH added to the C-terminus of DnaA was purified from *E*. *coli* (S1 Text). Final DnaA-his preparations were typically 98% pure. A similarly his-tagged DnaA (with 12 instead of six histidines) is functional *in vivo* [9].

### Affinity purification of DNA:DnaA-his complexes

Binding reactions (in 250 μl) were done with DnaA-his (at concentrations of 0, 55 nM, 140 nM, 550 nM, 1.4 μM, and 4.1 μM) and sheared genomic DNA (0.2 mg/ml) in 40 mM HEPES-KOH, pH 7.6, 150 mM potassium glutamate, 2.5 mM ATP or ADP, 10 mM magnesium acetate, 0.2 mM DTT, 50 μg/ml BSA, 0.1 mM EDTA, 20% glycerol, and 4% sucrose for 30 min at room temperature. Genomic DNA was purified from a *dnaBts* mutant [29, 43, 44]. DnaA-his was bound with nucleotide by preincubating in storage buffer with 2.5 mM ATP or ADP on ice for two hours immediately before using in binding reactions, as described previously [23].

Each reaction was mixed with 100 μl Talon Co^+^ resin (Clontech) pre-equilibrated with equilibration/wash buffer (40 mM HEPES-KOH, pH 7.6, 150 mM potassium glutamate, 2.5 mM ATP or ADP, 10 mM magnesium acetate, 50 μg/ml BSA, 20% glycerol) and rotated for 30 min at room temperature. Each mixture was transferred to a Poly-Prep column (Bio-Rad, Hercules, CA), and washed three times with 1 ml equilibration/wash buffer, with care taken to ensure that all washes were done under virtually identical conditions. Complexes of DnaA-his bound to DNA were eluted by adding 0.5 ml ChIP elution buffer (50 mM Tris-HCl, pH 8.0, 10 mM EDTA, 1% SDS), capping the bottoms and covering the tops of the columns tightly with foil, and incubating at 65°C for 15 min. The eluate was collected, and the resin was washed twice with 200 μl ChIP elution buffer to recover all of the eluted DNA. The recovered DNA was purified using a QiaQuick PCR purification kit (Qiagen).

### DNA sequencing

Sample preparation, including incorporation of a 3’ barcode, selection of 200–400 bp fragments (after addition of adaptors and amplification), and single read sequencing (40 nt) on an Illumina HiSeq were performed by the MIT BioMicro Center.

### Seq data processing and peak calling algorithm

Alignment of DNA fragments bound by DnaA-his to the genome of AG1839 (a.k.a., KPL69; GenBank accession number CP008698) [29] was performed using Bowtie [45], with adjustments to compensate for the fact that the chromosome is circular. Peak calling on the 1.4 μM and 4.1 μM ATP-DnaA-his data was done using cisGenome v. 2.0 [46], and in some cases PeakSplitter [47], and visualized in the genome browser MochiView [48] for manual refinement (see S1 Text for details). The genome position of the summit of each peak was determined using data from the 4.1 μM ATP-DnaA-his binding reaction, because the peaks (especially the weaker ones) were better defined at this DnaA concentration. Seq data are available at NCBI under accession SRX648534.

### Quantitation of binding and determination of apparent binding constants

To determine the amount of DNA bound by DnaA-his for each chromosomal region, we determined the number of sequence reads across that region. Each sequence read (mapped to the chromosome using Bowtie) was computationally extended by the estimated average fragment length of 250 base pairs (presented schematically in S3A and S3B Fig). The relative coverage at each bp along the chromosome was obtained by summing the number of fragments on both the positive and negative strands that are inferred to span that position (S3C Fig). Custom R scripts were used for these steps. The resulting coverage map allowed different regions along the chromosome to be compared for any given sample.

To compare individual loci under a variety of binding conditions (e.g., ATP v. ADP, or at different concentrations of DnaA), we normalized the number of sequence reads (coverage map amplitudes) to the total amount of DNA recovered in each reaction (S1 Text, S3D–S3G Fig). The amount of DNA that was recovered in each sample increased with increasing amounts DnaA (S3F Fig), due to 1) increases in background binding, 2) increases in binding at regions that have not yet reached saturation, and 3) binding at new weaker binding regions. Measurements of the maximum binding following normalization vs. DNA concentration gave data that could be fit to a binding curve (S3H Fig).

Apparent K_d_’s were determined using Prism5 (GraphPad Software). Data were fit to the equation y = (B_max_)(x^n^)/(K_d_^n^ + x^n^), where B_max_ is maximum binding, x is the DnaA-his concentration, y was the amount of binding observed, and n is the Hill constant. For each binding region, the position of the peak was determined in the 4.1 μM DnaA dataset, and the peak height at the same position was determined for the lower DnaA concentrations and used as the amount of binding. For binding regions that approached saturation, B_max_ was fitted from the binding data. For several binding regions, B_max_ could be determined for ATP- but not ADP-DnaA-his binding. In these cases, the B_max_ determined for ATP-DnaA-his was used to fit the ADP-DnaA-his data. In all other instances, B_max_ of 0.8 was used to determine an apparent K_d_.

### Annotation of DnaA boxes

DnaA boxes in the *B*. *subtilis* genome were annotated using the PSSM generated as part of this study (S1 Text). This PSSM was used to search the genome sequence of AG1839 genome using RSAT [49] with a p-value cutoff of ≤ 0.0015. Where overlapping DnaA boxes were detected, the one with the higher p-value was discarded. This collection of DnaA boxes was used in all figures and tables. A “DnaA Box Score” for the binding regions was calculated by summing the negative log of the P-value from the PSSM for each binding region.

### *In vivo* DnaA ChIP-PCRs and strains used

DnaA binding to specific chromosomal regions *in vivo* was determined by ChIP followed by quantitative PCR (ChIP-PCR). Wild type (AG174; genotype: *trp*, *phe*) and *rok* null mutant {HM57; genotype: *trp*, *phe*, *rok*::pDG641rok (*mls*)} cells were grown at 37°C in LB medium. (The *rok* null mutation is an integration of plasmid pDG641rok into *rok* by single crossover, disrupting the *rok* open reading frame.) Cells in mid-exponential phase were treated with 1% formaldehyde for 20 min to crosslink protein and DNA. Crosslinking was quenched by adding glycine (0.22 M). Preparation of lysates and immunoprecipitations were done essentially as describe previously [50]. DnaA was immunoprecipitated with rabbit polyclonal antiserum and the DNA was recovered using a QiaQuick PCR purification kit (Qiagen). Quantitative PCR was performed on a Roche LightCycler 480, with reduced annealing (48°C), extension (68°C), and acquisition (63°C) temperatures to compensate for the low melting temperatures of many of the loci being examined. Fold-enrichments were calculated as described in [51], using *nicK*, a region in ICE*Bs1* [52] that does not bind DnaA, for normalization. The primer pairs used are listed in S5 Table.

## Supporting Information

S1 Fig Catalog of all 269 binding regions detected at 1.4 μM DnaA. Each binding region was identified using cisGenome [46] and then manually validated and refined (Materials and Methods). Panel numbers 1–269 correspond to the peak numbers in S1 Table. The two binding regions from *oriC* (upstream of *dnaA*, and between *dnaA* and *dnaN*) are shown first, followed by binding regions in order of the amount of DNA that was recovered from each region at 1.4 μM ATP-DnaA-his (S1 Table). The *left side* of each panel shows the binding data along an 800 bp chromosomal region centered on the position of maximum binding (indicated by the dashed vertical red line). The labeled x-axis indicates genomic coordinates from strain AG1839. *Top left*: the overall amount of binding inferred from the sequence data. black curve, binding with 4.1 μM DnaA; red curve, binding with no added DnaA. Relative binding (y-axis) was normalized to a global maximum of 1 at 1.4 μM ATP-DnaA. *Middle left*: a histogram of the number of sequence reads (from 4.1 μM ATP-DnaA-his) that start at each nucleotide; blue, sequence reads mapping to the top strand; green, sequence reads mapping to the bottom strand. Red circles indicate potential DnaA binding sites predicted using the PSSM described in this paper. *Bottom left*: genes with arrowheads indicating the direction of transcription. *Top right*: sequence of each of the putative DnaA boxes identified by the PSSM and shown in the middle left. For regions with >5 putative DnaA boxes, the complete list is in S5 Table). *Bottom right*: binding curves plotting the amount of DNA recovered as a function of the concentration of DnaA-his. ATP-DnaA-his, open circles and dashed lines; ADP-DnaA-his, filled triangles and dotted lines.
(PDF)

S2 Fig The C-terminal DNA binding domain of DnaA is required for association of DnaA with chromosomal regions *in vitro*. Binding reactions were performed under the same conditions as for DnaA-his, except that 4.1 μM DnaA∆C-his was used. DnaA∆C lacks the C-terminal 91 amino acids that are required for DNA binding. A reaction containing full-length DnaA-his was performed in parallel. The binding reactions contained 2.5 mM ATP. The recovered DNA was assayed using qPCR, with the primers indicated in S5 Table. The following loci were assayed (peak numbers refer to those in S1 Fig and S1 Table): *cotH* (peak #198), *ypfD* (peak #235), *yphF* (too weak to be called as a peak at 1.4 μM DnaA but clearly discernible at 4.1 μM), *ydiO* (peak #250), *rplB* (peak #10), *dnaA* (peak #1), and *nicK*, a control region that does not bind DnaA.
(TIFF)

S3 Fig Method for quantitating binding data over a range of DnaA-his concentrations. In panels *A-C*, a schematic representation using a toy dataset shows how deep sequencing data were converted to coverage along the chromosome. *(A)* start positions of sequence reads are plotted as histograms, and are shown clustered around a DnaA binding site depicted by the red dotted line. *(B)* Each read was extended in the appropriate direction (rightward for reads corresponding to the top strand, and leftward for reads corresponding to the bottom strand) by the average fragment length of 250 bp. *(C)* The number of fragments containing each nucleotide along the genome is determined, yielding the relative coverage along the genome. Although this allows for comparison between different genomic loci in the same binding reaction, it does not support comparison between different binding reactions (*i*.*e*., comparing ATP and ADP, or comparing different concentrations of DnaA-his.) *(D)* Actual sequence data from the *sda* promoter region from samples containing the indicated concentrations of ATP-DnaA-his. The y-axis scale for each of the samples is the same. The same total number of reads was mapped for each binding reaction, but the number of reads mapping to the *sda* promoter region (and other high-affinity DnaA binding regions) decreased at the two highest concentrations of DnaA-his. This is because at these DnaA concentrations, binding to *sda* has already saturated, while an increasing portion of the reads map to weaker binding regions, and there is also an increase in background binding. *(E)* The relative coverage along the same region as in D, obtained by extending the reads by the average read length and summing the number of extended reads spanning each position, as depicted in A, B, and C. *(F)* The amount of DNA recovered in each binding reaction (prior to any preparation steps for deep sequencing) was determined. *(G)* The coverage in panel *E*, which was calculated using the same number of reads for each sample, was scaled so that the total coverage (summed over the whole genome) is proportional to the total amount of DNA recovered. The entire data set (comprised of 12 samples—six with ATP and six with ADP) was then re-scaled so that the global maximum was 1. *(H)* The coverage at the peak summit (indicated with a dashed red line in panels D, E, and G) was plotted as a function of DnaA concentration, and used to determine binding constants.
(TIFF)

S4 Fig Correlation between DnaA binding the predicted sites based on the PSSM. The amount of binding observed at each binding site using 4.1 μM ATP-DnaA-his is plotted as a function of the predicted strength of the DnaA box based on the PSSM. The DnaA box score for each binding region was calculated by summing the negative logs of the p-values from the PSSM for each predicted DnaA box in a 200 bp window centered on the peak summit. The line shown is a linear least squares regression fit of the data.
(TIFF)

S5 Fig Overview of genomic binding by different concentrations of ADP-DnaA-his. The relative amount of binding by ADP-DnaA-his is plotted on the y-axis versus the position along the chromosome on the x-axis. The 4.2 mb circular chromosome is depicted linearly such that the origin of replication is near the middle of the x-axis at 4.2 mb and 0 mb. The concentration of ADP-DnaA-his in each binding reaction was *(A)* no DnaA; *(B)* 55 nM; *(C)* 140 nM; *(D)* 550 nM; *(E)* 1.4 μM; *(F)* 4.1 μM. The binding profiles along the chromosome were determined by deep sequencing the DNA recovered in each binding reaction. Binding data are presented in 200 nt bins, with the maximum binding amplitude in each bin drawn. The amplitudes of each binding reaction were adjusted so that the total amount of binding is proportional to the amount of DNA recovered in that binding reaction (see S2 Fig for details.) The data were normalized together with binding data using ATP-DnaA-his (Fig 1) so that maximum binding had an amplitude of 1.
(TIFF)

S6 Fig DnaA protein levels were similar in wild type and *rok*-null cells. Whole cell lysates from wild type (*rok*+; AG174) and *rok* null mutant (*rok*-; HM57) cells grown to mid-exponential phase in LB medium were subjected to SDS polyacrylamide gel electrophoresis, and duplicate gels were analyzed by *(A)* Coomassie staining, and *(B)* western blotting with a DnaA antibody. Lane 1, AG174 (wild type), Lane 2, HM57 (*rok* null mutant). The position of the DnaA band is indicated with an asterisk. Quantitation of total Coomassie staining and DnaA levels was performed using near-infrared detection on an Odyssey imager (Licor). *(C)* The amount of DnaA relative to total protein was calculated, and normalized to a value of 1 for wild type. The mean of four replicates is presented, with error bars to indicate the standard deviation. The observed 1.16X difference in the means is not statistically significant (P = 0.28).
(TIFF)

S1 Table List of 269 chromosomal regions bound at 1.4 μM DnaA. (PDF)

S2 Table DnaA boxes used to determine the PSSM. The 5' and 3' nucleotide position (AG1839 genome coordinates), DNA strand (+/-), and sequence of each of the 150 DnaA boxes is shown.
(PDF)

S3 Table Nucleotide frequencies for the DnaA box PSSM. Nucleotide frequencies for each position in the 150 DnaA boxes in S2 Table are presented.
(PDF)

S4 Table Predicted DnaA boxes in all 269 binding regions. All DnaA boxes drawn in S1 Fig are presented. Peak numbers are the same as in S1 Table and correspond to the panel numbers in S1 Fig. Start coordinates correspond to the genome sequence of strain AG1839 [29]. P values are from RSAT [49]. The distance from the center nucleotide of the DnaA box to the peak summit (found in S1 Table) is shown.
(PDF)

S5 Table Primers used for qPCR. (PDF)

S1 Text Contains expanded Materials and Methods. (PDF)

## References

[1] Kaguni JM. DnaA: controlling the initiation of bacterial DNA replication and more. Annu Rev Microbiol. 2006;60:351–75. 16753031 10.1146/annurev.micro.60.080805.142111

[2] Mott ML, Berger JM. DNA replication initiation: mechanisms and regulation in bacteria. Nat Rev Microbiol. 2007 5;5(5):343–54. 17435790 10.1038/nrmicro1640

[3] Leonard AC, Grimwade JE. Regulating DnaA complex assembly: it is time to fill the gaps. Curr Opin Microbiol. 2010 12;13(6):766–72. 10.1016/j.mib.2010.10.001 21035377 PMC3005629

[4] Kawakami H, Katayama T. DnaA, ORC, and Cdc6: similarity beyond the domains of life and diversity. Biochem Cell Biol. 2010 2;88(1):49–62. 10.1139/o09-154 20130679

[5] Duderstadt KE, Berger JM. AAA+ ATPases in the initiation of DNA replication. Crit Rev Biochem Mol Biol. 2008 May-Jun;43(3):163–87. 10.1080/10409230802058296 18568846

[6] Katayama T, Ozaki S, Keyamura K, Fujimitsu K. Regulation of the replication cycle: conserved and diverse regulatory systems for DnaA and *oriC* . Nat Rev Microbiol. 2010 3;8(3):163–70. 10.1038/nrmicro2314 20157337

[7] Burkholder WF, Kurtser I, Grossman AD. Replication initiation proteins regulate a developmental checkpoint in *Bacillus subtilis* . Cell. 2001 1 26;104(2):269–79. 11207367 10.1016/s0092-8674(01)00211-2

[8] Breier AM, Grossman AD. Dynamic association of the replication initiator and transcription factor DnaA with the *Bacillus subtilis* chromosome during replication stress. J Bacteriol. 2009 1;191(2):486–93. 10.1128/JB.01294-08 19011033 PMC2620820

[9] Ishikawa S, Ogura Y, Yoshimura M, Okumura H, Cho E, Kawai Y, et al Distribution of stable DnaA-binding sites on the *Bacillus subtilis* genome detected using a modified ChIP-chip method. DNA Res. 2007 8;14(4):155–68. 17932079 10.1093/dnares/dsm017 PMC2533591

[10] Hoover SE, Xu W, Xiao W, Burkholder WF. Changes in DnaA-dependent gene expression contribute to the transcriptional and developmental response of *Bacillus subtilis* to manganese limitation in Luria-Bertani medium. J Bacteriol. 2010 8;192(15):3915–24. 10.1128/JB.00210-10 20511500 PMC2916362

[11] Veening JW, Murray H, Errington J. A mechanism for cell cycle regulation of sporulation initiation in *Bacillus subtilis* . Genes Dev. 2009 8 15;23(16):1959–70. 10.1101/gad.528209 19684115 PMC2725940

[12] Smits WK, Merrikh H, Bonilla CY, Grossman AD. Primosomal proteins DnaD and DnaB are recruited to chromosomal regions bound by DnaA in *Bacillus subtilis* . J Bacteriol. 2011 11 19;193(3):640–8. 10.1128/JB.01253-10 21097613 PMC3021214

[13] Cho E, Ogasawara N, Ishikawa S. The functional analysis of YabA, which interacts with DnaA and regulates initiation of chromosome replication in *Bacillus subtils* . Genes Genet Syst. 2008 4;83(2):111–25. 18506095 10.1266/ggs.83.111

[14] Fuller RS, Funnell BE, Kornberg A. The dnaA protein complex with the *E*. *coli* chromosomal replication origin (*oriC*) and other DNA sites. Cell. 1984 10;38(3):889–900. 6091903 10.1016/0092-8674(84)90284-8

[15] Simmons LA, Felczak M, Kaguni JM. DnaA Protein of *Escherichia coli*: oligomerization at the *E*. *coli* chromosomal origin is required for initiation and involves specific N-terminal amino acids. Mol Microbiol. 2003 8;49(3):849–58. 12864864 10.1046/j.1365-2958.2003.03603.x

[16] Miller DT, Grimwade JE, Betteridge T, Rozgaja T, Torgue JJ, Leonard AC. Bacterial origin recognition complexes direct assembly of higher-order DnaA oligomeric structures. Proc Natl Acad Sci U S A. 2009 11 3;106(44):18479–84. 10.1073/pnas.0909472106 19833870 PMC2773971

[17] Felczak MM, Kaguni JM. The box VII motif of *Escherichia coli* DnaA protein is required for DnaA oligomerization at the *E*. *coli* replication origin. J Biol Chem. 2004 12 3;279(49):51156–62. 15371441 10.1074/jbc.M409695200

[18] Krause M, Ruckert B, Lurz R, Messer W. Complexes at the replication origin of *Bacillus subtilis* with homologous and heterologous DnaA protein. J Mol Biol. 1997 12 5;274(3):365–80. 9405146 10.1006/jmbi.1997.1404

[19] Speck C, Weigel C, Messer W. ATP- and ADP-dnaA protein, a molecular switch in gene regulation. EMBO J. 1999 11 1;18(21):6169–76. 10545126 10.1093/emboj/18.21.6169 PMC1171680

[20] Skarstad K, Katayama T. Regulating DNA replication in bacteria. Cold Spring Harb Perspect Biol. 2013 4;5(4):a012922 10.1101/cshperspect.a012922 23471435 PMC3683904

[21] Leonard AC, Grimwade JE. Regulation of DnaA assembly and activity: taking directions from the genome. Annu Rev Microbiol. 2011;65:19–35. 10.1146/annurev-micro-090110-102934 21639790 PMC4075013

[22] Merrikh H, Grossman AD. Control of the replication initiator DnaA by an anti-cooperativity factor. Mol Microbiol. 2011 10;82(2):434–46. 10.1111/j.1365-2958.2011.07821.x 21895792 PMC3192265

[23] Bonilla CY, Grossman AD. The primosomal protein DnaD inhibits cooperative DNA binding by the replication initiator DnaA in *Bacillus subtilis* . J Bacteriol. 2012 9;194(18):5110–7. 10.1128/JB.00958-12 22821970 PMC3430336

[24] Scholefield G, Errington J, Murray H. Soj/ParA stalls DNA replication by inhibiting helix formation of the initiator protein DnaA. EMBO J. 2012 3 21;31(6):1542–55. 10.1038/emboj.2012.6 22286949 PMC3321191

[25] Majerczyk CD, Dunman PM, Luong TT, Lee CY, Sadykov MR, Somerville GA, et al Direct targets of CodY in *Staphylococcus aureus* . J Bacteriol. 2010 6;192(11):2861–77. 10.1128/JB.00220-10 20363936 PMC2876493

[26] Belitsky BR, Sonenshein AL. Genome-wide identification of *Bacillus subtilis* CodY-binding sites at single-nucleotide resolution. Proc Natl Acad Sci U S A. 2013 4 23;110(17):7026–31. 10.1073/pnas.1300428110 23569278 PMC3637721

[27] Chateau A, van Schaik W, Joseph P, Handke LD, McBride SM, Smeets FM, et al Identification of CodY targets in *Bacillus anthracis* by genome-wide *in vitro* binding analysis. J Bacteriol. 2013 3;195(6):1204–13. 10.1128/JB.02041-12 23292769 PMC3591999

[28] Goranov AI, Katz L, Breier AM, Burge CB, Grossman AD. A transcriptional response to replication status mediated by the conserved bacterial replication protein DnaA. Proc Natl Acad Sci U S A. 2005 9 6;102(36):12932–7. 16120674 10.1073/pnas.0506174102 PMC1200305

[29] Smith JL, Goldberg JM, Grossman AD. Complete genome sequences of *Bacillus subtilis subtilis* laboratory strains JH642 (AG174) and AG1839. Genome Announc. 2014;2(4):e00663–14. 10.1128/genomeA.00663-14 24994804 PMC4082004

[30] Fukuoka T, Moriya S, Yoshikawa H, Ogasawara N. Purification and characterization of an initiation protein for chromosomal replication, DnaA, in *Bacillus subtilis* . J Biochem (Tokyo). 1990 5;107(5):732–9. 2168872 10.1093/oxfordjournals.jbchem.a123117

[31] Messer W. The bacterial replication initiator DnaA. DnaA and *oriC*, the bacterial mode to initiate DNA replication. FEMS Microbiol Rev. 2002 11;26(4):355–74. 12413665 10.1111/j.1574-6976.2002.tb00620.x

[32] Crooks GE, Hon G, Chandonia JM, Brenner SE. WebLogo: a sequence logo generator. Genome Res. 2004 6;14(6):1188–90. 15173120 10.1101/gr.849004 PMC419797

[33] Goranov AI, Breier AM, Merrikh H, Grossman AD. YabA of *Bacillus subtilis* controls DnaA-mediated replication initiation but not the transcriptional response to replication stress. Mol Microbiol. 2009 10;74(2):454–66. 10.1111/j.1365-2958.2009.06876.x 19737352 PMC2823125

[34] Scholefield G, Murray H. YabA and DnaD inhibit helix assembly of the DNA replication initiation protein DnaA. Mol Microbiol. 2013 8 5;90(1):147–59. 10.1111/mmi.12353 23909787

[35] Rahn-Lee L, Gorbatyuk B, Skovgaard O, Losick R. The conserved sporulation protein YneE inhibits DNA replication in *Bacillus subtilis* . J Bacteriol. 2009 6;191(11):3736–9. 10.1128/JB.00216-09 19329632 PMC2681902

[36] Wagner JK, Marquis KA, Rudner DZ. SirA enforces diploidy by inhibiting the replication initiator DnaA during spore formation in *Bacillus subtilis* . Mol Microbiol. 2009 9;73(5):963–74. 10.1111/j.1365-2958.2009.06825.x 19682252 PMC2992877

[37] Rahn-Lee L, Merrikh H, Grossman AD, Losick R. The sporulation protein SirA inhibits the binding of DnaA to the origin of replication by contacting a patch of clustered amino acids. J Bacteriol. 2011 3;193(6):1302–7. 10.1128/JB.01390-10 21239581 PMC3067619

[38] Zakrzewska-Czerwinska J, Jakimowicz D, Zawilak-Pawlik A, Messer W. Regulation of the initiation of chromosomal replication in bacteria. FEMS Microbiol Rev. 2007 7;31(4):378–87. 17459114 10.1111/j.1574-6976.2007.00070.x

[39] Ruvolo MV, Mach KE, Burkholder WF. Proteolysis of the replication checkpoint protein Sda is necessary for the efficient initiation of sporulation after transient replication stress in *Bacillus subtilis* . Mol Microbiol. 2006 6;60(6):1490–508. 16796683 10.1111/j.1365-2958.2006.05167.x

[40] Okumura H, Yoshimura M, Ueki M, Oshima T, Ogasawara N, Ishikawa S. Regulation of chromosomal replication initiation by *oriC*-proximal DnaA-box clusters in *Bacillus subtilis* . Nucleic Acids Res. 2012 1;40(1):220–34. 10.1093/nar/gkr716 21911367 PMC3245932

[41] Nicolas P, Mader U, Dervyn E, Rochat T, Leduc A, Pigeonneau N, et al Condition-dependent transcriptome reveals high-level regulatory architecture in *Bacillus subtilis* . Science. 2012 3 2;335(6072):1103–6. 10.1126/science.1206848 22383849

[42] Smits WK, Grossman AD. The transcriptional regulator Rok binds A+T-rich DNA and is involved in repression of a mobile genetic element in *Bacillus subtilis* . PLoS Genet. 2010;6(11):e1001207 10.1371/journal.pgen.1001207 21085634 PMC2978689

[43] Callister H, Le Mesurier S, Wake RG. Initiation of deoxyribonucleic acid replication in germinating spores of *Bacillus subtilis* 168 carrying the *dnaB* (Ts)134 mutation. J Bacteriol. 1977 6;130(3):1030–7. 405369 10.1128/jb.130.3.1030-1037.1977 PMC235324

[44] Rokop ME, Auchtung JM, Grossman AD. Control of DNA replication initiation by recruitment of an essential initiation protein to the membrane of *Bacillus subtilis* . Mol Microbiol. 2004 6;52(6):1757–67. 15186423 10.1111/j.1365-2958.2004.04091.x

[45] Langmead B, Trapnell C, Pop M, Salzberg SL. Ultrafast and memory-efficient alignment of short DNA sequences to the human genome. Genome Biol. 2009;10(3):R25 10.1186/gb-2009-10-3-r25 19261174 PMC2690996

[46] Ji H, Jiang H, Ma W, Johnson DS, Myers RM, Wong WH. An integrated software system for analyzing ChIP-chip and ChIP-seq data. Nat Biotechnol. 2008 11;26(11):1293–300. 10.1038/nbt.1505 18978777 PMC2596672

[47] Salmon-Divon M, Dvinge H, Tammoja K, Bertone P. PeakAnalyzer: genome-wide annotation of chromatin binding and modification loci. BMC Bioinformatics. 2010;11:415 10.1186/1471-2105-11-415 20691053 PMC2923140

[48] Homann OR, Johnson AD. MochiView: versatile software for genome browsing and DNA motif analysis. BMC Biol. 2010;8:49 10.1186/1741-7007-8-49 20409324 PMC2867778

[49] Thomas-Chollier M, Defrance M, Medina-Rivera A, Sand O, Herrmann C, Thieffry D, et al RSAT 2011: regulatory sequence analysis tools. Nucleic Acids Res. 2011 7;39(Web Server issue):W86–91. 10.1093/nar/gkr377 21715389 PMC3125777

[50] Lin DC, Grossman AD. Identification and characterization of a bacterial chromosome partitioning site. Cell. 1998 3 6;92(5):675–85. 9506522 10.1016/s0092-8674(00)81135-6

[51] Merrikh H, Machón C, Grainger WH, Grossman AD, Soultanas P. Co-directional replication-transcription conflicts lead to replication restart. Nature. 2011 2 24;470(7335):554–7. 10.1038/nature09758 21350489 PMC3059490

[52] Lee CA, Grossman AD. Identification of the origin of transfer (*oriT*) and DNA relaxase required for conjugation of the integrative and conjugative element ICE*Bs1* of *Bacillus subtilis* . J Bacteriol. 2007 10;189(20):7254–61. 17693500 10.1128/JB.00932-07 PMC2168444

