# Supplementary material for: In Vitro Whole Genome DNA Binding Analysis of the Bacterial Replication Initiator and Transcription Factor DnaA
Source: PLoS Genet. 2015 May 28;11(5):e1005258. doi: 10.1371/journal.pgen.1005258 (PMC4447404; doi:10.1371/journal.pgen.1005258)
Supplement: S1 Fig — Each binding region was identified using cisGenome [46] and then manually validated and refined (Materials and Methods). Panel numbers 1–269 correspond to the peak numbers in S1 Table. The two binding regions from oriC (upstream of dnaA, and between dnaA and dnaN) are shown first, followed by binding regions in order of the amount of DNA that was recovered from each region at 1.4 μM ATP-DnaA-his (S1 Table). The left side of each panel shows the binding data along an 800 bp chromosomal region centered on the position of maximum binding (indicated by the dashed vertical red line). The labeled x-axis indicates genomic coordinates from strain AG1839. Top left: the overall amount of binding inferred from the sequence data. black curve, binding with 4.1 μM DnaA; red curve, binding with no added DnaA. Relative binding (y-axis) was normalized to a global maximum of 1 at 1.4 μM ATP-DnaA. Middle left: a histogram of the number of sequence reads (from 4.1 μM ATP-DnaA-his) that start at each nucleotide; blue, sequence reads mapping to the top strand; green, sequence reads mapping to the bottom strand. Red circles indicate potential DnaA binding sites predicted using the PSSM described in this paper. Bottom left: genes with arrowheads indicating the direction of transcription. Top right: sequence of each of the putative DnaA boxes identified by the PSSM and shown in the middle left. For regions with >5 putative DnaA boxes, the complete list is in S5 Table). Bottom right: binding curves plotting the amount of DNA recovered as a function of the concentration of DnaA-his. ATP-DnaA-his, open circles and dashed lines; ADP-DnaA-his, filled triangles and dotted lines. (PDF) [file pgen.1005258.s001.pdf]

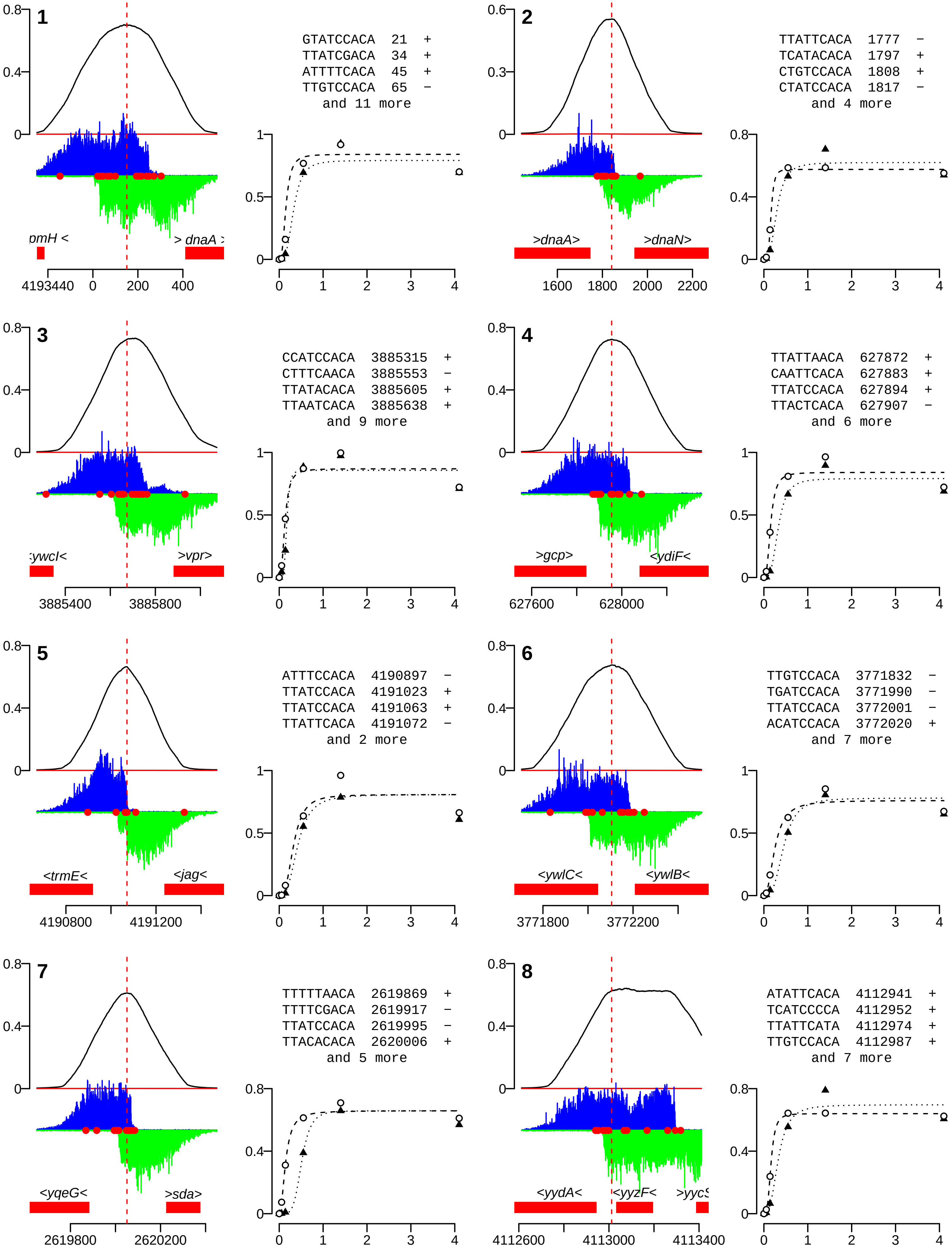
1
GTATCCACA 21 +
TTATCGACA 34 +
ATTTTCACA 45 +
TTGTCCACA 65 −
and 11 more
pmH <
> dnaA >
4193440 0 200 400
2
TTATTCACA 1777 −
TCATACACA 1797 +
CTGTCCACA 1808 +
CTATCCACA 1817 −
and 4 more
>dnaA>
>dnaN>
1600 1800 2000 2200
3
CCATCCACA 3885315 +
CTTTCAACA 3885553 −
TTATACACA 3885605 +
TTAATCACA 3885638 +
and 9 more
ywcI<
>vpr>
3885400 3885800
4
TTATTAACA 627872 +
CAATTCACA 627883 +
TTATCCACA 627894 +
TTACTCACA 627907 −
and 6 more
>gcp>
<ydiF<
627600 628000
5
ATTTCCACA 4190897 −
TTATCCACA 4191023 +
TTATCCACA 4191063 +
TTATTCACA 4191072 −
and 2 more
<trmE<
<jag<
4190800 4191200
6
TTGTCCACA 3771832 −
TGATCCACA 3771990 −
TTATCCACA 3772001 −
ACATCCACA 3772020 +
and 7 more
<ywlC<
<ywlB<
3771800 3772200
7
TTTTTAACA 2619869 +
TTTTCGACA 2619917 −
TTATCCACA 2619995 −
TTACACACA 2620006 +
and 5 more
<yqeG<
>sda>
2619800 2620200
8
ATATTCACA 4112941 +
TCATCCCCA 4112952 +
TTATTCATA 4112974 +
TTGTCCACA 4112987 +
and 7 more
<yydA<
<yyzF<
4112600 4113000 4113400

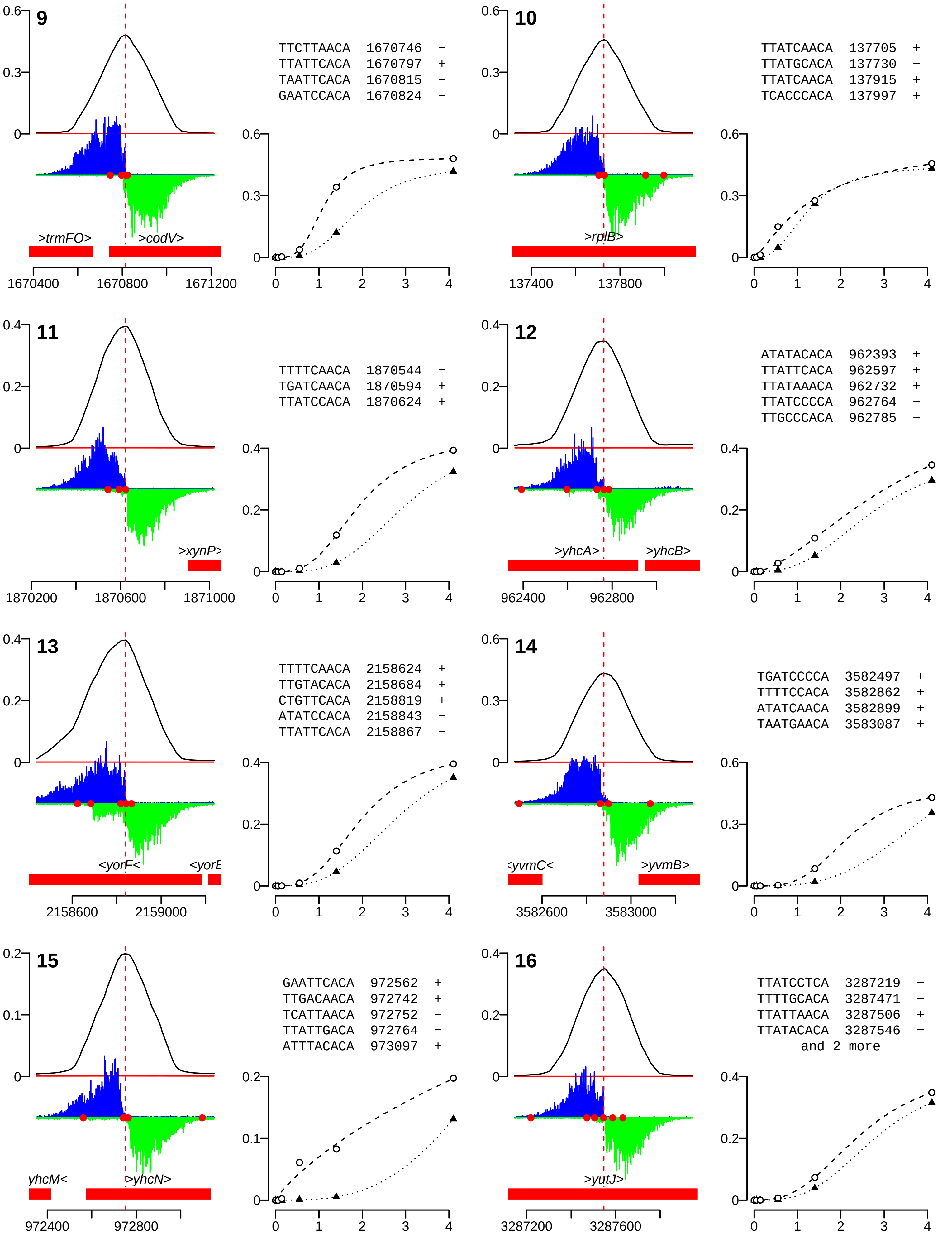

9
0.6
0.3
0
>trmFO>
>codV>
1670400
1670800
1671200
TTCTTAACA 1670746 −
TTATTCACA 1670797 +
TAATTCACA 1670815 −
GAATCCACA 1670824 −
0 1 2 3 4
10
>rplB>
137400
137800
TTATCAACA 137705 +
TTATGCACA 137730 −
TTATCAACA 137915 +
TCACCCACA 137997 +
11
0.4
0.2
>xynP>
1870200
1870600
1871000
TTTTCAACA 1870544 −
TGATCAACA 1870594 +
TTATCCACA 1870624 +
12
>yhcA>
>yhcB>
962400
962800
ATATACACA 962393 +
TTATTCACA 962597 +
TTATAAACA 962732 +
TTATCCCCA 962764 −
TTGCCCACA 962785 −
13
<yorF<
<yorE
2158600
2159000
TTTTCAACA 2158624 +
TTGTACACA 2158684 +
CTGTTCACA 2158819 +
ATATCCACA 2158843 −
TTATTCACA 2158867 −
14
<yvmC<
>yvmB>
3582600
3583000
TGATCCCCA 3582497 +
TTTTCCACA 3582862 +
ATATCAACA 3582899 +
TAATGAACA 3583087 +
15
0.2
0.1
yhcM<
>yhcN>
972400
972800
GAATTCACA 972562 +
TTGACAACA 972742 +
TCATTAACA 972752 −
TTATTGACA 972764 −
ATTTACACA 973097 +
16
>yutJ>
3287200
3287600
TTATCCTCA 3287219 −
TTTTGCACA 3287471 −
TTATTAACA 3287506 +
TTATACACA 3287546 −
and 2 more

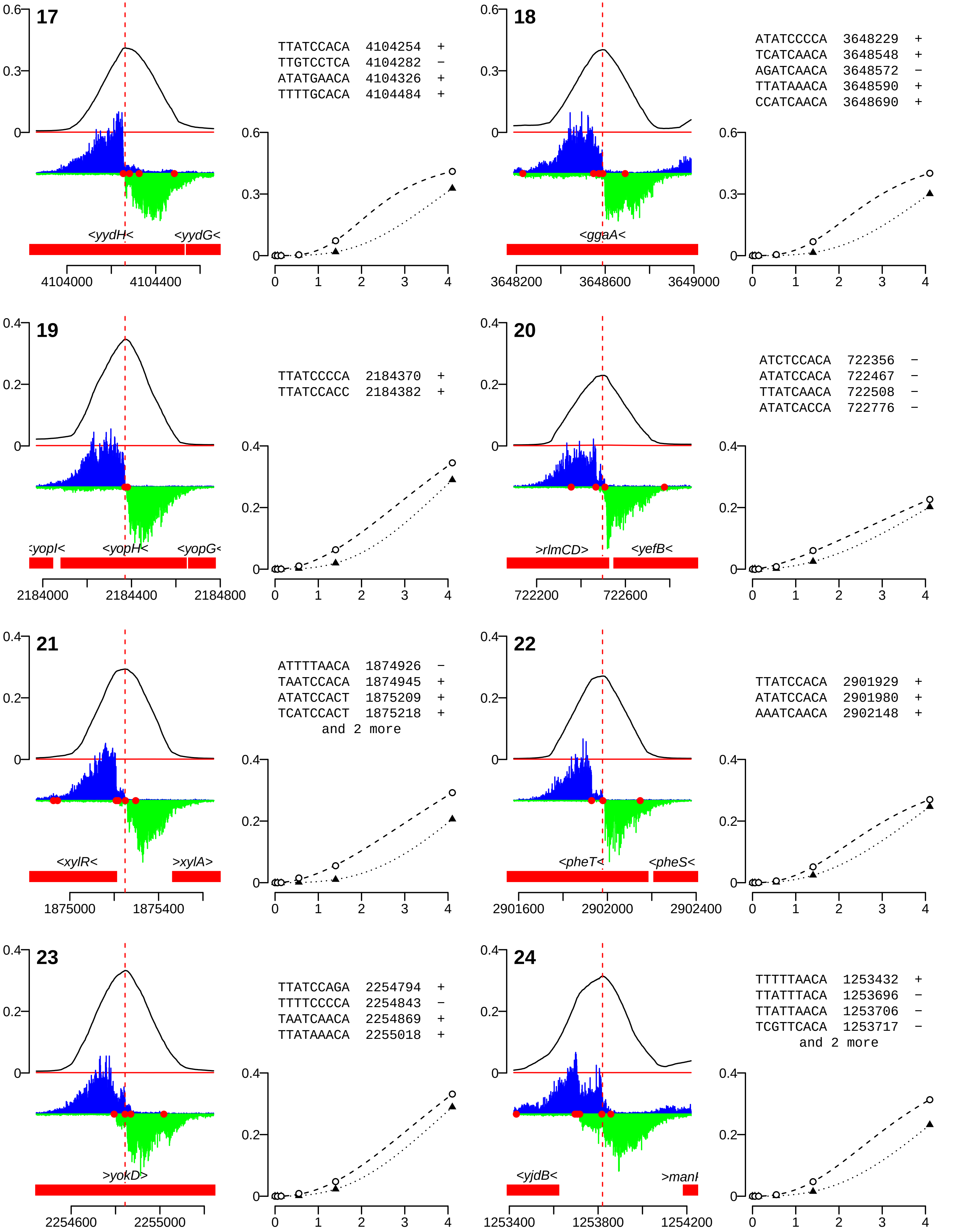
17
TTATCCACA 4104254 +
TTGTCCTCA 4104282 −
ATATGAACA 4104326 +
TTTTGCACA 4104484 +
<yydH<
<yydG<
4104000
4104400
18
ATATCCCCA 3648229 +
TCATCAACA 3648548 +
AGATCAACA 3648572 −
TTATAAACA 3648590 +
CCATCAACA 3648690 +
<ggaA<
3648200
3648600
3649000
19
TTATCCCCA 2184370 +
TTATCCACC 2184382 +
<yopI<
<yopH<
<yopG<
2184000
2184400
2184800
20
ATCTCCACA 722356 −
ATATCCACA 722467 −
TTATCAACA 722508 −
ATATCACCA 722776 −
>rlmCD>
<yefB<
722200
722600
21
ATTTTAACA 1874926 −
TAATCCACA 1874945 +
ATATCCACT 1875209 +
TCATCCACT 1875218 +
and 2 more
<xylR<
>xylA>
1875000
1875400
22
TTATCCACA 2901929 +
ATATCCACA 2901980 +
AAATCAACA 2902148 +
<pheT<
<pheS<
2901600
2902000
2902400
23
TTATCCAGA 2254794 +
TTTTCCCCA 2254843 −
TAATCAACA 2254869 +
TTATAAACA 2255018 +
>yokD>
2254600
2255000
24
TTTTTAACA 1253432 +
TTATTTACA 1253696 −
TTATTAACA 1253706 −
TCGTTCACA 1253717 −
and 2 more
<yjdB<
1253400
1253800
1254200

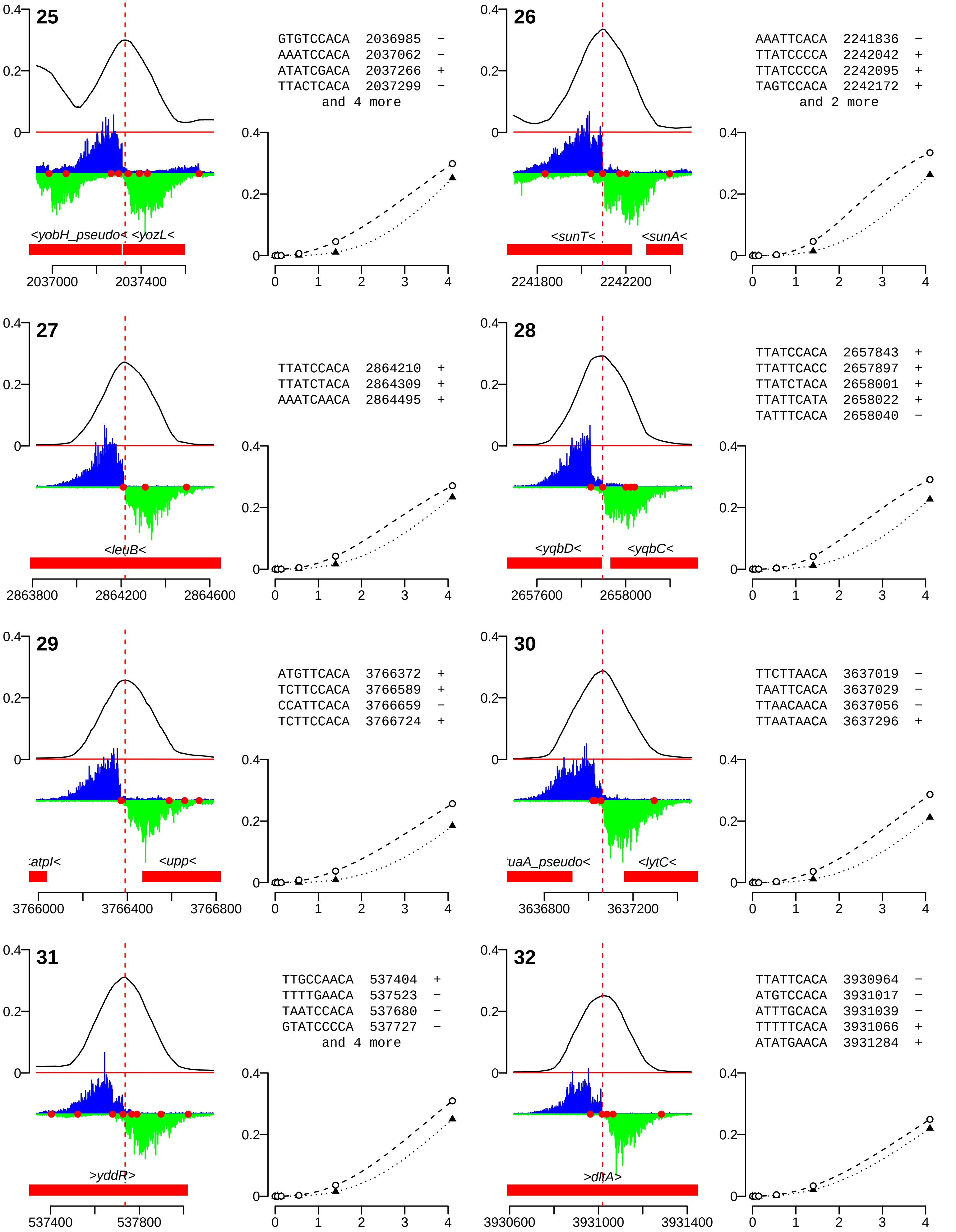
25
GTGTCCACA 2036985 −
AAATCCACA 2037062 −
ATATCGACA 2037266 +
TTACTCACA 2037299 −
and 4 more
<yobH_pseudo< <yozL<
2037000 2037400
26
AAATTCACA 2241836 −
TTATCCCCA 2242042 +
TTATCCCCA 2242095 +
TAGTCCACA 2242172 +
and 2 more
<sunT< <sunA<
2241800 2242200
27
TTATCCACA 2864210 +
TTATCTACA 2864309 +
AAATCAACA 2864495 +
<leuB<
2863800 2864200 2864600
28
TTATCCACA 2657843 +
TTATTCACC 2657897 +
TTATCTACA 2658001 +
TTATTCATA 2658022 +
TATTTCACA 2658040 −
<yqbD< <yqbC<
2657600 2658000
29
ATGTTCACA 3766372 +
TCTTCCACA 3766589 +
CCATTCACA 3766659 −
TCTTCCACA 3766724 +
atpI< <upp<
3766000 3766400 3766800
30
TTCTTAACA 3637019 −
TAATTCACA 3637029 −
TTAACAACA 3637056 −
TTAATAACA 3637296 +
uaA_pseudo< <lytC<
3636800 3637200
31
TTGCCAACA 537404 +
TTTTGAACA 537523 −
TAATCCACA 537680 −
GTATCCCCA 537727 −
and 4 more
>yddR>
537400 537800
32
TTATTCACA 3930964 −
ATGTCCACA 3931017 −
ATTTGCACA 3931039 −
TTTTTCACA 3931066 +
ATATGAACA 3931284 +
>dltA>
3930600 3931000 3931400
0.4
0.2
0
0 1 2 3 4

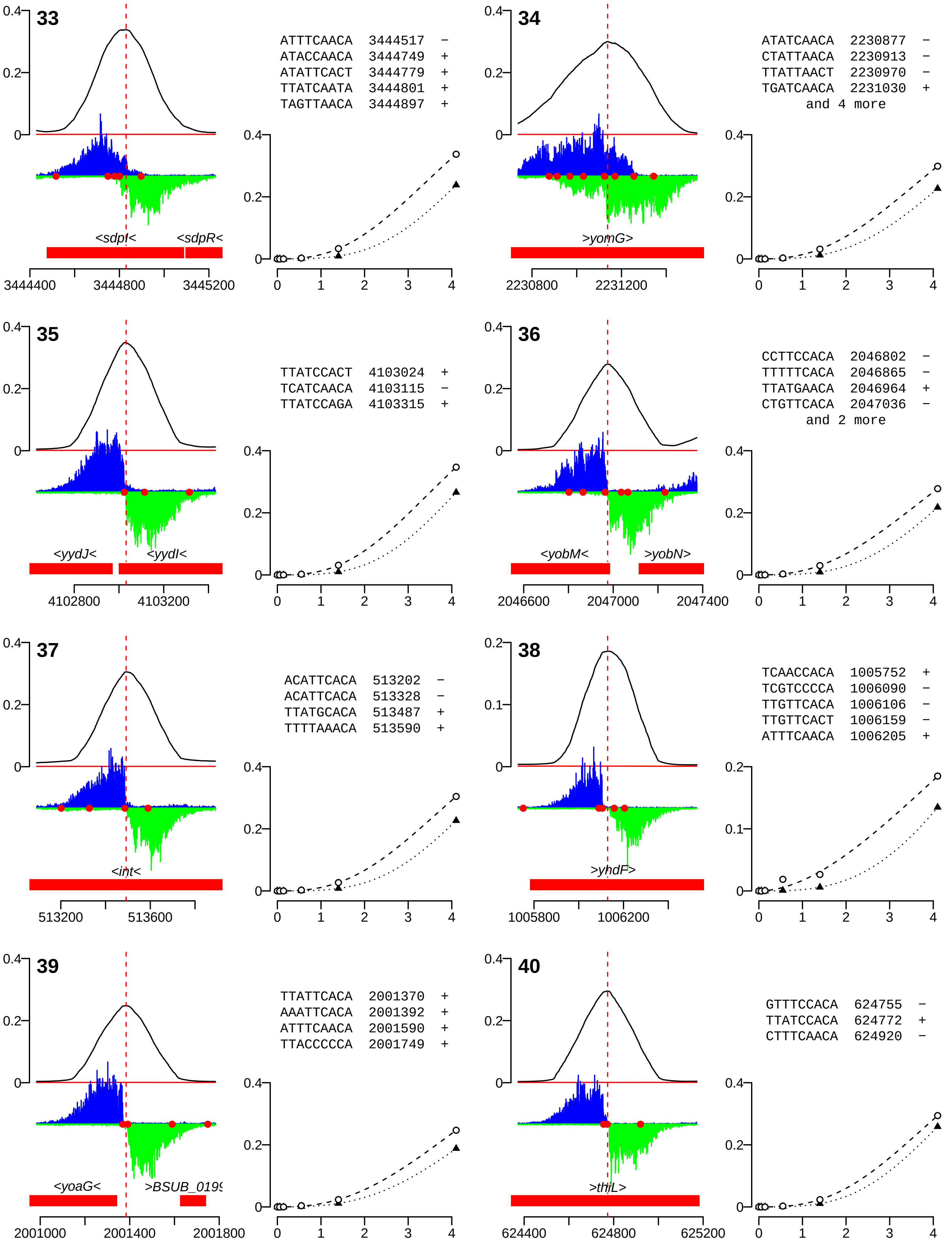

33
ATTTCAACA 3444517 −
ATACCAACA 3444749 +
ATATTCACT 3444779 +
TTATCAATA 3444801 +
TAGTTAACA 3444897 +
<sdpI<
<sdpR<
3444400
3444800
3445200
34
ATATCAACA 2230877 −
CTATTAACA 2230913 −
TTATTAACT 2230970 −
TGATCAACA 2231030 +
and 4 more
>yomG>
2230800
2231200
35
TTATCCACT 4103024 +
TCATCAACA 4103115 −
TTATCCAGA 4103315 +
<yydJ<
<yydI<
4102800
4103200
36
CCTTCCACA 2046802 −
TTTTTCACA 2046865 −
TTATGAACA 2046964 +
CTGTTCACA 2047036 −
and 2 more
<yobM<
>yobN>
2046600
2047000
2047400
37
ACATTCACA 513202 −
ACATTCACA 513328 −
TTATGCACA 513487 +
TTTTAAACA 513590 +
<int<
513200
513600
38
TCAACCACA 1005752 +
TCGTCCCCA 1006090 −
TTGTTCACA 1006106 −
TTGTTCACT 1006159 −
ATTTCAACA 1006205 +
>yhdF>
1005800
1006200
39
TTATTCACA 2001370 +
AAATTCACA 2001392 +
ATTTCAACA 2001590 +
TTACCCCCA 2001749 +
<yoaG<
>BSUB_019
2001000
2001400
2001800
40
GTTTCCACA 624755 −
TTATCCACA 624772 +
CTTTCAACA 624920 −
>thiL>
624400
624800
625200

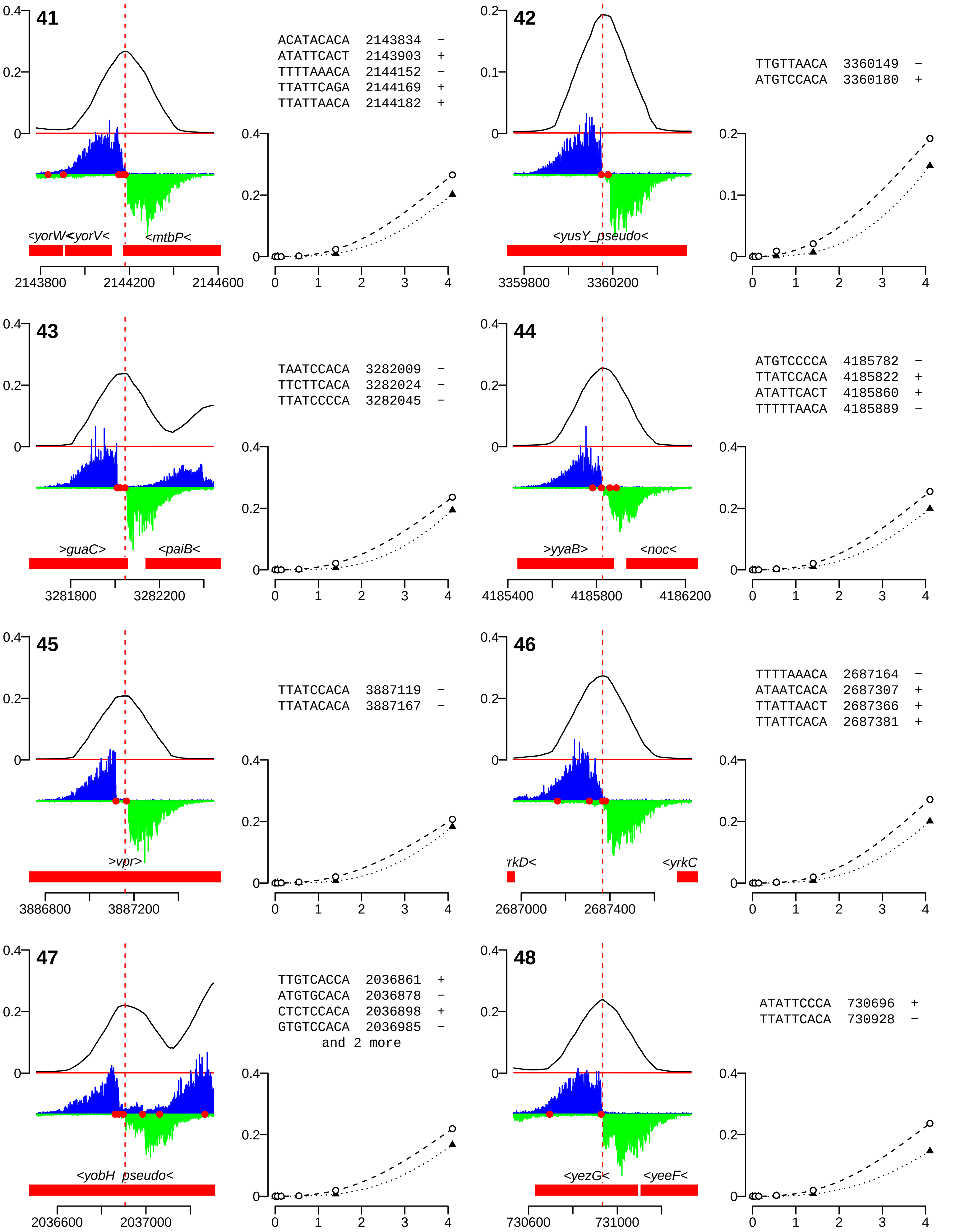
41
ACATACACA 2143834 −
ATATTCACT 2143903 +
TTTTAAACA 2144152 −
TTATTCAGA 2144169 +
TTATTAACA 2144182 +
<yorW<yorV<
<mtbP<
2143800
2144200
2144600
42
TTGTTAACA 3360149 −
ATGTCCACA 3360180 +
<yusY_pseudo<
3359800
3360200
43
TAATCCACA 3282009 −
TTCTTCACA 3282024 −
TTATCCCCA 3282045 −
>guaC>
<paiB<
3281800
3282200
44
ATGTCCCCA 4185782 −
TTATCCACA 4185822 +
ATATTCACT 4185860 +
TTTTTAACA 4185889 −
>yyaB>
<noc<
4185400
4185800
4186200
45
TTATCCACA 3887119 −
TTATACACA 3887167 −
>vpr>
3886800
3887200
46
TTTTAAACA 2687164 −
ATAATCACA 2687307 +
TTATTAACT 2687366 +
TTATTCACA 2687381 +
rkD<
<yrkC
2687000
2687400
47
TTGTCACCA 2036861 +
ATGTGCACA 2036878 −
CTCTCCACA 2036898 +
GTGTCCACA 2036985 −
and 2 more
<yobH_pseudo<
2036600
2037000
48
ATATTCCCA 730696 +
TTATTCACA 730928 −
<yezG<
<yeeF<
730600
731000

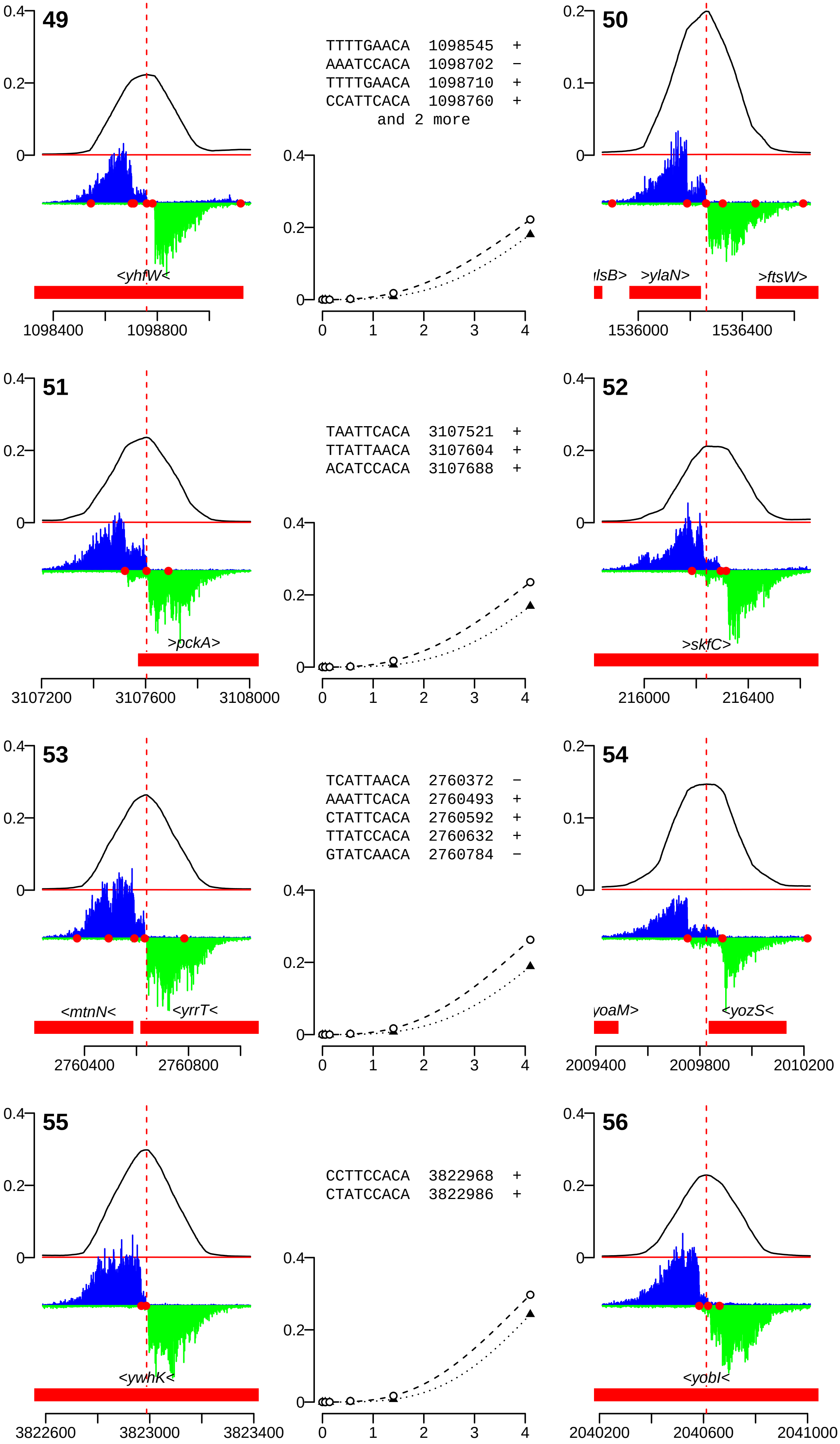
49
TTTTGAACA 1098545 +
AAATCCACA 1098702 −
TTTTGAACA 1098710 +
CCATTCACA 1098760 +
and 2 more
<yhfW<
1098400
1098800
50
GTATCAACA 1535900 −
TTATACACA 1536188 +
TTATTAACA 1536261 +
TTTTACACA 1536325 +
and 2 more
ilsB>
>ylaN>
>ftsW>
1536000
1536400
51
TAATTCACA 3107521 +
TTATTAACA 3107604 +
ACATCCACA 3107688 +
>pckA>
3107200
3107600
3108000
52
TAATCAACA 216184 −
TTGTCTACA 216294 +
TTATTAACA 216315 +
>skfC>
216000
216400
53
TCATTAACA 2760372 −
AAATTCACA 2760493 +
CTATTCACA 2760592 +
TTATCCACA 2760632 +
GTATCAACA 2760784 −
<mtnN<
<yrrT<
2760400
2760800
54
CAATCCACA 2009753 −
TCATCAACA 2009887 −
ATTTCCACA 2010214 +
yoaM>
<yozS<
2009400
2009800
2010200
55
CCTTCCACA 3822968 +
CTATCCACA 3822986 +
<ywhK<
3822600
3823000
3823400
56
AAATCCACA 2040584 +
TTGCTAACA 2040618 +
ATACCAACA 2040662 −
<yobI<
2040200
2040600
2041000

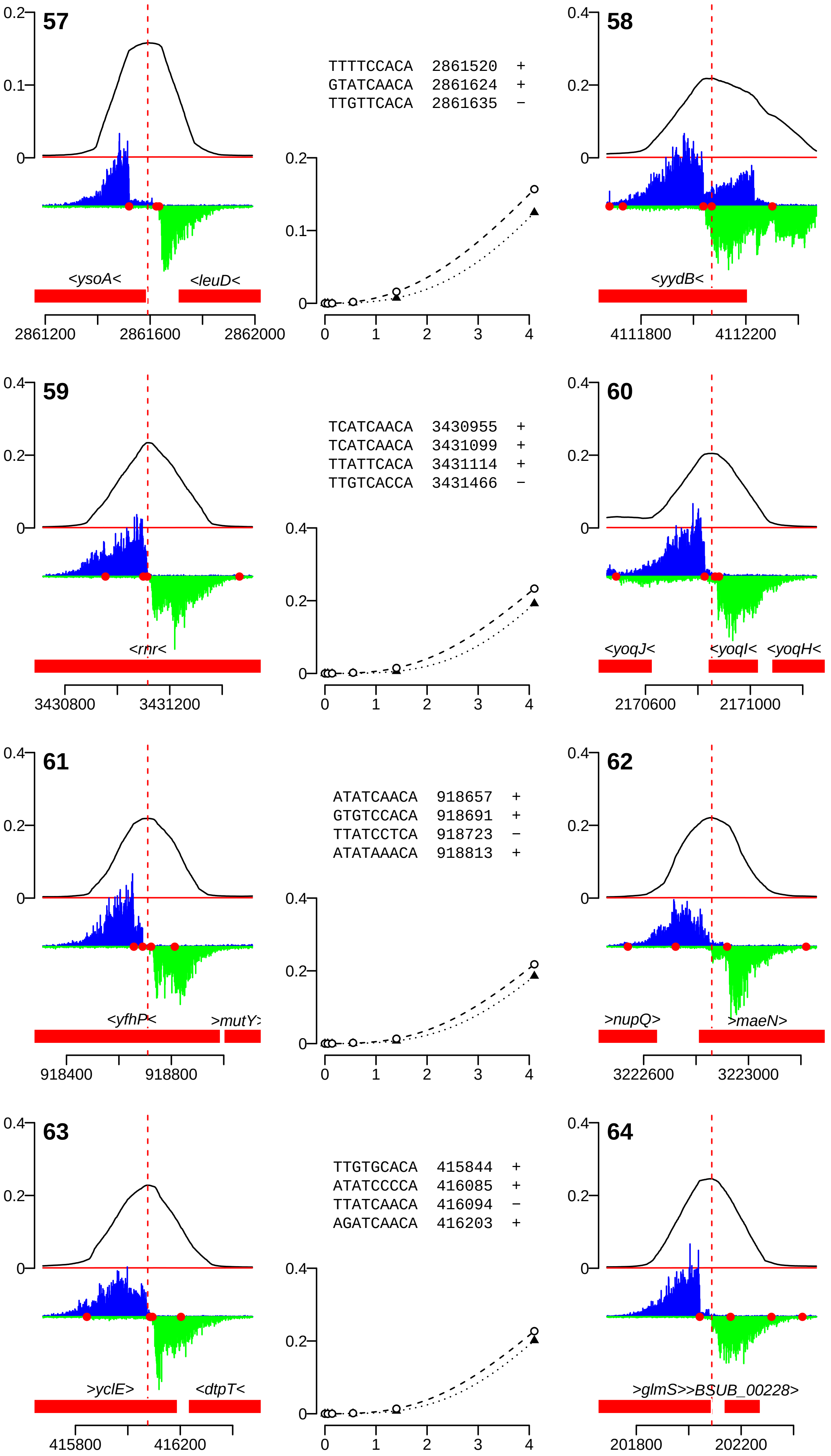
57
TTTTCCACA 2861520 +
GTATCAACA 2861624 +
TTGTTCACA 2861635 −
<ysoA<
<leuD<
2861200 2861600 2862000
58
TTATTAACT 4111680 −
TTATCAATA 4111731 −
TTATACACA 4112038 +
TTATTAACT 4112070 −
CTTTCCACA 4112301 +
<yydB<
4111800 4112200
59
TCATCAACA 3430955 +
TCATCAACA 3431099 +
TTATTCACA 3431114 +
TTGTCACCA 3431466 −
<rnr<
3430800 3431200
60
ATATGCACA 2170488 +
TTATTAACA 2170826 +
TTATCTACA 2170866 +
TCATTCCCA 2170881 +
<yoqJ<
<yoqI<
<yoqH<
2170600 2171000
61
ATATCAACA 918657 +
GTGTCCACA 918691 +
TTATCCTCA 918723 −
ATATAAACA 918813 +
<yfhP<
>mutY>
918400 918800
62
ATATACACA 3222540 −
TAATAAACA 3222722 −
TTATACACA 3222919 +
GTATGAACA 3223220 +
>nupQ>
>maeN>
3222600 3223000
63
TTGTGCACA 415844 +
ATATCCCCA 416085 +
TTATCAACA 416094 −
AGATCAACA 416203 +
>yclE>
<dtpT<
415800 416200
64
TTATCCACA 202042 −
TTTTCCACT 202160 −
GTATTAACA 202316 −
TTATTGACA 202434 +
>glmS>>BSUB_00228>
201800 202200

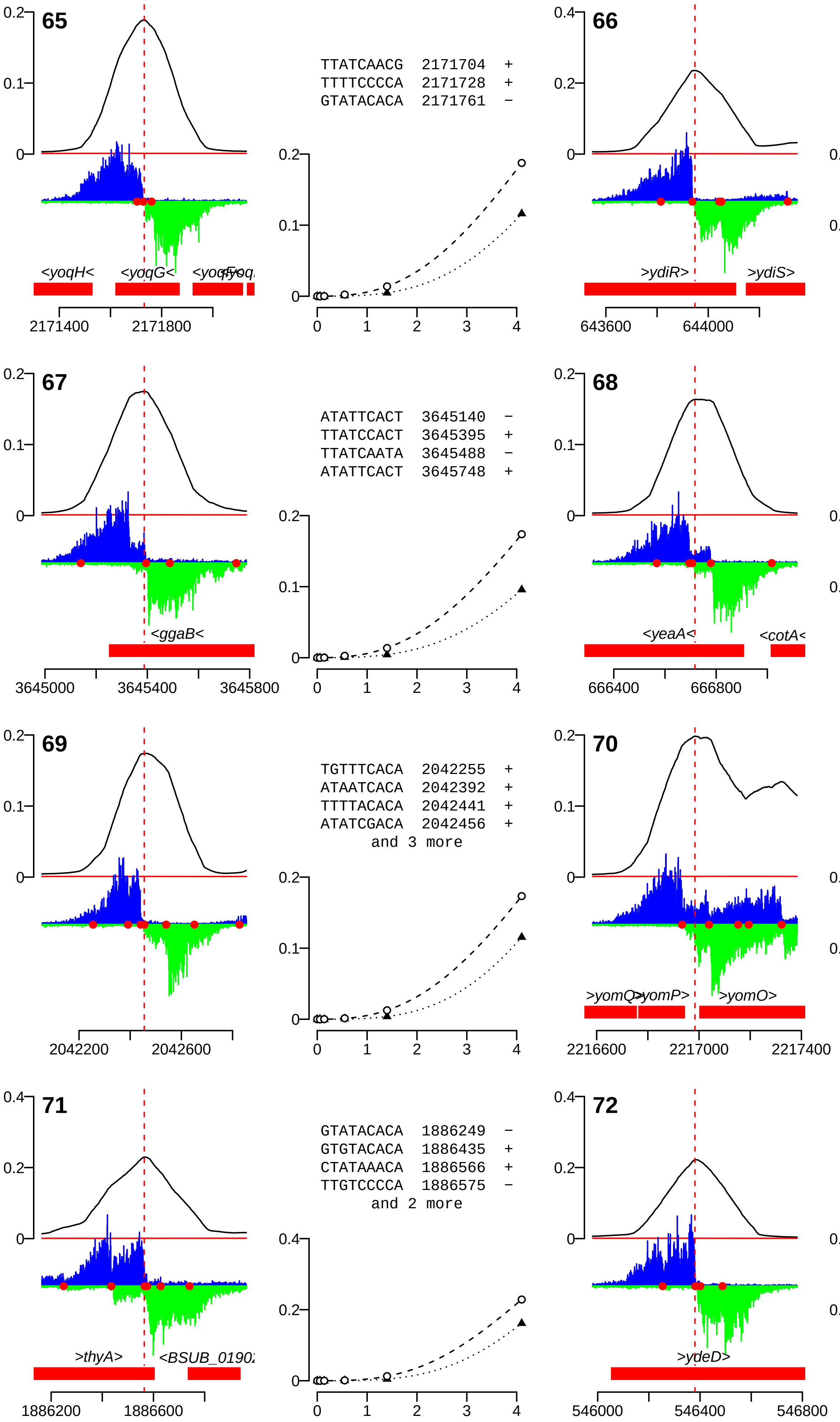

65
TTATCAACG 2171704 +
TTTTCCCCA 2171728 +
GTATACACA 2171761 −
<yoqH<
<yoqG<
2171400
2171800
66
ATATCACCA 643815 −
TTATCAACA 643938 −
ATGTCCCCA 644043 −
TCATCAACA 644052 −
TTATCACCA 644311 −
>ydiR>
>ydiS>
643600
644000
67
ATATTCACT 3645140 −
TTATCCACT 3645395 +
TTATCAATA 3645488 −
ATATTCACT 3645748 +
<ggaB<
3645000
3645400
3645800
68
ATGTTCACA 666568 −
CAATTCACA 666695 −
TTCTCAACA 666706 −
TTATTCACA 666779 +
TGATCCCCA 667017 −
<yeaA<
<cotA<
666400
666800
69
TGTTTCACA 2042255 +
ATAATCACA 2042392 +
TTTTACACA 2042441 +
ATATCGACA 2042456 +
and 3 more
2042200
2042600
70
TTATCTACA 2216934 +
TTATTGACA 2217039 −
TGATCAACA 2217153 +
AAATCAACA 2217194 +
TTATTAACA 2217323 +
>yomQ>
>yomP>
>yomO>
2216600
2217000
2217400
71
GTATACACA 1886249 −
GTGTACACA 1886435 +
CTATAAACA 1886566 +
TTGTCCCCA 1886575 −
and 2 more
>thyA>
<BSUB_0190
1886200
1886600
72
GTTTCCACA 546254 −
TTATCCCCA 546382 +
TTATCTACA 546401 +
TTGTTAACA 546488 −
>ydeD>
546000
546400
546800

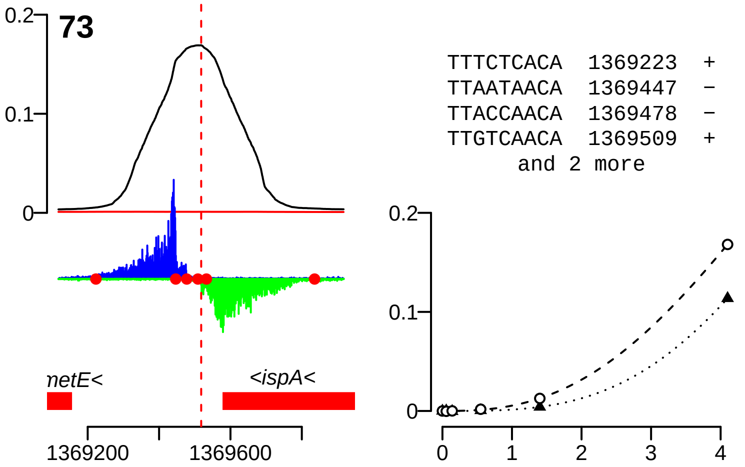
73
TTTCTCACA 1369223 +
TTAATAACA 1369447 −
TTACCAACA 1369478 −
TTGTCAACA 1369509 +
and 2 more
netE<
<ispA<
1369200
1369600

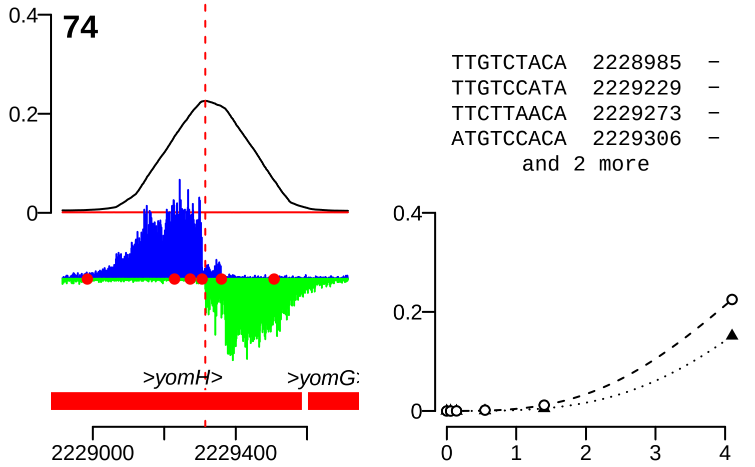
74
TTGTCTACA 2228985 −
TTGTCCATA 2229229 −
TTCTTAACA 2229273 −
ATGTCCACA 2229306 −
and 2 more
>yomH>
>yomG
2229000
2229400

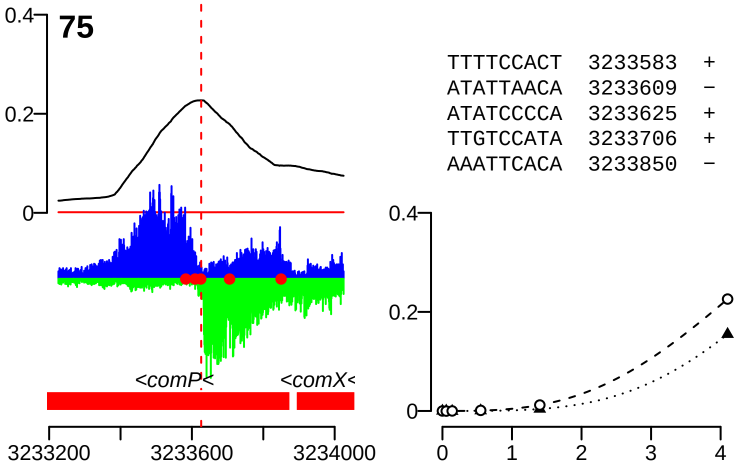
75
TTTTCCACT 3233583 +
ATATTAACA 3233609 −
ATATCCCCA 3233625 +
TTGTCCATA 3233706 +
AAATTCACA 3233850 −
<comP<
<comX<
3233200
3233600
3234000

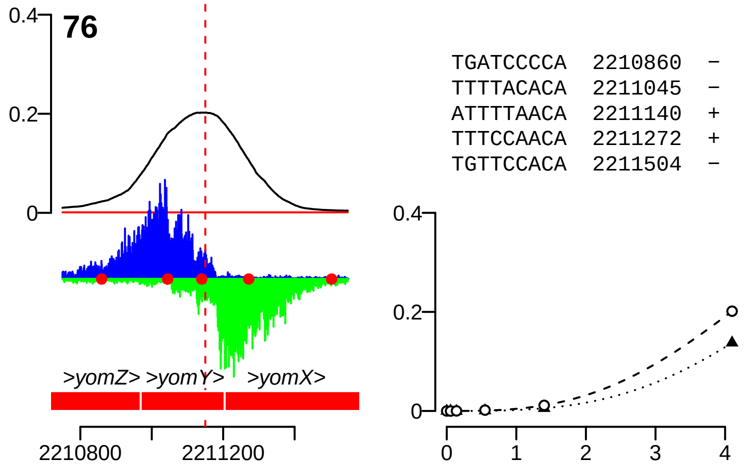
76
TGATCCCCA 2210860 −
TTTTACACA 2211045 −
ATTTTAACA 2211140 +
TTTCCAACA 2211272 +
TGTTCCACA 2211504 −
>yomZ>
>yomY>
>yomX>
2210800
2211200

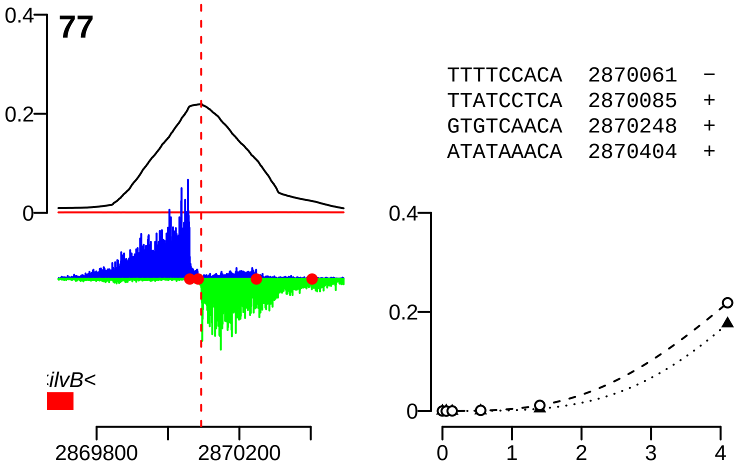
77
TTTTCCACA 2870061 −
TTATCCTCA 2870085 +
GTGTCAACA 2870248 +
ATATAAACA 2870404 +
ilvB<
2869800
2870200

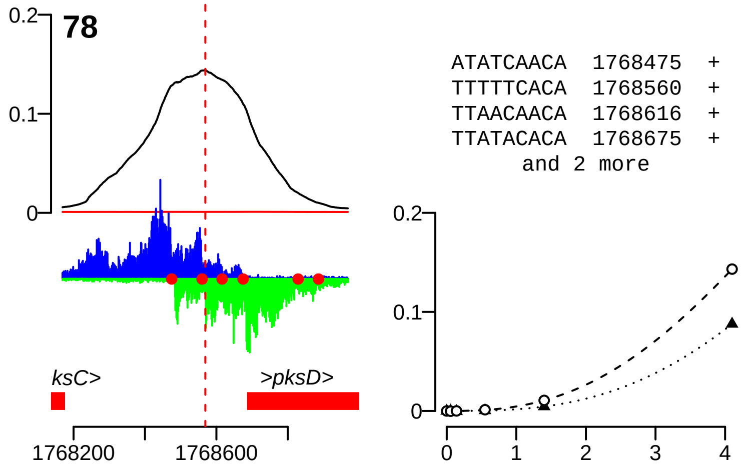
78
ATATCAACA 1768475 +
TTTTTCACA 1768560 +
TTAACAACA 1768616 +
TTATACACA 1768675 +
and 2 more
ksC>
>pksD>
1768200
1768600

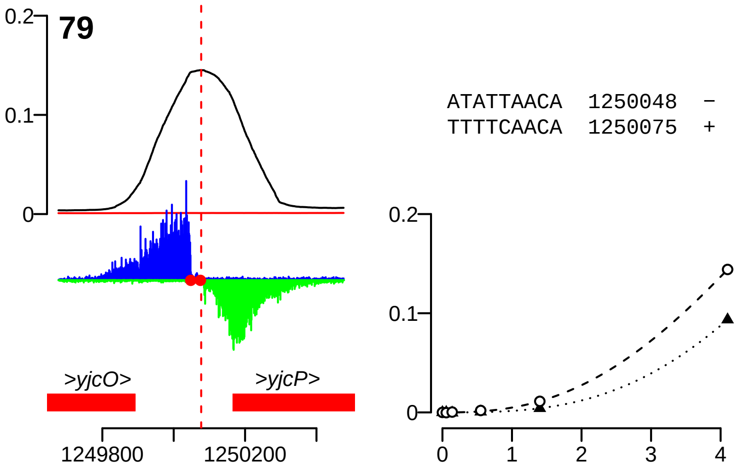
79
ATATTAACA 1250048 −
TTTTCAACA 1250075 +
>yjcO>
>yjcP>
1249800
1250200

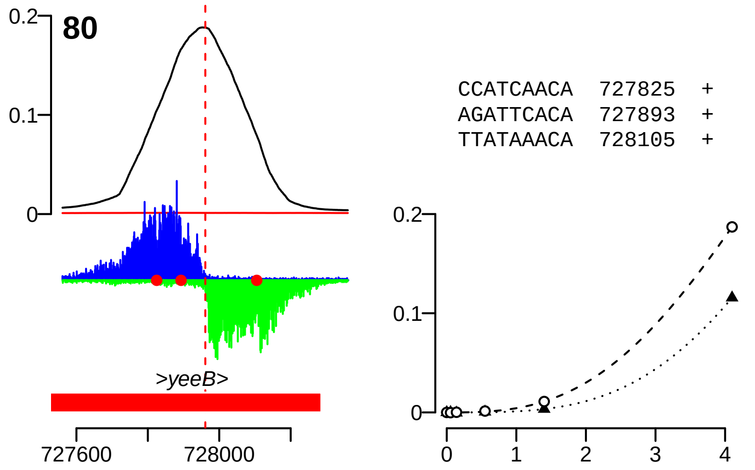
80
CCATCAACA 727825 +
AGATTCACA 727893 +
TTATAAACA 728105 +
>yeeB>
727600
728000

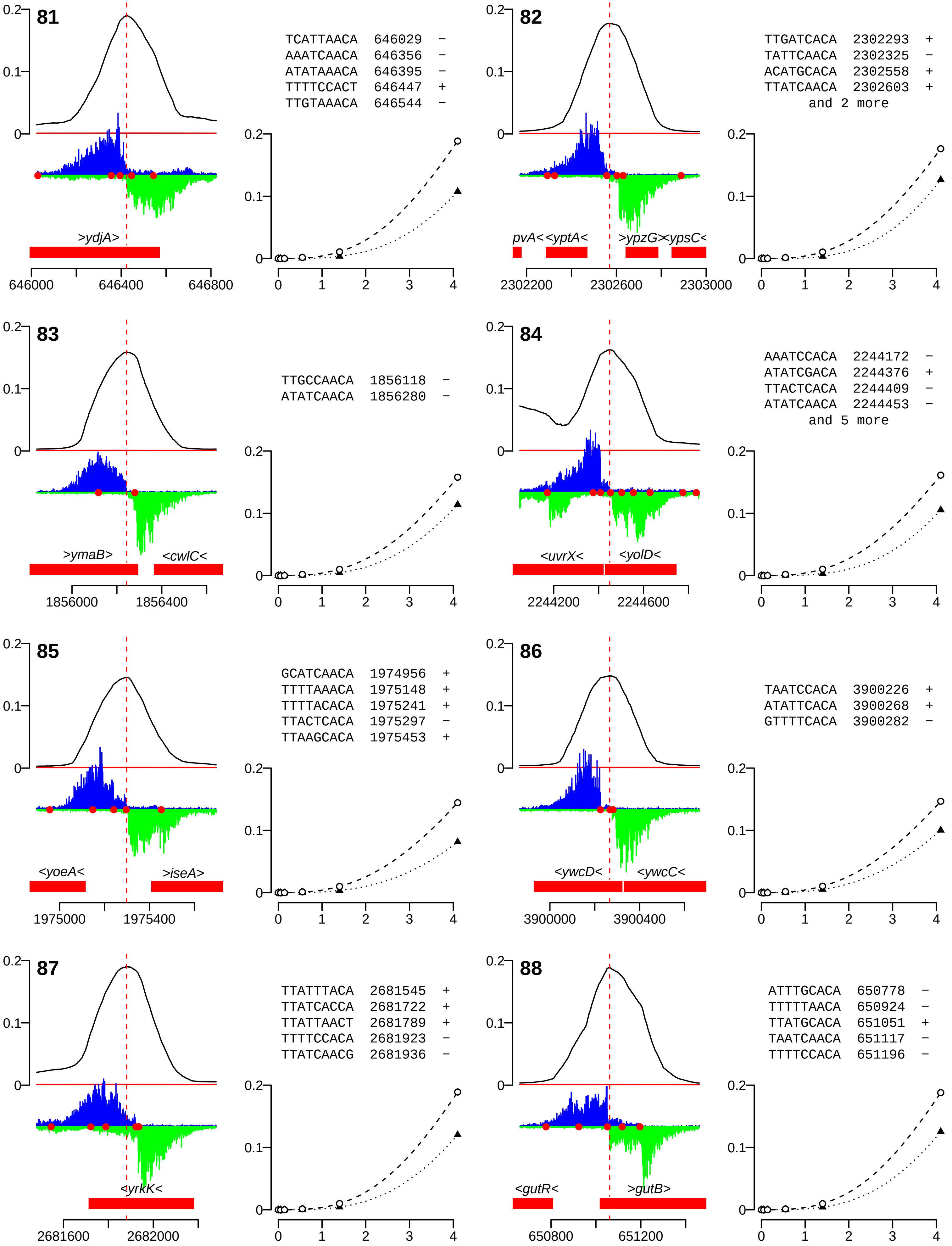
81
TCATTAACA 646029 −
AAATCAACA 646356 −
ATATAAACA 646395 −
TTTTCCACT 646447 +
TTGTAAACA 646544 −
>ydjA>
646000
646400
646800
82
TTGATCACA 2302293 +
TATTCAACA 2302325 −
ACATGCACA 2302558 +
TTATCAACA 2302603 +
and 2 more
pvA<
<yptA<
>ypzG>
<ypsC<
2302200
2302600
2303000
83
TTGCCAACA 1856118 −
ATATCAACA 1856280 −
>ymaB>
<cwlC<
1856000
1856400
84
AAATCCACA 2244172 −
ATATCGACA 2244376 +
TTACTCACA 2244409 −
ATATCAACA 2244453 −
and 5 more
<uvrX<
<yolD<
2244200
2244600
85
GCATCAACA 1974956 +
TTTTAAACA 1975148 +
TTTTACACA 1975241 +
TTACTCACA 1975297 −
TTAAGCACA 1975453 +
<yoeA<
>iseA>
1975000
1975400
86
TAATCCACA 3900226 +
ATATTCACA 3900268 +
GTTTTCACA 3900282 −
<ywcD<
<ywcC<
3900000
3900400
87
TTATTTACA 2681545 +
TTATCACCA 2681722 +
TTATTAACT 2681789 +
TTTTCCACA 2681923 −
TTATCAACG 2681936 −
<yrkK<
2681600
2682000
88
ATTTGCACA 650778 −
TTTTTAACA 650924 −
TTATGCACA 651051 +
TAATCAACA 651117 −
TTTTCCACA 651196 −
<gutR<
>gutB>
650800
651200
0.2
0.1
0
0 1 2 3 4

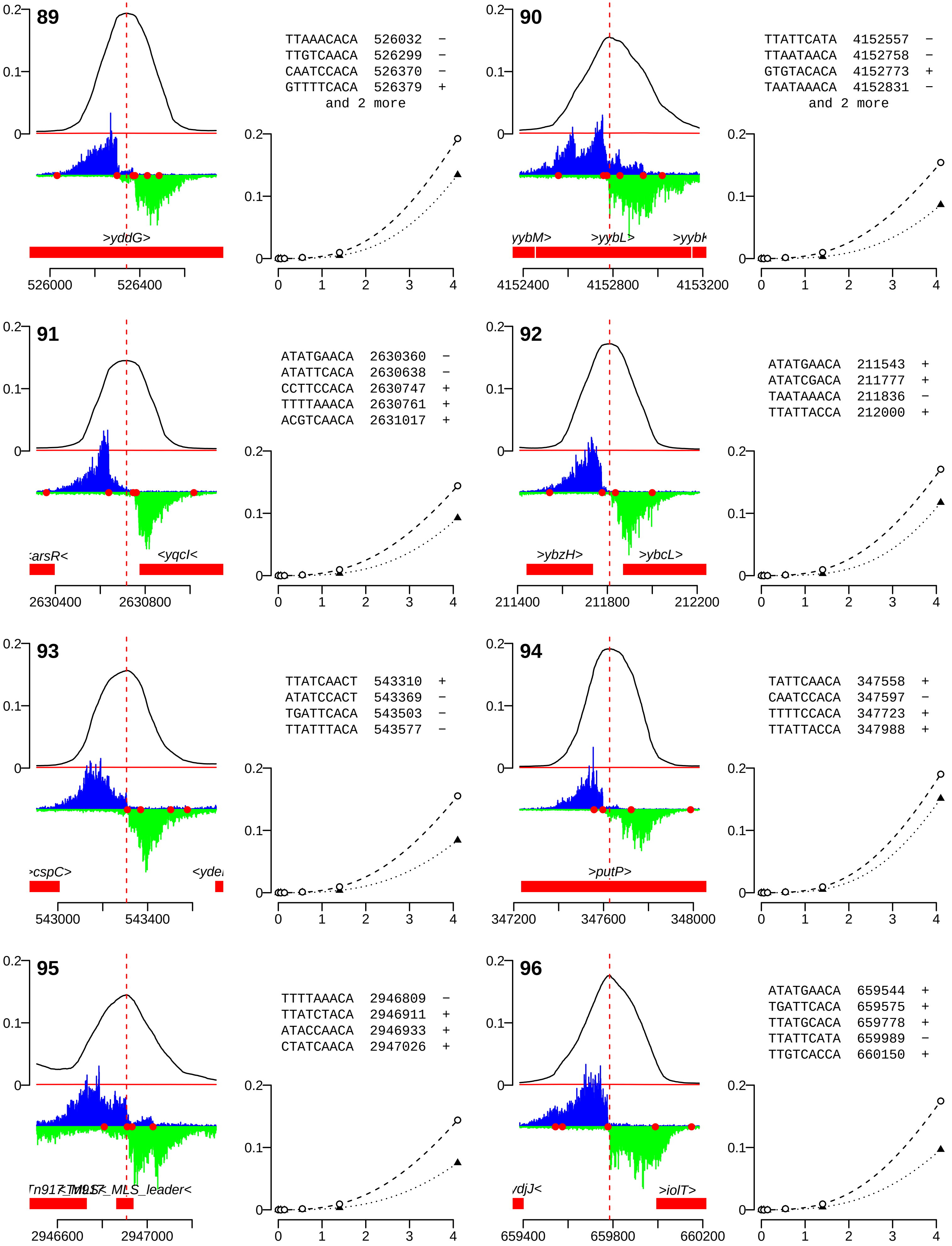

89
TTAAACACA 526032 −
TTGTCAACA 526299 −
CAATCCACA 526370 −
GTTTTCACA 526379 +
and 2 more
>yddG>
526000 526400
90
TTATTCATA 4152557 −
TTAATAACA 4152758 −
GTGTACACA 4152773 +
TAATAAACA 4152831 −
and 2 more
yybM> >yybL>
4152400 4152800 4153200
91
ATATGAACA 2630360 −
ATATTCACA 2630638 −
CCTTCCACA 2630747 +
TTTTAAACA 2630761 +
ACGTCAACA 2631017 +
<yqcI<
2630400 2630800
92
ATATGAACA 211543 +
ATATCGACA 211777 +
TAATAAACA 211836 −
TTATTACCA 212000 +
>ybzH> >ybcL>
211400 211800 212200
93
TTATCAACT 543310 +
ATATCCACT 543369 −
TGATTCACA 543503 −
TTATTTACA 543577 −
543000 543400
94
TATTCAACA 347558 +
CAATCCACA 347597 −
TTTTCCACA 347723 +
TTATTACCA 347988 +
>putP>
347200 347600 348000
95
TTTTAAACA 2946809 −
TTATCTACA 2946911 +
ATACCAACA 2946933 +
CTATCAACA 2947026 +
2946600 2947000
96
ATATGAACA 659544 +
TGATTCACA 659575 +
TTATGCACA 659778 +
TTATTCATA 659989 −
TTGTCACCA 660150 +
>iolT>
659400 659800 660200
0.2
0.1
0
0 1 2 3 4

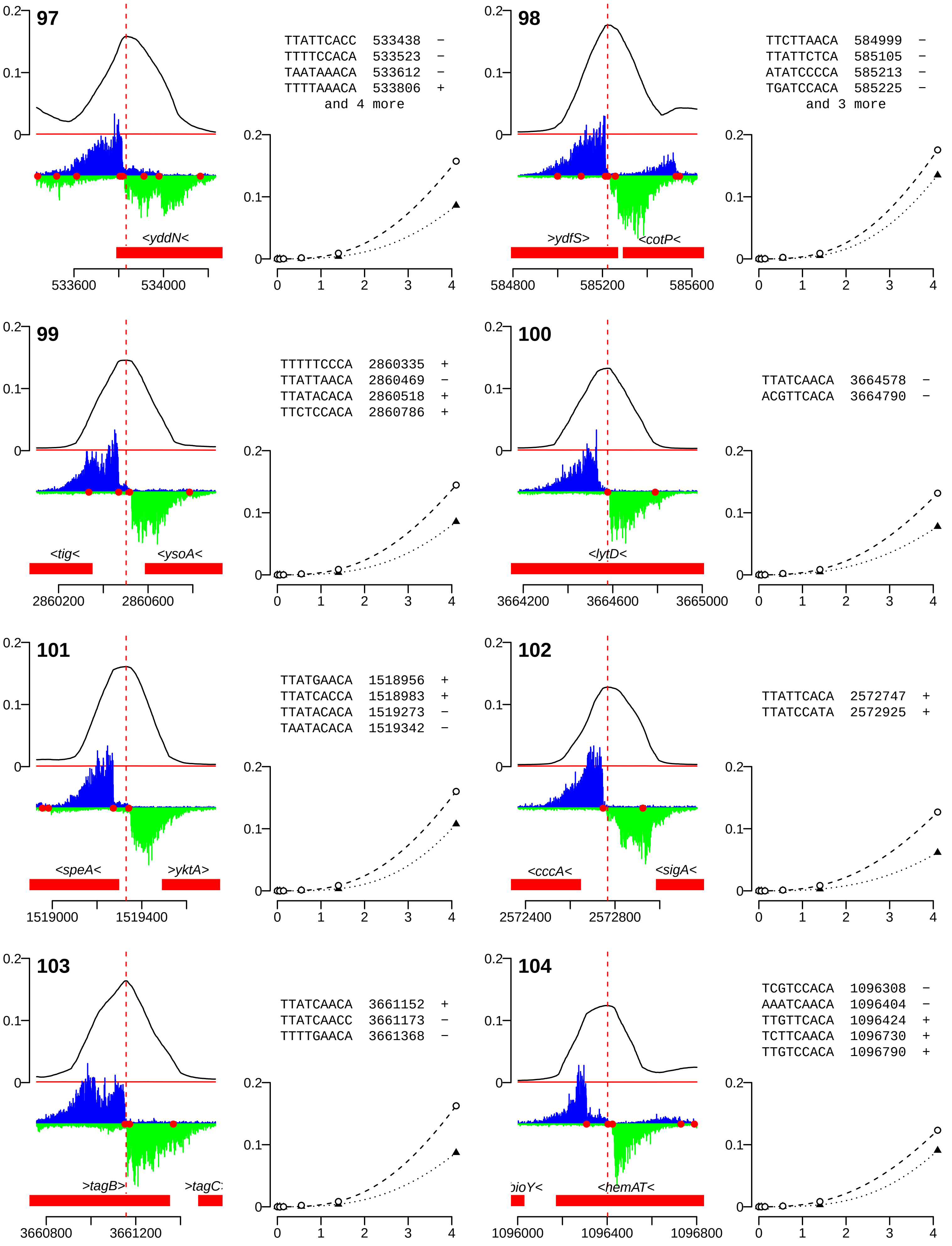
97
TTATTCACC 533438 −
TTTTCCACA 533523 −
TAATAAACA 533612 −
TTTTAAACA 533806 +
and 4 more
<yddN<
533600 534000
98
TTCTTAACA 584999 −
TTATTCTCA 585105 −
ATATCCCCA 585213 −
TGATCCACA 585225 −
and 3 more
>ydfS>
<cotP<
584800 585200 585600
99
TTTTTCCCA 2860335 +
TTATTAACA 2860469 −
TTATACACA 2860518 +
TTCTCCACA 2860786 +
<tig<
<ysoA<
2860200 2860600
100
TTATCAACA 3664578 −
ACGTTCACA 3664790 −
<lytD<
3664200 3664600 3665000
101
TTATGAACA 1518956 +
TTATCACCA 1518983 +
TTATACACA 1519273 −
TAATACACA 1519342 −
<speA<
>yktA>
1519000 1519400
102
TTATTCACA 2572747 +
TTATCCATA 2572925 +
<cccA<
<sigA<
2572400 2572800
103
TTATCAACA 3661152 +
TTATCAACC 3661173 −
TTTTGAACA 3661368 −
>tagB>
>tagC
3660800 3661200
104
TCGTCCACA 1096308 −
AAATCAACA 1096404 −
TTGTTCACA 1096424 +
TCTTCAACA 1096730 +
TTGTCCACA 1096790 +
<hemAT<
1096000 1096400 1096800
0.2 0.1 0
0 1 2 3 4

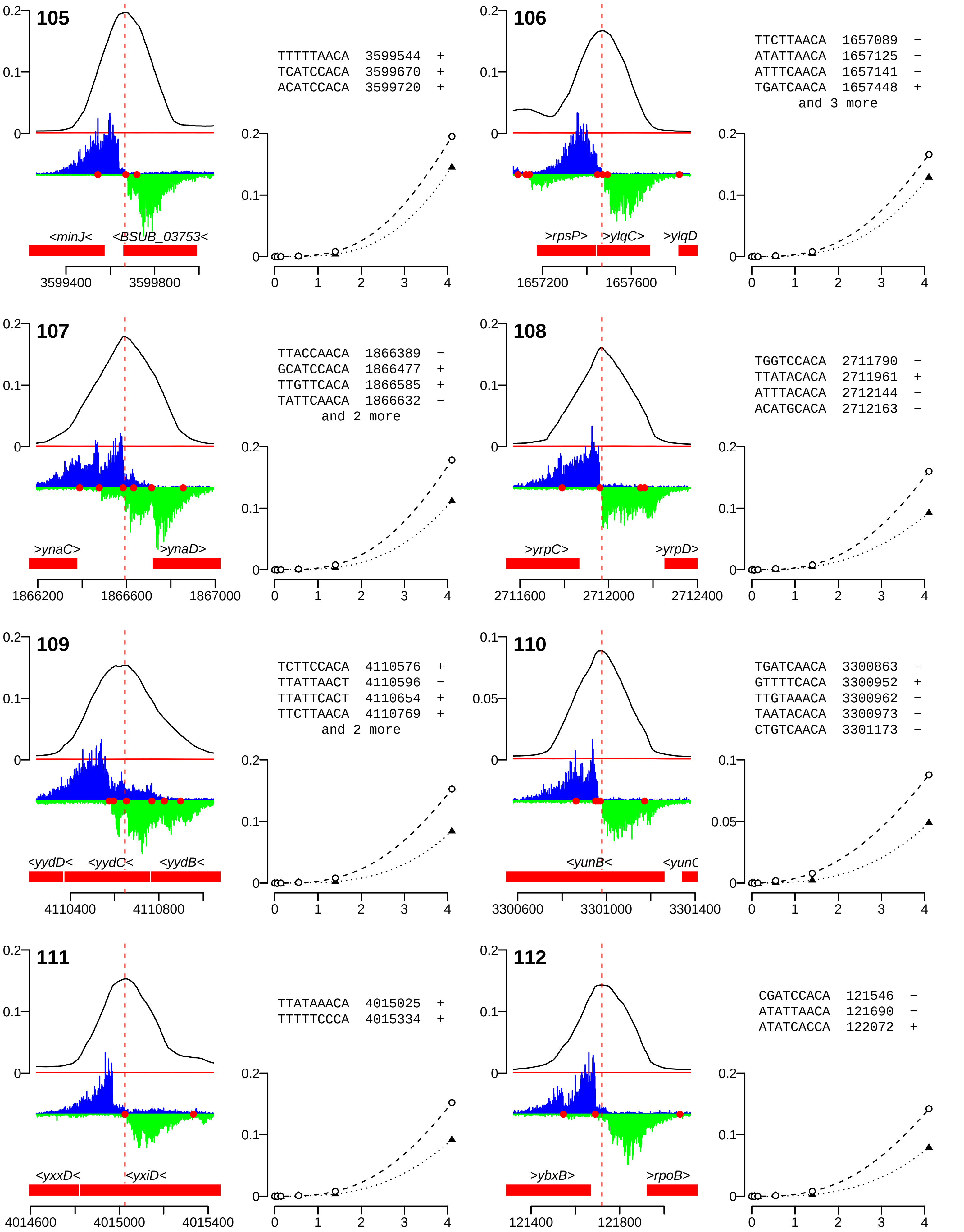
105
TTTTTAACA 3599544 +
TCATCCACA 3599670 +
ACATCCACA 3599720 +
<minJ<
<BSUB_03753<
3599400
3599800
106
TTCTTAACA 1657089 −
ATATTAACA 1657125 −
ATTTCAACA 1657141 −
TGATCAACA 1657448 +
and 3 more
>rpsP>
>ylqC>
>ylqD
1657200
1657600
107
TTACCAACA 1866389 −
GCATCCACA 1866477 +
TTGTTCACA 1866585 +
TATTCAACA 1866632 −
and 2 more
>ynaC>
>ynaD>
1866200
1866600
1867000
108
TGGTCCACA 2711790 −
TTATACACA 2711961 +
ATTTACACA 2712144 −
ACATGCACA 2712163 −
>yrpC>
>yrpD>
2711600
2712000
2712400
109
TCTTCCACA 4110576 +
TTATTAACT 4110596 −
TTATTCACT 4110654 +
TTCTTAACA 4110769 +
and 2 more
<yydD<
<yydC<
<yydB<
4110400
4110800
110
TGATCAACA 3300863 −
GTTTTCACA 3300952 +
TTGTAAACA 3300962 −
TAATACACA 3300973 −
CTGTCAACA 3301173 −
<yunB<
<yun
3300600
3301000
3301400
111
TTATAAACA 4015025 +
TTTTTCCCA 4015334 +
<yxxD<
<yxiD<
4014600
4015000
4015400
112
CGATCCACA 121546 −
ATATTAACA 121690 −
ATATCACCA 122072 +
>ybxB>
>rpoB>
121400
121800

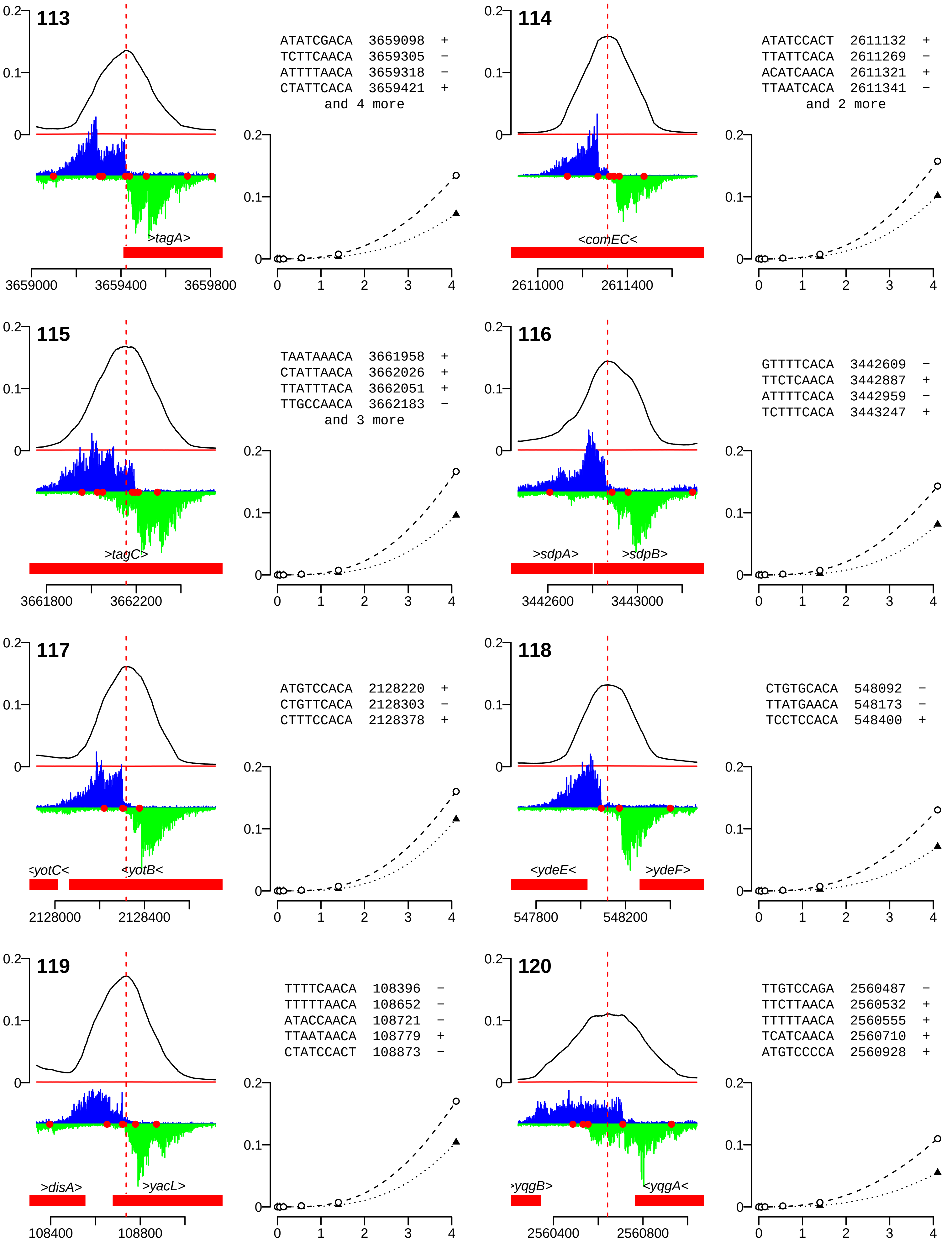
113
ATATCGACA 3659098 +
TCTTCAACA 3659305 −
ATTTTAACA 3659318 −
CTATTCACA 3659421 +
and 4 more
>tagA>
3659000 3659400 3659800
114
ATATCCACT 2611132 +
TTATTCACA 2611269 −
ACATCAACA 2611321 +
TTAATCACA 2611341 −
and 2 more
<comEC<
2611000 2611400
115
TAATAAACA 3661958 +
CTATTAACA 3662026 +
TTATTTACA 3662051 +
TTGCCAACA 3662183 −
and 3 more
>tagC>
3661800 3662200
116
GTTTTCACA 3442609 −
TTCTCAACA 3442887 +
ATTTTCACA 3442959 −
TCTTTCACA 3443247 +
>sdpA> >sdpB>
3442600 3443000
117
ATGTCCACA 2128220 +
CTGTTCACA 2128303 −
CTTTCCACA 2128378 +
<yotC< <yotB<
2128000 2128400
118
CTGTGCACA 548092 −
TTATGAACA 548173 −
TCCTCCACA 548400 +
<ydeE< >ydeF>
547800 548200
119
TTTTCAACA 108396 −
TTTTTAACA 108652 −
ATACCAACA 108721 −
TTAATAACA 108779 +
CTATCCACT 108873 −
>disA> >yacL>
108400 108800
120
TTGTCCAGA 2560487 −
TTCTTAACA 2560532 +
TTTTTAACA 2560555 +
TCATCAACA 2560710 +
ATGTCCCCA 2560928 +
>yqgB> <yqgA<
2560400 2560800

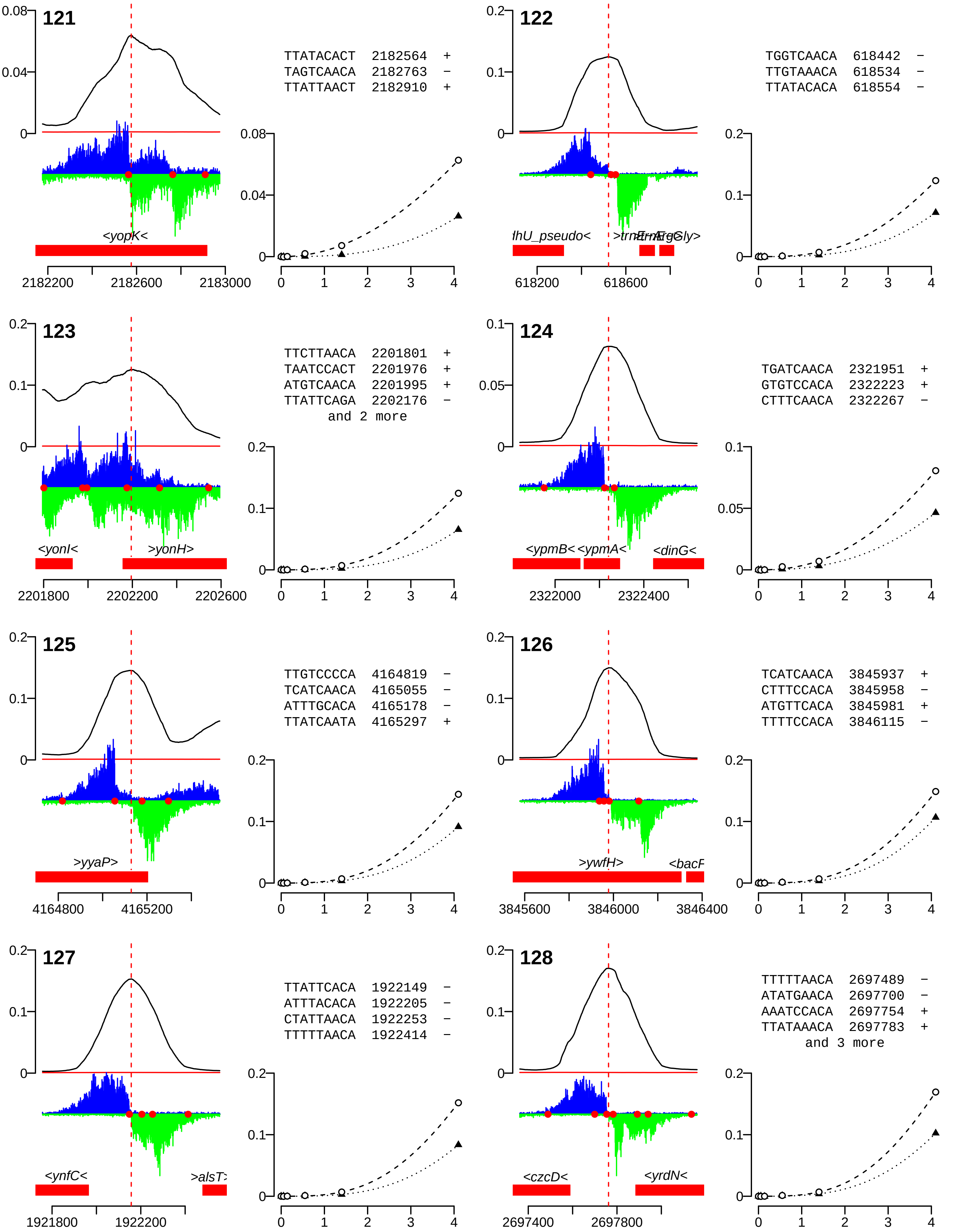
121
TTATACACT 2182564 +
TAGTCAACA 2182763 −
TTATTAACT 2182910 +
<yopK<
2182200 2182600 2183000
122
TGGTCAACA 618442 −
TTGTAAACA 618534 −
TTATACACA 618554 −
lhU_pseudo<
618200 618600
123
TTCTTAACA 2201801 +
TAATCCACT 2201976 +
ATGTCAACA 2201995 +
TTATTCAGA 2202176 −
and 2 more
<yonI<
>yonH>
2201800 2202200 2202600
124
TGATCAACA 2321951 +
GTGTCCACA 2322223 +
CTTTCAACA 2322267 −
<ypmB<
<ypmA<
<dinG<
2322000 2322400
125
TTGTCCCCA 4164819 −
TCATCAACA 4165055 −
ATTTGCACA 4165178 −
TTATCAATA 4165297 +
>yyaP>
4164800 4165200
126
TCATCAACA 3845937 +
CTTTCCACA 3845958 −
ATGTTCACA 3845981 +
TTTTCCACA 3846115 −
>ywfH>
<bacF
3845600 3846000 3846400
127
TTATTCACA 1922149 −
ATTTACACA 1922205 −
CTATTAACA 1922253 −
TTTTTAACA 1922414 −
<ynfC<
>alsT
1921800 1922200
128
TTTTTAACA 2697489 −
ATATGAACA 2697700 −
AAATCCACA 2697754 +
TTATAAACA 2697783 +
and 3 more
<czcD<
<yrdN<
2697400 2697800

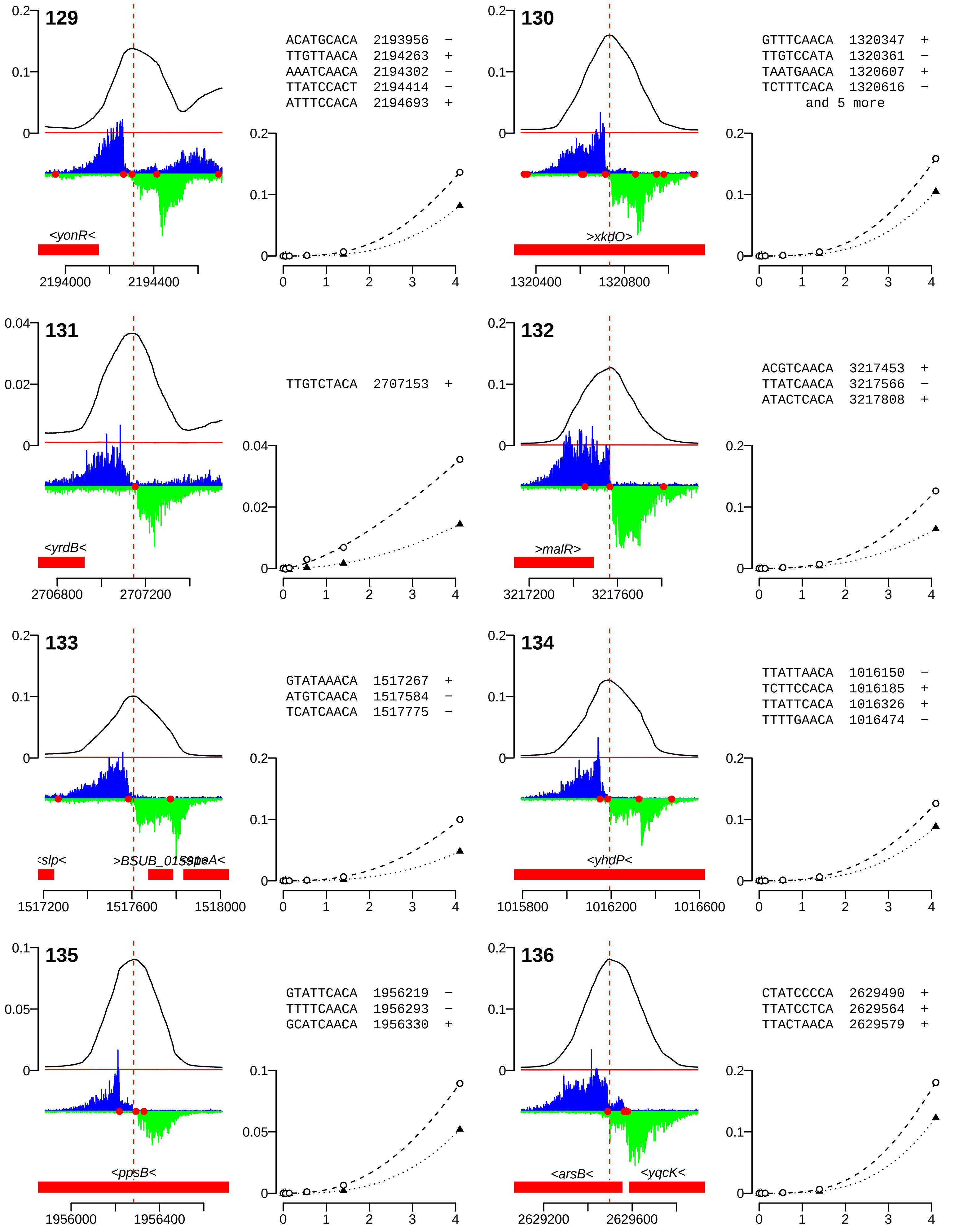
129
0.2
0.1
0
<yonR<
2194000
2194400
ACATGCACA 2193956 −
TTGTTAACA 2194263 +
AAATCAACA 2194302 −
TTATCCACT 2194414 −
ATTTCCACA 2194693 +
0.2
0.1
0
0 1 2 3 4
130
0.2
0.1
0
>xkdO>
1320400
1320800
GTTTCAACA 1320347 +
TTGTCCATA 1320361 −
TAATGAACA 1320607 +
TCTTTCACA 1320616 −
and 5 more
0.2
0.1
0
0 1 2 3 4
131
0.04
0.02
0
<yrdB<
2706800
2707200
TTGTCTACA 2707153 +
0.04
0.02
0
0 1 2 3 4
132
0.2
0.1
0
>malR>
3217200
3217600
ACGTCAACA 3217453 +
TTATCAACA 3217566 −
ATACTCACA 3217808 +
0.2
0.1
0
0 1 2 3 4
133
0.2
0.1
0
<slp<
>BSUB_01591<
<pheA<
1517200
1517600
1518000
GTATAAACA 1517267 +
ATGTCAACA 1517584 −
TCATCAACA 1517775 −
0.2
0.1
0
0 1 2 3 4
134
0.2
0.1
0
<yhdP<
1015800
1016200
1016600
TTATTAACA 1016150 −
TCTTCCACA 1016185 +
TTATTCACA 1016326 +
TTTTGAACA 1016474 −
0.2
0.1
0
0 1 2 3 4
135
0.1
0.05
0
<ppsB<
1956000
1956400
GTATTCACA 1956219 −
TTTTCAACA 1956293 −
GCATCAACA 1956330 +
0.1
0.05
0
0 1 2 3 4
136
0.2
0.1
0
<arsB<
<yqcK<
2629200
2629600
CTATCCCCA 2629490 +
TTATCCTCA 2629564 +
TTACTAACA 2629579 +
0.2
0.1
0
0 1 2 3 4

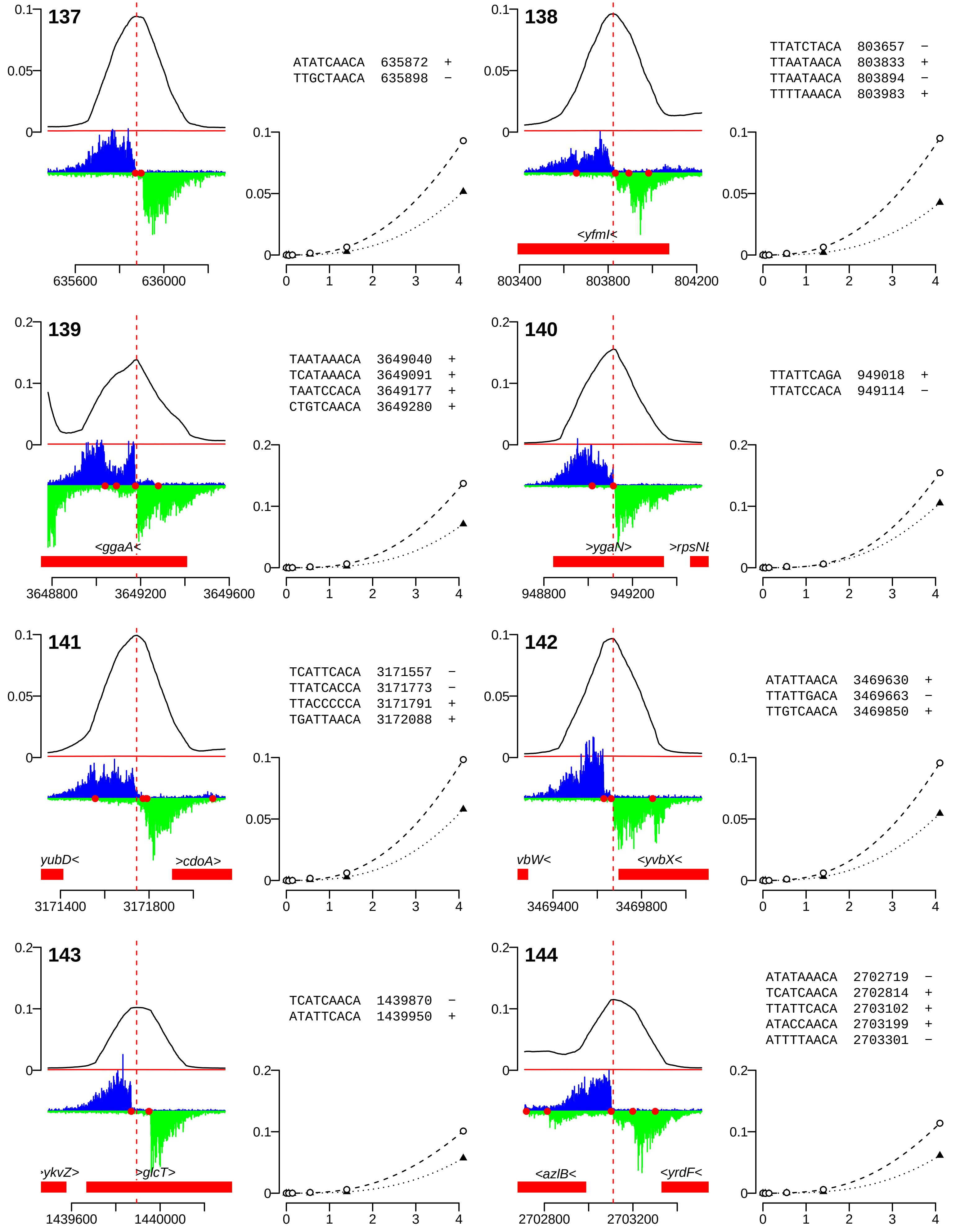
137
ATATCAACA 635872 +
TTGCTAACA 635898 −
635600
636000
138
TTATCTACA 803657 −
TTAATAACA 803833 +
TTAATAACA 803894 −
TTTTAACA 803983 +
<yfmI<
803400
803800
804200
139
TAATAAACA 3649040 +
TCATAAACA 3649091 +
TAATCCACA 3649177 +
CTGTCAACA 3649280 +
<ggaA<
3648800
3649200
3649600
140
TTATTCAGA 949018 +
TTATCCACA 949114 −
>ygaN>
948800
949200
141
TCATTCACA 3171557 −
TTATCACCA 3171773 −
TTACCCCCA 3171791 +
TGATTAACA 3172088 +
yubD<
>cdoA>
3171400
3171800
142
ATATTAACA 3469630 +
TTATTGACA 3469663 −
TTGTCAACA 3469850 +
vbW<
<yvbX<
3469400
3469800
143
TCATCAACA 1439870 −
ATATTCACA 1439950 +
ykvZ>
>glcT>
1439600
1440000
144
ATATAAACA 2702719 −
TCATCAACA 2702814 +
TTATTCACA 2703102 +
ATACCAACA 2703199 +
ATTTTAACA 2703301 −
<azlB<
<yrdF<
2702800
2703200

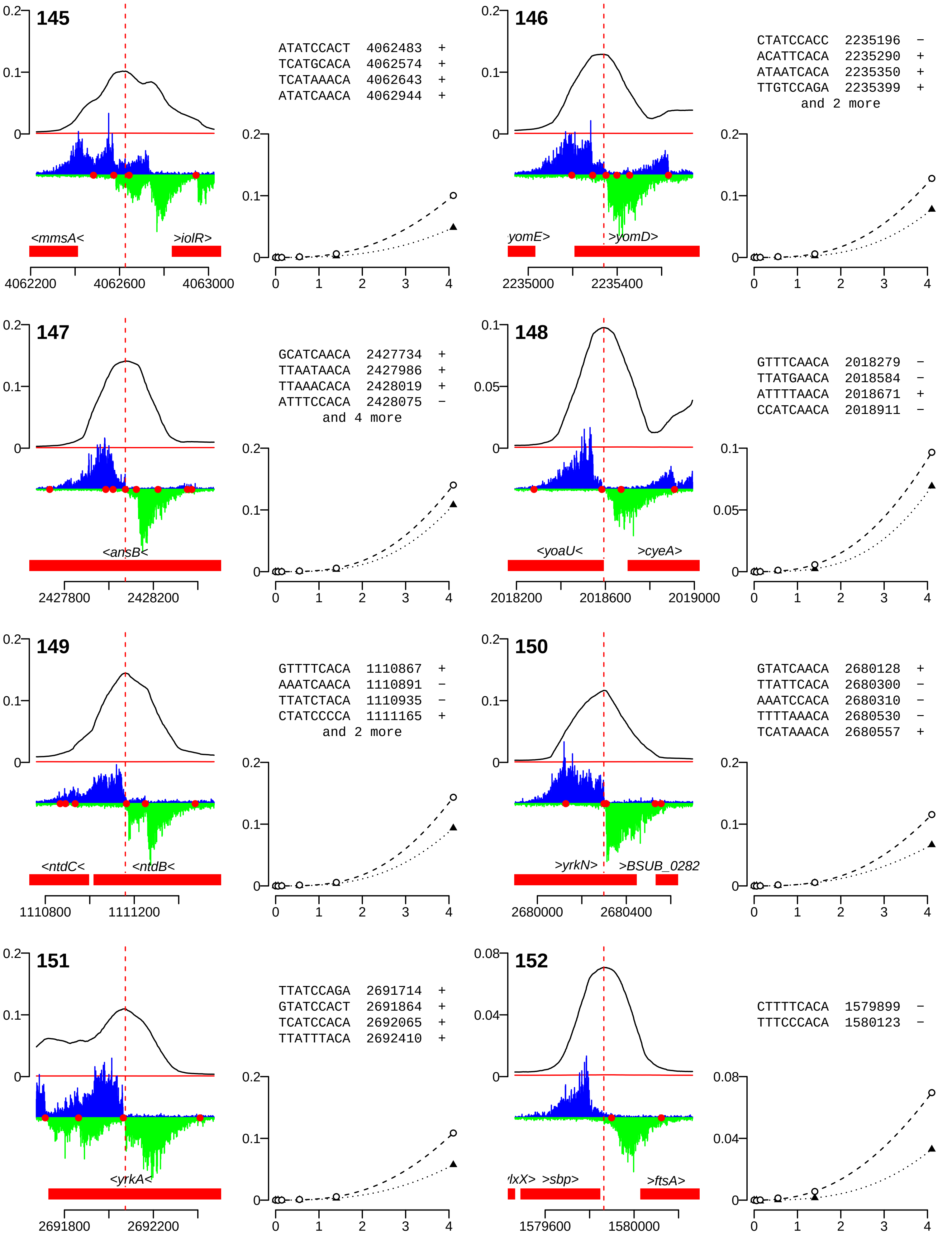
145
ATATCCACT 4062483 +
TCATGCACA 4062574 +
TCATAAACA 4062643 +
ATATCAACA 4062944 +
<mmsA<
>iolR>
4062200
4062600
4063000
146
CTATCCACC 2235196 −
ACATTCACA 2235290 +
ATAATCACA 2235350 +
TTGTCCAGA 2235399 +
and 2 more
yomE>
>yomD>
2235000
2235400
147
GCATCAACA 2427734 +
TTAATAACA 2427986 +
TTAAACACA 2428019 +
ATTTCCACA 2428075 −
and 4 more
<ansB<
2427800
2428200
148
GTTTCAACA 2018279 −
TTATGAACA 2018584 −
ATTTTAACA 2018671 +
CCATCAACA 2018911 −
<yoaU<
>cyeA>
2018200
2018600
2019000
149
GTTTTCACA 1110867 +
AAATCAACA 1110891 −
TTATCTACA 1110935 −
CTATCCCCA 1111165 +
and 2 more
<ntdC<
<ntdB<
1110800
1111200
150
GTATCAACA 2680128 +
TTATTCACA 2680300 −
AAATCCACA 2680310 −
TTTTAAACA 2680530 −
TCATAAACA 2680557 +
>yrkN>
>BSUB_0282
2680000
2680400
151
TTATCCAGA 2691714 +
GTATCCACT 2691864 +
TCATCCACA 2692065 +
TTATTTACA 2692410 +
<yrkA<
2691800
2692200
152
CTTTTCACA 1579899 −
TTTCCCACA 1580123 −
>sbp>
>ftsA>
1579600
1580000

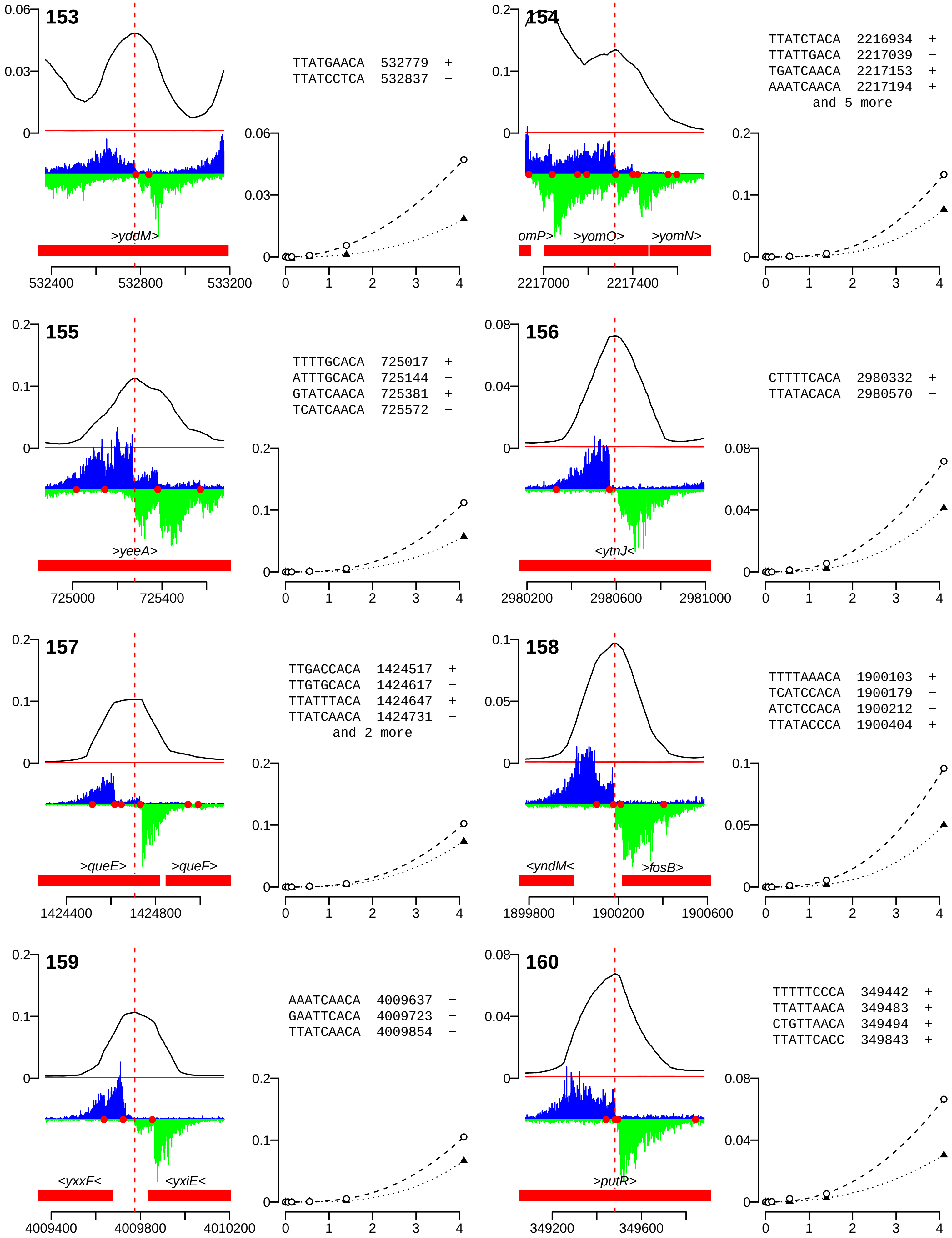

153
TTATGAACA 532779 +
TTATCCTCA 532837 −
>yddM>
532400
532800
533200
154
TTATCTACA 2216934 +
TTATTGACA 2217039 −
TGATCAACA 2217153 +
AAATCAACA 2217194 +
and 5 more
omP>
>yomO>
>yomN>
2217000
2217400
155
TTTTGCACA 725017 +
ATTTGCACA 725144 −
GTATCAACA 725381 +
TCATCAACA 725572 −
>yeeA>
725000
725400
156
CTTTTCACA 2980332 +
TTATACACA 2980570 −
<ytnJ<
2980200
2980600
2981000
157
TTGACCACA 1424517 +
TTGTGCACA 1424617 −
TTATTTACA 1424647 +
TTATCAACA 1424731 −
and 2 more
>queE>
>queF>
1424400
1424800
158
TTTTAAACA 1900103 +
TCATCCACA 1900179 −
ATCTCCACA 1900212 −
TTATACCCA 1900404 +
<yndM<
>fosB>
1899800
1900200
1900600
159
AAATCAACA 4009637 −
GAATTCACA 4009723 −
TTATCAACA 4009854 −
<yxxF<
<yxiE<
4009400
4009800
4010200
160
TTTTTCCCA 349442 +
TTATTAACA 349483 +
CTGTTAACA 349494 +
TTATTCACC 349843 +
>putR>
349200
349600

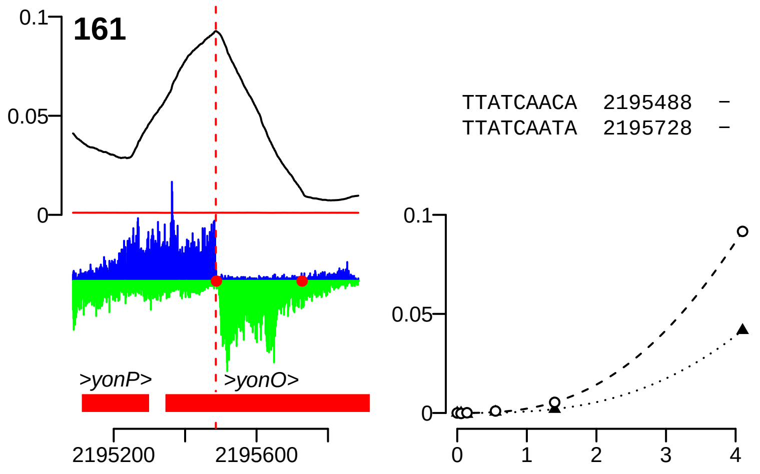
161
>yonP>
>yonO>
2195200
2195600
TTATCAACA 2195488 −
TTATCAATA 2195728 −

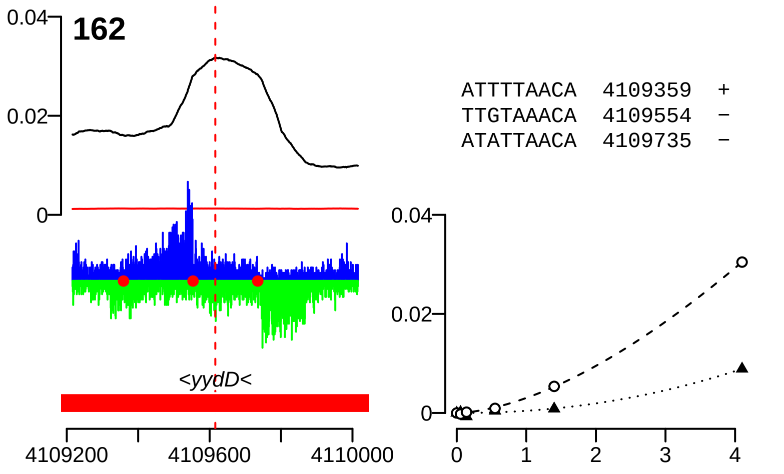
162
<yydD<
4109200
4109600
4110000
ATTTTAACA 4109359 +
TTGTAAACA 4109554 −
ATATTAACA 4109735 −

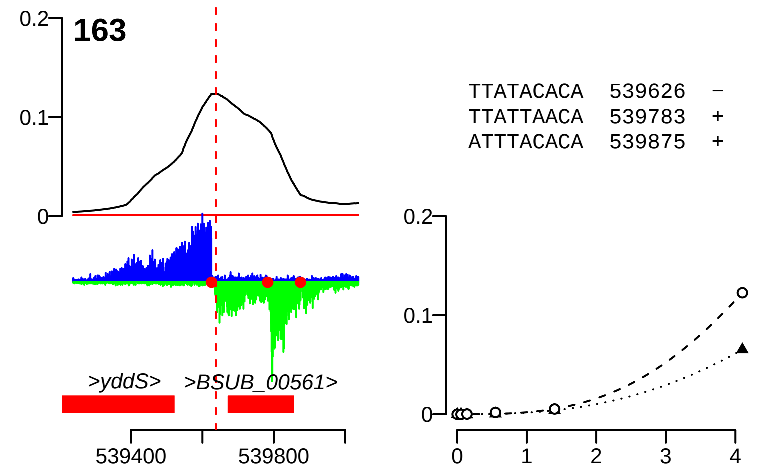
163
>yddS>
>BSUB_00561>
539400
539800
TTATACACA 539626 −
TTATTAACA 539783 +
ATTTACACA 539875 +

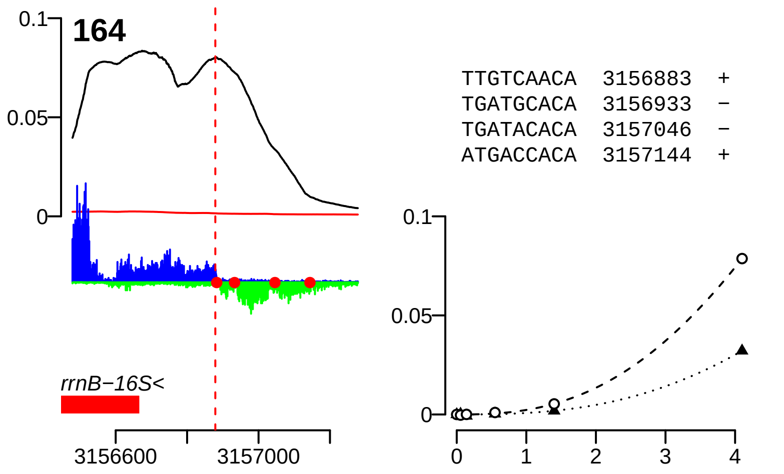
164
rrnB−16S<
3156600
3157000
TTGTCAACA 3156883 +
TGATGCACA 3156933 −
TGATACACA 3157046 −
ATGACCACA 3157144 +

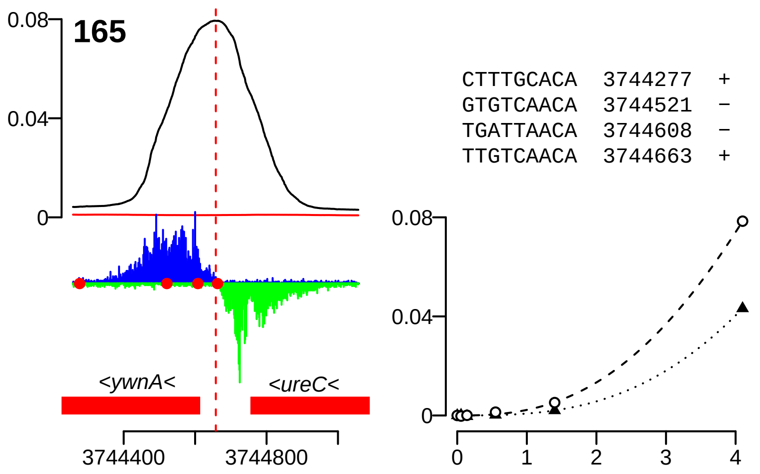
165
<ywnA<
<ureC<
3744400
3744800
CTTTGCACA 3744277 +
GTGTCAACA 3744521 −
TGATTAACA 3744608 −
TTGTCAACA 3744663 +

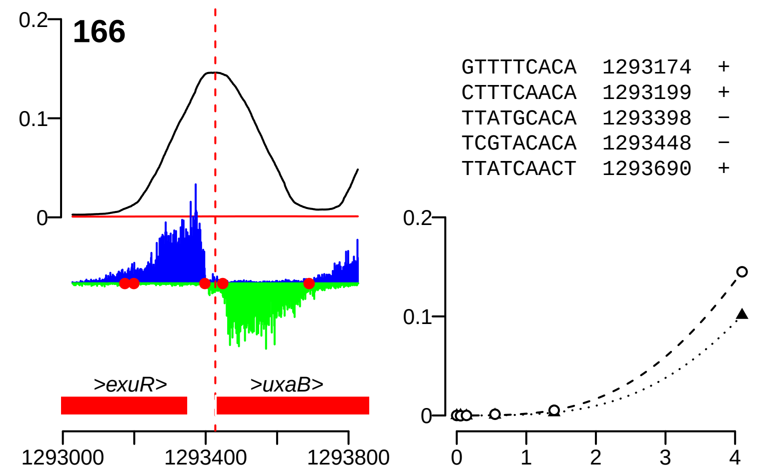
166
>exuR>
>uxaB>
1293000
1293400
1293800
GTTTTCACA 1293174 +
CTTTCAACA 1293199 +
TTATGCACA 1293398 −
TCGTACACA 1293448 −
TTATCAACT 1293690 +

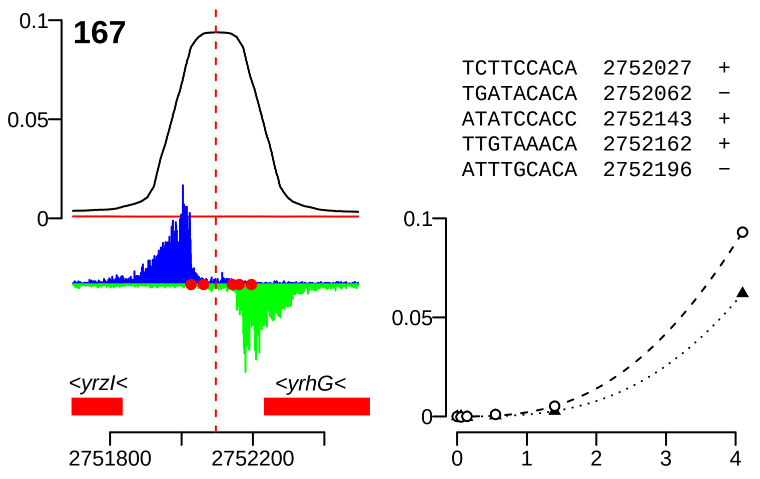
167
<yrzI<
<yrhG<
2751800
2752200
TCTTCCACA 2752027 +
TGATACACA 2752062 −
ATATCCACC 2752143 +
TTGTAAACA 2752162 +
ATTTGCACA 2752196 −

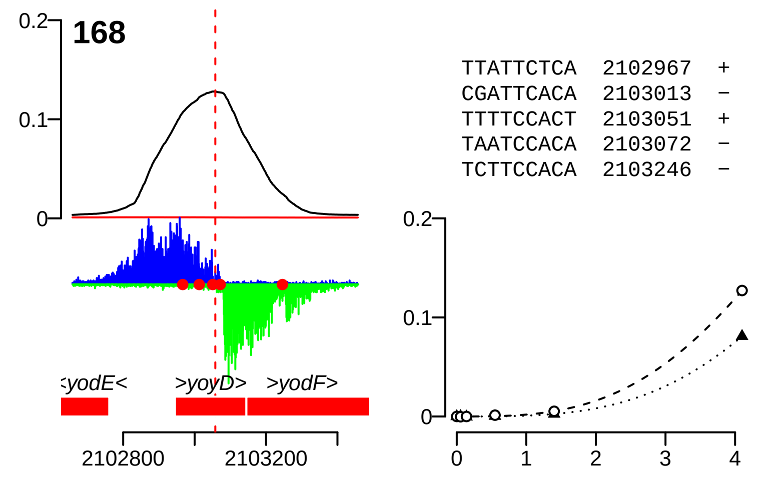
168
<yodE<
>yoyD>
>yodF>
2102800
2103200
TTATTCTCA 2102967 +
CGATTCACA 2103013 −
TTTTCCACT 2103051 +
TAATCCACA 2103072 −
TCTTCCACA 2103246 −

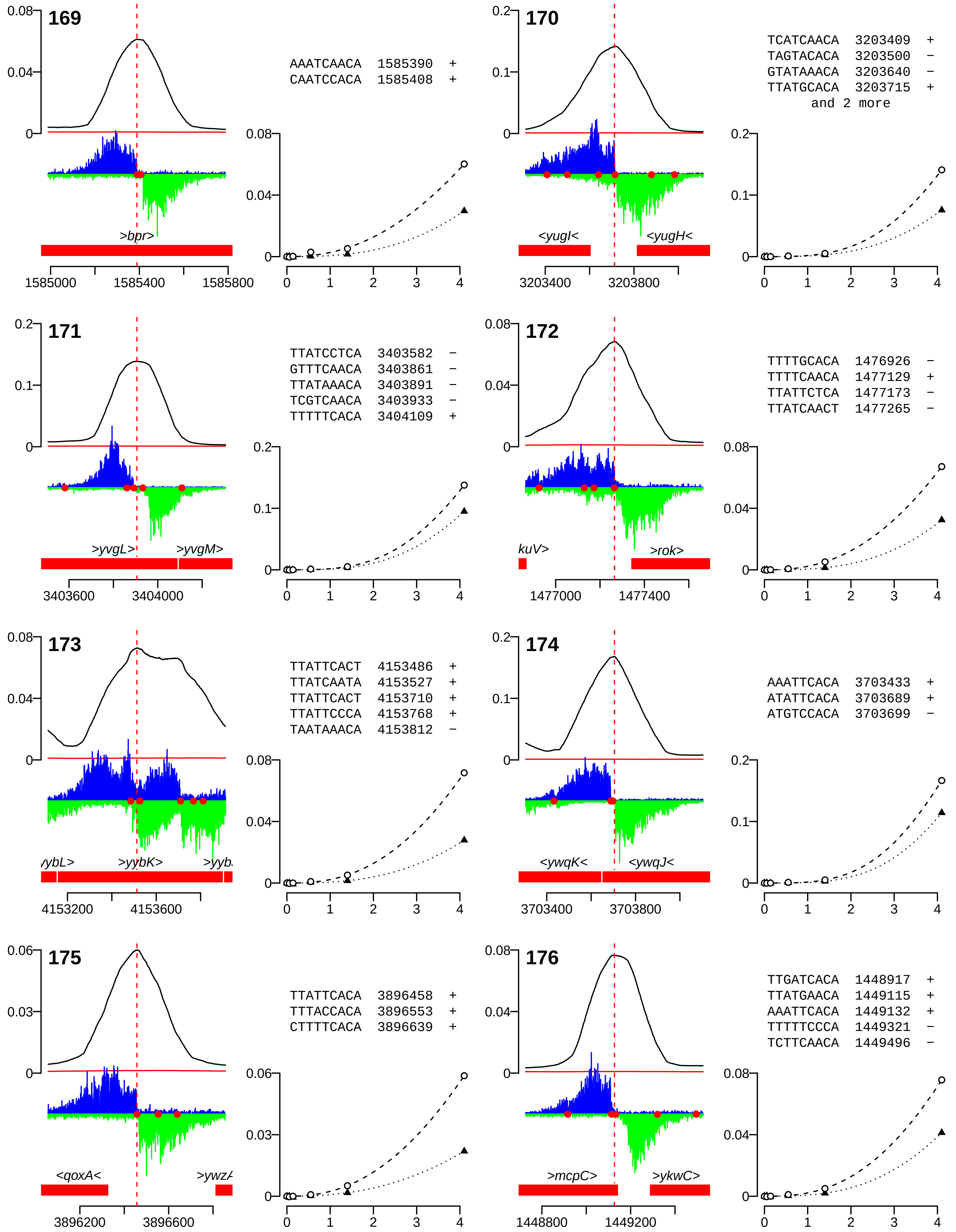

169
AAATCAACA 1585390 +
CAATCCACA 1585408 +
>bpr>
1585000
1585400
1585800
170
TCATCAACA 3203409 +
TAGTACACA 3203500 −
GTATAAACA 3203640 −
TTATGCACA 3203715 +
and 2 more
<yugI<
<yugH<
3203400
3203800
171
TTATCCTCA 3403582 −
GTTTCAACA 3403861 −
TTATAAACA 3403891 −
TCGTCAACA 3403933 −
TTTTTCACA 3404109 +
>yvgL>
>yvgM>
3403600
3404000
172
TTTTGCACA 1476926 −
TTTTCAACA 1477129 +
TTATTCTCA 1477173 −
TTATCAACT 1477265 −
kuV>
>rok>
1477000
1477400
173
TTATTCACT 4153486 +
TTATCAATA 4153527 +
TTATTCACT 4153710 +
TTATTCCCA 4153768 +
TAATAAACA 4153812 −
>yybK>
4153200
4153600
174
AAATTCACA 3703433 +
ATATTCACA 3703689 +
ATGTCCACA 3703699 −
<ywqK<
<ywqJ<
3703400
3703800
175
TTATTCACA 3896458 +
TTTACCACA 3896553 +
CTTTTCACA 3896639 +
<qoxA<
3896200
3896600
176
TTGATCACA 1448917 +
TTATGAACA 1449115 +
AAATTCACA 1449132 +
TTTTTCCCA 1449321 −
TCTTCAACA 1449496 −
>mcpC>
>ykwC>
1448800
1449200

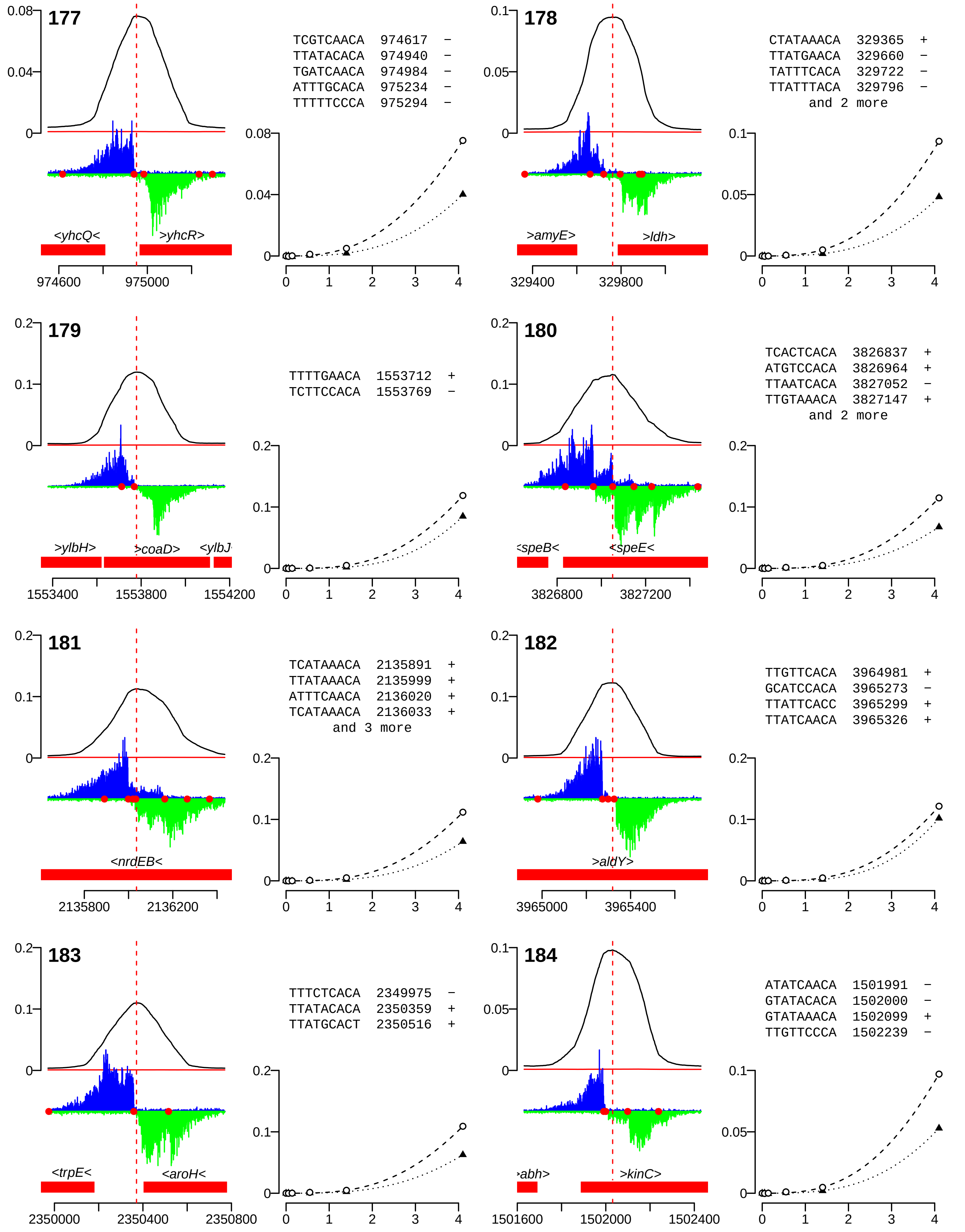
177
<yhcQ<
>yhcR>
974600
975000
TCGTCAACA 974617 −
TTATACACA 974940 −
TGATCAACA 974984 −
ATTTGCACA 975234 −
TTTTTCCCA 975294 −
178
>amyE>
>ldh>
329400
329800
CTATAAACA 329365 +
TTATGAACA 329660 −
TATTTCACA 329722 −
TTATTTACA 329796 −
and 2 more
179
>ylbH>
>coaD>
<ylbJ
1553400
1553800
1554200
TTTTGAACA 1553712 +
TCTTCCACA 1553769 −
180
<speB<
<speE<
3826800
3827200
TCACTCACA 3826837 +
ATGTCCACA 3826964 +
TTAATCACA 3827052 −
TTGTAAACA 3827147 +
and 2 more
181
<nrdEB<
2135800
2136200
TCATAAACA 2135891 +
TTATAAACA 2135999 +
ATTTCAACA 2136020 +
TCATAAACA 2136033 +
and 3 more
182
>aldY>
3965000
3965400
TTGTTCACA 3964981 +
GCATCCACA 3965273 −
TTATTCACC 3965299 +
TTATCAACA 3965326 +
183
<trpE<
<aroH<
2350000
2350400
2350800
TTTCTCACA 2349975 −
TTATACACA 2350359 +
TTATGCACT 2350516 +
184
abh>
>kinC>
1501600
1502000
1502400
ATATCAACA 1501991 −
GTATACACA 1502000 −
GTATAAACA 1502099 +
TTGTTCCCA 1502239 −

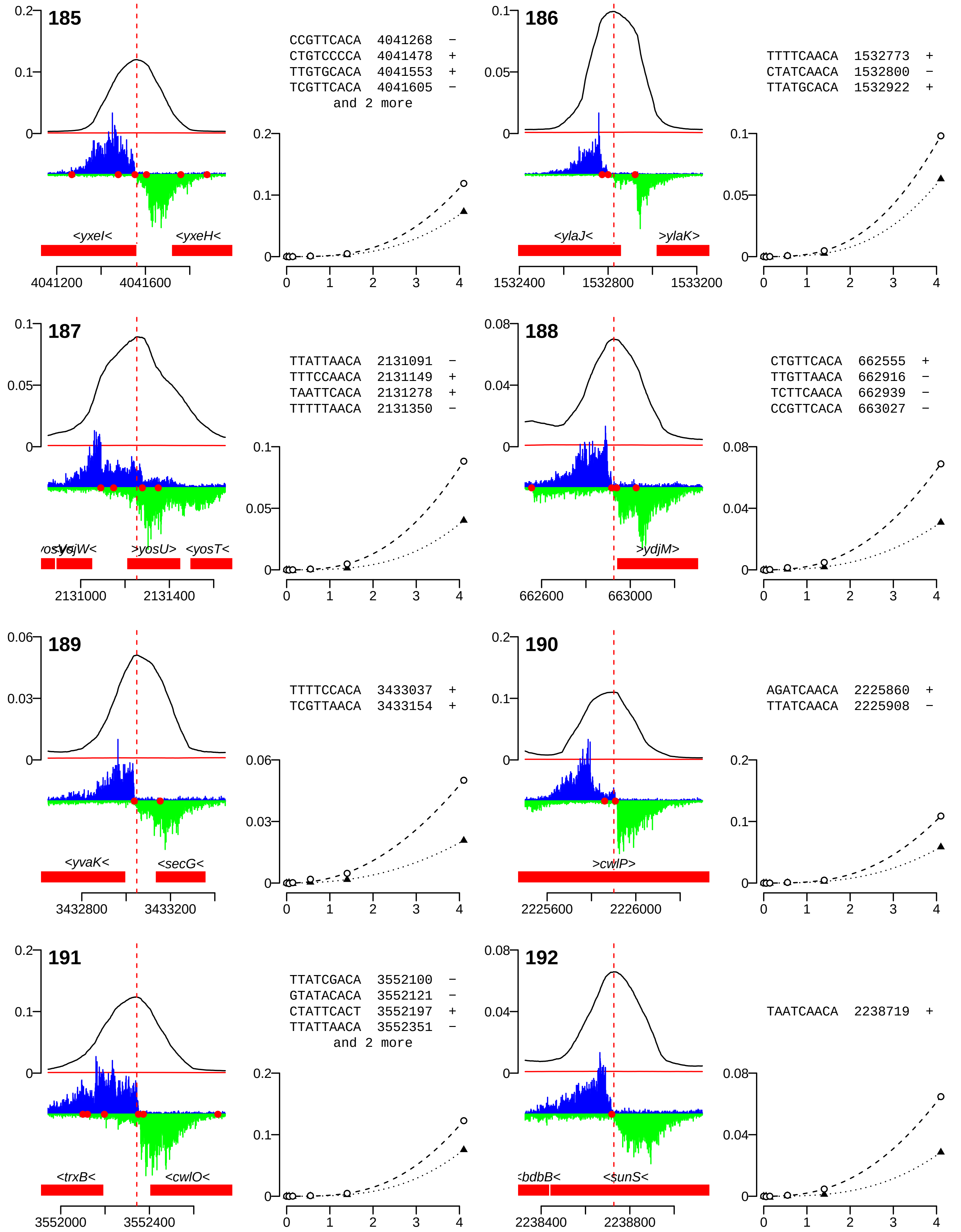

185
CCGTTCACA 4041268 −
CTGTCCCCA 4041478 +
TTGTGCACA 4041553 +
TCGTTCACA 4041605 −
and 2 more
<yxeI<
<yxeH<
4041200
4041600
186
TTTTCAACA 1532773 +
CTATCAACA 1532800 −
TTATGCACA 1532922 +
<ylaJ<
>ylaK>
1532400
1532800
1533200
187
TTATTAACA 2131091 −
TTTCCAACA 2131149 +
TAATTCACA 2131278 +
TTTTTAACA 2131350 −
>yosU>
<yosT<
2131000
2131400
188
CTGTTCACA 662555 +
TTGTTAACA 662916 −
TCTTCAACA 662939 −
CCGTTCACA 663027 −
>ydjM>
662600
663000
189
TTTTCCACA 3433037 +
TCGTTAACA 3433154 +
<yvaK<
<secG<
3432800
3433200
190
AGATCAACA 2225860 +
TTATCAACA 2225908 −
>cwlP>
2225600
2226000
191
TTATCGACA 3552100 −
GTATACACA 3552121 −
CTATTCACT 3552197 +
TTATTAACA 3552351 −
and 2 more
<trxB<
<cwlO<
3552000
3552400
192
TAATCAACA 2238719 +
<bdbB<
<sunS<
2238400
2238800

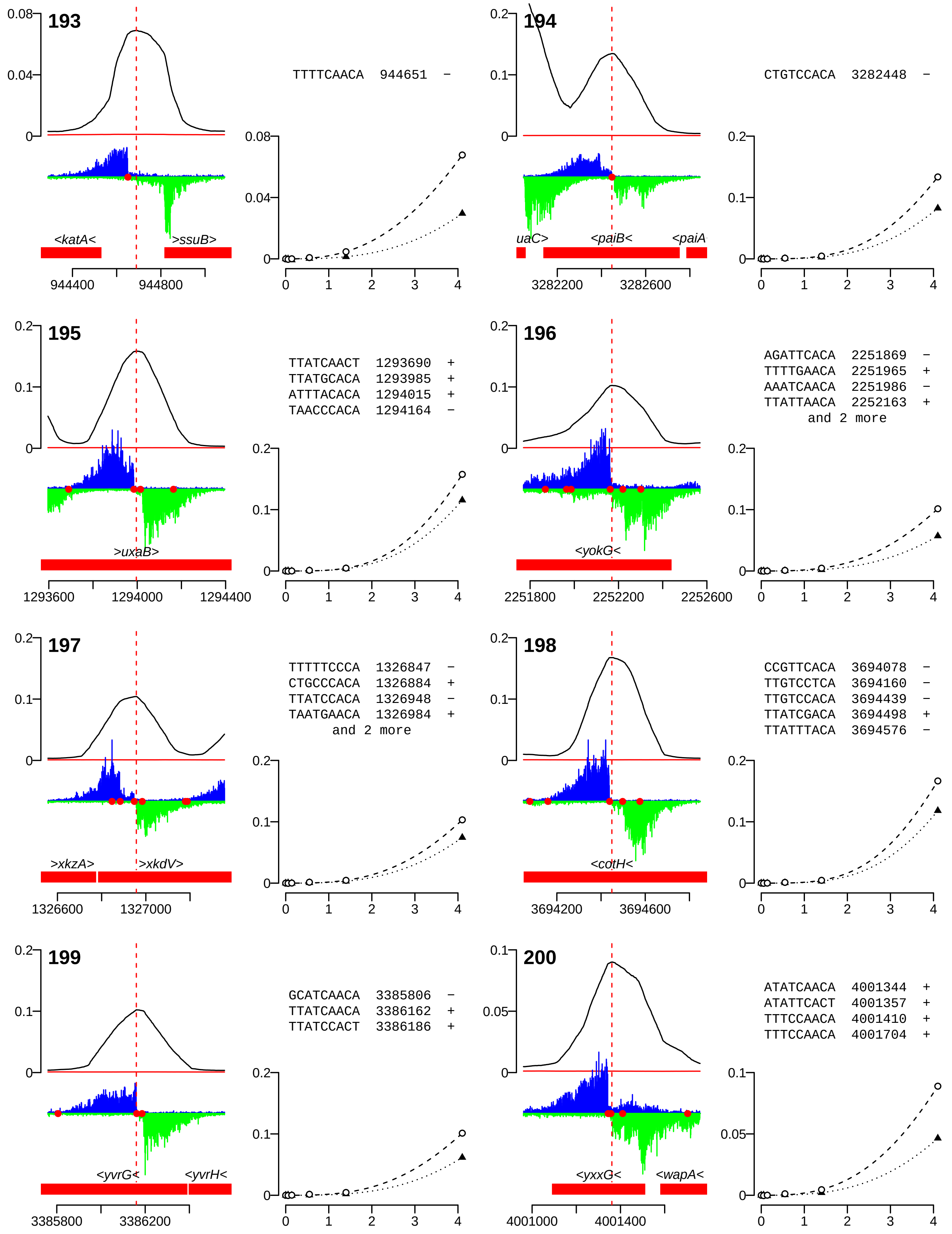
193
TTTTCAACA 944651 −
<katA<
>ssuB>
944400
944800
194
CTGTCCACA 3282448 −
uaC>
<paiB<
<paiA
3282200
3282600
195
TTATCAACT 1293690 +
TTATGCACA 1293985 +
ATTTACACA 1294015 +
TAACCCACA 1294164 −
>uxaB>
1293600
1294000
1294400
196
AGATTCACA 2251869 −
TTTTGAACA 2251965 +
AAATCAACA 2251986 −
TTATTAACA 2252163 +
and 2 more
<yokG<
2251800
2252200
2252600
197
TTTTTCCCA 1326847 −
CTGCCCACA 1326884 +
TTATCCACA 1326948 −
TAATGAACA 1326984 +
and 2 more
>xkzA>
>xkdV>
1326600
1327000
198
CCGTTCACA 3694078 −
TTGTCCTCA 3694160 −
TTGTCCACA 3694439 −
TTATCGACA 3694498 +
TTATTTACA 3694576 −
<cotH<
3694200
3694600
199
GCATCAACA 3385806 −
TTATCAACA 3386162 +
TTATCCACT 3386186 +
<yvrG<
<yvrH<
3385800
3386200
200
ATATCAACA 4001344 +
ATATTCACT 4001357 +
TTTCCAACA 4001410 +
TTTCCAACA 4001704 +
<yxxG<
<wapA<
4001000
4001400

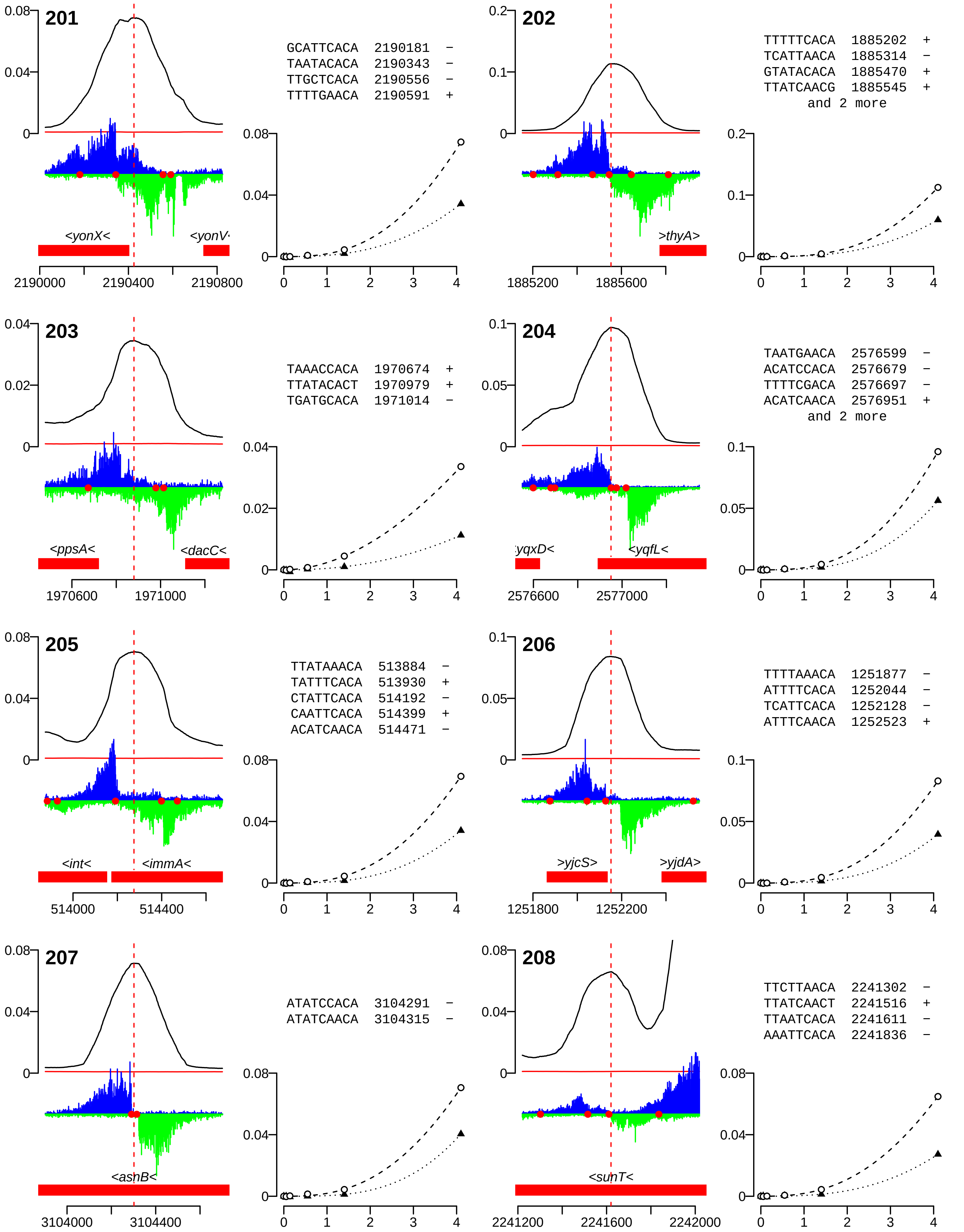

201
GCATTCACA 2190181 −
TAATACACA 2190343 −
TTGCTCACA 2190556 −
TTTTGAACA 2190591 +
<yonX<
<yonV
2190000 2190400 2190800
202
TTTTTCACA 1885202 +
TCATTAACA 1885314 −
GTATACACA 1885470 +
TTATCAACG 1885545 +
and 2 more
>thyA>
1885200 1885600
203
TAAACCACA 1970674 +
TTATACACT 1970979 +
TGATGCACA 1971014 −
<ppsA<
<dacC<
1970600 1971000
204
TAATGAACA 2576599 −
ACATCCACA 2576679 −
TTTTCGACA 2576697 −
ACATCAACA 2576951 +
and 2 more
yqxD<
<yqfL<
2576600 2577000
205
TTATAAACA 513884 −
TATTTCACA 513930 +
CTATTCACA 514192 −
CAATTCACA 514399 +
ACATCAACA 514471 −
<int<
<immA<
514000 514400
206
TTTTAAACA 1251877 −
ATTTTCACA 1252044 −
TCATTCACA 1252128 −
ATTTCAACA 1252523 +
>yjcS>
>yjdA>
1251800 1252200
207
ATATCCACA 3104291 −
ATATCAACA 3104315 −
<asnB<
3104000 3104400
208
TTCTTAACA 2241302 −
TTATCAACT 2241516 +
TTAATCACA 2241611 −
AAATTCACA 2241836 −
<sunT<
2241200 2241600 2242000

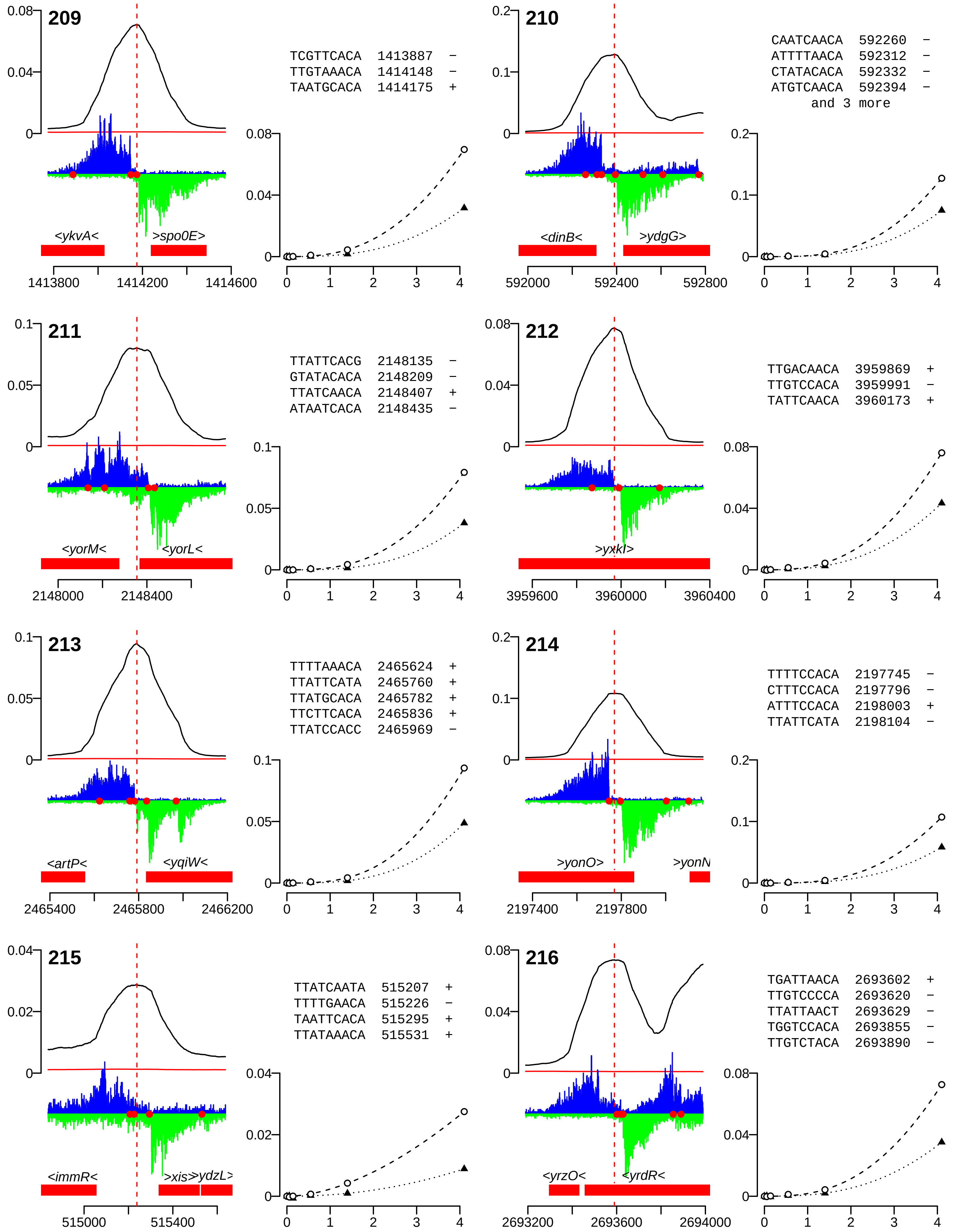
209
TCGTTCACA 1413887 −
TTGTAAACA 1414148 −
TAATGCACA 1414175 +
<ykvA<
>spo0E>
1413800
1414200
1414600
210
CAATCAACA 592260 −
ATTTTAACA 592312 −
CTATACACA 592332 −
ATGTCAACA 592394 −
and 3 more
<dinB<
>ydgG>
592000
592400
592800
211
TTATTCACG 2148135 −
GTATACACA 2148209 −
TTATCAACA 2148407 +
ATAATCACA 2148435 −
<yorM<
<yorL<
2148000
2148400
212
TTGACAACA 3959869 +
TTGTCCACA 3959991 −
TATTCAACA 3960173 +
>yxkI>
3959600
3960000
3960400
213
TTTTAAACA 2465624 +
TTATTCATA 2465760 +
TTATGCACA 2465782 +
TTCTTCACA 2465836 +
TTATCCACC 2465969 −
<artP<
<yqiW<
2465400
2465800
2466200
214
TTTTCCACA 2197745 −
CTTTCCACA 2197796 −
ATTTCCACA 2198003 +
TTATTCATA 2198104 −
>yonO>
>yonN
2197400
2197800
215
TTATCAATA 515207 +
TTTTGAACA 515226 −
TAATTCACA 515295 +
TTATAAACA 515531 +
<immR<
>xis>ydzL>
515000
515400
216
TGATTAACA 2693602 +
TTGTCCCCA 2693620 −
TTATTAACT 2693629 −
TGGTCCACA 2693855 −
TTGTCTACA 2693890 −
<yrzO<
<yrdR<
2693200
2693600
2694000

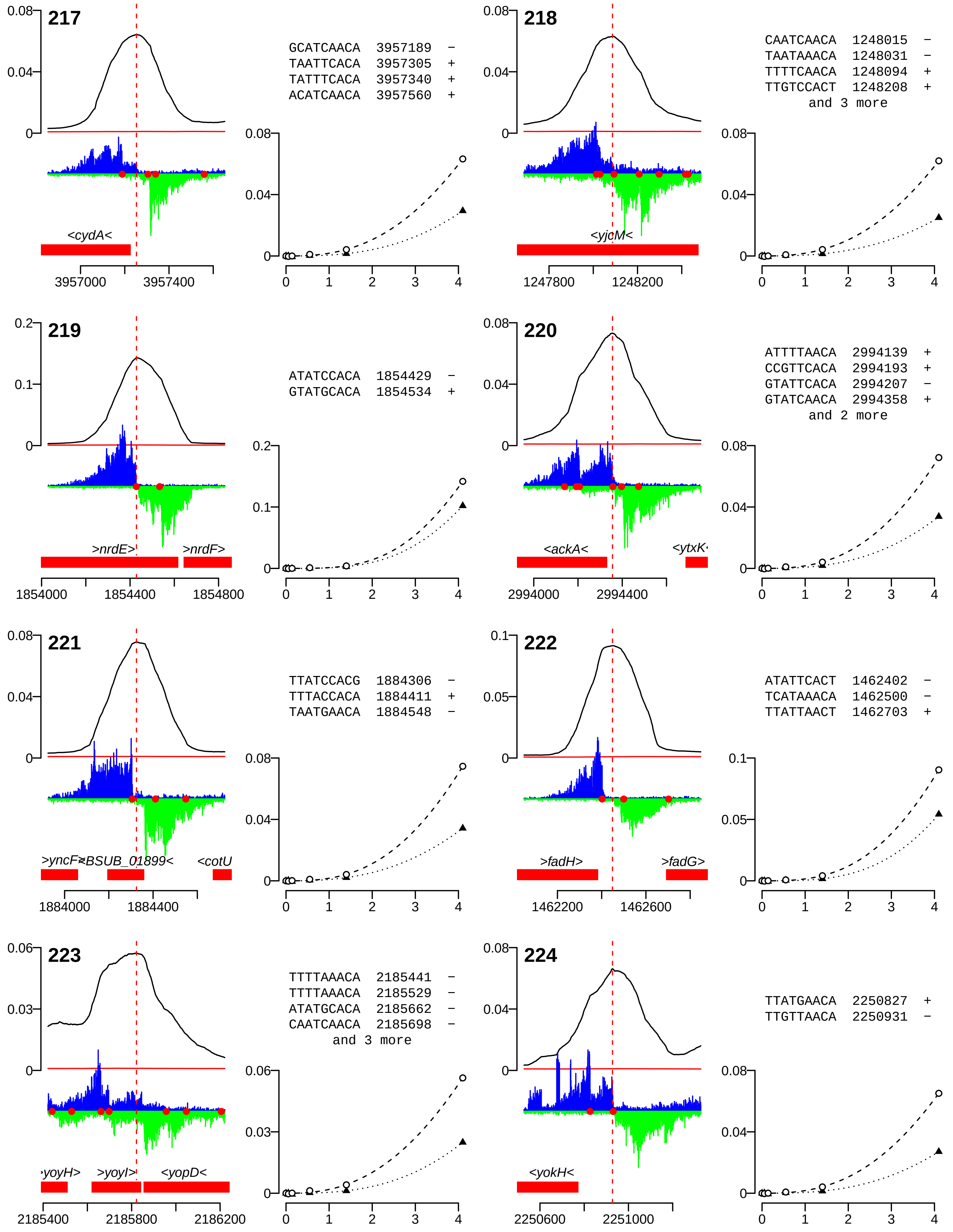
217
GCATCAACA 3957189 −
TAATTCACA 3957305 +
TATTTCACA 3957340 +
ACATCAACA 3957560 +
<cydA<
3957000
3957400
218
CAATCAACA 1248015 −
TAATAAACA 1248031 −
TTTTCAACA 1248094 +
TTGTCCACT 1248208 +
and 3 more
<yjcM<
1247800
1248200
219
ATATCCACA 1854429 −
GTATGCACA 1854534 +
>nrdE>
>nrdF>
1854000
1854400
1854800
220
ATTTTAACA 2994139 +
CCGTTCACA 2994193 +
GTATTCACA 2994207 −
GTATCAACA 2994358 +
and 2 more
<ackA<
2994000
2994400
221
TTATCCACG 1884306 −
TTTACCACA 1884411 +
TAATGAACA 1884548 −
>yncF>
<BSUB_01899<
<cotU
1884000
1884400
222
ATATTCACT 1462402 −
TCATAAACA 1462500 −
TTATTAACT 1462703 +
>fadH>
>fadG>
1462200
1462600
223
TTTTAAACA 2185441 −
TTTTAAACA 2185529 −
ATATGCACA 2185662 −
CAATCAACA 2185698 −
and 3 more
>yoyI>
<yopD<
2185400
2185800
2186200
224
TTATGAACA 2250827 +
TTGTTAACA 2250931 −
<yokH<
2250600
2251000

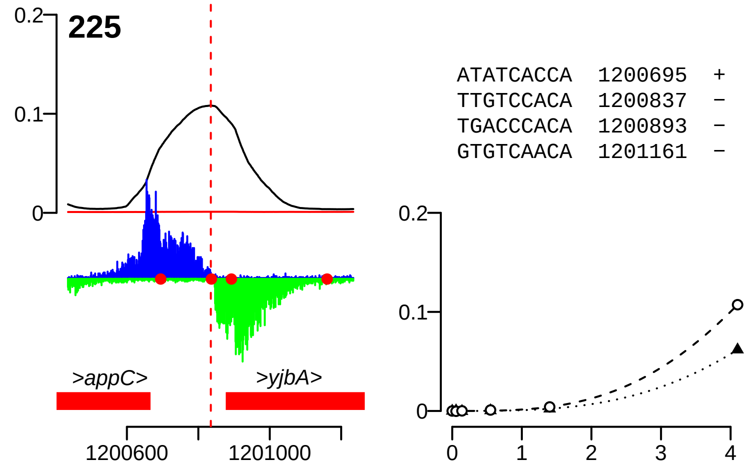
225
ATATCACCA 1200695 +
TTGTCCACA 1200837 −
TGACCCACA 1200893 −
GTGTCAACA 1201161 −
>appC>
>yjbA>
1200600
1201000
0.2
0.1
0

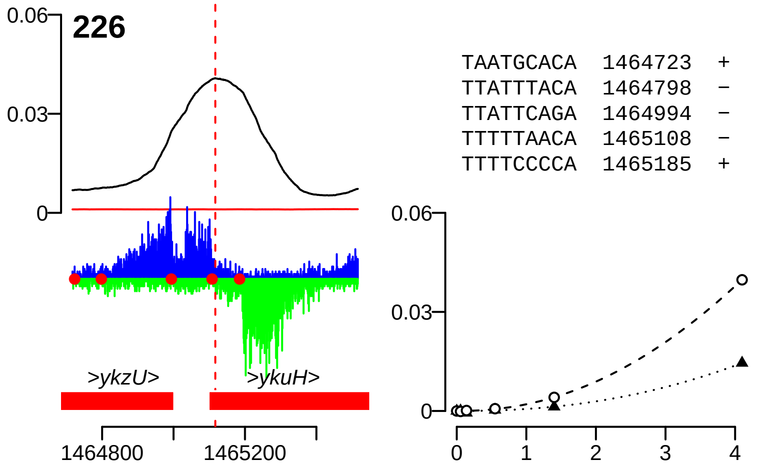
226
TAATGCACA 1464723 +
TTATTTACA 1464798 −
TTATTCAGA 1464994 −
TTTTTAACA 1465108 −
TTTTCCCCA 1465185 +
>ykzU>
>ykuH>
1464800
1465200
0.06
0.03
0

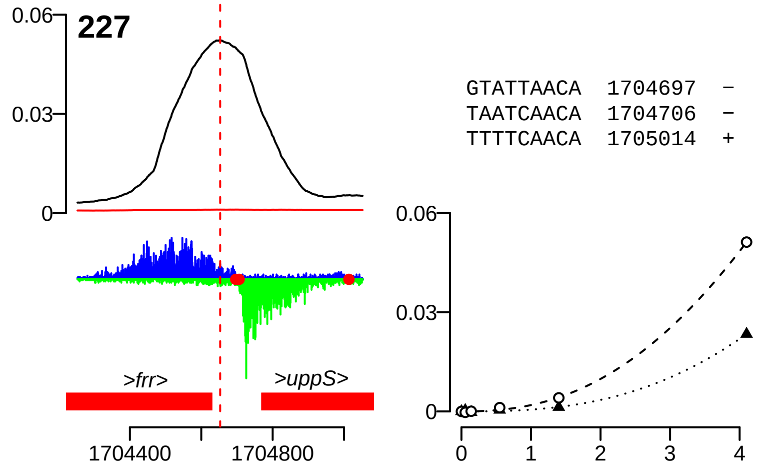
227
GTATTAACA 1704697 −
TAATCAACA 1704706 −
TTTTCAACA 1705014 +
>frr>
>uppS>
1704400
1704800
0.06
0.03
0

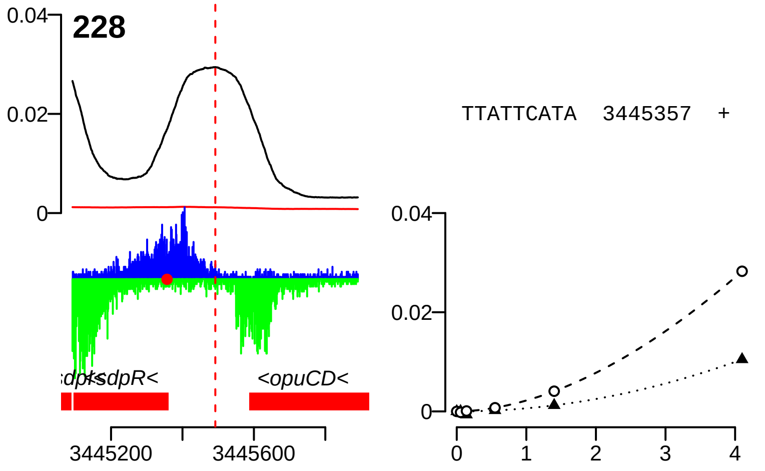
228
TTATTCATA 3445357 +
<sdpR<
<opuCD<
3445200
3445600
0.04
0.02
0

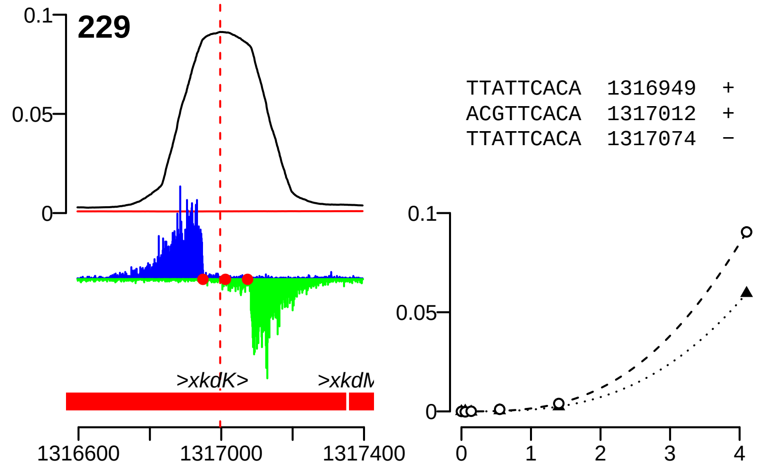
229
TTATTCACA 1316949 +
ACGTTCACA 1317012 +
TTATTCACA 1317074 −
>xkdK>
1316600
1317000
1317400
0.1
0.05
0

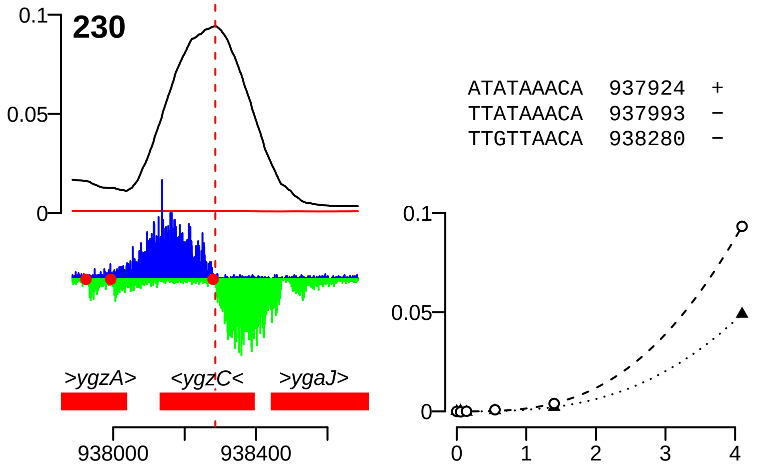
230
ATATAAACA 937924 +
TTATAAACA 937993 −
TTGTTAACA 938280 −
>ygzA>
<ygzC<
>ygaJ>
938000
938400
0.1
0.05
0

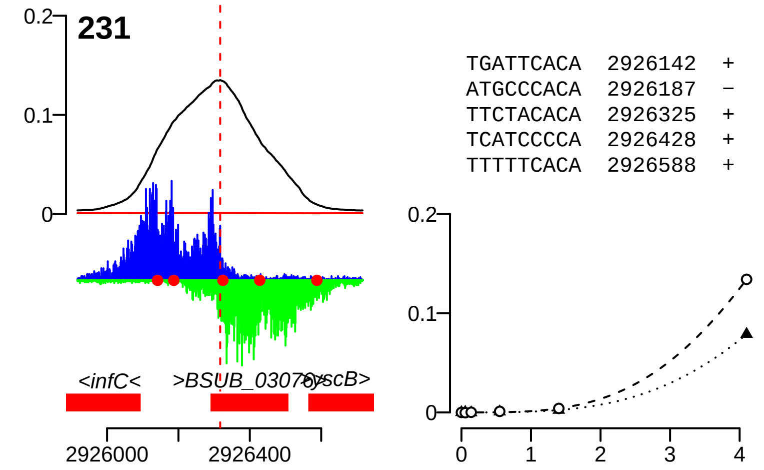
231
TGATTCACA 2926142 +
ATGCCCACA 2926187 −
TTCTACACA 2926325 +
TCATCCCCA 2926428 +
TTTTTCACA 2926588 +
<infC<
2926000
2926400
0.2
0.1
0

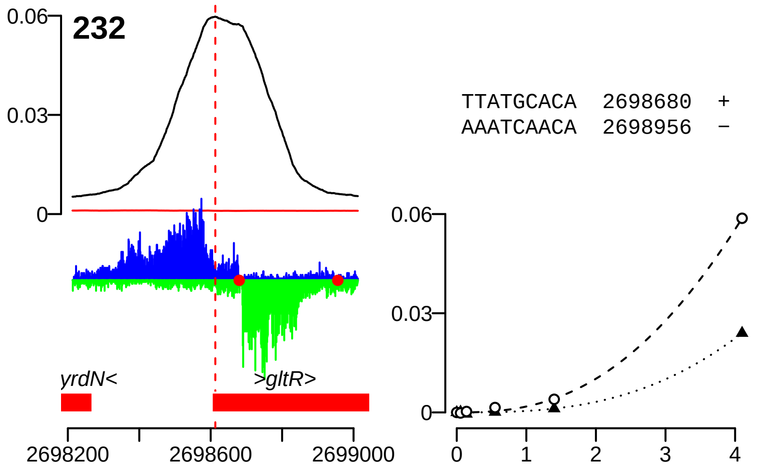
232
TTATGCACA 2698680 +
AAATCAACA 2698956 −
yrdN<
>gltR>
2698200
2698600
2699000
0.06
0.03
0

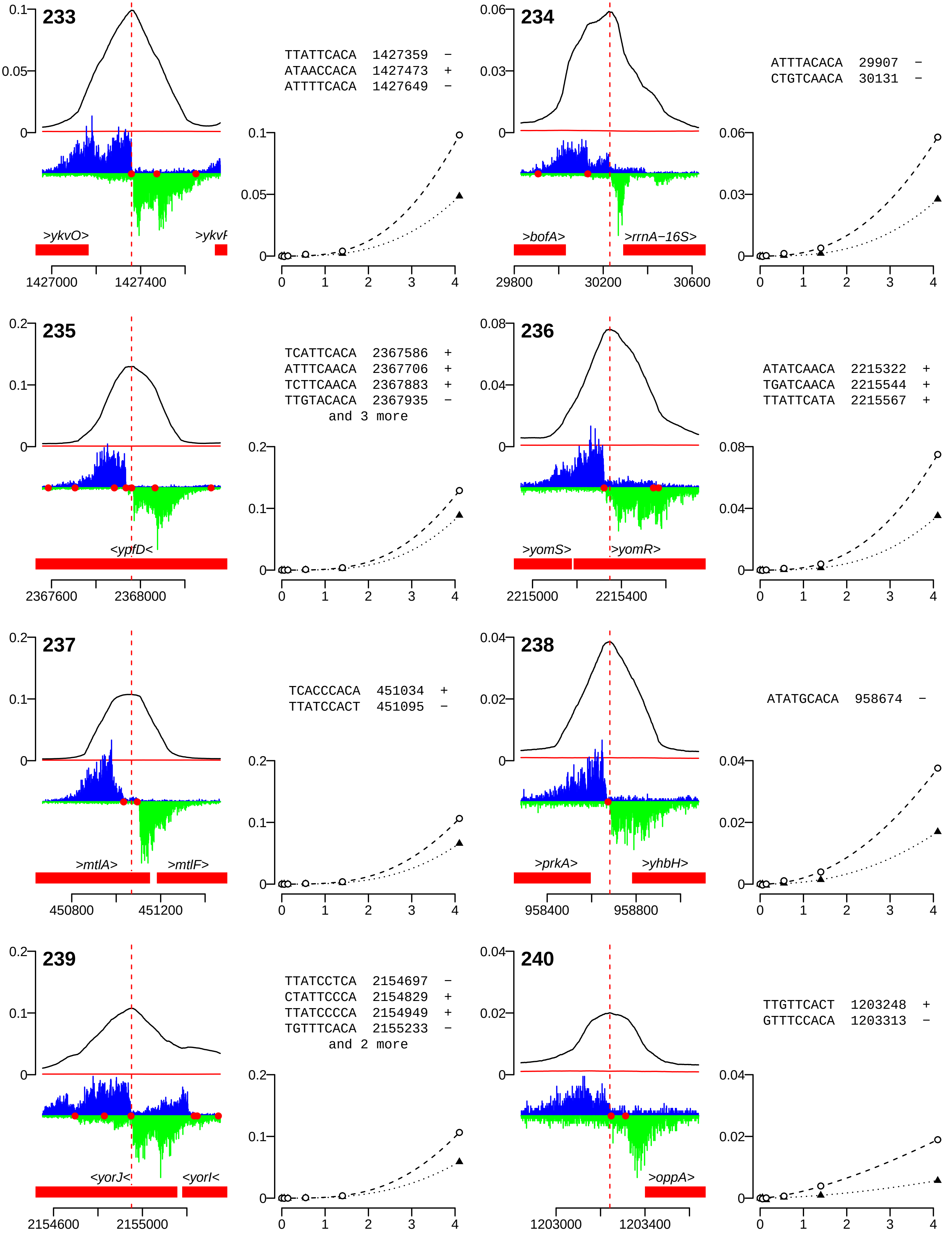
233
TTATTCACA 1427359 −
ATAACCACA 1427473 +
ATTTTCACA 1427649 −
>ykvO>
1427000
1427400
234
ATTTACACA 29907 −
CTGTCAACA 30131 −
>bofA>
>rrnA−16S>
29800
30200
30600
235
TCATTCACA 2367586 +
ATTTCAACA 2367706 +
TCTTCAACA 2367883 +
TTGTACACA 2367935 −
and 3 more
<ypfD<
2367600
2368000
236
ATATCAACA 2215322 +
TGATCAACA 2215544 +
TTATTCATA 2215567 +
>yomS>
>yomR>
2215000
2215400
237
TCACCCACA 451034 +
TTATCCACT 451095 −
>mtlA>
>mtlF>
450800
451200
238
ATATGCACA 958674 −
>prkA>
>yhbH>
958400
958800
239
TTATCCTCA 2154697 −
CTATTCCCA 2154829 +
TTATCCCCA 2154949 +
TGTTTCACA 2155233 −
and 2 more
<yorJ<
<yorI<
2154600
2155000
240
TTGTTCACT 1203248 +
GTTTCCACA 1203313 −
>oppA>
1203000
1203400

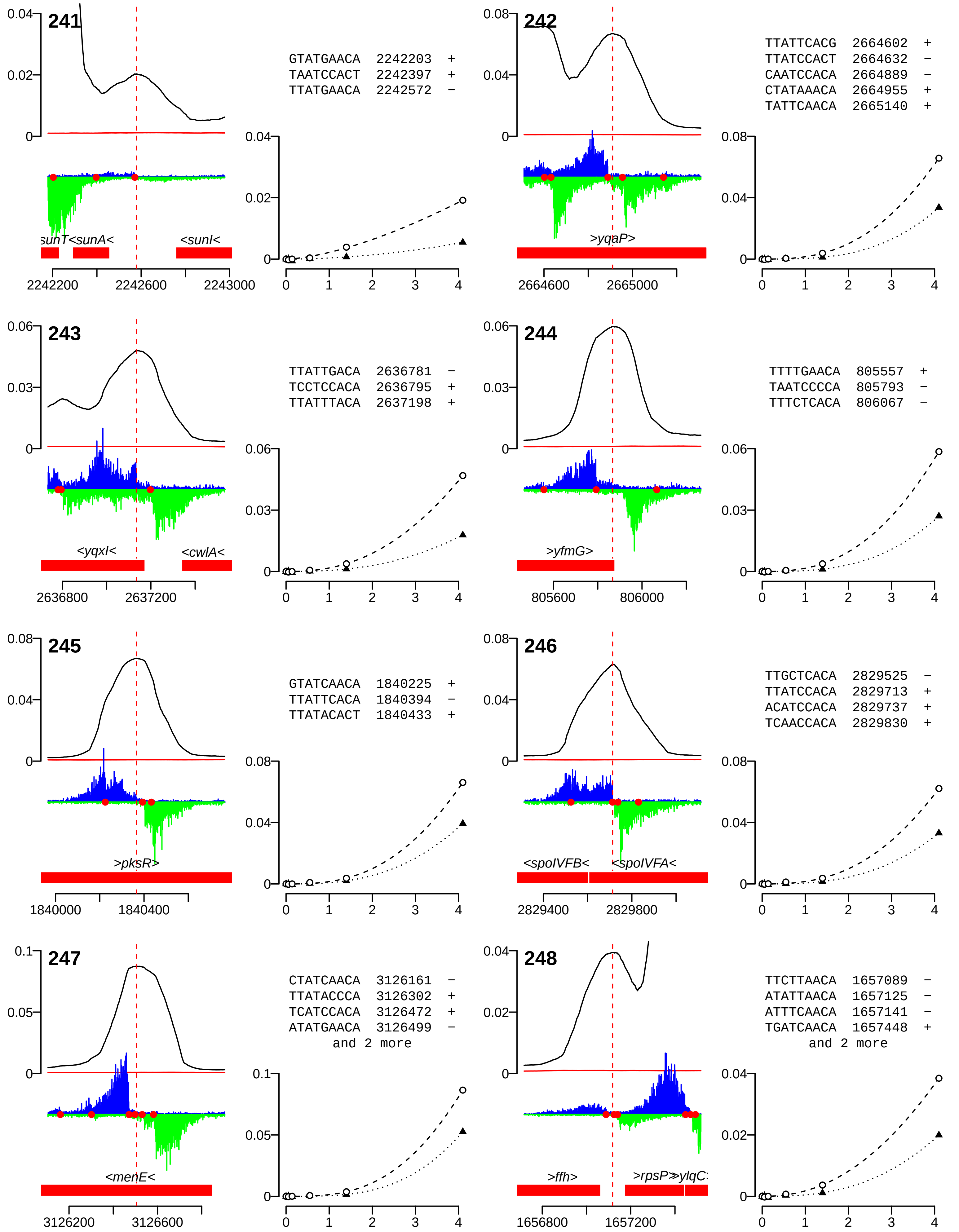
241
GTATGAACA 2242203 +
TAATCCACT 2242397 +
TTATGAACA 2242572 −
sunT<sunA<
<sunI<
2242200
2242600
2243000
242
TTATTCACG 2664602 +
TTATCCACT 2664632 −
CAATCCACA 2664889 −
CTATAAACA 2664955 +
TATTCAACA 2665140 +
>yqaP>
2664600
2665000
243
TTATTGACA 2636781 −
TCCTCCACA 2636795 +
TTATTTACA 2637198 +
<yqxI<
<cwlA<
2636800
2637200
244
TTTTGAACA 805557 +
TAATCCCCA 805793 −
TTTCTCACA 806067 −
>yfmG>
805600
806000
245
GTATCAACA 1840225 +
TTATTCACA 1840394 −
TTATACACT 1840433 +
>pksR>
1840000
1840400
246
TTGCTCACA 2829525 −
TTATCCACA 2829713 +
ACATCCACA 2829737 +
TCAACCACA 2829830 +
<spoIVFB<
<spoIVFA<
2829400
2829800
247
CTATCAACA 3126161 −
TTATACCCA 3126302 +
TCATCCACA 3126472 +
ATATGAACA 3126499 −
and 2 more
<menE<
3126200
3126600
248
TTCTTAACA 1657089 −
ATATTAACA 1657125 −
ATTTCAACA 1657141 −
TGATCAACA 1657448 +
and 2 more
>ffh>
>rpsP>>ylqC:
1656800
1657200

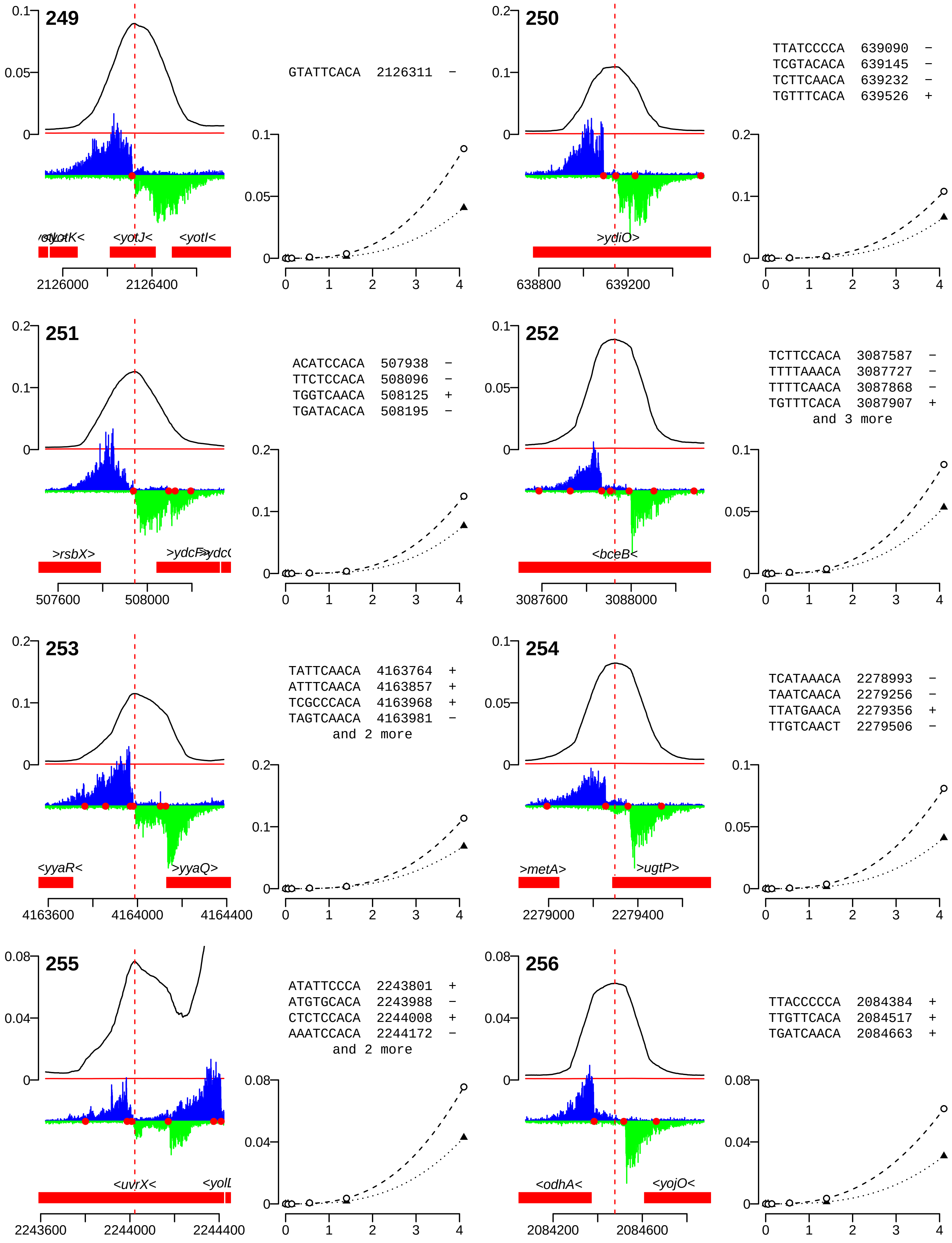

249
GTATTCACA 2126311 −
<yotJ<
<yotI<
2126000
2126400
250
TTATCCCCA 639090 −
TCGTACACA 639145 −
TCTTCAACA 639232 −
TGTTTCACA 639526 +
>ydiO>
638800
639200
251
ACATCCACA 507938 −
TTCTCCACA 508096 −
TGGTCAACA 508125 +
TGATACACA 508195 −
>rsbX>
507600
508000
252
TCTTCCACA 3087587 −
TTTTAAACA 3087727 −
TTTTCAACA 3087868 −
TGTTTCACA 3087907 +
and 3 more
<bceB<
3087600
3088000
253
TATTCAACA 4163764 +
ATTTCAACA 4163857 +
TCGCCCACA 4163968 +
TAGTCAACA 4163981 −
and 2 more
<yyaR<
>yyaQ>
4163600
4164000
4164400
254
TCATAAACA 2278993 −
TAATCAACA 2279256 −
TTATGAACA 2279356 +
TTGTCAACT 2279506 −
>metA>
>ugtP>
2279000
2279400
255
ATATTCCCA 2243801 +
ATGTGCACA 2243988 −
CTCTCCACA 2244008 +
AAATCCACA 2244172 −
and 2 more
<uvrX<
2243600
2244000
2244400
256
TTACCCCCA 2084384 +
TTGTTCACA 2084517 +
TGATCAACA 2084663 +
<odhA<
<yojO<
2084200
2084600

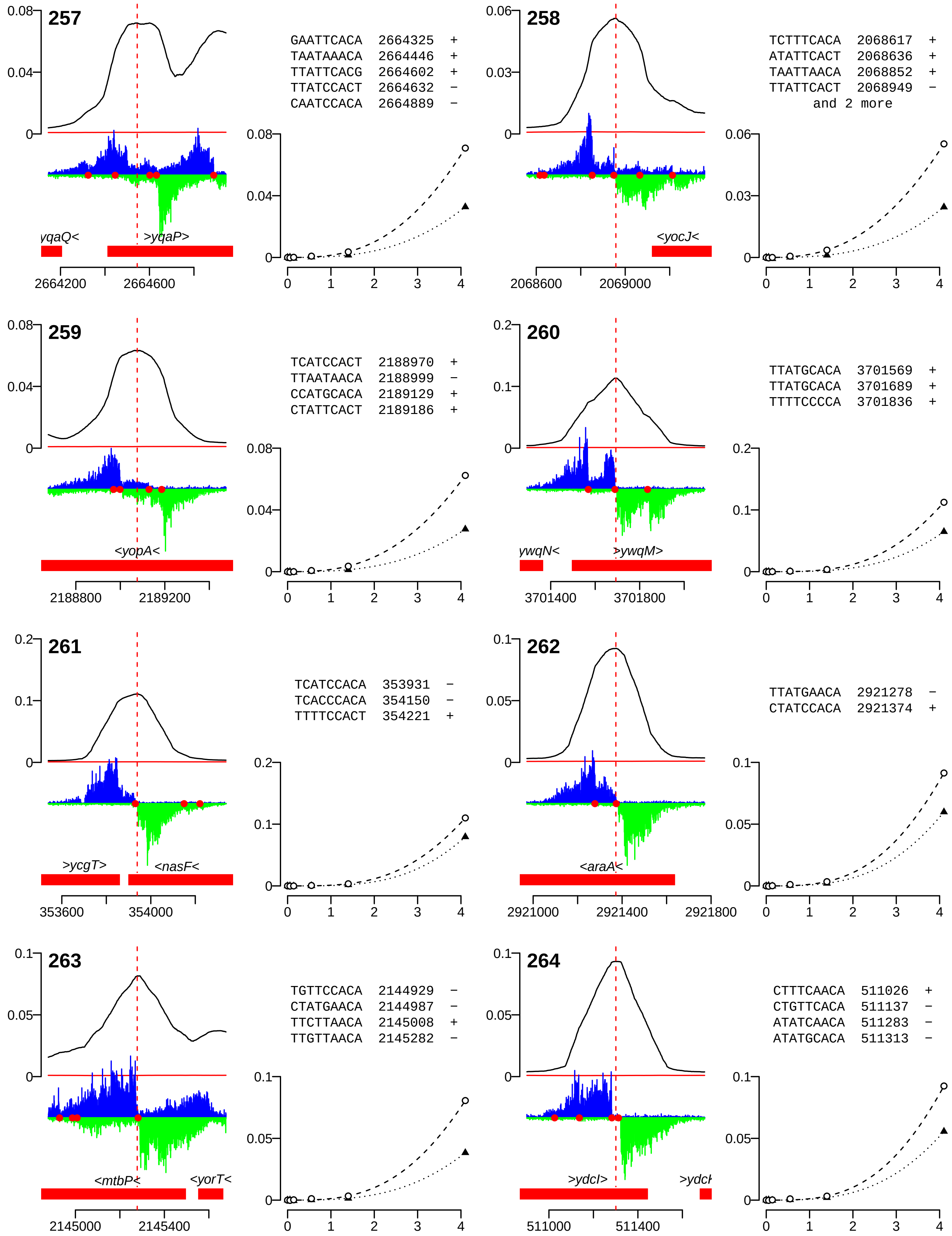
257
GAATTCACA 2664325 +
TAATAAACA 2664446 +
TTATTCACG 2664602 +
TTATCCACT 2664632 −
CAATCCACA 2664889 −
yqaQ<
>yqaP>
2664200
2664600
258
TCTTTCACA 2068617 +
ATATTCACT 2068636 +
TAATTAACA 2068852 +
TTATTCACT 2068949 −
and 2 more
<yocJ<
2068600
2069000
259
TCATCCACT 2188970 +
TTAATAACA 2188999 −
CCATGCACA 2189129 +
CTATTCACT 2189186 +
<yopA<
2188800
2189200
260
TTATGCACA 3701569 +
TTATGCACA 3701689 +
TTTTCCCCA 3701836 +
ywqN<
>ywqM>
3701400
3701800
261
TCATCCACA 353931 −
TCACCCACA 354150 −
TTTTCCACT 354221 +
>ycgT>
<nasF<
353600
354000
262
TTATGAACA 2921278 −
CTATCCACA 2921374 +
<araA<
2921000
2921400
2921800
263
TGTTCCACA 2144929 −
CTATGAACA 2144987 −
TTCTTAACA 2145008 +
TTGTTAACA 2145282 −
<mtbP<
<yorT<
2145000
2145400
264
CTTTCAACA 511026 +
CTGTTCACA 511137 −
ATATCAACA 511283 −
ATATGCACA 511313 −
>ydcI>
2145000
511000
511400

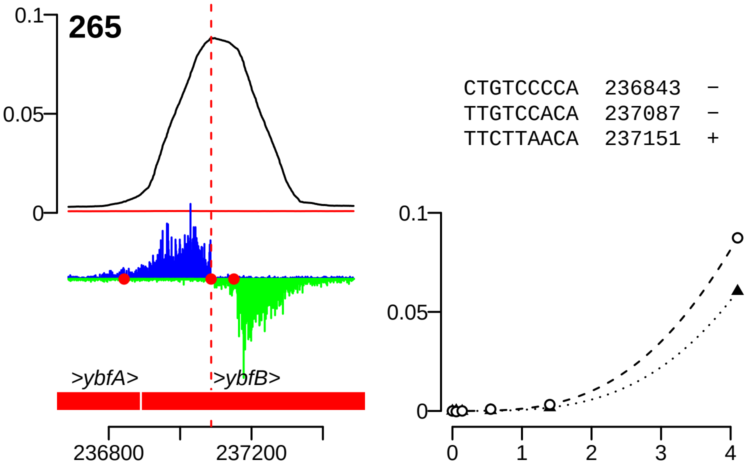
265
CTGTCCCCA 236843 −
TTGTCCACA 237087 −
TTCTTAACA 237151 +
>ybfA>
>ybfB>
236800
237200
0.1
0.05
0
1
2
3
4

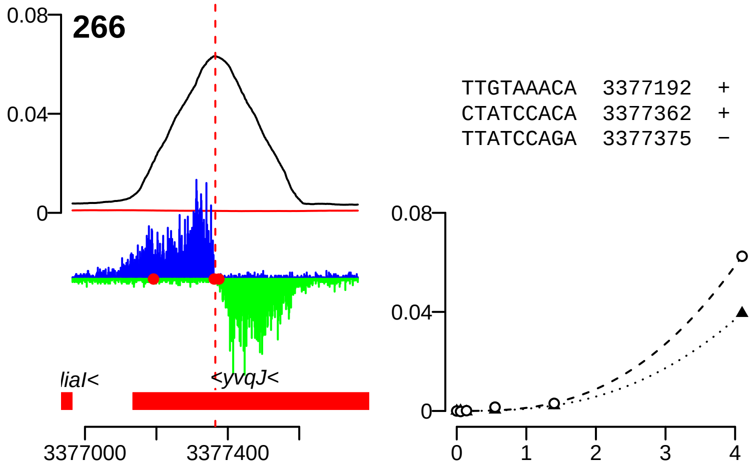
266
TTGTAAACA 3377192 +
CTATCCACA 3377362 +
TTATCCAGA 3377375 −
liaI<
<yvqJ<
3377000
3377400
0.08
0.04
0
1
2
3
4

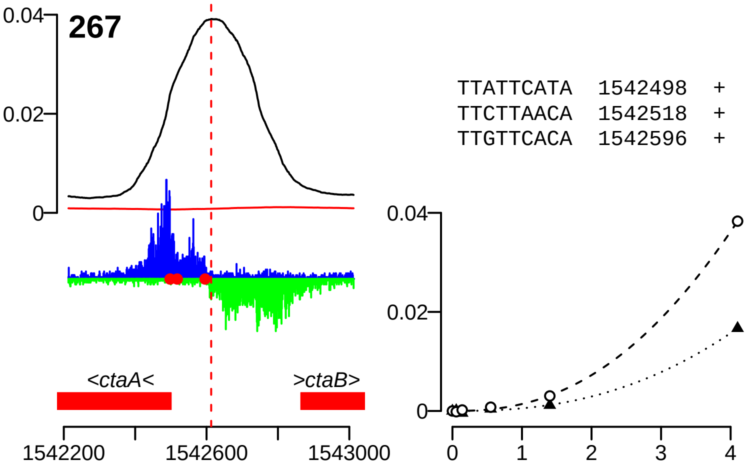
267
TTATTCATA 1542498 +
TTCTTAACA 1542518 +
TTGTTCACA 1542596 +
<ctaA<
>ctaB>
1542200
1542600
1543000
0.04
0.02
0
1
2
3
4

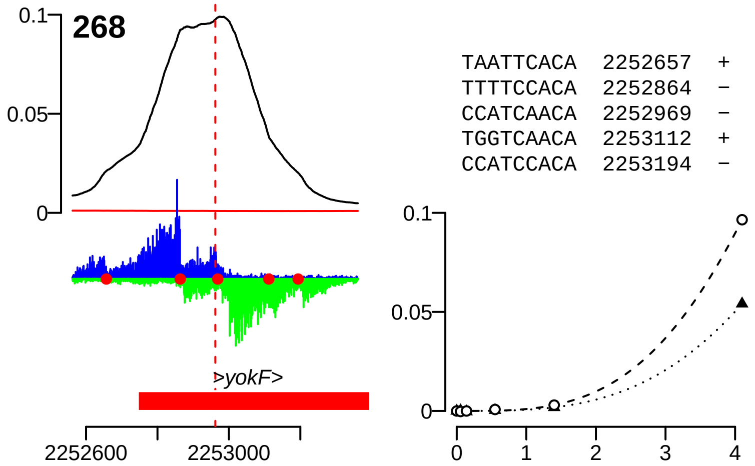
268
TAATTCACA 2252657 +
TTTTCCACA 2252864 −
CCATCAACA 2252969 −
TGGTCAACA 2253112 +
CCATCCACA 2253194 −
>yokF>
2252600
2253000
0.1
0.05
0
1
2
3
4

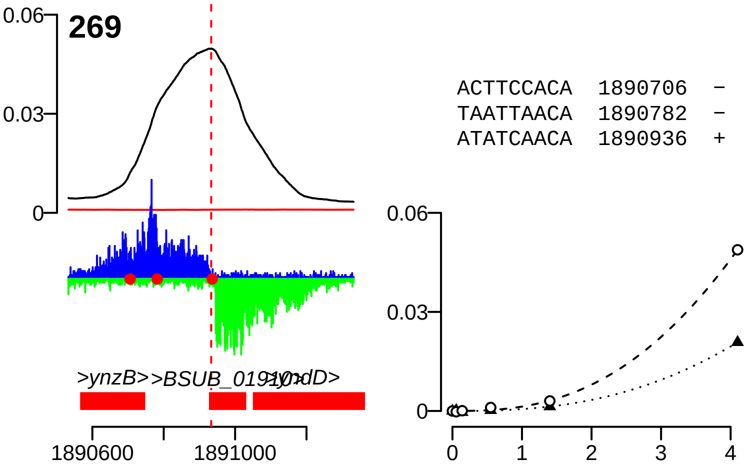
269
ACTTCCACA 1890706 −
TAATTAACA 1890782 −
ATATCAACA 1890936 +
>ynzB>
>BSUB_01910>yndD>
1890600
1891000
0.06
0.03
0
1
2
3
4
