# Supplementary figures and images for: In Vitro Whole Genome DNA Binding Analysis of the Bacterial Replication Initiator and Transcription Factor DnaA
Source: PLoS Genet. 2015 May 28;11(5):e1005258. doi: 10.1371/journal.pgen.1005258 (PMC4447404; doi:10.1371/journal.pgen.1005258)

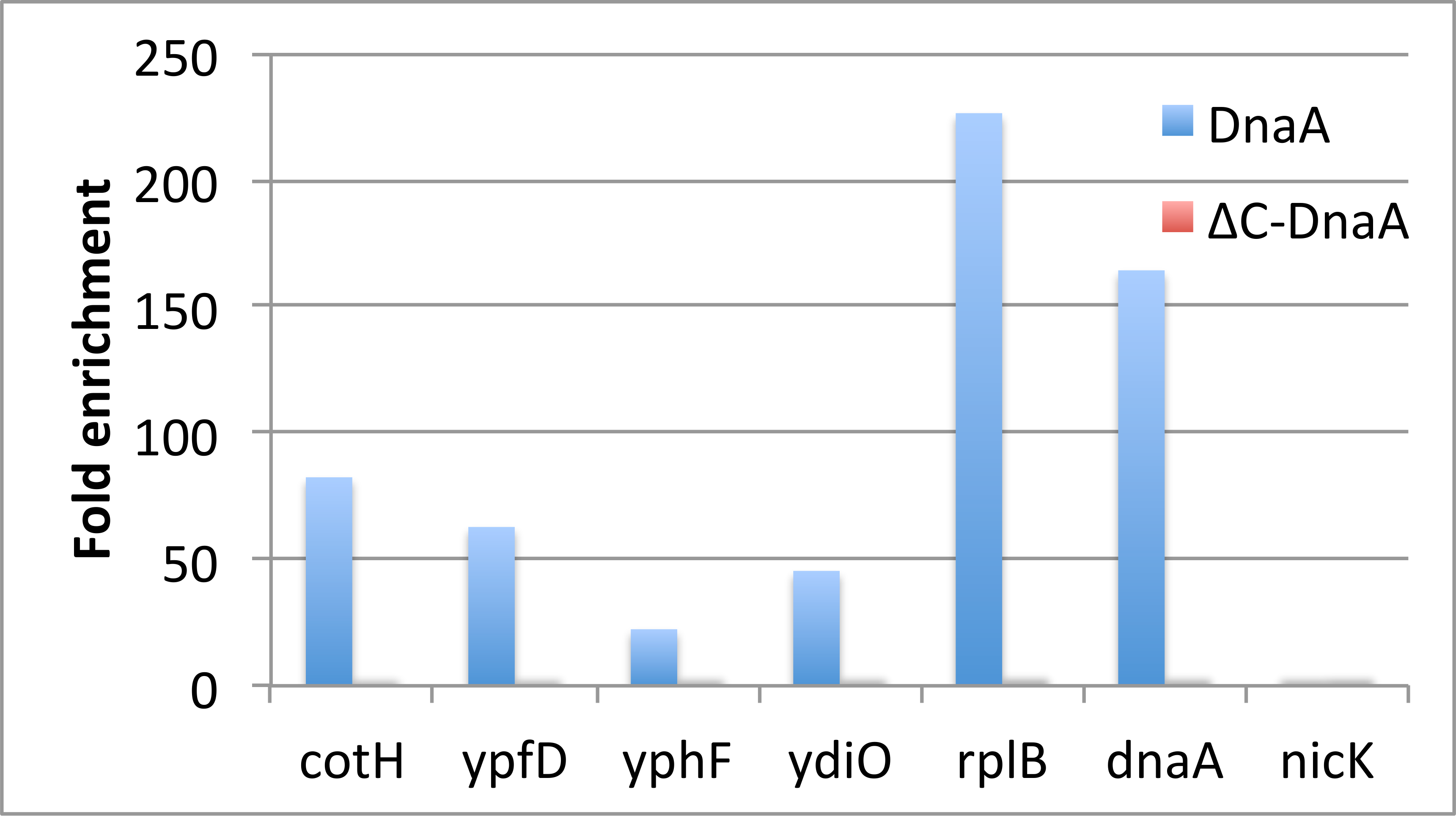

Supplement: S2 Fig — Binding reactions were performed under the same conditions as for DnaA-his, except that 4.1 μM DnaA∆C-his was used. DnaA∆C lacks the C-terminal 91 amino acids that are required for DNA binding. A reaction containing full-length DnaA-his was performed in parallel. The binding reactions contained 2.5 mM ATP. The recovered DNA was assayed using qPCR, with the primers indicated in S5 Table. The following loci were assayed (peak numbers refer to those in S1 Fig and S1 Table): cotH (peak #198), ypfD (peak #235), yphF (too weak to be called as a peak at 1.4 μM DnaA but clearly discernible at 4.1 μM), ydiO (peak #250), rplB (peak #10), dnaA (peak #1), and nicK, a control region that does not bind DnaA. (TIFF) [file pgen.1005258.s002.tiff]

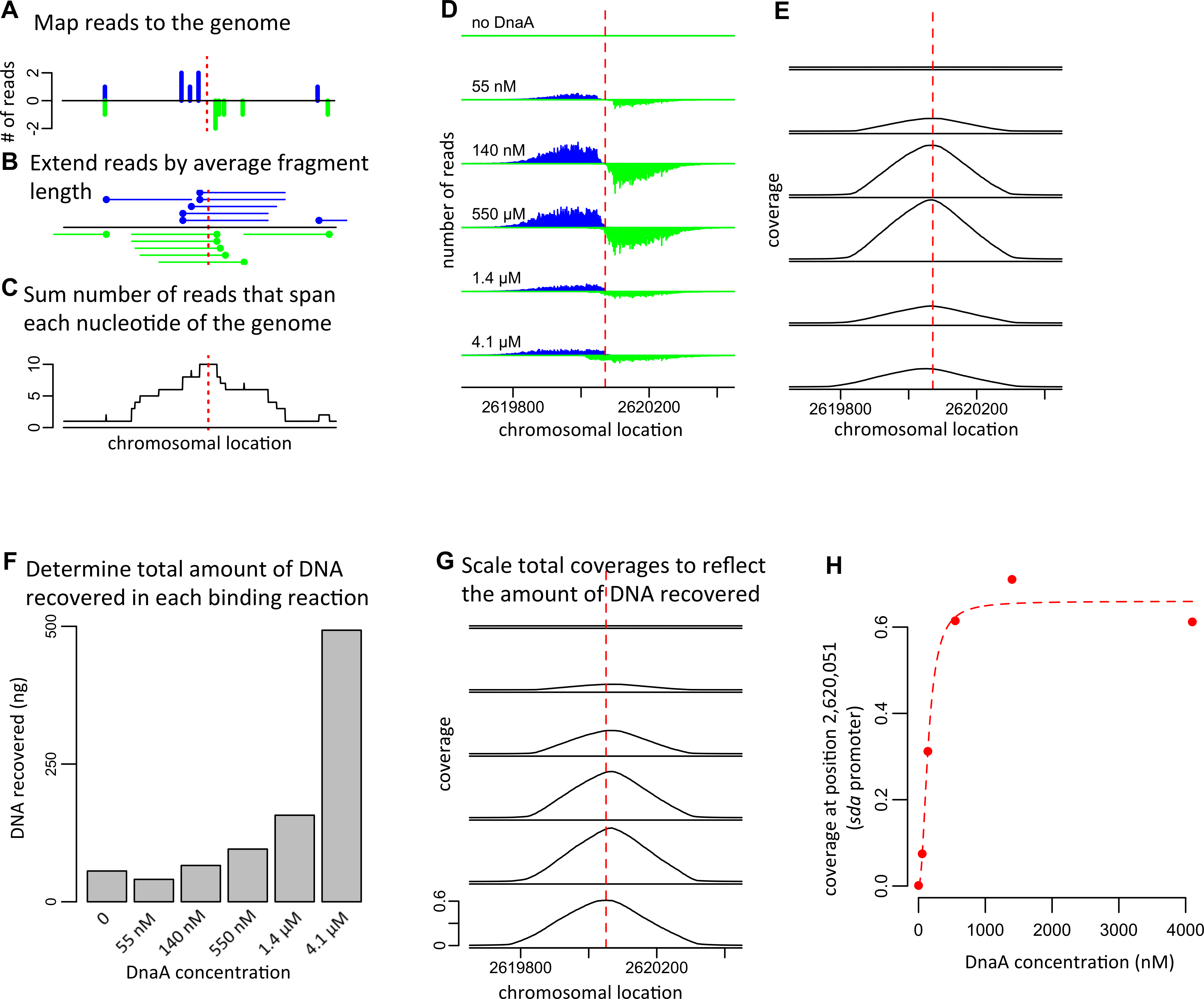

Supplement: S3 Fig — In panels A-C, a schematic representation using a toy dataset shows how deep sequencing data were converted to coverage along the chromosome. (A) start positions of sequence reads are plotted as histograms, and are shown clustered around a DnaA binding site depicted by the red dotted line. (B) Each read was extended in the appropriate direction (rightward for reads corresponding to the top strand, and leftward for reads corresponding to the bottom strand) by the average fragment length of 250 bp. (C) The number of fragments containing each nucleotide along the genome is determined, yielding the relative coverage along the genome. Although this allows for comparison between different genomic loci in the same binding reaction, it does not support comparison between different binding reactions (i.e., comparing ATP and ADP, or comparing different concentrations of DnaA-his.) (D) Actual sequence data from the sda promoter region from samples containing the indicated concentrations of ATP-DnaA-his. The y-axis scale for each of the samples is the same. The same total number of reads was mapped for each binding reaction, but the number of reads mapping to the sda promoter region (and other high-affinity DnaA binding regions) decreased at the two highest concentrations of DnaA-his. This is because at these DnaA concentrations, binding to sda has already saturated, while an increasing portion of the reads map to weaker binding regions, and there is also an increase in background binding. (E) The relative coverage along the same region as in D, obtained by extending the reads by the average read length and summing the number of extended reads spanning each position, as depicted in A, B, and C. (F) The amount of DNA recovered in each binding reaction (prior to any preparation steps for deep sequencing) was determined. (G) The coverage in panel E, which was calculated using the same number of reads for each sample, was scaled so that the total coverage (summed over the whole gen [file pgen.1005258.s003.tiff]

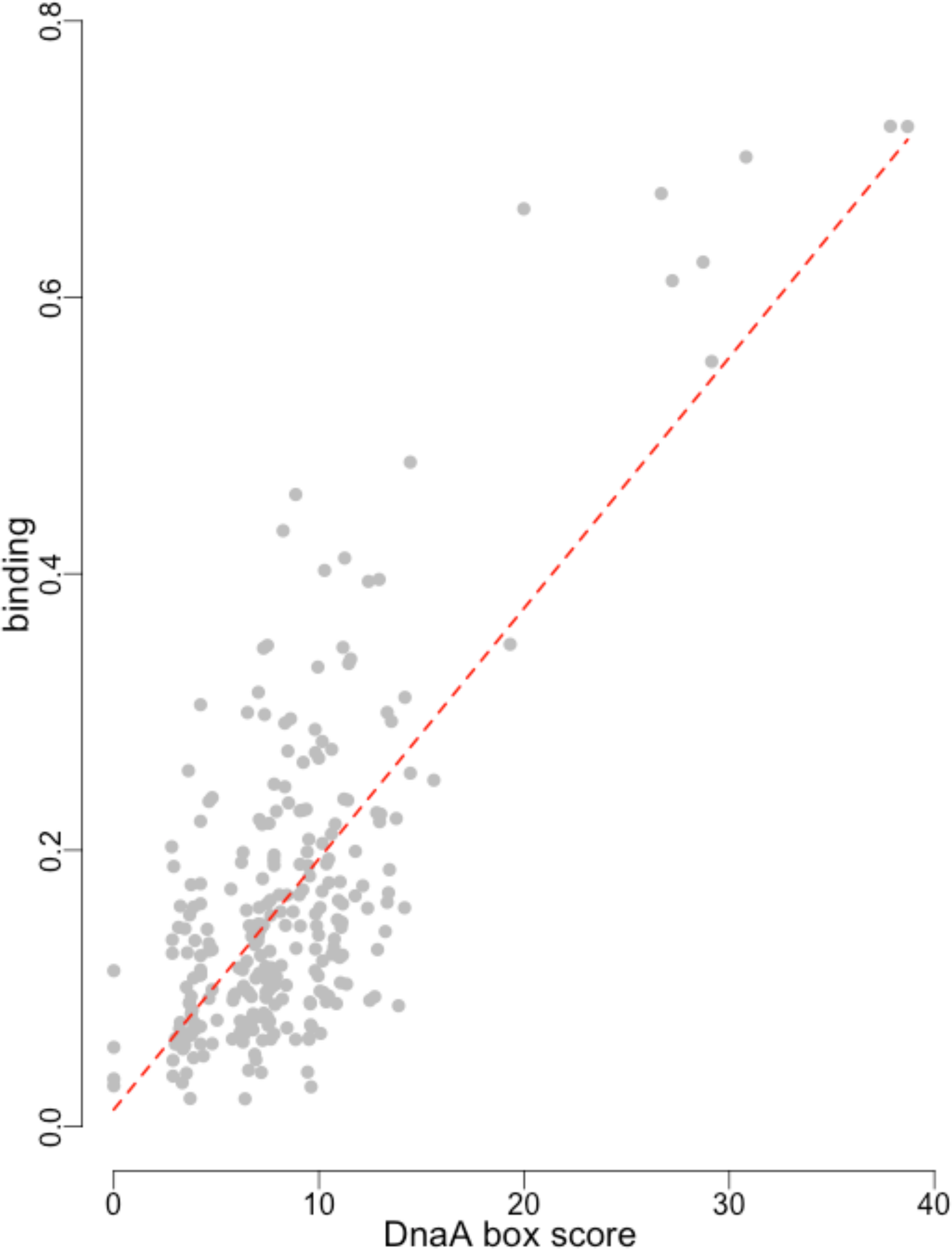

Supplement: S4 Fig — The amount of binding observed at each binding site using 4.1 μM ATP-DnaA-his is plotted as a function of the predicted strength of the DnaA box based on the PSSM. The DnaA box score for each binding region was calculated by summing the negative logs of the p-values from the PSSM for each predicted DnaA box in a 200 bp window centered on the peak summit. The line shown is a linear least squares regression fit of the data. (TIFF) [file pgen.1005258.s004.tiff]

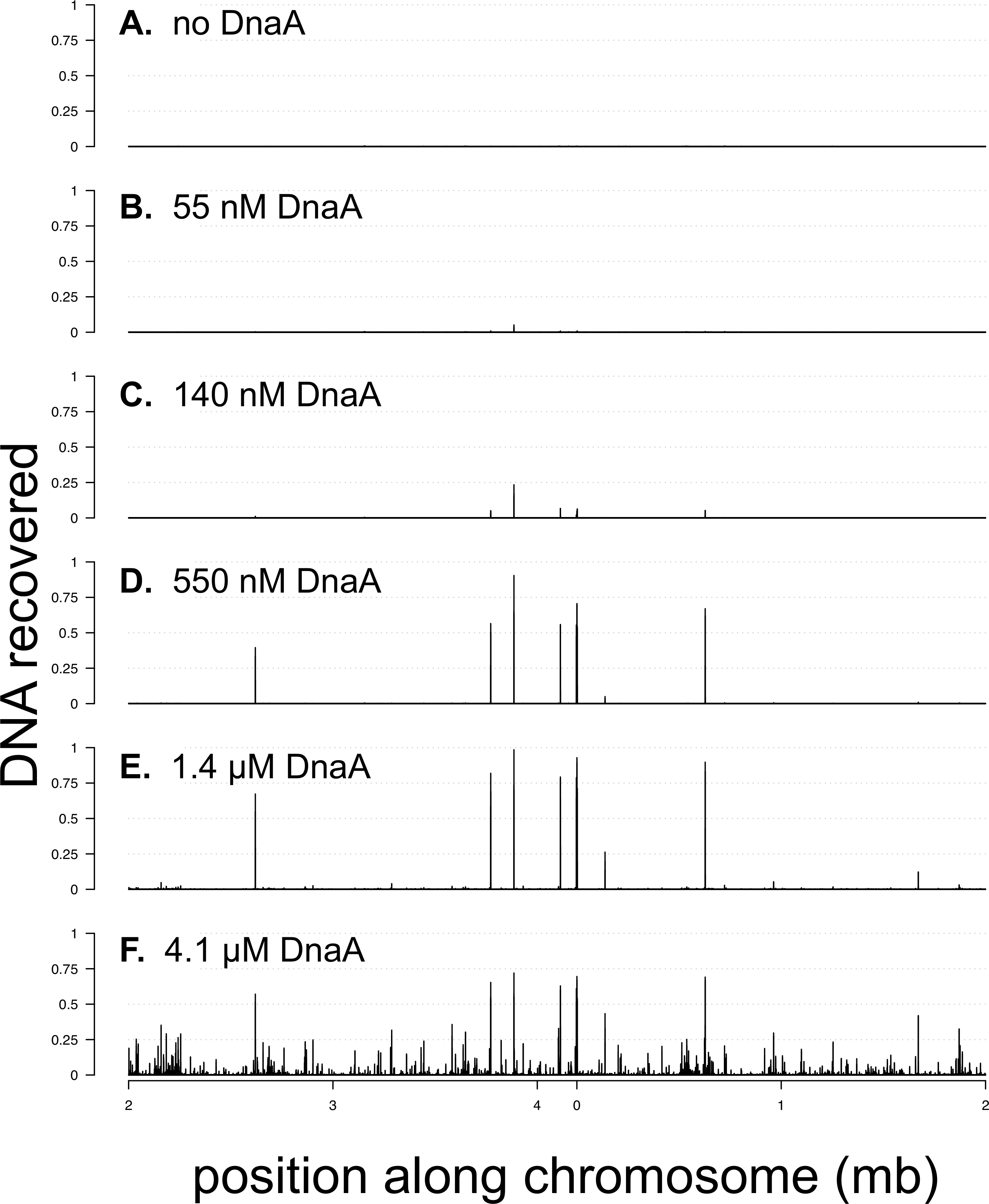

Supplement: S5 Fig — The relative amount of binding by ADP-DnaA-his is plotted on the y-axis versus the position along the chromosome on the x-axis. The 4.2 mb circular chromosome is depicted linearly such that the origin of replication is near the middle of the x-axis at 4.2 mb and 0 mb. The concentration of ADP-DnaA-his in each binding reaction was (A) no DnaA; (B) 55 nM; (C) 140 nM; (D) 550 nM; (E) 1.4 μM; (F) 4.1 μM. The binding profiles along the chromosome were determined by deep sequencing the DNA recovered in each binding reaction. Binding data are presented in 200 nt bins, with the maximum binding amplitude in each bin drawn. The amplitudes of each binding reaction were adjusted so that the total amount of binding is proportional to the amount of DNA recovered in that binding reaction (see S2 Fig for details.) The data were normalized together with binding data using ATP-DnaA-his (Fig 1) so that maximum binding had an amplitude of 1. (TIFF) [file pgen.1005258.s005.tiff]

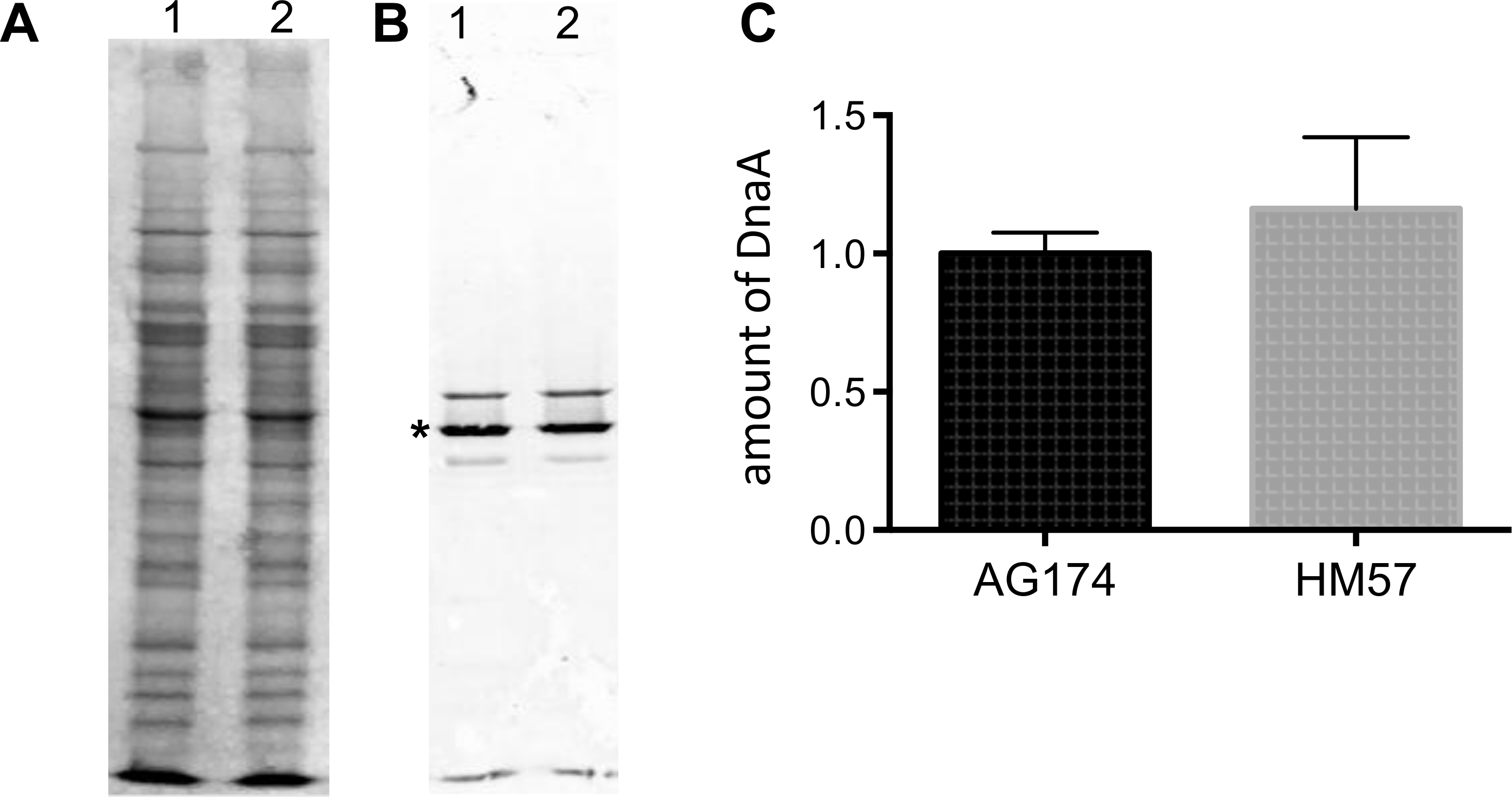

Supplement: S6 Fig — Whole cell lysates from wild type (rok+; AG174) and rok null mutant (rok-; HM57) cells grown to mid-exponential phase in LB medium were subjected to SDS polyacrylamide gel electrophoresis, and duplicate gels were analyzed by (A) Coomassie staining, and (B) western blotting with a DnaA antibody. Lane 1, AG174 (wild type), Lane 2, HM57 (rok null mutant). The position of the DnaA band is indicated with an asterisk. Quantitation of total Coomassie staining and DnaA levels was performed using near-infrared detection on an Odyssey imager (Licor). (C) The amount of DnaA relative to total protein was calculated, and normalized to a value of 1 for wild type. The mean of four replicates is presented, with error bars to indicate the standard deviation. The observed 1.16X difference in the means is not statistically significant (P = 0.28). (TIFF) [file pgen.1005258.s006.tiff]
