# Supplementary material for: In Vitro Whole Genome DNA Binding Analysis of the Bacterial Replication Initiator and Transcription Factor DnaA
Source: PLoS Genet. 2015 May 28;11(5):e1005258. doi: 10.1371/journal.pgen.1005258 (PMC4447404; doi:10.1371/journal.pgen.1005258)
Supplement: S1 Table — (PDF) [file pgen.1005258.s007.pdf]

peak number[1]	nearby gene(s)[2]	peak summit[3]	number of DnaA boxes[4]	peak amplitude at 1.4 µM DnaA[5]	peak amplitude at 4.1 µM DnaA[5]	K_d (µM) (ATP-DnaA-His)[6]	K_d (µM) (ADP-DnaA-His)[6]
1	upstream of rpmH; upstream of dnaA	150	12	0.92	0.70	0.21	0.33
2	downstream of dnaA; upstream of dnaN	1841	8	0.59	0.55	0.16	0.30
3	upstream of ywcI; upstream of vpr	3885674	11	1.00	0.72	0.13	0.17
4	downstream of gcp; downstream of ydiF	627955	10	0.96	0.72	0.15	0.33
5	upstream of trmE; downstream of jag	4191071	4	0.96	0.66	0.33	0.43
6	upstream of ywlC; downstream of ywlB	3772105	9	0.85	0.67	0.25	0.45
7	upstream of yqeG; upstream of sda	2620051	8	0.71	0.61	0.15	0.51
8	upstream of yydA; downstream of yyzF	4113012	7	0.64	0.62	0.16	0.33
9	inside codV	1670814	4	0.34	0.48	1.1	1.9
10	inside rplB	137727	2	0.28	0.46	1.4	1.2
11	downstream of ynaI; upstream of xynP	1870622	3	0.12	0.39	2.0	3.0
12	inside yhcA	962763	3	0.11	0.35	3.6	2.8
13	inside yorF	2158839	3	0.11	0.39	2.0	3.0
14	upstream of yvmC; upstream of yvmB	3582878	2	0.084	0.43	2.2	4.4
15	inside yhcN	972751	3	0.083	0.20	19	6.9
16	inside yutJ	3287547	5	0.074	0.35	2.5	2.9
17	inside yydH	4104262	3	0.073	0.41	2.4	4.6
18	inside ggaA	3648588	4	0.068	0.40	2.7	4.8
19	inside yopH	2184371	2	0.063	0.35	4.9	5.0
20	inside rlmCD	722497	3	0.060	0.23	7.5	6.7
21	upstream of xylR; upstream of xylA	1875249	4	0.055	0.29	5.5	5.8
22	inside pheT	2901978	2	0.051	0.27	3.1	5.7
23	inside yokD	2254843	3	0.047	0.33	4.8	5.0
24	upstream of yjdB; upstream of manR	1253820	5	0.047	0.31	3.5	5.6
25	inside yozL	2037328	5	0.046	0.30	5.2	5.2
26	inside sunT	2242095	4	0.046	0.33	3.0	5.2
27	inside leuB	2864218	2	0.042	0.27	3.9	5.6
28	upstream of yqbD; downstream of yqbC	2657896	4	0.041	0.29	3.3	5.5
29	upstream of atpI; downstream of upp	3766390	1	0.038	0.26	5.9	6.1
30	upstream of tuaA_pseudo; downstream of lytC	3637063	3	0.037	0.29	5.3	5.8
31	inside yddR	537736	4	0.036	0.31	5.0	5.4
32	inside dltA	3931019	4	0.034	0.25	5.9	6.1
33	inside sdpI	3444830	4	0.033	0.34		
34	inside yomG	2231138	4	0.032	0.30	5.0	5.6
35	inside yydI	4103032	2	0.031	0.35		
36	inside yobM	2046975	4	0.030	0.28	5.3	5.6
37	inside int	513492	2	0.027	0.30		
38	inside yhdF	1006129	4	0.026	0.18	7.8	6.9
39	upstream of yoaG; upstream of BSUB_01999	2001384	2	0.024	0.25	5.7	6.2
40	inside thiL	624773	2	0.023	0.29		
41	inside mtbP	2144181	3	0.023	0.27		
42	inside yusY_pseudo	3360154	2	0.021	0.19	6.9	6.7
43	inside guaC	3282045	3	0.021	0.24		
44	inside yyaB	4185827	4	0.021	0.25	5.5	5.9
45	inside vpr	3887160	2	0.020	0.21	6.4	6.0
46	upstream of yrkD; downstream of yrkC	2687367	3	0.020	0.27		
47	inside yobH_pseudo	2036906	4	0.019	0.22		
48	inside yezG	730934	1	0.019	0.24		
49	inside yhfW	1098759	4	0.018	0.22		
50	downstream of ylaN; upstream of ftsW	1536262	3	0.018	0.20	6.4	7.2
51	inside pckA	3107604	3	0.018	0.24		
52	inside skfC	216239	3	0.018	0.21		
53	inside yrrT	2760639	3	0.017	0.26		
54	downstream of yoaM; downstream of yozS	2009825	2	0.017	0.15		
55	inside ywhK	3822988	2	0.017	0.30	4.9	5.2
56	inside yobI	2040611	3	0.017	0.23		

peak number[1]	nearby gene(s)[2]	peak summit[3]	number of DnaA boxes[4]	peak amplitude at 1.4 µM DnaA[5]	peak amplitude at 4.1 µM DnaA[5]	K_d (µM) (ATP-DnaA-His)[6]	K_d (µM) (ADP-DnaA-His)[6]
57	upstream of ysoA; downstream of leuD	2861591	3	0.016	0.16		
58	inside yydB	4112070	2	0.016	0.22		
59	inside rnr	3431116	2	0.015	0.23		
60	inside yoqI	2170853	3	0.015	0.20		
61	inside yfhP	918710	4	0.014	0.22		
62	inside maeN	3222860	2	0.014	0.22		
63	inside yclE	416076	3	0.014	0.23		
64	downstream of glmS; upstream of BSUB_00228	202088	2	0.014	0.24		
65	inside yoqG	2171732	3	0.014	0.19		
66	inside ydiR	643948	4	0.014	0.23		
67	inside ggaB	3645388	2	0.014	0.17		
68	inside yeaA	666717	4	0.013	0.16		
69	upstream of yobI; downstream of yobJ	2042455	4	0.013	0.17		
70	downstream of yomP; upstream of yomO	2216984	2	0.013	0.20		
71	inside thyA	1886564	4	0.013	0.23		
72	inside ydeD	546380	4	0.013	0.22	5.7	6.3
73	upstream of metE; downstream of ispA	1369518	4	0.013	0.17		
74	inside yomH	2229315	4	0.012	0.23		
75	inside comP	3233626	4	0.012	0.23		
76	inside yomY	2211150	3	0.011	0.20		
77	upstream of ilvB; upstream of ysnD	2870093	2	0.011	0.22		
78	downstream of pksC; upstream of pksD	1768569	4	0.011	0.14		
79	downstream of yjcO; upstream of yjcP	1250077	2	0.011	0.14		
80	inside yeeB	727961	2	0.011	0.19		
81	inside ydjA	646424	4	0.011	0.19		
82	upstream of yptA; upstream of ypzG	2302570	3	0.010	0.18		
83	inside ymaB	1856243	2	0.010	0.16		
84	inside yolD	2244449	5	0.010	0.16		
85	upstream of yoeA; upstream of iseA	1975298	3	0.010	0.14		
86	inside ywcD	3900266	3	0.010	0.15		
87	inside yrkK	2681881	3	0.010	0.19		
88	inside gutB	651061	4	0.0098	0.19		
89	inside yddG	526341	4	0.0098	0.19		
90	inside yybL	4152785	3	0.0097	0.15		
91	upstream of arsR; downstream of yqcI	2630717	3	0.0096	0.14		
92	downstream of ybzH; upstream of ybcL	211810	2	0.0095	0.17		
93	downstream of cspC; downstream of ydeB	543306	2	0.0095	0.16		
94	inside putP	347627	3	0.0094	0.19		
95	inside Tn917_MLS_leader	2946908	4	0.0093	0.14		
96	upstream of ydjJ; upstream of iolT	659785	1	0.0093	0.17		
97	inside yddN	533833	3	0.0091	0.16		
98	inside ydfS	585223	4	0.0089	0.18		
99	upstream of tig; downstream of ysoA	2860502	2	0.0088	0.14		
100	inside lytD	3664577	1	0.0086	0.13		
101	upstream of speA; upstream of yktA	1519330	2	0.0086	0.16		
102	upstream of cccA; downstream of sigA	2572767	1	0.0086	0.13		
103	inside tagB	3661157	2	0.0085	0.16		
104	inside hemAT	1096402	3	0.0084	0.12		
105	inside BSUB_03753	3599666	3	0.0084	0.20		
106	inside ylqC	1657468	3	0.0083	0.17		
107	downstream of ynaC; upstream of ynaD	1866593	4	0.0081	0.18		
108	downstream of yrpC; upstream of yrpD	2711970	1	0.0081	0.16		
109	inside yydC	4110647	4	0.0081	0.15		
110	inside yunB	3300980	4	0.0079	0.088		
111	inside yxiD	4015025	1	0.0079	0.15		
112	downstream of ybxB; upstream of rpoB	121720	1	0.0079	0.14		

peak number[1]	nearby gene(s)[2]	peak summit[3]	number of DnaA boxes[4]	peak amplitude at 1.4 µM DnaA[5]	peak amplitude at 4.1 µM DnaA[5]	K_d (µM) (ATP-DnaA-His)[6]	K_d (µM) (ADP-DnaA-His)[6]
113	inside tagA	3659423	5	0.0078	0.13		
114	inside comEC	2611312	4	0.0076	0.16		
115	inside tagC	3662155	6	0.0075	0.17		
116	inside sdpB	3442867	2	0.0075	0.14		
117	inside yotB	2128318	3	0.0074	0.16		
118	upstream of ydeE; upstream of ydeF	548120	2	0.0074	0.13		
119	inside yacL	108737	4	0.0073	0.17		
120	downstream of yqgB; downstream of yqgA	2560642	3	0.0072	0.11		
121	inside yopK	2182576	1	0.0072	0.063		
122	upstream of ydhU_pseudo; upstream of trnE-Arg	618522	3	0.0071	0.12		
123	inside yonH	2202195	2	0.0070	0.12		
124	inside ypmA	2322241	2	0.0070	0.081		
125	inside yyaP	4165129	2	0.0070	0.14		
126	inside ywfH	3845978	4	0.0069	0.15		
127	upstream of ynfC; upstream of alsT	1922157	3	0.0069	0.15		
128	upstream of czcD; downstream of yrdN	2697762	4	0.0069	0.17		
129	upstream of yonR; upstream of yonP	2194309	3	0.0069	0.14		
130	inside xkdO	1320733	4	0.0068	0.16		
131	upstream of yrdB; downstream of yrdA	2707146	1	0.0068	0.036	32	29
132	downstream of malR; upstream of nupN	3217564	2	0.0068	0.13		
133	upstream of slp; upstream of BSUB_01591	1517608	1	0.0067	0.10		
134	inside yhdP	1016193	3	0.0066	0.13		
135	inside ppsB	1956283	3	0.0065	0.089		
136	inside arsB	2629498	3	0.0064	0.18		
137	downstream of groEL; upstream of ydiM	635877	2	0.0064	0.093		
138	inside yfmI	803823	2	0.0064	0.095		
139	inside ggaA	3649182	4	0.0063	0.14		
140	inside ygaN	949113	2	0.0061	0.15		
141	upstream of yubD; upstream of cdoA	3171744	2	0.0061	0.098		
142	upstream of yvbW; downstream of yvbX	3469672	2	0.0061	0.096		
143	inside glcT	1439894	2	0.0060	0.10		
144	upstream of azlB; downstream of yrdF	2703111	2	0.0060	0.11		
145	upstream of mmsA; upstream of iolR	4062626	3	0.0060	0.10		
146	inside yomD	2235340	5	0.0058	0.13		
147	inside ansB	2428074	4	0.0057	0.14		
148	inside yoaU	2018593	2	0.0057	0.097		
149	inside ntdB	1111160	2	0.0057	0.14		
150	inside yrkN	2680299	2	0.0056	0.12		
151	inside yrkA	2692074	1	0.0056	0.11		
152	downstream of sbp; upstream of ftsA	1579864	1	0.0056	0.070		
153	inside yddM	532774	2	0.0056	0.047		
154	inside yomO	2217320	4	0.0055	0.13		
155	inside yeeA	725278	2	0.0055	0.11		
156	inside ytnJ	2980594	1	0.0054	0.072		
157	inside queE	1424707	3	0.0054	0.10		
158	upstream of yndM; upstream of fosB	1900185	3	0.0054	0.096		
159	upstream of yxxF; downstream of yxiE	4009775	3	0.0054	0.11		
160	inside putR	349481	3	0.0054	0.066		
161	inside yonO	2195486	1	0.0054	0.092		
162	inside yydD	4109616	2	0.0054	0.030		
163	downstream of yddS; upstream of BSUB_00561	539638	1	0.0053	0.12		
164	upstream of rrnB-16S; upstream of thiT	3156879	2	0.0053	0.079		
165	upstream of ywnA; downstream of ureC	3744658	3	0.0052	0.078		
166	downstream of exuR; upstream of uxaB	1293427	2	0.0052	0.15		
167	upstream of yrzI; downstream of yrhG	2752096	5	0.0052	0.093		
168	inside yoyD	2103058	4	0.0052	0.13		

peak number[1]	nearby gene(s)[2]	peak summit[3]	number of DnaA boxes[4]	peak amplitude at 1.4 µM DnaA[5]	peak amplitude at 4.1 µM DnaA[5]	K_d (µM) (ATP-DnaA-His)[6]	K_d (µM) (ADP-DnaA-His)[6]
169	inside bpr	1585389	2	0.0052	0.060		
170	upstream of yugI; downstream of yugH	3203712	2	0.0052	0.14		
171	inside yvgL	3403906	3	0.0052	0.14		
172	downstream of ykuV; upstream of rok	1477265	3	0.0052	0.067		
173	inside yybK	4153513	2	0.0052	0.072		
174	inside ywqJ	3703705	2	0.0051	0.17		
175	upstream of qoxA; upstream of ywzA	3896457	2	0.0051	0.059		
176	inside mcpC	1449127	2	0.0050	0.076		
177	upstream of yhcQ; upstream of yhcR	974951	2	0.0050	0.075		
178	downstream of amyE; upstream of ldh	329762	5	0.0050	0.093		
179	inside coaD	1553779	2	0.0049	0.12		
180	inside speE	3827051	3	0.0049	0.11		
181	inside nrdEB	2136036	5	0.0049	0.11		
182	inside aldY	3965319	3	0.0048	0.12		
183	upstream of trpE; downstream of aroH	2350371	1	0.0048	0.11		
184	inside kinC	1502031	3	0.0048	0.097		
185	inside yxeI	4041561	3	0.0048	0.12		
186	inside ylaJ	1532826	3	0.0048	0.098		
187	inside yosU	2131253	3	0.0048	0.088		
188	upstream of bdhA; upstream of ydjM	662926	3	0.0048	0.069		
189	upstream of yvaK; downstream of secG	3433048	2	0.0047	0.050		
190	inside cwlP	2225901	2	0.0047	0.11		
191	upstream of trxB; downstream of cwlO	3552343	3	0.0047	0.12		
192	inside sunS	2238728	1	0.0047	0.065		
193	upstream of katA; upstream of ssuB	944689	1	0.0047	0.068		
194	inside paiB	3282447	1	0.0046	0.13		
195	inside uxaB	1293996	2	0.0046	0.16		
196	inside yokG	2252170	3	0.0046	0.10		
197	inside xkdV	1326957	4	0.0046	0.10		
198	inside cotH	3694449	3	0.0046	0.17		
199	inside yvrG	3386160	2	0.0046	0.10		
200	inside yxxG	4001361	3	0.0046	0.089		
201	upstream of yonX; downstream of yonV	2190425	2	0.0045	0.074		
202	upstream of cotU; upstream of thyA	1885553	3	0.0045	0.11		
203	upstream of ppsA; downstream of dacC	1970880	2	0.0045	0.034		
204	inside yqfL	2576950	3	0.0045	0.096		
205	inside immA	514275	2	0.0045	0.069		
206	downstream of yjcS; upstream of yjdA	1252152	2	0.0045	0.083		
207	inside asnB	3104302	2	0.0044	0.071		
208	inside sunT	2241620	2	0.0044	0.065		
209	upstream of ykvA; upstream of spo0E	1414175	2	0.0044	0.070		
210	upstream of dinB; upstream of ydgG	592390	5	0.0044	0.13		
211	upstream of yorM; downstream of yorL	2148355	3	0.0043	0.079		
212	inside yxkI	3959970	2	0.0043	0.076		
213	upstream of artP; downstream of yqiW	2465792	3	0.0043	0.093		
214	inside yonO	2197769	2	0.0043	0.11		
215	upstream of immR; upstream of xis	515238	3	0.0043	0.028		
216	inside yrdR	2693590	3	0.0042	0.073		
217	upstream of cydA; upstream of cimH	3957253	3	0.0042	0.063		
218	inside yjcM	1248087	4	0.0042	0.062		
219	inside nrdE	1854429	2	0.0041	0.14		
220	upstream of ackA; downstream of ytxK	2994356	4	0.0041	0.072		
221	inside BSUB_01899	1884325	2	0.0041	0.075		
222	downstream of fadH; upstream of fadG	1462449	2	0.0041	0.090		
223	inside yoyI	2185822	2	0.0041	0.056		
224	upstream of yokH; downstream of yokG	2250928	2	0.0041	0.065		

peak number[1]	nearby gene(s)[2]	peak summit[3]	number of DnaA boxes[4]	peak amplitude at 1.4 μM DnaA[5]	peak amplitude at 4.1 μM DnaA[5]	K_d (μM) (ATP-DnaA-His)[6]	K_d (μM) (ADP-DnaA-His)[6]
225	downstream of appC; upstream of yjbA	1200835	3	0.0041	0.11		
226	inside ykuH	1465117	3	0.0041	0.040		
227	downstream of frr; upstream of uppS	1704653	2	0.0041	0.051		
228	upstream of sdpR; downstream of opuCD	3445492	1	0.0041	0.028		
229	inside xkdK	1316997	3	0.0040	0.090		
230	inside ygzC	938286	1	0.0040	0.093		
231	inside BSUB_03076	2926317	3	0.0040	0.13		
232	inside gltR	2698613	1	0.0040	0.059		
233	downstream of ykvO; upstream of ykvP	1427359	2	0.0040	0.098		
234	downstream of bofA; upstream of rrnA-16S	30230	1	0.0039	0.058		
235	inside ypfD	2367960	4	0.0039	0.13		
236	inside yomR	2215348	1	0.0039	0.075		
237	inside mtlA	451069	2	0.0039	0.11		
238	downstream of prkA; upstream of yhbH	958682	1	0.0039	0.038		
239	inside yorJ	2154951	2	0.0039	0.11		
240	upstream of trpS; upstream of oppA	1203242	2	0.0039	0.019		
241	upstream of sunA; downstream of sunI	2242579	1	0.0039	0.019		
242	inside yqaP	2664910	2	0.0038	0.066		
243	inside yqxI	2637135	1	0.0038	0.047		
244	inside yfmG	805867	1	0.0038	0.059		
245	inside pksR	1840366	3	0.0037	0.066		
246	inside spoIVFA	2829713	3	0.0037	0.062		
247	inside menE	3126505	4	0.0037	0.086		
248	downstream of ffh; upstream of rpsP	1657118	3	0.0037	0.038		
249	inside yotJ	2126323	1	0.0037	0.088		
250	inside ydiO	639141	3	0.0037	0.11		
251	downstream of rsbX; upstream of ydcF	507944	1	0.0037	0.12		
252	inside bceB	3087927	3	0.0037	0.088		
253	upstream of yyaR; upstream of yyaQ	4163988	5	0.0037	0.11		
254	inside ugtP	2279297	2	0.0037	0.081		
255	inside uvrX	2244022	2	0.0036	0.076		
256	upstream of odhA; downstream of yojO	2084477	2	0.0036	0.061		
257	inside yqaP	2664545	3	0.0036	0.071		
258	upstream of yocI; downstream of yocJ	2068958	3	0.0036	0.055		
259	inside yopA	2189076	4	0.0036	0.062		
260	inside ywqM	3701693	2	0.0036	0.11		
261	inside nasF	353939	1	0.0035	0.11		
262	inside araA	2921372	2	0.0034	0.091		
263	inside mtbP	2145278	1	0.0033	0.081		
264	inside ydcI	511301	2	0.0032	0.092		
265	inside ybfB	237087	2	0.0032	0.087		
266	inside yvqJ	3377365	2	0.0031	0.062		
267	upstream of ctaA; upstream of ctaB	1542613	3	0.0030	0.038		
268	inside yokF	2252962	2	0.0030	0.097		
269	inside BSUB_01910	1890933	1	0.0030	0.049		
