# Supplementary material for: In Vitro Whole Genome DNA Binding Analysis of the Bacterial Replication Initiator and Transcription Factor DnaA
Source: PLoS Genet. 2015 May 28;11(5):e1005258. doi: 10.1371/journal.pgen.1005258 (PMC4447404; doi:10.1371/journal.pgen.1005258)
Supplement: S2 Table — The 5' and 3' nucleotide position (AG1839 genome coordinates), DNA strand (+/-), and sequence of each of the 150 DnaA boxes is shown. (PDF) [file pgen.1005258.s008.pdf]

Table S2. DnaA boxes used to determine the PSSM.

5'	3'	+/-	sequence
1006105	1006113	+	TTGTGAACA
1006129	1006137	–	TGGTCCACT
1012441	1012449	+	TTATACACG
1012588	1012596	+	TTATTCACT
1014428	1014436	+	TTATGCAGA
1014544	1014552	–	TGATTCACA
1014952	1014960	+	TTTTGTGCA
1015050	1015058	+	GTGTACACA
1016150	1016158	–	TTATTAACA
1016185	1016193	+	TCTTCCACA
1016326	1016334	+	TTATTCACA
1022553	1022561	+	TTATTCACA
1040122	1040130	+	CTATCAACA
1040211	1040219	+	CTATACACA
1070690	1070698	+	ATATTCACA
1070872	1070880	+	TTATACACA
1081420	1081428	+	GTATCAACA
1088429	1088437	–	TTATCAACA
1088569	1088577	+	TTATTAAGA
1088702	1088710	–	TTATCGACA
1088821	1088829	+	TCTTCCACA
1096308	1096316	–	TCGTCCACA
1096404	1096412	–	AAATCAACA
1096424	1096432	+	TTGTTCACA
1096790	1096798	+	TTGTCCACA
1104517	1104525	–	CAATTCACA
1104614	1104622	–	TTATTAACA
1109808	1109816	–	ATATCAACA
1109816	1109824	–	TTATCCATA
1111165	1111173	+	CTATCCCCA
1111250	1111258	–	TTATCCCCA
1111821	1111829	–	TTATCCACG
1111853	1111861	+	TTAATAACA
1112642	1112650	–	TAACCCACA
1112824	1112832	–	TTATCTACA
1113232	1113240	+	ATGTCAACA
1116247	1116255	–	ATATGCACA
1116422	1116430	+	ATATCCACA
1121963	1121971	–	TTGTTAACA
1122015	1122023	–	TTATTCACA
1139967	1139975	–	GTATCCACA
1140009	1140017	–	TTTTCAACA
1140076	1140084	–	ATGTACACA
1142254	1142262	–	TATTCCACA
1142122	1142130	–	TTATCCACA
1154518	1154526	–	ATATCCCCA
1155386	1155394	+	GTGTACACA

5'	3'	+/-	sequence
1155488	1155496	–	TTATCCACT
1164978	1164986	–	TAATCAACA
1165048	1165056	–	TTGTTAACA
1165202	1165210	–	ATATCCTCA
1166036	1166044	+	CGATCCACA
1166144	1166152	+	GTATCAACA
1166154	1166162	–	TTGTCAACA
1166261	1166269	+	TCATCCACT
1175748	1175756	+	TTATTAACA
1175701	1175709	–	TTTTTAACA
1190177	1190185	+	TTGATCACA
1190228	1190236	–	TTGTCCACA
1190353	1190361	–	ACGTCCACA
1198413	1198421	+	TTATCCACA
1200206	1200214	–	TGATCAACA
1200240	1200248	–	TTGTCCACA
1200695	1200703	+	ATATCACCA
1200837	1200845	–	TTGTCCACA
1200893	1200901	–	TGACCCACA
1211254	1211262	+	CTATACACA
1212525	1212533	–	CCGTCCACA
1214737	1214745	+	ATATTCACA
1214885	1214893	–	ATATCCTCA
1218389	1218397	+	GTATCAACA
1218456	1218464	–	TAGTCAACA
1225637	1225645	–	CTGTCCACA
1225771	1225779	–	ATATTAACA
1226767	1226775	+	TTATTCACA
1226954	1226962	+	ATTTCCACA
1227479	1227487	+	CTATGCACA
1227537	1227545	–	ATATCCACA
1233829	1233837	+	TCATCCACA
1233858	1233866	–	TTCTCCACA
1233994	1234002	+	TTTTCCACT
1235164	1235172	–	CTTTTCACA
1235283	1235291	–	TTATCAACA
1236886	1236894	–	GAATGCACA
1236932	1236940	–	TTATCCATA
1237052	1237060	–	TTAATCACA
1248015	1248023	–	CAATCAACA
1248094	1248102	+	TTTTCAACA
1248208	1248216	+	TTGTCCACT
1252064	1252072	+	AAAAACACA
1252128	1252136	–	TCATTCACA
1252186	1252194	–	TTAGTCACA
1253817	1253825	–	CTATTAACA
1253706	1253714	–	TTATTAACA

5'	3'	+/-	sequence
1259479	1259487	–	TTGTCCACA
1259554	1259562	–	TTATGAACA
1275031	1275039	–	CGCTCCACA
1275071	1275079	–	TTATCCCCA
1281791	1281799	–	TTATTGACA
1282049	1282057	–	TCATGCACA
1293398	1293406	–	TTATGCACA
1293448	1293456	–	TCGTACACA
1293935	1293943	–	CAGTTCACA
1293985	1293993	+	TTATGCACA
1294015	1294023	+	ATTTACACA
1304924	1304932	–	TTATCAACT
1304958	1304966	–	TTTTCCCCA
1305042	1305050	–	TTATTAACG
1316949	1316957	+	TTATTCACA
1317074	1317082	–	TTATTCACA
1320714	1320722	+	TAATACACA
1320735	1320743	+	TTACTCACC
1321378	1321386	–	TTGCTCACA
1321465	1321473	–	GTACTCACA
1321477	1321485	–	TTGTCAACA
1326884	1326892	+	CTGCCCACA
1326948	1326956	–	TTATCCACA
1327388	1327396	+	TTGTAAACA
1327496	1327504	–	CAATCCACA
1342829	1342837	+	TTATCCATC
1342949	1342957	–	TTTTTCACA
1343063	1343071	–	GTTTCAACA
1369447	1369455	–	TTAATAACA

5'	3'	+/-	sequence
1369478	1369486	–	TTACCAACA
1369509	1369517	+	TTGTCAACA
1382276	1382284	+	TTATTGACA
1382343	1382351	+	TTACGCACA
1382480	1382488	+	GCATACACA
1392493	1392501	–	TCGTCCACA
1392577	1392585	+	TGAATAACA
1414148	1414156	–	TTGTAAACA
1414176	1414184	–	TTGTGCATT
1424617	1424625	–	TTGTGCACA
1424731	1424739	–	TTATCAACA
1427359	1427367	–	TTATTCACA
1427984	1427992	–	TTTTACACA
1428006	1428014	–	TTAATCCCC
1429148	1429156	–	TTCCCCACA
1429168	1429176	–	TCATCCCCA
1429338	1429346	–	TTAATCACA
1430349	1430357	+	TCATACACA
1430529	1430537	–	TTATCAACA
1431195	1431203	–	ATTTCCACA
1431334	1431342	+	TTTTCAACA
1431391	1431399	+	ATTTTCACA
1439870	1439878	–	TCATCAACA
1439950	1439958	+	ATATTCACA
1446614	1446622	+	TTATCAACA
1446692	1446700	+	ATATCCACT
1449177	1449185	–	AAATCCCCA
