# Supplementary material for: In Vitro Whole Genome DNA Binding Analysis of the Bacterial Replication Initiator and Transcription Factor DnaA
Source: PLoS Genet. 2015 May 28;11(5):e1005258. doi: 10.1371/journal.pgen.1005258 (PMC4447404; doi:10.1371/journal.pgen.1005258)
Supplement: S3 Table — Nucleotide frequencies for each position in the 150 DnaA boxes in S2 Table are presented. (PDF) [file pgen.1005258.s009.pdf]

Table S3. Nucleotide frequencies for the DnaA box PSSM.

position	A	C	G	T
1	23	16	10	101
2	13	15	7	115
3	96	3	33	18
4	8	9	1	132
5	16	78	13	43
6	45	100	3	2
7	138	9	1	2
8	0	144	2	4
9	135	3	3	9
