# Supplementary material for: In Vitro Whole Genome DNA Binding Analysis of the Bacterial Replication Initiator and Transcription Factor DnaA
Source: PLoS Genet. 2015 May 28;11(5):e1005258. doi: 10.1371/journal.pgen.1005258 (PMC4447404; doi:10.1371/journal.pgen.1005258)
Supplement: S4 Table — All DnaA boxes drawn in S1 Fig are presented. Peak numbers are the same as in S1 Table and correspond to the panel numbers in S1 Fig. Start coordinates correspond to the genome sequence of strain AG1839 [29]. P values are from RSAT [49]. The distance from the center nucleotide of the DnaA box to the peak summit (found in S1 Table) is shown. (PDF) [file pgen.1005258.s010.pdf]

| peak | sequence | p_value | start | strand | bp to peak |
|---|---|---|---|---|---|
| 1 | TTGTCAACT | 1.0E-03 | 4193493 | + | -297 |
| 1 | GTATCCACA | 7.7E-05 | 21 | + | -129 |
| 1 | TTATCGACA | 3.9E-04 | 34 | + | -116 |
| 1 | ATTTTCACA | 5.7E-04 | 45 | + | -105 |
| 1 | TTGTCCACA | 9.3E-06 | 65 | - | -85 |
| 1 | TTTTCAACA | 1.7E-04 | 78 | + | -72 |
| 1 | TTATCCACA | 5.3E-06 | 99 | - | -51 |
| 1 | TTATCCACA | 5.3E-06 | 193 | + | 43 |
| 1 | TTATCCACA | 5.3E-06 | 205 | - | 55 |
| 1 | CAATCCACA | 4.6E-04 | 216 | - | 66 |
| 1 | TTCTACACA | 1.4E-03 | 238 | - | 88 |
| 1 | TTGTCCACA | 9.3E-06 | 249 | + | 99 |
| 1 | TTTTCGACA | 1.5E-03 | 270 | - | 120 |
| 1 | TTTTCAACA | 1.7E-04 | 303 | + | 153 |
| 1 | TCGTACACA | 8.7E-04 | 317 | - | 167 |
| 2 | TTATTCACA | 1.6E-05 | 1777 | - | -60 |
| 2 | TCATACACA | 4.3E-04 | 1797 | + | -40 |
| 2 | CTGTCCACA | 1.1E-04 | 1808 | + | -29 |
| 2 | CTATCCACA | 3.2E-05 | 1817 | - | -20 |
| 2 | TTTTTCACA | 1.3E-04 | 1841 | + | 4 |
| 2 | TTATCCACA | 5.3E-06 | 1852 | + | 15 |
| 2 | AAATCCACA | 4.3E-04 | 1860 | + | 23 |
| 2 | CTTTCAACA | 8.1E-04 | 1968 | - | 131 |
| 3 | CCATCCACA | 3.4E-04 | 3885315 | + | -355 |
| 3 | CTTTCAACA | 8.1E-04 | 3885553 | - | -117 |
| 3 | TTATACACA | 5.9E-05 | 3885605 | + | -65 |
| 3 | TTAATCACA | 4.3E-04 | 3885638 | + | -32 |
| 3 | ATATCCACA | 2.8E-05 | 3885651 | + | -19 |
| 3 | ATGTCCACA | 9.3E-05 | 3885659 | + | -11 |
| 3 | ATATTCCCA | 1.2E-03 | 3885697 | + | 27 |
| 3 | ATATACACA | 2.9E-04 | 3885711 | + | 41 |
| 3 | TTATTCACT | 4.3E-04 | 3885723 | + | 53 |
| 3 | TTATACACA | 5.9E-05 | 3885731 | + | 61 |
| 3 | TTATACACA | 5.9E-05 | 3885746 | - | 76 |
| 3 | TTATACACA | 5.9E-05 | 3885763 | + | 93 |
| 3 | TTATCCACA | 5.3E-06 | 3885932 | + | 262 |
| 4 | TTATTAACA | 7.3E-05 | 627872 | + | -79 |
| 4 | CAATTCACA | 1.0E-03 | 627883 | + | -68 |
| 4 | TTATCCACA | 5.3E-06 | 627894 | + | -57 |
| 4 | TTACTCACA | 2.9E-04 | 627907 | - | -44 |
| 4 | TTATTAACA | 7.3E-05 | 627952 | - | 1 |
| 4 | TTATCCACT | 1.7E-04 | 627961 | - | 10 |
| 4 | CTATCCACA | 3.2E-05 | 627983 | - | 32 |
| 4 | TTATACACA | 5.9E-05 | 627992 | - | 41 |
| 4 | TTATCCACA | 5.3E-06 | 628035 | - | 84 |
| 4 | CTATCCACA | 3.2E-05 | 628088 | - | 137 |

| peak | sequence | p_value | start | strand | bp to peak |
|---|---|---|---|---|---|
| 5 | ATTTCCACA | 2.3E-04 | 4190897 | - | -170 |
| 5 | TTATCCACA | 5.3E-06 | 4191023 | + | -44 |
| 5 | TTATCCACA | 5.3E-06 | 4191063 | + | -4 |
| 5 | TTATTCACA | 1.6E-05 | 4191072 | - | 5 |
| 5 | TTATCAACA | 2.3E-05 | 4191110 | + | 43 |
| 5 | TTATTCACG | 8.7E-04 | 4191326 | - | 259 |
| 6 | TTGTCCACA | 9.3E-06 | 3771832 | - | -269 |
| 6 | TGATCCACA | 1.3E-04 | 3771990 | - | -111 |
| 6 | TTATCCACA | 5.3E-06 | 3772001 | - | -100 |
| 6 | ACATCCACA | 2.6E-04 | 3772020 | + | -81 |
| 6 | TTATTCACA | 1.6E-05 | 3772064 | + | -37 |
| 6 | TTATCCACA | 5.3E-06 | 3772144 | + | 43 |
| 6 | TTGTCCACA | 9.3E-06 | 3772156 | - | 55 |
| 6 | TTATCCACT | 1.7E-04 | 3772177 | - | 76 |
| 6 | TTATACACA | 5.9E-05 | 3772189 | - | 88 |
| 6 | TTATCCAGA | 7.0E-04 | 3772205 | + | 104 |
| 6 | CGATTCACA | 1.4E-03 | 3772250 | + | 149 |
| 7 | TTTTTAACA | 4.3E-04 | 2619869 | + | -178 |
| 7 | TTTTCGACA | 1.5E-03 | 2619917 | - | -130 |
| 7 | TTATCCACA | 5.3E-06 | 2619995 | - | -52 |
| 7 | TTACACACA | 7.0E-04 | 2620006 | + | -41 |
| 7 | CTATCCACT | 8.1E-04 | 2620015 | + | -32 |
| 7 | TTATAAACA | 2.0E-04 | 2620049 | + | 2 |
| 7 | ATACTCACA | 1.1E-03 | 2620060 | + | 13 |
| 7 | TTATCCACT | 1.7E-04 | 2620071 | + | 24 |
| 7 | TTATCCACA | 5.3E-06 | 2620086 | - | 39 |
| 8 | ATATTCACA | 9.3E-05 | 4112941 | + | -67 |
| 8 | TCATCCCCA | 7.0E-04 | 4112952 | + | -56 |
| 8 | TTATTCATA | 1.0E-03 | 4112974 | + | -34 |
| 8 | TTGTCCACA | 9.3E-06 | 4112987 | + | -21 |
| 8 | ATATCCACA | 2.8E-05 | 4112998 | - | -10 |
| 8 | TAATTCACA | 2.1E-04 | 4113068 | + | 60 |
| 8 | TTATCCACA | 5.3E-06 | 4113080 | - | 72 |
| 8 | TTCTTCACA | 5.7E-04 | 4113170 | + | 162 |
| 8 | TTATTCACA | 1.6E-05 | 4113261 | + | 253 |
| 8 | TTATCCACA | 5.3E-06 | 4113295 | + | 287 |
| 8 | TCATCAACA | 1.9E-04 | 4113319 | - | 311 |
| 9 | TTCTTAACA | 1.5E-03 | 1670746 | - | -64 |
| 9 | TTATTCACA | 1.6E-05 | 1670797 | + | -13 |
| 9 | TAATTCACA | 2.1E-04 | 1670815 | - | 5 |
| 9 | GAATCCACA | 7.0E-04 | 1670824 | - | 14 |
| 10 | TTATCAACA | 2.3E-05 | 137705 | + | -18 |
| 10 | TTATGCACA | 5.9E-05 | 137730 | - | 7 |
| 10 | TTATCAACA | 2.3E-05 | 137915 | + | 192 |
| 10 | TCACCCACA | 7.0E-04 | 137997 | + | 274 |
| 11 | TTTTCAACA | 1.7E-04 | 1870544 | - | -74 |

| peak | sequence | p_value | start | strand | bp to peak |
|---|---|---|---|---|---|
| 11 | TGATCAACA | 4.3E-04 | 1870594 | + | -24 |
| 11 | TTATCCACA | 5.3E-06 | 1870624 | + | 6 |
| 12 | ATATACACA | 2.9E-04 | 962393 | + | -366 |
| 12 | TTATTCACA | 1.6E-05 | 962597 | + | -162 |
| 12 | TTATAAACA | 2.0E-04 | 962732 | + | -27 |
| 12 | TTATCCCCA | 1.3E-04 | 962764 | - | 5 |
| 12 | TTGCCCACA | 2.6E-04 | 962785 | - | 26 |
| 13 | TTTTCAACA | 1.7E-04 | 2158624 | + | -211 |
| 13 | TTGTACACA | 1.5E-04 | 2158684 | + | -151 |
| 13 | CTGTTCACA | 2.6E-04 | 2158819 | + | -16 |
| 13 | ATATCCACA | 2.8E-05 | 2158843 | - | 8 |
| 13 | TTATTCACA | 1.6E-05 | 2158867 | - | 32 |
| 14 | TGATCCCCA | 1.4E-03 | 3582497 | + | -377 |
| 14 | TTTTCCACA | 4.3E-05 | 3582862 | + | -12 |
| 14 | ATATCAACA | 1.3E-04 | 3582899 | + | 25 |
| 14 | TAATGAACA | 1.4E-03 | 3583087 | + | 213 |
| 15 | GAATTCACA | 1.5E-03 | 972562 | + | -185 |
| 15 | TTGACAACA | 1.0E-03 | 972742 | + | -5 |
| 15 | TCATTAACA | 4.6E-04 | 972752 | - | 5 |
| 15 | TTATTGACA | 8.1E-04 | 972764 | - | 17 |
| 15 | ATTTACACA | 1.4E-03 | 973097 | + | 350 |
| 16 | TTATCCTCA | 6.2E-04 | 3287219 | - | -324 |
| 16 | TTTTGCACA | 3.9E-04 | 3287471 | - | -72 |
| 16 | TTATTAACA | 7.3E-05 | 3287506 | + | -37 |
| 16 | TTATACACA | 5.9E-05 | 3287546 | - | 3 |
| 16 | CTGTCCACA | 1.1E-04 | 3287587 | - | 44 |
| 16 | TTCTCCACA | 2.6E-04 | 3287633 | - | 90 |
| 17 | TTATCCACA | 5.3E-06 | 4104254 | + | -4 |
| 17 | TTGTCCTCA | 1.3E-03 | 4104282 | - | 24 |
| 17 | ATATGAACA | 8.1E-04 | 4104326 | + | 68 |
| 17 | TTTTGCACA | 3.9E-04 | 4104484 | + | 226 |
| 18 | ATATCCCCA | 5.2E-04 | 3648229 | + | -355 |
| 18 | TCATCAACA | 1.9E-04 | 3648548 | + | -36 |
| 18 | AGATCAACA | 1.4E-03 | 3648572 | - | -12 |
| 18 | TTATAAACA | 2.0E-04 | 3648590 | + | 6 |
| 18 | CCATCAACA | 8.7E-04 | 3648690 | + | 106 |
| 19 | TTATCCCCA | 1.3E-04 | 2184370 | + | 3 |
| 19 | TTATCCACC | 3.9E-04 | 2184382 | + | 15 |
| 20 | ATCTCCACA | 1.0E-03 | 722356 | - | -137 |
| 20 | ATATCCACA | 2.8E-05 | 722467 | - | -26 |
| 20 | TTATCAACA | 2.3E-05 | 722508 | - | 15 |
| 20 | ATATCACCA | 1.4E-03 | 722776 | - | 283 |
| 21 | ATTTTAACA | 1.5E-03 | 1874926 | - | -319 |
| 21 | TAATCCACA | 8.2E-05 | 1874945 | + | -300 |
| 21 | ATATCCACT | 7.0E-04 | 1875209 | + | -36 |
| 21 | TCATCCACT | 9.6E-04 | 1875218 | + | -27 |

| peak | sequence | p_value | start | strand | bp to peak |
|---|---|---|---|---|---|
| 21 | GTATCAACA | 2.3E-04 | 1875251 | + | 6 |
| 21 | TTATGAACA | 1.9E-04 | 1875297 | - | 52 |
| 22 | TTATCCACA | 5.3E-06 | 2901929 | + | -45 |
| 22 | ATATCCACA | 2.8E-05 | 2901980 | + | 6 |
| 22 | AAATCAACA | 1.1E-03 | 2902148 | + | 174 |
| 23 | TTATCCAGA | 7.0E-04 | 2254794 | + | -45 |
| 23 | TTTTCCCCA | 6.2E-04 | 2254843 | - | 4 |
| 23 | TAATCAACA | 2.6E-04 | 2254869 | + | 30 |
| 23 | TTATAAACA | 2.0E-04 | 2255018 | + | 179 |
| 24 | TTTTTAACA | 4.3E-04 | 1253432 | + | -384 |
| 24 | TTATTTACA | 1.3E-03 | 1253696 | - | -120 |
| 24 | TTATTAACA | 7.3E-05 | 1253706 | - | -110 |
| 24 | TCGTTCACA | 3.4E-04 | 1253717 | - | -99 |
| 24 | CTATTAACA | 3.9E-04 | 1253817 | - | 1 |
| 24 | ATTTCCACA | 2.3E-04 | 1253858 | - | 42 |
| 25 | GTGTCCACA | 2.0E-04 | 2036985 | - | -339 |
| 25 | AAATCCACA | 4.3E-04 | 2037062 | - | -262 |
| 25 | ATATCGACA | 1.3E-03 | 2037266 | + | -58 |
| 25 | TTACTCACA | 2.9E-04 | 2037299 | - | -25 |
| 25 | ATATCAACA | 1.3E-04 | 2037343 | - | 19 |
| 25 | TTTTGAACA | 9.6E-04 | 2037393 | - | 69 |
| 25 | TTTTCAACA | 1.7E-04 | 2037429 | + | 105 |
| 25 | ATATCAACA | 1.3E-04 | 2037662 | + | 338 |
| 26 | AAATTCACA | 9.6E-04 | 2241836 | - | -255 |
| 26 | TTATCCCCA | 1.3E-04 | 2242042 | + | -49 |
| 26 | TTATCCCCA | 1.3E-04 | 2242095 | + | 4 |
| 26 | TAGTCCACA | 2.1E-04 | 2242172 | + | 81 |
| 26 | GTATGAACA | 1.3E-03 | 2242203 | + | 112 |
| 26 | TAATCCACT | 1.3E-03 | 2242397 | + | 306 |
| 27 | TTATCCACA | 5.3E-06 | 2864210 | + | -4 |
| 27 | TTATCTACA | 6.2E-04 | 2864309 | + | 95 |
| 27 | AAATCAACA | 1.1E-03 | 2864495 | + | 281 |
| 28 | TTATCCACA | 5.3E-06 | 2657843 | + | -49 |
| 28 | TTATTCACC | 8.7E-04 | 2657897 | + | 5 |
| 28 | TTATCTACA | 6.2E-04 | 2658001 | + | 109 |
| 28 | TTATTCATA | 1.0E-03 | 2658022 | + | 130 |
| 28 | TATTTCACA | 1.1E-03 | 2658040 | - | 148 |
| 29 | ATGTTCACA | 2.3E-04 | 3766372 | + | -14 |
| 29 | TCTTCCACA | 3.4E-04 | 3766589 | + | 203 |
| 29 | CCATTCACA | 7.0E-04 | 3766659 | - | 273 |
| 29 | TCTTCCACA | 3.4E-04 | 3766724 | + | 338 |
| 30 | TTCTTAACA | 1.5E-03 | 3637019 | - | -40 |
| 30 | TAATTCACA | 2.1E-04 | 3637029 | - | -30 |
| 30 | TTAACAACA | 4.9E-04 | 3637056 | - | -3 |
| 30 | TTAATAACA | 1.1E-03 | 3637296 | + | 237 |
| 31 | TTGCCAACA | 8.1E-04 | 537404 | + | -328 |

| peak | sequence | p_value | start | strand | bp to peak |
|---|---|---|---|---|---|
| 31 | TTTTGAACA | 9.6E-04 | 537523 | - | -209 |
| 31 | TAATCCACA | 8.2E-05 | 537680 | - | -52 |
| 31 | GTATCCCCA | 9.6E-04 | 537727 | - | -5 |
| 31 | TCATACACA | 4.3E-04 | 537766 | + | 34 |
| 31 | TTAACCACA | 1.9E-04 | 537790 | - | 58 |
| 31 | ATATGAACA | 8.1E-04 | 537898 | - | 166 |
| 31 | ATATGAACA | 8.1E-04 | 538021 | + | 289 |
| 32 | TTATTCACA | 1.6E-05 | 3930964 | - | -51 |
| 32 | ATGTCCACA | 9.3E-05 | 3931017 | - | 2 |
| 32 | ATTTGCACA | 1.3E-03 | 3931039 | - | 24 |
| 32 | TTTTTCACA | 1.3E-04 | 3931066 | + | 51 |
| 32 | ATATGAACA | 8.1E-04 | 3931284 | + | 269 |
| 33 | ATTTCAACA | 7.0E-04 | 3444517 | - | -309 |
| 33 | ATACCAACA | 1.3E-03 | 3444749 | + | -77 |
| 33 | ATATTCACT | 1.4E-03 | 3444779 | + | -47 |
| 33 | TTATCAATA | 1.2E-03 | 3444801 | + | -25 |
| 33 | TAGTTAACA | 1.3E-03 | 3444897 | + | 71 |
| 34 | ATATCAACA | 1.3E-04 | 2230877 | - | -257 |
| 34 | CTATTAACA | 3.9E-04 | 2230913 | - | -221 |
| 34 | TTATTAACT | 1.0E-03 | 2230970 | - | -164 |
| 34 | TGATCAACA | 4.3E-04 | 2231030 | + | -104 |
| 34 | GCATCCACA | 4.9E-04 | 2231124 | + | -10 |
| 34 | TAATTAACA | 6.2E-04 | 2231171 | - | 37 |
| 34 | TTGACAACA | 1.0E-03 | 2231255 | - | 121 |
| 34 | TTGTCCACT | 3.9E-04 | 2231344 | + | 210 |
| 35 | TTATCCACT | 1.7E-04 | 4103024 | + | -4 |
| 35 | TCATCAACA | 1.9E-04 | 4103115 | - | 87 |
| 35 | TTATCCAGA | 7.0E-04 | 4103315 | + | 287 |
| 36 | CCTTCCACA | 1.4E-03 | 2046802 | - | -169 |
| 36 | TTTTTCACA | 1.3E-04 | 2046865 | - | -106 |
| 36 | TTATGAACA | 1.9E-04 | 2046964 | + | -7 |
| 36 | CTGTTCACA | 2.6E-04 | 2047036 | - | 65 |
| 36 | ATATTCACT | 1.4E-03 | 2047066 | - | 95 |
| 36 | TTTTCAACA | 1.7E-04 | 2047232 | - | 261 |
| 37 | ACATTCACA | 6.2E-04 | 513202 | - | -286 |
| 37 | ACATTCACA | 6.2E-04 | 513328 | - | -160 |
| 37 | TTATGCACA | 5.9E-05 | 513487 | + | -1 |
| 37 | TTTTAAACA | 1.0E-03 | 513590 | + | 102 |
| 38 | TCAACCACA | 1.0E-03 | 1005752 | + | -373 |
| 38 | TCGTCCCCA | 1.4E-03 | 1006090 | - | -35 |
| 38 | TTGTTCACA | 4.3E-05 | 1006106 | - | -19 |
| 38 | TTGTTCACT | 8.7E-04 | 1006159 | - | 34 |
| 38 | ATTTCAACA | 7.0E-04 | 1006205 | + | 80 |
| 39 | TTATTCACA | 1.6E-05 | 2001370 | + | -10 |
| 39 | AAATTCACA | 9.6E-04 | 2001392 | + | 12 |
| 39 | ATTTCAACA | 7.0E-04 | 2001590 | + | 210 |

| peak | sequence | p_value | start | strand | bp to peak |
|---|---|---|---|---|---|
| 39 | TTACCCCCA | 1.3E-03 | 2001749 | + | 369 |
| 40 | GTTTCCACA | 4.6E-04 | 624755 | - | -14 |
| 40 | TTATCCACA | 5.3E-06 | 624772 | + | 3 |
| 40 | CTTTCAACA | 8.1E-04 | 624920 | - | 151 |
| 41 | ACATACACA | 1.5E-03 | 2143834 | - | -343 |
| 41 | ATATTCACT | 1.4E-03 | 2143903 | + | -274 |
| 41 | TTTTAAACA | 1.0E-03 | 2144152 | - | -25 |
| 41 | TTATTCAGA | 1.4E-03 | 2144169 | + | -8 |
| 41 | TTATTAACA | 7.3E-05 | 2144182 | + | 5 |
| 42 | TTGTTAACA | 1.7E-04 | 3360149 | - | -1 |
| 42 | ATGTCCACA | 9.3E-05 | 3360180 | + | 30 |
| 43 | TAATCCACA | 8.2E-05 | 3282009 | - | -32 |
| 43 | TTCTTCACA | 5.7E-04 | 3282024 | - | -17 |
| 43 | TTATCCCCA | 1.3E-04 | 3282045 | - | 4 |
| 44 | ATGTCCCCA | 1.1E-03 | 4185782 | - | -41 |
| 44 | TTATCCACA | 5.3E-06 | 4185822 | + | -1 |
| 44 | ATATTCACT | 1.4E-03 | 4185860 | + | 37 |
| 44 | TTTTTAACA | 4.3E-04 | 4185889 | - | 66 |
| 45 | TTATCCACA | 5.3E-06 | 3887119 | - | -37 |
| 45 | TTATACACA | 5.9E-05 | 3887167 | - | 11 |
| 46 | TTTTAAACA | 1.0E-03 | 2687164 | - | -199 |
| 46 | ATAATCACA | 1.5E-03 | 2687307 | + | -56 |
| 46 | TTATTAACT | 1.0E-03 | 2687366 | + | 3 |
| 46 | TTATTCACA | 1.6E-05 | 2687381 | + | 18 |
| 47 | TTGTCACCA | 8.1E-04 | 2036861 | + | -41 |
| 47 | ATGTGCACA | 6.2E-04 | 2036878 | - | -24 |
| 47 | CTCTCCACA | 1.1E-03 | 2036898 | + | -4 |
| 47 | GTGTCCACA | 2.0E-04 | 2036985 | - | 83 |
| 47 | AAATCCACA | 4.3E-04 | 2037062 | - | 160 |
| 47 | ATATCGACA | 1.3E-03 | 2037266 | + | 364 |
| 48 | ATATTCCCA | 1.2E-03 | 730696 | + | -234 |
| 48 | TTATTCACA | 1.6E-05 | 730928 | - | -2 |
| 49 | TTTTGAACA | 9.6E-04 | 1098545 | + | -210 |
| 49 | AAATCCACA | 4.3E-04 | 1098702 | - | -53 |
| 49 | TTTTGAACA | 9.6E-04 | 1098710 | + | -45 |
| 49 | CCATTCACA | 7.0E-04 | 1098760 | + | 5 |
| 49 | TCATCCACA | 5.9E-05 | 1098782 | + | 27 |
| 49 | TAATCAACA | 2.6E-04 | 1099121 | - | 366 |
| 50 | GTATCAACA | 2.3E-04 | 1535900 | - | -358 |
| 50 | TTATACACA | 5.9E-05 | 1536188 | + | -70 |
| 50 | TTATTAACA | 7.3E-05 | 1536261 | + | 3 |
| 50 | TTTTACACA | 3.9E-04 | 1536325 | + | 67 |
| 50 | TTTTTAACA | 4.3E-04 | 1536451 | - | 193 |
| 50 | TTATTCTCA | 1.4E-03 | 1536634 | + | 376 |
| 51 | TAATTCACA | 2.1E-04 | 3107521 | + | -79 |
| 51 | TTATTAACA | 7.3E-05 | 3107604 | + | 4 |

| peak | sequence | p_value | start | strand | bp to peak |
|---|---|---|---|---|---|
| 51 | ACATCCACA | 2.6E-04 | 3107688 | + | 88 |
| 52 | TAATCAACA | 2.6E-04 | 216184 | - | -51 |
| 52 | TTGTCTACA | 1.3E-03 | 216294 | + | 59 |
| 52 | TTATTAACA | 7.3E-05 | 216315 | + | 80 |
| 53 | TCATTAACA | 4.6E-04 | 2760372 | - | -263 |
| 53 | AAATTCACA | 9.6E-04 | 2760493 | + | -142 |
| 53 | CTATTCACA | 1.1E-04 | 2760592 | + | -43 |
| 53 | TTATCCACA | 5.3E-06 | 2760632 | + | -3 |
| 53 | GTATCAACA | 2.3E-04 | 2760784 | - | 149 |
| 54 | CAATCCACA | 4.6E-04 | 2009753 | - | -68 |
| 54 | TCATCAACA | 1.9E-04 | 2009887 | - | 66 |
| 54 | ATTTCCACA | 2.3E-04 | 2010214 | + | 393 |
| 55 | CCTTCCACA | 1.4E-03 | 3822968 | + | -16 |
| 55 | CTATCCACA | 3.2E-05 | 3822986 | + | 2 |
| 56 | AAATCCACA | 4.3E-04 | 2040584 | + | -23 |
| 56 | TTGCTAACA | 1.5E-03 | 2040618 | + | 11 |
| 56 | ATACCAACA | 1.3E-03 | 2040662 | - | 55 |
| 57 | TTTTCCACA | 4.3E-05 | 2861520 | + | -67 |
| 57 | GTATCAACA | 2.3E-04 | 2861624 | + | 37 |
| 57 | TTGTTCACA | 4.3E-05 | 2861635 | - | 48 |
| 58 | TTATTAACT | 1.0E-03 | 4111680 | - | -386 |
| 58 | TTATCAATA | 1.2E-03 | 4111731 | - | -335 |
| 58 | TTATACACA | 5.9E-05 | 4112038 | + | -28 |
| 58 | TTATTAACT | 1.0E-03 | 4112070 | - | 4 |
| 58 | CTTTCCACA | 2.9E-04 | 4112301 | + | 235 |
| 59 | TCATCAACA | 1.9E-04 | 3430955 | + | -157 |
| 59 | TCATCAACA | 1.9E-04 | 3431099 | + | -13 |
| 59 | TTATTCACA | 1.6E-05 | 3431114 | + | 2 |
| 59 | TTGTCACCA | 8.1E-04 | 3431466 | - | 354 |
| 60 | ATATGCACA | 2.9E-04 | 2170488 | + | -361 |
| 60 | TTATTAACA | 7.3E-05 | 2170826 | + | -23 |
| 60 | TTATCTACA | 6.2E-04 | 2170866 | + | 17 |
| 60 | TCATTCCCA | 1.5E-03 | 2170881 | + | 32 |
| 61 | ATATCAACA | 1.3E-04 | 918657 | + | -49 |
| 61 | GTGTCCACA | 2.0E-04 | 918691 | + | -15 |
| 61 | TTATCCTCA | 6.2E-04 | 918723 | - | 17 |
| 61 | ATATAAACA | 8.7E-04 | 918813 | + | 107 |
| 62 | ATATACACA | 2.9E-04 | 3222540 | - | -316 |
| 62 | TAATAAACA | 1.5E-03 | 3222722 | - | -134 |
| 62 | TTATACACA | 5.9E-05 | 3222919 | + | 63 |
| 62 | GTATGAACA | 1.3E-03 | 3223220 | + | 364 |
| 63 | TTGTGCACA | 1.5E-04 | 415844 | + | -228 |
| 63 | ATATCCCCA | 5.2E-04 | 416085 | + | 13 |
| 63 | TTATCAACA | 2.3E-05 | 416094 | - | 22 |
| 63 | AGATCAACA | 1.4E-03 | 416203 | + | 131 |
| 64 | TTATCCACA | 5.3E-06 | 202042 | - | -42 |

| peak | sequence | p_value | start | strand | bp to peak |
|---|---|---|---|---|---|
| 64 | TTTTCCACT | 8.7E-04 | 202160 | - | 76 |
| 64 | GTATTAACA | 5.2E-04 | 202316 | - | 232 |
| 64 | TTATTGACA | 8.1E-04 | 202434 | + | 350 |
| 65 | TTATCAACG | 1.0E-03 | 2171704 | + | -24 |
| 65 | TTTTCCCCA | 6.2E-04 | 2171728 | + | 0 |
| 65 | GTATACACA | 5.2E-04 | 2171761 | - | 33 |
| 66 | ATATCACCA | 1.4E-03 | 643815 | - | -129 |
| 66 | TTATCAACA | 2.3E-05 | 643938 | - | -6 |
| 66 | ATGTCCCCA | 1.1E-03 | 644043 | - | 99 |
| 66 | TCATCAACA | 1.9E-04 | 644052 | - | 108 |
| 66 | TTATCACCA | 3.9E-04 | 644311 | - | 367 |
| 67 | ATATTCACT | 1.4E-03 | 3645140 | - | -244 |
| 67 | TTATCCACT | 1.7E-04 | 3645395 | + | 11 |
| 67 | TTATCAATA | 1.2E-03 | 3645488 | - | 104 |
| 67 | ATATTCACT | 1.4E-03 | 3645748 | + | 364 |
| 68 | ATGTTCACA | 2.3E-04 | 666568 | - | -145 |
| 68 | CAATTCACA | 1.0E-03 | 666695 | - | -18 |
| 68 | TTCTCAACA | 7.0E-04 | 666706 | - | -7 |
| 68 | TTATTCACA | 1.6E-05 | 666779 | + | 66 |
| 68 | TGATCCCCA | 1.4E-03 | 667017 | - | 304 |
| 69 | TGTTTCACA | 1.4E-03 | 2042255 | + | -196 |
| 69 | ATAATCACA | 1.5E-03 | 2042392 | + | -59 |
| 69 | TTTTACACA | 3.9E-04 | 2042441 | + | -10 |
| 69 | ATATCGACA | 1.3E-03 | 2042456 | + | 5 |
| 69 | AAATTCACA | 9.6E-04 | 2042541 | - | 90 |
| 69 | ATGTCAACA | 2.9E-04 | 2042651 | + | 200 |
| 69 | CTACCAACA | 1.4E-03 | 2042827 | - | 376 |
| 70 | TTATCTACA | 6.2E-04 | 2216934 | + | -46 |
| 70 | TTATTGACA | 8.1E-04 | 2217039 | - | 59 |
| 70 | TGATCAACA | 4.3E-04 | 2217153 | + | 173 |
| 70 | AAATCAACA | 1.1E-03 | 2217194 | + | 214 |
| 70 | TTATTAACA | 7.3E-05 | 2217323 | + | 343 |
| 71 | GTATACACA | 5.2E-04 | 1886249 | - | -311 |
| 71 | GTGTACACA | 1.1E-03 | 1886435 | + | -125 |
| 71 | CTATAAACA | 9.6E-04 | 1886566 | + | 6 |
| 71 | TTGTCCCCA | 2.9E-04 | 1886575 | - | 15 |
| 71 | CTATTCACT | 1.5E-03 | 1886628 | - | 68 |
| 71 | TTATTCAGA | 1.4E-03 | 1886741 | + | 181 |
| 72 | GTTTCCACA | 4.6E-04 | 546254 | - | -122 |
| 72 | TTATCCCCA | 1.3E-04 | 546382 | + | 6 |
| 72 | TTATCTACA | 6.2E-04 | 546401 | + | 25 |
| 72 | TTGTTAACA | 1.7E-04 | 546488 | - | 112 |
| 73 | TTTCTCACA | 1.3E-03 | 1369223 | + | -291 |
| 73 | TTAATAACA | 1.1E-03 | 1369447 | - | -67 |
| 73 | TTACCAACA | 3.9E-04 | 1369478 | - | -36 |
| 73 | TTGTCAACA | 7.3E-05 | 1369509 | + | -5 |

| peak | sequence | p_value | start | strand | bp to peak |
|---|---|---|---|---|---|
| 73 | TTATTTACA | 1.3E-03 | 1369533 | + | 19 |
| 73 | TTATCCACC | 3.9E-04 | 1369836 | - | 322 |
| 74 | TTGTCTACA | 1.3E-03 | 2228985 | - | -326 |
| 74 | TTGTCCATA | 1.0E-03 | 2229229 | - | -82 |
| 74 | TTCTTAACA | 1.5E-03 | 2229273 | - | -38 |
| 74 | ATGTCCACA | 9.3E-05 | 2229306 | - | -5 |
| 74 | TTCTCAACA | 7.0E-04 | 2229360 | - | 49 |
| 74 | ATTTTAACA | 1.5E-03 | 2229508 | + | 197 |
| 75 | TTTTCCACT | 8.7E-04 | 3233583 | + | -39 |
| 75 | ATATTAACA | 3.4E-04 | 3233609 | - | -13 |
| 75 | ATATCCCCA | 5.2E-04 | 3233625 | + | 3 |
| 75 | TTGTCCATA | 1.0E-03 | 3233706 | + | 84 |
| 75 | AAATTCACA | 9.6E-04 | 3233850 | - | 228 |
| 76 | TGATCCCCA | 1.4E-03 | 2210860 | - | -286 |
| 76 | TTTTACACA | 3.9E-04 | 2211045 | - | -101 |
| 76 | ATTTTAACA | 1.5E-03 | 2211140 | + | -6 |
| 76 | TTTCCAACA | 1.5E-03 | 2211272 | + | 126 |
| 76 | TGTTCCACA | 7.0E-04 | 2211504 | - | 358 |
| 77 | TTTTCCACA | 4.3E-05 | 2870061 | - | -28 |
| 77 | TTATCCTCA | 6.2E-04 | 2870085 | + | -4 |
| 77 | GTGTCAACA | 5.2E-04 | 2870248 | + | 159 |
| 77 | ATATAAACA | 8.7E-04 | 2870404 | + | 315 |
| 78 | ATATCAACA | 1.3E-04 | 1768475 | + | -90 |
| 78 | TTTTTCACA | 1.3E-04 | 1768560 | + | -5 |
| 78 | TTAACAACA | 4.9E-04 | 1768616 | + | 51 |
| 78 | TTATACACA | 5.9E-05 | 1768675 | + | 110 |
| 78 | TCTTCAACA | 9.6E-04 | 1768829 | - | 264 |
| 78 | TTTTTCCCA | 1.4E-03 | 1768886 | + | 321 |
| 79 | ATATTAACA | 3.4E-04 | 1250048 | - | -25 |
| 79 | TTTTCAACA | 1.7E-04 | 1250075 | + | 2 |
| 80 | CCATCAACA | 8.7E-04 | 727825 | + | -132 |
| 80 | AGATTCACA | 1.2E-03 | 727893 | + | -64 |
| 80 | TTATAAACA | 2.0E-04 | 728105 | + | 148 |
| 81 | TCATTAACA | 4.6E-04 | 646029 | - | -391 |
| 81 | AAATCAACA | 1.1E-03 | 646356 | - | -64 |
| 81 | ATATAAACA | 8.7E-04 | 646395 | - | -25 |
| 81 | TTTTCCACT | 8.7E-04 | 646447 | + | 27 |
| 81 | TTGTAAACA | 4.6E-04 | 646544 | - | 124 |
| 82 | TTGATCACA | 8.7E-04 | 2302293 | + | -273 |
| 82 | TATTCAACA | 1.3E-03 | 2302325 | - | -241 |
| 82 | ACATGCACA | 1.4E-03 | 2302558 | + | -8 |
| 82 | TTATCAACA | 2.3E-05 | 2302603 | + | 37 |
| 82 | ATGTCAACA | 2.9E-04 | 2302631 | - | 65 |
| 82 | TTATCAATA | 1.2E-03 | 2302889 | - | 323 |
| 83 | TTGCCAACA | 8.1E-04 | 1856118 | - | -121 |
| 83 | ATATCAACA | 1.3E-04 | 1856280 | - | 41 |

| peak | sequence | p_value | start | strand | bp to peak |
|---|---|---|---|---|---|
| 84 | AAATCCACA | 4.3E-04 | 2244172 | - | -273 |
| 84 | ATATCGACA | 1.3E-03 | 2244376 | + | -69 |
| 84 | TTACTCACA | 2.9E-04 | 2244409 | - | -36 |
| 84 | ATATCAACA | 1.3E-04 | 2244453 | - | 8 |
| 84 | TTTTGAACA | 9.6E-04 | 2244503 | - | 58 |
| 84 | TTTTTCACA | 1.3E-04 | 2244555 | - | 110 |
| 84 | TGATCAACA | 4.3E-04 | 2244629 | - | 184 |
| 84 | ATATTAACA | 3.4E-04 | 2244776 | + | 331 |
| 84 | TTACCAACA | 3.9E-04 | 2244835 | - | 390 |
| 85 | GCATCAACA | 1.3E-03 | 1974956 | + | -338 |
| 85 | TTTTAAACA | 1.0E-03 | 1975148 | + | -146 |
| 85 | TTTTACACA | 3.9E-04 | 1975241 | + | -53 |
| 85 | TTACTCACA | 2.9E-04 | 1975297 | - | 3 |
| 85 | TTAAGCACA | 1.0E-03 | 1975453 | + | 159 |
| 86 | TAATCCACA | 8.2E-05 | 3900226 | + | -36 |
| 86 | ATATTCACA | 9.3E-05 | 3900268 | + | 6 |
| 86 | GTTTTCACA | 1.0E-03 | 3900282 | - | 20 |
| 87 | TTATTTACA | 1.3E-03 | 2681545 | + | -332 |
| 87 | TTATCACCA | 3.9E-04 | 2681722 | + | -155 |
| 87 | TTATTAACT | 1.0E-03 | 2681789 | + | -88 |
| 87 | TTTTCCACA | 4.3E-05 | 2681923 | - | 46 |
| 87 | TTATCAACG | 1.0E-03 | 2681936 | - | 59 |
| 88 | ATTTGCACA | 1.3E-03 | 650778 | - | -279 |
| 88 | TTTTTAACA | 4.3E-04 | 650924 | - | -133 |
| 88 | TTATGCACA | 5.9E-05 | 651051 | + | -6 |
| 88 | TAATCAACA | 2.6E-04 | 651117 | - | 60 |
| 88 | TTTTCCACA | 4.3E-05 | 651196 | - | 139 |
| 89 | TTAAACACA | 1.0E-03 | 526032 | - | -305 |
| 89 | TTGTCAACA | 7.3E-05 | 526299 | - | -38 |
| 89 | CAATCCACA | 4.6E-04 | 526370 | - | 33 |
| 89 | GTTTTCACA | 1.0E-03 | 526379 | + | 42 |
| 89 | TTATCAACT | 4.9E-04 | 526434 | - | 97 |
| 89 | TTGTTCCCA | 6.2E-04 | 526486 | - | 149 |
| 90 | TTATTCATA | 1.0E-03 | 4152557 | - | -224 |
| 90 | TTAATAACA | 1.1E-03 | 4152758 | - | -23 |
| 90 | GTGTACACA | 1.1E-03 | 4152773 | + | -8 |
| 90 | TAATAAACA | 1.5E-03 | 4152831 | - | 50 |
| 90 | ATATACACA | 2.9E-04 | 4152935 | - | 154 |
| 90 | ATATTCACA | 9.3E-05 | 4153020 | + | 239 |
| 91 | ATATGAACA | 8.1E-04 | 2630360 | - | -353 |
| 91 | ATATTCACA | 9.3E-05 | 2630638 | - | -75 |
| 91 | CCTTCCACA | 1.4E-03 | 2630747 | + | 34 |
| 91 | TTTTAAACA | 1.0E-03 | 2630761 | + | 48 |
| 91 | ACGTCAACA | 1.5E-03 | 2631017 | + | 304 |
| 92 | ATATGAACA | 8.1E-04 | 211543 | + | -263 |
| 92 | ATATCGACA | 1.3E-03 | 211777 | + | -29 |

| peak | sequence | p_value | start | strand | bp to peak |
|---|---|---|---|---|---|
| 92 | TAATAAACA | 1.5E-03 | 211836 | - | 30 |
| 92 | TTATTACCA | 8.7E-04 | 212000 | + | 194 |
| 93 | TTATCAACT | 4.9E-04 | 543310 | + | 8 |
| 93 | ATATCCACT | 7.0E-04 | 543369 | - | 67 |
| 93 | TGATTCACA | 3.4E-04 | 543503 | - | 201 |
| 93 | TTATTTACA | 1.3E-03 | 543577 | - | 275 |
| 94 | TATTCAACA | 1.3E-03 | 347558 | + | -65 |
| 94 | CAATCCACA | 4.6E-04 | 347597 | - | -26 |
| 94 | TTTTCCACA | 4.3E-05 | 347723 | + | 100 |
| 94 | TTATTACCA | 8.7E-04 | 347988 | + | 365 |
| 95 | TTTTAAACA | 1.0E-03 | 2946809 | - | -95 |
| 95 | TTATCTACA | 6.2E-04 | 2946911 | + | 7 |
| 95 | ATACCAACA | 1.3E-03 | 2946933 | + | 29 |
| 95 | CTATCAACA | 1.5E-04 | 2947026 | + | 122 |
| 96 | ATATGAACA | 8.1E-04 | 659544 | + | -237 |
| 96 | TGATTCACA | 3.4E-04 | 659575 | + | -206 |
| 96 | TTATGCACA | 5.9E-05 | 659778 | + | -3 |
| 96 | TTATTCATA | 1.0E-03 | 659989 | - | 208 |
| 96 | TTGTCACCA | 8.1E-04 | 660150 | + | 369 |
| 97 | TTATTCACC | 8.7E-04 | 533438 | - | -391 |
| 97 | TTTTCCACA | 4.3E-05 | 533523 | - | -306 |
| 97 | TAATAAACA | 1.5E-03 | 533612 | - | -217 |
| 97 | TTTTAAACA | 1.0E-03 | 533806 | + | -23 |
| 97 | TCATCCACA | 5.9E-05 | 533817 | + | -12 |
| 97 | TTTCCAACA | 1.5E-03 | 533913 | - | 84 |
| 97 | TTCTCCACA | 2.6E-04 | 533980 | - | 151 |
| 97 | TTATTTACA | 1.3E-03 | 534165 | - | 336 |
| 98 | TTCTTAACA | 1.5E-03 | 584999 | - | -220 |
| 98 | TTATTCTCA | 1.4E-03 | 585105 | - | -114 |
| 98 | ATATCCCCA | 5.2E-04 | 585213 | - | -6 |
| 98 | TGATCCACA | 1.3E-04 | 585225 | - | 6 |
| 98 | TTATCCATA | 4.9E-04 | 585258 | - | 39 |
| 98 | TCATACACA | 4.3E-04 | 585529 | + | 310 |
| 98 | ATTTCCACA | 2.3E-04 | 585544 | + | 325 |
| 99 | TTTTTCCCA | 1.4E-03 | 2860335 | + | -163 |
| 99 | TTATTAACA | 7.3E-05 | 2860469 | - | -29 |
| 99 | TTATACACA | 5.9E-05 | 2860518 | + | 20 |
| 99 | TTCTCCACA | 2.6E-04 | 2860786 | + | 288 |
| 100 | TTATCAACA | 2.3E-05 | 3664578 | - | 5 |
| 100 | ACGTTCACA | 1.3E-03 | 3664790 | - | 217 |
| 101 | TTATGAACA | 1.9E-04 | 1518956 | + | -370 |
| 101 | TTATCACCA | 3.9E-04 | 1518983 | + | -343 |
| 101 | TTATACACA | 5.9E-05 | 1519273 | - | -53 |
| 101 | TAATACACA | 5.7E-04 | 1519342 | - | 16 |
| 102 | TTATTCACA | 1.6E-05 | 2572747 | + | -16 |
| 102 | TTATCCATA | 4.9E-04 | 2572925 | + | 162 |

| peak | sequence | p_value | start | strand | bp to peak |
|---|---|---|---|---|---|
| 103 | TTATCAACA | 2.3E-05 | 3661152 | + | -1 |
| 103 | TTATCAACC | 1.0E-03 | 3661173 | - | 20 |
| 103 | TTTTGAACA | 9.6E-04 | 3661368 | - | 215 |
| 104 | TCGTCCACA | 1.5E-04 | 1096308 | - | -90 |
| 104 | AAATCAACA | 1.1E-03 | 1096404 | - | 6 |
| 104 | TTGTTCACA | 4.3E-05 | 1096424 | + | 26 |
| 104 | TCTTCAACA | 9.6E-04 | 1096730 | + | 332 |
| 104 | TTGTCCACA | 9.3E-06 | 1096790 | + | 392 |
| 105 | TTTTTAACA | 4.3E-04 | 3599544 | + | -118 |
| 105 | TCATCCACA | 5.9E-05 | 3599670 | + | 8 |
| 105 | ACATCCACA | 2.6E-04 | 3599720 | + | 58 |
| 106 | TTCTTAACA | 1.5E-03 | 1657089 | - | -375 |
| 106 | ATATTAACA | 3.4E-04 | 1657125 | - | -339 |
| 106 | ATTTCAACA | 7.0E-04 | 1657141 | - | -323 |
| 106 | TGATCAACA | 4.3E-04 | 1657448 | + | -16 |
| 106 | TTGTCCACA | 9.3E-06 | 1657471 | + | 7 |
| 106 | TGATCAACA | 4.3E-04 | 1657493 | - | 29 |
| 106 | TTATTCACC | 8.7E-04 | 1657817 | + | 353 |
| 107 | TTACCAACA | 3.9E-04 | 1866389 | - | -200 |
| 107 | GCATCCACA | 4.9E-04 | 1866477 | + | -112 |
| 107 | TTGTTCACA | 4.3E-05 | 1866585 | + | -4 |
| 107 | TATTCAACA | 1.3E-03 | 1866632 | - | 43 |
| 107 | TAATGCACA | 5.2E-04 | 1866714 | + | 125 |
| 107 | TTATTTACA | 1.3E-03 | 1866856 | - | 267 |
| 108 | TGGTCCACA | 2.9E-04 | 2711790 | - | -176 |
| 108 | TTATACACA | 5.9E-05 | 2711961 | + | -5 |
| 108 | ATTTACACA | 1.4E-03 | 2712144 | - | 178 |
| 108 | ACATGCACA | 1.4E-03 | 2712163 | - | 197 |
| 109 | TCTTCCACA | 3.4E-04 | 4110576 | + | -67 |
| 109 | TTATTAACT | 1.0E-03 | 4110596 | - | -47 |
| 109 | TTATTCACT | 4.3E-04 | 4110654 | + | 11 |
| 109 | TTCTTAACA | 1.5E-03 | 4110769 | + | 126 |
| 109 | ATTTTAACA | 1.5E-03 | 4110825 | - | 182 |
| 109 | TCATTAACA | 4.6E-04 | 4110898 | + | 255 |
| 110 | TGATCAACA | 4.3E-04 | 3300863 | - | -113 |
| 110 | GTTTTCACA | 1.0E-03 | 3300952 | + | -24 |
| 110 | TTGTAAACA | 4.6E-04 | 3300962 | - | -14 |
| 110 | TAATACACA | 5.7E-04 | 3300973 | - | -3 |
| 110 | CTGTCAACA | 3.4E-04 | 3301173 | - | 197 |
| 111 | TTATAAACA | 2.0E-04 | 4015025 | + | 4 |
| 111 | TTTTTCCCA | 1.4E-03 | 4015334 | + | 313 |
| 112 | CGATCCACA | 6.2E-04 | 121546 | - | -170 |
| 112 | ATATTAACA | 3.4E-04 | 121690 | - | -26 |
| 112 | ATATCACCA | 1.4E-03 | 122072 | + | 356 |
| 113 | ATATCGACA | 1.3E-03 | 3659098 | + | -321 |
| 113 | TCTTCAACA | 9.6E-04 | 3659305 | - | -114 |

| peak | sequence | p_value | start | strand | bp to peak |
|---|---|---|---|---|---|
| 113 | ATTTTAACA | 1.5E-03 | 3659318 | - | -101 |
| 113 | CTATTCACA | 1.1E-04 | 3659421 | + | 2 |
| 113 | ATGTTAACA | 7.0E-04 | 3659439 | + | 20 |
| 113 | ATTTCCACA | 2.3E-04 | 3659513 | + | 94 |
| 113 | TTGCTAACA | 1.5E-03 | 3659697 | + | 278 |
| 113 | TTATTCAGA | 1.4E-03 | 3659806 | + | 387 |
| 114 | ATATCCACT | 7.0E-04 | 2611132 | + | -176 |
| 114 | TTATTCACA | 1.6E-05 | 2611269 | - | -39 |
| 114 | ACATCAACA | 8.1E-04 | 2611321 | + | 13 |
| 114 | TTAATCACA | 4.3E-04 | 2611341 | - | 33 |
| 114 | TTATCAATA | 1.2E-03 | 2611364 | + | 56 |
| 114 | CCATCAACA | 8.7E-04 | 2611475 | + | 167 |
| 115 | TAATAAACA | 1.5E-03 | 3661958 | + | -193 |
| 115 | CTATTAACA | 3.9E-04 | 3662026 | + | -125 |
| 115 | TTATTTACA | 1.3E-03 | 3662051 | + | -100 |
| 115 | TTGCCAACA | 8.1E-04 | 3662183 | - | 32 |
| 115 | TTGTCCACC | 8.1E-04 | 3662196 | + | 45 |
| 115 | TTATTCTCA | 1.4E-03 | 3662210 | - | 59 |
| 115 | TTTTCCACT | 8.7E-04 | 3662295 | - | 144 |
| 116 | GTTTTCACA | 1.0E-03 | 3442609 | - | -254 |
| 116 | TTCTCAACA | 7.0E-04 | 3442887 | + | 24 |
| 116 | ATTTTCACA | 5.7E-04 | 3442959 | - | 96 |
| 116 | TCTTTCACA | 8.1E-04 | 3443247 | + | 384 |
| 117 | ATGTCCACA | 9.3E-05 | 2128220 | + | -94 |
| 117 | CTGTTCACA | 2.6E-04 | 2128303 | - | -11 |
| 117 | CTTTCCACA | 2.9E-04 | 2128378 | + | 64 |
| 118 | CTGTGCACA | 7.0E-04 | 548092 | - | -24 |
| 118 | TTATGAACA | 1.9E-04 | 548173 | - | 57 |
| 118 | TCCTCCACA | 1.3E-03 | 548400 | + | 284 |
| 119 | TTTTCAACA | 1.7E-04 | 108396 | - | -337 |
| 119 | TTTTTAACA | 4.3E-04 | 108652 | - | -81 |
| 119 | ATACCAACA | 1.3E-03 | 108721 | - | -12 |
| 119 | TTAATAACA | 1.1E-03 | 108779 | + | 46 |
| 119 | CTATCCACT | 8.1E-04 | 108873 | - | 140 |
| 120 | TTGTCCAGA | 1.4E-03 | 2560487 | - | -151 |
| 120 | TTCTTAACA | 1.5E-03 | 2560532 | + | -106 |
| 120 | TTTTTAACA | 4.3E-04 | 2560555 | + | -83 |
| 120 | TCATCAACA | 1.9E-04 | 2560710 | + | 72 |
| 120 | ATGTCCCCA | 1.1E-03 | 2560928 | + | 290 |
| 121 | TTATACACT | 9.6E-04 | 2182564 | + | -8 |
| 121 | TAGTCAACA | 5.7E-04 | 2182763 | - | 191 |
| 121 | TTATTAACT | 1.0E-03 | 2182910 | + | 338 |
| 122 | TGGTCAACA | 8.7E-04 | 618442 | - | -76 |
| 122 | TTGTAAACA | 4.6E-04 | 618534 | - | 16 |
| 122 | TTATACACA | 5.9E-05 | 618554 | - | 36 |
| 123 | TTCTTAACA | 1.5E-03 | 2201801 | + | -390 |

| peak | sequence | p_value | start | strand | bp to peak |
|---|---|---|---|---|---|
| 123 | TAATCCACT | 1.3E-03 | 2201976 | + | -215 |
| 123 | ATGTCAACA | 2.9E-04 | 2201995 | + | -196 |
| 123 | TTATTCAGA | 1.4E-03 | 2202176 | - | -15 |
| 123 | TTTTCAACA | 1.7E-04 | 2202323 | - | 132 |
| 123 | CAATCAACA | 1.2E-03 | 2202544 | - | 353 |
| 124 | TGATCAACA | 4.3E-04 | 2321951 | + | -286 |
| 124 | GTGTCCACA | 2.0E-04 | 2322223 | + | -14 |
| 124 | CTTTCAACA | 8.1E-04 | 2322267 | - | 30 |
| 125 | TTGTCCCCA | 2.9E-04 | 4164819 | - | -306 |
| 125 | TCATCAACA | 1.9E-04 | 4165055 | - | -70 |
| 125 | ATTTGCACA | 1.3E-03 | 4165178 | - | 53 |
| 125 | TTATCAATA | 1.2E-03 | 4165297 | + | 172 |
| 126 | TCATCAACA | 1.9E-04 | 3845937 | + | -37 |
| 126 | CTTTCCACA | 2.9E-04 | 3845958 | - | -16 |
| 126 | ATGTTCACA | 2.3E-04 | 3845981 | + | 7 |
| 126 | TTTTCCACA | 4.3E-05 | 3846115 | - | 141 |
| 127 | TTATTCACA | 1.6E-05 | 1922149 | - | -4 |
| 127 | ATTTACACA | 1.4E-03 | 1922205 | - | 52 |
| 127 | CTATTAACA | 3.9E-04 | 1922253 | - | 100 |
| 127 | TTTTTAACA | 4.3E-04 | 1922414 | - | 261 |
| 128 | TTTTTAACA | 4.3E-04 | 2697489 | - | -269 |
| 128 | ATATGAACA | 8.1E-04 | 2697700 | - | -58 |
| 128 | AAATCCACA | 4.3E-04 | 2697754 | + | -4 |
| 128 | TTATAAACA | 2.0E-04 | 2697783 | + | 25 |
| 128 | TTTTTAACA | 4.3E-04 | 2697892 | - | 134 |
| 128 | TTTTCAACA | 1.7E-04 | 2697941 | + | 183 |
| 128 | TCATGAACA | 1.0E-03 | 2698136 | + | 378 |
| 129 | ACATGCACA | 1.4E-03 | 2193956 | - | -349 |
| 129 | TTGTTAACA | 1.7E-04 | 2194263 | + | -42 |
| 129 | AAATCAACA | 1.1E-03 | 2194302 | - | -3 |
| 129 | TTATCCACT | 1.7E-04 | 2194414 | - | 109 |
| 129 | ATTTCCACA | 2.3E-04 | 2194693 | + | 388 |
| 130 | GTTTCAACA | 1.2E-03 | 1320347 | + | -382 |
| 130 | TTGTCCATA | 1.0E-03 | 1320361 | - | -368 |
| 130 | TAATGAACA | 1.4E-03 | 1320607 | + | -122 |
| 130 | TCTTTCACA | 8.1E-04 | 1320616 | - | -113 |
| 130 | TAATACACA | 5.7E-04 | 1320714 | + | -15 |
| 130 | TTTTCAACA | 1.7E-04 | 1320850 | + | 121 |
| 130 | TTATCAACC | 1.0E-03 | 1320946 | - | 217 |
| 130 | TCTTTCACA | 8.1E-04 | 1320979 | + | 250 |
| 130 | TAATGAACA | 1.4E-03 | 1321113 | + | 384 |
| 131 | TTGTCTACA | 1.3E-03 | 2707153 | + | 11 |
| 132 | ACGTCAACA | 1.5E-03 | 3217453 | + | -107 |
| 132 | TTATCAACA | 2.3E-05 | 3217566 | - | 6 |
| 132 | ATACTCACA | 1.1E-03 | 3217808 | + | 248 |
| 133 | GTATAAACA | 1.3E-03 | 1517267 | + | -337 |

| peak | sequence | p_value | start | strand | bp to peak |
|---|---|---|---|---|---|
| 133 | ATGTCAACA | 2.9E-04 | 1517584 | - | -20 |
| 133 | TCATCAACA | 1.9E-04 | 1517775 | - | 171 |
| 134 | TTATTAACA | 7.3E-05 | 1016150 | - | -39 |
| 134 | TCTTCCACA | 3.4E-04 | 1016185 | + | -4 |
| 134 | TTATTCACA | 1.6E-05 | 1016326 | + | 137 |
| 134 | TTTTGAACA | 9.6E-04 | 1016474 | - | 285 |
| 135 | GTATTCACA | 2.0E-04 | 1956219 | - | -60 |
| 135 | TTTTCAACA | 1.7E-04 | 1956293 | - | 14 |
| 135 | GCATCAACA | 1.3E-03 | 1956330 | + | 51 |
| 136 | CTATCCCCA | 5.7E-04 | 2629490 | + | -4 |
| 136 | TTATCCTCA | 6.2E-04 | 2629564 | + | 70 |
| 136 | TTACTAACA | 8.1E-04 | 2629579 | + | 85 |
| 137 | ATATCAACA | 1.3E-04 | 635872 | + | -1 |
| 137 | TTGCTAACA | 1.5E-03 | 635898 | - | 25 |
| 138 | TTATCTACA | 6.2E-04 | 803657 | - | -162 |
| 138 | TTAATAACA | 1.1E-03 | 803833 | + | 14 |
| 138 | TTAATAACA | 1.1E-03 | 803894 | - | 75 |
| 138 | TTTTAAACA | 1.0E-03 | 803983 | + | 164 |
| 139 | TAATAAACA | 1.5E-03 | 3649040 | + | -138 |
| 139 | TCATAAACA | 1.1E-03 | 3649091 | + | -87 |
| 139 | TAATCCACA | 8.2E-05 | 3649177 | + | -1 |
| 139 | CTGTCAACA | 3.4E-04 | 3649280 | + | 102 |
| 140 | TTATTCAGA | 1.4E-03 | 949018 | + | -91 |
| 140 | TTATCCACA | 5.3E-06 | 949114 | - | 5 |
| 141 | TCATTCACA | 1.5E-04 | 3171557 | - | -183 |
| 141 | TTATCACCA | 3.9E-04 | 3171773 | - | 33 |
| 141 | TTACCCCCA | 1.3E-03 | 3171791 | + | 51 |
| 141 | TGATTAACA | 8.7E-04 | 3172088 | + | 348 |
| 142 | ATATTAACA | 3.4E-04 | 3469630 | + | -38 |
| 142 | TTATTGACA | 8.1E-04 | 3469663 | - | -5 |
| 142 | TTGTCAACA | 7.3E-05 | 3469850 | + | 182 |
| 143 | TCATCAACA | 1.9E-04 | 1439870 | - | -20 |
| 143 | ATATTCACA | 9.3E-05 | 1439950 | + | 60 |
| 144 | ATATAAACA | 8.7E-04 | 2702719 | - | -388 |
| 144 | TCATCAACA | 1.9E-04 | 2702814 | + | -293 |
| 144 | TTATTCACA | 1.6E-05 | 2703102 | + | -5 |
| 144 | ATACCAACA | 1.3E-03 | 2703199 | + | 92 |
| 144 | ATTTTAACA | 1.5E-03 | 2703301 | - | 194 |
| 145 | ATATCCACT | 7.0E-04 | 4062483 | + | -139 |
| 145 | TCATGCACA | 4.3E-04 | 4062574 | + | -48 |
| 145 | TCATAAACA | 1.1E-03 | 4062643 | + | 21 |
| 145 | ATATCAACA | 1.3E-04 | 4062944 | + | 322 |
| 146 | CTATCCACC | 1.5E-03 | 2235196 | - | -140 |
| 146 | ACATTCACA | 6.2E-04 | 2235290 | + | -46 |
| 146 | ATAATCACA | 1.5E-03 | 2235350 | + | 14 |
| 146 | TTGTCCAGA | 1.4E-03 | 2235399 | + | 63 |

| peak | sequence | p_value | start | strand | bp to peak |
|---|---|---|---|---|---|
| 146 | CTTTTCACA | 6.2E-04 | 2235456 | - | 120 |
| 146 | TTGTGCACA | 1.5E-04 | 2235631 | - | 295 |
| 147 | GCATCAACA | 1.3E-03 | 2427734 | + | -336 |
| 147 | TTAATAACA | 1.1E-03 | 2427986 | + | -84 |
| 147 | TTAAACACA | 1.0E-03 | 2428019 | + | -51 |
| 147 | ATTTCCACA | 2.3E-04 | 2428075 | - | 5 |
| 147 | ATGTTCACA | 2.3E-04 | 2428124 | + | 54 |
| 147 | CTATGAACA | 8.7E-04 | 2428221 | - | 151 |
| 147 | TTGCCAACA | 8.1E-04 | 2428353 | - | 283 |
| 147 | TTTTTCACA | 1.3E-04 | 2428373 | + | 303 |
| 148 | GTTTCAACA | 1.2E-03 | 2018279 | - | -310 |
| 148 | TTATGAACA | 1.9E-04 | 2018584 | - | -5 |
| 148 | ATTTTAACA | 1.5E-03 | 2018671 | + | 82 |
| 148 | CCATCAACA | 8.7E-04 | 2018911 | - | 322 |
| 149 | GTTTTCACA | 1.0E-03 | 1110867 | + | -289 |
| 149 | AAATCAACA | 1.1E-03 | 1110891 | - | -265 |
| 149 | TTATCTACA | 6.2E-04 | 1110935 | - | -221 |
| 149 | CTATCCCCA | 5.7E-04 | 1111165 | + | 9 |
| 149 | TTATCCCCA | 1.3E-04 | 1111250 | - | 94 |
| 149 | TGTTTCACA | 1.4E-03 | 1111474 | + | 318 |
| 150 | GTATCAACA | 2.3E-04 | 2680128 | + | -167 |
| 150 | TTATTCACA | 1.6E-05 | 2680300 | - | 5 |
| 150 | AAATCCACA | 4.3E-04 | 2680310 | - | 15 |
| 150 | TTTTAAACA | 1.0E-03 | 2680530 | - | 235 |
| 150 | TCATAAACA | 1.1E-03 | 2680557 | + | 262 |
| 151 | TTATCCAGA | 7.0E-04 | 2691714 | + | -356 |
| 151 | GTATCCACT | 1.1E-03 | 2691864 | + | -206 |
| 151 | TCATCCACA | 5.9E-05 | 2692065 | + | -5 |
| 151 | TTATTTACA | 1.3E-03 | 2692410 | + | 340 |
| 152 | CTTTTCACA | 6.2E-04 | 1579899 | - | 39 |
| 152 | TTTCCCACA | 6.2E-04 | 1580123 | - | 263 |
| 153 | TTATGAACA | 1.9E-04 | 532779 | + | 9 |
| 153 | TTATCCTCA | 6.2E-04 | 532837 | - | 67 |
| 154 | TTATCTACA | 6.2E-04 | 2216934 | + | -382 |
| 154 | TTATTGACA | 8.1E-04 | 2217039 | - | -277 |
| 154 | TGATCAACA | 4.3E-04 | 2217153 | + | -163 |
| 154 | AAATCAACA | 1.1E-03 | 2217194 | + | -122 |
| 154 | TTATTAACA | 7.3E-05 | 2217323 | + | 7 |
| 154 | TAGTTAACA | 1.3E-03 | 2217401 | - | 85 |
| 154 | AAATCAACA | 1.1E-03 | 2217422 | + | 106 |
| 154 | TGTTTCACA | 1.4E-03 | 2217559 | - | 243 |
| 154 | TCTTCAACA | 9.6E-04 | 2217598 | - | 282 |
| 155 | TTTTGCACA | 3.9E-04 | 725017 | + | -257 |
| 155 | ATTTGCACA | 1.3E-03 | 725144 | - | -130 |
| 155 | GTATCAACA | 2.3E-04 | 725381 | + | 107 |
| 155 | TCATCAACA | 1.9E-04 | 725572 | - | 298 |

| peak | sequence | p_value | start | strand | bp to peak |
|---|---|---|---|---|---|
| 156 | CTTTTCACA | 6.2E-04 | 2980332 | + | -258 |
| 156 | TTATACACA | 5.9E-05 | 2980570 | - | -20 |
| 157 | TTGACCACA | 4.3E-04 | 1424517 | + | -186 |
| 157 | TTGTGCACA | 1.5E-04 | 1424617 | - | -86 |
| 157 | TTATTTACA | 1.3E-03 | 1424647 | + | -56 |
| 157 | TTATCAACA | 2.3E-05 | 1424731 | - | 28 |
| 157 | TAATAAACA | 1.5E-03 | 1424946 | + | 243 |
| 157 | GAATTCACA | 1.5E-03 | 1424992 | + | 289 |
| 158 | TTTTAAACA | 1.0E-03 | 1900103 | + | -78 |
| 158 | TCATCCACA | 5.9E-05 | 1900179 | - | -2 |
| 158 | ATCTCCACA | 1.0E-03 | 1900212 | - | 31 |
| 158 | TTATACCCA | 8.1E-04 | 1900404 | + | 223 |
| 159 | AAATCAACA | 1.1E-03 | 4009637 | - | -134 |
| 159 | GAATTCACA | 1.5E-03 | 4009723 | - | -48 |
| 159 | TTATCAACA | 2.3E-05 | 4009854 | - | 83 |
| 160 | TTTTTCCCA | 1.4E-03 | 349442 | + | -35 |
| 160 | TTATTAACA | 7.3E-05 | 349483 | + | 6 |
| 160 | CTGTTAACA | 8.1E-04 | 349494 | + | 17 |
| 160 | TTATTCACC | 8.7E-04 | 349843 | + | 366 |
| 161 | TTATCAACA | 2.3E-05 | 2195488 | - | 6 |
| 161 | TTATCAATA | 1.2E-03 | 2195728 | - | 246 |
| 162 | ATTTTAACA | 1.5E-03 | 4109359 | + | -253 |
| 162 | TTGTAAACA | 4.6E-04 | 4109554 | - | -58 |
| 162 | ATATTAACA | 3.4E-04 | 4109735 | - | 123 |
| 163 | TTATACACA | 5.9E-05 | 539626 | - | -8 |
| 163 | TTATTAACA | 7.3E-05 | 539783 | + | 149 |
| 163 | ATTTACACA | 1.4E-03 | 539875 | + | 241 |
| 164 | TTGTCAACA | 7.3E-05 | 3156883 | + | 8 |
| 164 | TGATGCACA | 8.1E-04 | 3156933 | - | 58 |
| 164 | TGATACACA | 8.7E-04 | 3157046 | - | 171 |
| 164 | ATGACCACA | 1.5E-03 | 3157144 | + | 269 |
| 165 | CTTTGCACA | 1.4E-03 | 3744277 | + | -377 |
| 165 | GTGTCAACA | 5.2E-04 | 3744521 | - | -133 |
| 165 | TGATTAACA | 8.7E-04 | 3744608 | - | -46 |
| 165 | TTGTCAACA | 7.3E-05 | 3744663 | + | 9 |
| 166 | GTTTTCACA | 1.0E-03 | 1293174 | + | -249 |
| 166 | CTTTCAACA | 8.1E-04 | 1293199 | + | -224 |
| 166 | TTATGCACA | 5.9E-05 | 1293398 | - | -25 |
| 166 | TCGTACACA | 8.7E-04 | 1293448 | - | 25 |
| 166 | TTATCAACT | 4.9E-04 | 1293690 | + | 267 |
| 167 | TCTTCCACA | 3.4E-04 | 2752027 | + | -65 |
| 167 | TGATACACA | 8.7E-04 | 2752062 | - | -30 |
| 167 | ATATCCACC | 1.4E-03 | 2752143 | + | 51 |
| 167 | TTGTAAACA | 4.6E-04 | 2752162 | + | 70 |
| 167 | ATTTGCACA | 1.3E-03 | 2752196 | - | 104 |
| 168 | TTATTCTCA | 1.4E-03 | 2102967 | + | -87 |

| peak | sequence | p_value | start | strand | bp to peak |
|---|---|---|---|---|---|
| 168 | CGATTCACA | 1.4E-03 | 2103013 | - | -41 |
| 168 | TTTTCCACT | 8.7E-04 | 2103051 | + | -3 |
| 168 | TAATCCACA | 8.2E-05 | 2103072 | - | 18 |
| 168 | TCTTCCACA | 3.4E-04 | 2103246 | - | 192 |
| 169 | AAATCAACA | 1.1E-03 | 1585390 | + | 5 |
| 169 | CAATCCACA | 4.6E-04 | 1585408 | + | 23 |
| 170 | TCATCAACA | 1.9E-04 | 3203409 | + | -299 |
| 170 | TAGTACACA | 1.2E-03 | 3203500 | - | -208 |
| 170 | GTATAAACA | 1.3E-03 | 3203640 | - | -68 |
| 170 | TTATGCACA | 5.9E-05 | 3203715 | + | 7 |
| 170 | GTATGAACA | 1.3E-03 | 3203879 | + | 171 |
| 170 | TCATTCCCA | 1.5E-03 | 3203983 | + | 275 |
| 171 | TTATCCTCA | 6.2E-04 | 3403582 | - | -320 |
| 171 | GTTTCAACA | 1.2E-03 | 3403861 | - | -41 |
| 171 | TTATAAACA | 2.0E-04 | 3403891 | - | -11 |
| 171 | TCGTCAACA | 4.3E-04 | 3403933 | - | 31 |
| 171 | TTTTTCACA | 1.3E-04 | 3404109 | + | 207 |
| 172 | TTTTGCACA | 3.9E-04 | 1476926 | - | -335 |
| 172 | TTTTCAACA | 1.7E-04 | 1477129 | + | -132 |
| 172 | TTATTCTCA | 1.4E-03 | 1477173 | - | -88 |
| 172 | TTATCAACT | 4.9E-04 | 1477265 | - | 4 |
| 173 | TTATTCACT | 4.3E-04 | 4153486 | + | -23 |
| 173 | TTATCAATA | 1.2E-03 | 4153527 | + | 18 |
| 173 | TTATTCACT | 4.3E-04 | 4153710 | + | 201 |
| 173 | TTATTCCCA | 2.9E-04 | 4153768 | + | 259 |
| 173 | TAATAAACA | 1.5E-03 | 4153812 | - | 303 |
| 174 | AAATTCACA | 9.6E-04 | 3703433 | + | -268 |
| 174 | ATATTCACA | 9.3E-05 | 3703689 | + | -12 |
| 174 | ATGTCCACA | 9.3E-05 | 3703699 | - | -2 |
| 175 | TTATTCACA | 1.6E-05 | 3896458 | + | 5 |
| 175 | TTTACCACA | 9.6E-04 | 3896553 | + | 100 |
| 175 | CTTTTCACA | 6.2E-04 | 3896639 | + | 186 |
| 176 | TTGATCACA | 8.7E-04 | 1448917 | + | -206 |
| 176 | TTATGAACA | 1.9E-04 | 1449115 | + | -8 |
| 176 | AAATTCACA | 9.6E-04 | 1449132 | + | 9 |
| 176 | TTTTTCCCA | 1.4E-03 | 1449321 | - | 198 |
| 176 | TCTTCAACA | 9.6E-04 | 1449496 | - | 373 |
| 177 | TCGTCAACA | 4.3E-04 | 974617 | - | -330 |
| 177 | TTATACACA | 5.9E-05 | 974940 | - | -7 |
| 177 | TGATCAACA | 4.3E-04 | 974984 | - | 37 |
| 177 | ATTTGCACA | 1.3E-03 | 975234 | - | 287 |
| 177 | TTTTTCCCA | 1.4E-03 | 975294 | - | 347 |
| 178 | CTATAAACA | 9.6E-04 | 329365 | + | -393 |
| 178 | TTATGAACA | 1.9E-04 | 329660 | - | -98 |
| 178 | TATTTCACA | 1.1E-03 | 329722 | - | -36 |
| 178 | TTATTTACA | 1.3E-03 | 329796 | - | 38 |

| peak | sequence | p_value | start | strand | bp to peak |
|---|---|---|---|---|---|
| 178 | ATGACCACA | 1.5E-03 | 329883 | - | 125 |
| 178 | TTATTTACA | 1.3E-03 | 329895 | - | 137 |
| 179 | TTTTGAACA | 9.6E-04 | 1553712 | + | -63 |
| 179 | TCTTCCACA | 3.4E-04 | 1553769 | - | -6 |
| 180 | TCACTCACA | 1.4E-03 | 3826837 | + | -210 |
| 180 | ATGTCCACA | 9.3E-05 | 3826964 | + | -83 |
| 180 | TTAATCACA | 4.3E-04 | 3827052 | - | 5 |
| 180 | TTGTAAACA | 4.6E-04 | 3827147 | + | 100 |
| 180 | TTATGCACA | 5.9E-05 | 3827228 | - | 181 |
| 180 | TTATTCACG | 8.7E-04 | 3827436 | - | 389 |
| 181 | TCATAAACA | 1.1E-03 | 2135891 | + | -141 |
| 181 | TTATAAACA | 2.0E-04 | 2135999 | + | -33 |
| 181 | ATTTCAACA | 7.0E-04 | 2136020 | + | -12 |
| 181 | TCATAAACA | 1.1E-03 | 2136033 | + | 1 |
| 181 | GTATAAACA | 1.3E-03 | 2136164 | + | 132 |
| 181 | ATTTACACA | 1.4E-03 | 2136265 | + | 233 |
| 181 | GTATGAACA | 1.3E-03 | 2136366 | - | 334 |
| 182 | TTGTTCACA | 4.3E-05 | 3964981 | + | -334 |
| 182 | GCATCCACA | 4.9E-04 | 3965273 | - | -42 |
| 182 | TTATTCACC | 8.7E-04 | 3965299 | + | -16 |
| 182 | TTATCAACA | 2.3E-05 | 3965326 | + | 11 |
| 183 | TTTCTCACA | 1.3E-03 | 2349975 | - | -392 |
| 183 | TTATACACA | 5.9E-05 | 2350359 | + | -8 |
| 183 | TTATGCACT | 9.6E-04 | 2350516 | + | 149 |
| 184 | ATATCAACA | 1.3E-04 | 1501991 | - | -36 |
| 184 | GTATACACA | 5.2E-04 | 1502000 | - | -27 |
| 184 | GTATAAACA | 1.3E-03 | 1502099 | + | 72 |
| 184 | TTGTTCCCA | 6.2E-04 | 1502239 | - | 212 |
| 185 | CCGTTCACA | 1.4E-03 | 4041268 | - | -289 |
| 185 | CTGTCCCCA | 1.3E-03 | 4041478 | + | -79 |
| 185 | TTGTGCACA | 1.5E-04 | 4041553 | + | -4 |
| 185 | TCGTTCACA | 3.4E-04 | 4041605 | - | 48 |
| 185 | TAATGAACA | 1.4E-03 | 4041760 | - | 203 |
| 185 | CTATGCACA | 3.4E-04 | 4041878 | + | 321 |
| 186 | TTTTCAACA | 1.7E-04 | 1532773 | + | -49 |
| 186 | CTATCAACA | 1.5E-04 | 1532800 | - | -22 |
| 186 | TTATGCACA | 5.9E-05 | 1532922 | + | 100 |
| 187 | TTATTAACA | 7.3E-05 | 2131091 | - | -158 |
| 187 | TTTCCAACA | 1.5E-03 | 2131149 | + | -100 |
| 187 | TAATTCACA | 2.1E-04 | 2131278 | + | 29 |
| 187 | TTTTTAACA | 4.3E-04 | 2131350 | - | 101 |
| 188 | CTGTTCACA | 2.6E-04 | 662555 | + | -367 |
| 188 | TTGTTAACA | 1.7E-04 | 662916 | - | -6 |
| 188 | TCTTCAACA | 9.6E-04 | 662939 | - | 17 |
| 188 | CCGTTCACA | 1.4E-03 | 663027 | - | 105 |
| 189 | TTTTCCACA | 4.3E-05 | 3433037 | + | -7 |

| peak | sequence | p_value | start | strand | bp to peak |
|---|---|---|---|---|---|
| 189 | TCGTTAACA | 9.6E-04 | 3433154 | + | 110 |
| 190 | AGATCAACA | 1.4E-03 | 2225860 | + | -37 |
| 190 | TTATCAACA | 2.3E-05 | 2225908 | - | 11 |
| 191 | TTATCGACA | 3.9E-04 | 3552100 | - | -239 |
| 191 | GTATACACA | 5.2E-04 | 3552121 | - | -218 |
| 191 | CTATTCACT | 1.5E-03 | 3552197 | + | -142 |
| 191 | TTATTAACA | 7.3E-05 | 3552351 | - | 12 |
| 191 | CTATAAACA | 9.6E-04 | 3552373 | - | 34 |
| 191 | ATATCAACA | 1.3E-04 | 3552709 | - | 370 |
| 192 | TAATCAACA | 2.6E-04 | 2238719 | + | -5 |
| 193 | TTTTCAACA | 1.7E-04 | 944651 | - | -34 |
| 194 | CTGTCCACA | 1.1E-04 | 3282448 | - | 5 |
| 195 | TTATCAACT | 4.9E-04 | 1293690 | + | -302 |
| 195 | TTATGCACA | 5.9E-05 | 1293985 | + | -7 |
| 195 | ATTTACACA | 1.4E-03 | 1294015 | + | 23 |
| 195 | TAACCCACA | 1.0E-03 | 1294164 | - | 172 |
| 196 | AGATTCACA | 1.2E-03 | 2251869 | - | -297 |
| 196 | TTTTGAACA | 9.6E-04 | 2251965 | + | -201 |
| 196 | AAATCAACA | 1.1E-03 | 2251986 | - | -180 |
| 196 | TTATTAACA | 7.3E-05 | 2252163 | + | -3 |
| 196 | TTATAAACA | 2.0E-04 | 2252220 | + | 54 |
| 196 | TTGCTCACA | 6.2E-04 | 2252301 | + | 135 |
| 197 | TTTTTCCCA | 1.4E-03 | 1326847 | - | -106 |
| 197 | CTGCCCACA | 1.2E-03 | 1326884 | + | -69 |
| 197 | TTATCCACA | 5.3E-06 | 1326948 | - | -5 |
| 197 | TAATGAACA | 1.4E-03 | 1326984 | + | 31 |
| 197 | TTTTCGACA | 1.5E-03 | 1327179 | - | 226 |
| 197 | TGTTTCACA | 1.4E-03 | 1327188 | - | 235 |
| 198 | CCGTTCACA | 1.4E-03 | 3694078 | - | -367 |
| 198 | TTGTCCTCA | 1.3E-03 | 3694160 | - | -285 |
| 198 | TTGTCCACA | 9.3E-06 | 3694439 | - | -6 |
| 198 | TTATCGACA | 3.9E-04 | 3694498 | + | 53 |
| 198 | TTATTTACA | 1.3E-03 | 3694576 | - | 131 |
| 199 | GCATCAACA | 1.3E-03 | 3385806 | - | -350 |
| 199 | TTATCAACA | 2.3E-05 | 3386162 | + | 6 |
| 199 | TTATCCACT | 1.7E-04 | 3386186 | + | 30 |
| 200 | ATATCAACA | 1.3E-04 | 4001344 | + | -13 |
| 200 | ATATTCACT | 1.4E-03 | 4001357 | + | 0 |
| 200 | TTTCCAACA | 1.5E-03 | 4001410 | + | 53 |
| 200 | TTTCCAACA | 1.5E-03 | 4001704 | + | 347 |
| 201 | GCATTCACA | 1.1E-03 | 2190181 | - | -240 |
| 201 | TAATACACA | 5.7E-04 | 2190343 | - | -78 |
| 201 | TTGCTCACA | 6.2E-04 | 2190556 | - | 135 |
| 201 | TTTTGAACA | 9.6E-04 | 2190591 | + | 170 |
| 202 | TTTTTCACA | 1.3E-04 | 1885202 | + | -347 |
| 202 | TCATTAACA | 4.6E-04 | 1885314 | - | -235 |

| peak | sequence | p_value | start | strand | bp to peak |
|---|---|---|---|---|---|
| 202 | GTATACACA | 5.2E-04 | 1885470 | + | -79 |
| 202 | TTATCAACG | 1.0E-03 | 1885545 | + | -4 |
| 202 | TTAAACACA | 1.0E-03 | 1885645 | + | 96 |
| 202 | TTATCAATA | 1.2E-03 | 1885812 | + | 263 |
| 203 | TAAACCACA | 1.3E-03 | 1970674 | + | -202 |
| 203 | TTATACACT | 9.6E-04 | 1970979 | + | 103 |
| 203 | TGATGCACA | 8.1E-04 | 1971014 | - | 138 |
| 204 | TAATGAACA | 1.4E-03 | 2576599 | - | -347 |
| 204 | ACATCCACA | 2.6E-04 | 2576679 | - | -267 |
| 204 | TTTTCGACA | 1.5E-03 | 2576697 | - | -249 |
| 204 | ACATCAACA | 8.1E-04 | 2576951 | + | 5 |
| 204 | CGATCCACA | 6.2E-04 | 2576975 | + | 29 |
| 204 | ATATCAACA | 1.3E-04 | 2577018 | - | 72 |
| 205 | TTATAAACA | 2.0E-04 | 513884 | - | -387 |
| 205 | TATTTCACA | 1.1E-03 | 513930 | + | -341 |
| 205 | CTATTCACA | 1.1E-04 | 514192 | - | -79 |
| 205 | CAATTCACA | 1.0E-03 | 514399 | + | 128 |
| 205 | ACATCAACA | 8.1E-04 | 514471 | - | 200 |
| 206 | TTTTAAACA | 1.0E-03 | 1251877 | - | -271 |
| 206 | ATTTTCACA | 5.7E-04 | 1252044 | - | -104 |
| 206 | TCATTCACA | 1.5E-04 | 1252128 | - | -20 |
| 206 | ATTTCAACA | 7.0E-04 | 1252523 | + | 375 |
| 207 | ATATCCACA | 2.8E-05 | 3104291 | - | -7 |
| 207 | ATATCAACA | 1.3E-04 | 3104315 | - | 17 |
| 208 | TTCTTAACA | 1.5E-03 | 2241302 | - | -314 |
| 208 | TTATCAACT | 4.9E-04 | 2241516 | + | -100 |
| 208 | TTAATCACA | 4.3E-04 | 2241611 | - | -5 |
| 208 | AAATTCACA | 9.6E-04 | 2241836 | - | 220 |
| 209 | TCGTTCACA | 3.4E-04 | 1413887 | - | -284 |
| 209 | TTGTAAACA | 4.6E-04 | 1414148 | - | -23 |
| 209 | TAATGCACA | 5.2E-04 | 1414175 | + | 4 |
| 210 | CAATCAACA | 1.2E-03 | 592260 | - | -126 |
| 210 | ATTTTAACA | 1.5E-03 | 592312 | - | -74 |
| 210 | CTATACACA | 3.4E-04 | 592332 | - | -54 |
| 210 | ATGTCAACA | 2.9E-04 | 592394 | - | 8 |
| 210 | ACTTCCACA | 1.3E-03 | 592519 | - | 133 |
| 210 | TTTTCAACA | 1.7E-04 | 592608 | - | 222 |
| 210 | TTATTAACA | 7.3E-05 | 592770 | + | 384 |
| 211 | TTATTCACG | 8.7E-04 | 2148135 | - | -216 |
| 211 | GTATACACA | 5.2E-04 | 2148209 | - | -142 |
| 211 | TTATCAACA | 2.3E-05 | 2148407 | + | 56 |
| 211 | ATAATCACA | 1.5E-03 | 2148435 | - | 84 |
| 212 | TTGACAACA | 1.0E-03 | 3959869 | + | -97 |
| 212 | TTGTCCACA | 9.3E-06 | 3959991 | - | 25 |
| 212 | TATTCAACA | 1.3E-03 | 3960173 | + | 207 |
| 213 | TTTTAAACA | 1.0E-03 | 2465624 | + | -164 |

| peak | sequence | p_value | start | strand | bp to peak |
|---|---|---|---|---|---|
| 213 | TTATTCATA | 1.0E-03 | 2465760 | + | -28 |
| 213 | TTATGCACA | 5.9E-05 | 2465782 | + | -6 |
| 213 | TTCTTCACA | 5.7E-04 | 2465836 | + | 48 |
| 213 | TTATCCACC | 3.9E-04 | 2465969 | - | 181 |
| 214 | TTTTCCACA | 4.3E-05 | 2197745 | - | -20 |
| 214 | CTTTCCACA | 2.9E-04 | 2197796 | - | 31 |
| 214 | ATTTCCACA | 2.3E-04 | 2198003 | + | 238 |
| 214 | TTATTCATA | 1.0E-03 | 2198104 | - | 339 |
| 215 | TTATCAATA | 1.2E-03 | 515207 | + | -27 |
| 215 | TTTTGAACA | 9.6E-04 | 515226 | - | -8 |
| 215 | TAATTCACA | 2.1E-04 | 515295 | + | 61 |
| 215 | TTATAAACA | 2.0E-04 | 515531 | + | 297 |
| 216 | TGATTAACA | 8.7E-04 | 2693602 | + | 16 |
| 216 | TTGTCCCCA | 2.9E-04 | 2693620 | - | 34 |
| 216 | TTATTAACT | 1.0E-03 | 2693629 | - | 43 |
| 216 | TGGTCCACA | 2.9E-04 | 2693855 | - | 269 |
| 216 | TTGTCTACA | 1.3E-03 | 2693890 | - | 304 |
| 217 | GCATCAACA | 1.3E-03 | 3957189 | - | -60 |
| 217 | TAATTCACA | 2.1E-04 | 3957305 | + | 56 |
| 217 | TATTTCACA | 1.1E-03 | 3957340 | + | 91 |
| 217 | ACATCAACA | 8.1E-04 | 3957560 | + | 311 |
| 218 | CAATCAACA | 1.2E-03 | 1248015 | - | -68 |
| 218 | TAATAAACA | 1.5E-03 | 1248031 | - | -52 |
| 218 | TTTTCAACA | 1.7E-04 | 1248094 | + | 11 |
| 218 | TTGTCCACT | 3.9E-04 | 1248208 | + | 125 |
| 218 | TTTTGAACA | 9.6E-04 | 1248298 | - | 215 |
| 218 | GTGTCAACA | 5.2E-04 | 1248417 | - | 334 |
| 218 | TTGTCACCA | 8.1E-04 | 1248429 | - | 346 |
| 219 | ATATCCACA | 2.8E-05 | 1854429 | - | 4 |
| 219 | GTATGCACA | 4.9E-04 | 1854534 | + | 109 |
| 220 | ATTTTAACA | 1.5E-03 | 2994139 | + | -213 |
| 220 | CCGTTCACA | 1.4E-03 | 2994193 | + | -159 |
| 220 | GTATTCACA | 2.0E-04 | 2994207 | - | -145 |
| 220 | GTATCAACA | 2.3E-04 | 2994358 | + | 6 |
| 220 | ATATCAACA | 1.3E-04 | 2994397 | + | 45 |
| 220 | TTATCAATA | 1.2E-03 | 2994474 | - | 122 |
| 221 | TTATCCACG | 3.9E-04 | 1884306 | - | -15 |
| 221 | TTTACCACA | 9.6E-04 | 1884411 | + | 90 |
| 221 | TAATGAACA | 1.4E-03 | 1884548 | - | 227 |
| 222 | ATATTCACT | 1.4E-03 | 1462402 | - | -43 |
| 222 | TCATAAACA | 1.1E-03 | 1462500 | - | 55 |
| 222 | TTATTAACT | 1.0E-03 | 1462703 | + | 258 |
| 223 | TTTTAAACA | 1.0E-03 | 2185441 | - | -377 |
| 223 | TTTTAAACA | 1.0E-03 | 2185529 | - | -289 |
| 223 | ATATGCACA | 2.9E-04 | 2185662 | - | -156 |
| 223 | CAATCAACA | 1.2E-03 | 2185698 | - | -120 |

| peak | sequence | p_value | start | strand | bp to peak |
|---|---|---|---|---|---|
| 223 | TAATCAACA | 2.6E-04 | 2185957 | + | 139 |
| 223 | TTAGCCACA | 8.7E-04 | 2186048 | + | 230 |
| 223 | GTATAAACA | 1.3E-03 | 2186206 | + | 388 |
| 224 | TTATGAACA | 1.9E-04 | 2250827 | + | -97 |
| 224 | TTGTTAACA | 1.7E-04 | 2250931 | - | 7 |
| 225 | ATATCACCA | 1.4E-03 | 1200695 | + | -136 |
| 225 | TTGTCCACA | 9.3E-06 | 1200837 | - | 6 |
| 225 | TGACCCACA | 1.3E-03 | 1200893 | - | 62 |
| 225 | GTGTCAACA | 5.2E-04 | 1201161 | - | 330 |
| 226 | TAATGCACA | 5.2E-04 | 1464723 | + | -390 |
| 226 | TTATTTACA | 1.3E-03 | 1464798 | - | -315 |
| 226 | TTATTCAGA | 1.4E-03 | 1464994 | - | -119 |
| 226 | TTTTTAACA | 4.3E-04 | 1465108 | - | -5 |
| 226 | TTTTCCCCA | 6.2E-04 | 1465185 | + | 72 |
| 227 | GTATTAACA | 5.2E-04 | 1704697 | - | 48 |
| 227 | TAATCAACA | 2.6E-04 | 1704706 | - | 57 |
| 227 | TTTTCAACA | 1.7E-04 | 1705014 | + | 365 |
| 228 | TTATTCATA | 1.0E-03 | 3445357 | + | -131 |
| 229 | TTATTCACA | 1.6E-05 | 1316949 | + | -44 |
| 229 | ACGTTCACA | 1.3E-03 | 1317012 | + | 19 |
| 229 | TTATTCACA | 1.6E-05 | 1317074 | - | 81 |
| 230 | ATATAAACA | 8.7E-04 | 937924 | + | -358 |
| 230 | TTATAAACA | 2.0E-04 | 937993 | - | -289 |
| 230 | TTGTTAACA | 1.7E-04 | 938280 | - | -2 |
| 231 | TGATTCACA | 3.4E-04 | 2926142 | + | -171 |
| 231 | ATGCCCACA | 1.1E-03 | 2926187 | - | -126 |
| 231 | TTCTACACA | 1.4E-03 | 2926325 | + | 12 |
| 231 | TCATCCCCA | 7.0E-04 | 2926428 | + | 115 |
| 231 | TTTTTCACA | 1.3E-04 | 2926588 | + | 275 |
| 232 | TTATGCACA | 5.9E-05 | 2698680 | + | 71 |
| 232 | AAATCAACA | 1.1E-03 | 2698956 | - | 347 |
| 233 | TTATTCACA | 1.6E-05 | 1427359 | - | 4 |
| 233 | ATAACCACA | 8.1E-04 | 1427473 | + | 118 |
| 233 | ATTTTCACA | 5.7E-04 | 1427649 | - | 294 |
| 234 | ATTTACACA | 1.4E-03 | 29907 | - | -319 |
| 234 | CTGTCAACA | 3.4E-04 | 30131 | - | -95 |
| 235 | TCATTCACA | 1.5E-04 | 2367586 | + | -370 |
| 235 | ATTTCAACA | 7.0E-04 | 2367706 | + | -250 |
| 235 | TCTTCAACA | 9.6E-04 | 2367883 | + | -73 |
| 235 | TTGTACACA | 1.5E-04 | 2367935 | - | -21 |
| 235 | ATATCAACA | 1.3E-04 | 2367961 | + | 5 |
| 235 | CTTTCAACA | 8.1E-04 | 2368066 | + | 110 |
| 235 | GCATCAACA | 1.3E-03 | 2368318 | + | 362 |
| 236 | ATATCAACA | 1.3E-04 | 2215322 | + | -22 |
| 236 | TGATCAACA | 4.3E-04 | 2215544 | + | 200 |
| 236 | TTATTCATA | 1.0E-03 | 2215567 | + | 223 |

| peak | sequence | p_value | start | strand | bp to peak |
|---|---|---|---|---|---|
| 237 | TCACCCACA | 7.0E-04 | 451034 | + | -31 |
| 237 | TTATCCACT | 1.7E-04 | 451095 | - | 30 |
| 238 | ATATGCACA | 2.9E-04 | 958674 | - | -4 |
| 239 | TTATCCTCA | 6.2E-04 | 2154697 | - | -250 |
| 239 | CTATTCCCA | 1.3E-03 | 2154829 | + | -118 |
| 239 | TTATCCCCA | 1.3E-04 | 2154949 | + | 2 |
| 239 | TGTTTCACA | 1.4E-03 | 2155233 | - | 286 |
| 239 | TTATCCGCA | 9.6E-04 | 2155247 | + | 300 |
| 239 | TTATCCAGA | 7.0E-04 | 2155342 | - | 395 |
| 240 | TTGTTCACT | 8.7E-04 | 1203248 | + | 10 |
| 240 | GTTTCCACA | 4.6E-04 | 1203313 | - | 75 |
| 241 | GTATGAACA | 1.3E-03 | 2242203 | + | -372 |
| 241 | TAATCCACT | 1.3E-03 | 2242397 | + | -178 |
| 241 | TTATGAACA | 1.9E-04 | 2242572 | - | -3 |
| 242 | TTATTCACG | 8.7E-04 | 2664602 | + | -304 |
| 242 | TTATCCACT | 1.7E-04 | 2664632 | - | -274 |
| 242 | CAATCCACA | 4.6E-04 | 2664889 | - | -17 |
| 242 | CTATAAACA | 9.6E-04 | 2664955 | + | 49 |
| 242 | TATTCAACA | 1.3E-03 | 2665140 | + | 234 |
| 243 | TTATTGACA | 8.1E-04 | 2636781 | - | -350 |
| 243 | TCCTCCACA | 1.3E-03 | 2636795 | + | -336 |
| 243 | TTATTTACA | 1.3E-03 | 2637198 | + | 67 |
| 244 | TTTTGAACA | 9.6E-04 | 805557 | + | -306 |
| 244 | TAATCCCCA | 1.0E-03 | 805793 | - | -70 |
| 244 | TTTCTCACA | 1.3E-03 | 806067 | - | 204 |
| 245 | GTATCAACA | 2.3E-04 | 1840225 | + | -137 |
| 245 | TTATTCACA | 1.6E-05 | 1840394 | - | 32 |
| 245 | TTATACACT | 9.6E-04 | 1840433 | + | 71 |
| 246 | TTGCTCACA | 6.2E-04 | 2829525 | - | -184 |
| 246 | TTATCCACA | 5.3E-06 | 2829713 | + | 4 |
| 246 | ACATCCACA | 2.6E-04 | 2829737 | + | 28 |
| 246 | TCAACCACA | 1.0E-03 | 2829830 | + | 121 |
| 247 | CTATCAACA | 1.5E-04 | 3126161 | - | -340 |
| 247 | TTATACCCA | 8.1E-04 | 3126302 | + | -199 |
| 247 | TCATCCACA | 5.9E-05 | 3126472 | + | -29 |
| 247 | ATATGAACA | 8.1E-04 | 3126499 | - | -2 |
| 247 | TTATTGACA | 8.1E-04 | 3126531 | - | 30 |
| 247 | CTGTCAACA | 3.4E-04 | 3126582 | - | 81 |
| 248 | TTCTTAACA | 1.5E-03 | 1657089 | - | -25 |
| 248 | ATATTAACA | 3.4E-04 | 1657125 | - | 11 |
| 248 | ATTTCAACA | 7.0E-04 | 1657141 | - | 27 |
| 248 | TGATCAACA | 4.3E-04 | 1657448 | + | 334 |
| 248 | TTGTCCACA | 9.3E-06 | 1657471 | + | 357 |
| 248 | TGATCAACA | 4.3E-04 | 1657493 | - | 379 |
| 249 | GTATTCACA | 2.0E-04 | 2126311 | - | -8 |
| 250 | TTATCCCCA | 1.3E-04 | 639090 | - | -47 |

| peak | sequence | p_value | start | strand | bp to peak |
|---|---|---|---|---|---|
| 250 | TCGTACACA | 8.7E-04 | 639145 | - | 8 |
| 250 | TCTTCAACA | 9.6E-04 | 639232 | - | 95 |
| 250 | TGTTTCACA | 1.4E-03 | 639526 | + | 389 |
| 251 | ACATCCACA | 2.6E-04 | 507938 | - | -2 |
| 251 | TTCTCCACA | 2.6E-04 | 508096 | - | 156 |
| 251 | TGGTCAACA | 8.7E-04 | 508125 | + | 185 |
| 251 | TGATACACA | 8.7E-04 | 508195 | - | 255 |
| 252 | TCTTCCACA | 3.4E-04 | 3087587 | - | -336 |
| 252 | TTTTAAACA | 1.0E-03 | 3087727 | - | -196 |
| 252 | TTTTCAACA | 1.7E-04 | 3087868 | - | -55 |
| 252 | TGTTTCACA | 1.4E-03 | 3087907 | + | -16 |
| 252 | TCATCCACA | 5.9E-05 | 3087991 | + | 68 |
| 252 | TTAATAACA | 1.1E-03 | 3088102 | + | 179 |
| 252 | ATGTCAACA | 2.9E-04 | 3088282 | + | 359 |
| 253 | TATTCAACA | 1.3E-03 | 4163764 | + | -220 |
| 253 | ATTTCAACA | 7.0E-04 | 4163857 | + | -127 |
| 253 | TCGCCCACA | 1.4E-03 | 4163968 | + | -16 |
| 253 | TAGTCAACA | 5.7E-04 | 4163981 | - | -3 |
| 253 | TTATTCACT | 4.3E-04 | 4164103 | - | 119 |
| 253 | TTGTTAACA | 1.7E-04 | 4164127 | - | 143 |
| 254 | TCATAAACA | 1.1E-03 | 2278993 | - | -300 |
| 254 | TAATCAACA | 2.6E-04 | 2279256 | - | -37 |
| 254 | TTATGAACA | 1.9E-04 | 2279356 | + | 63 |
| 254 | TTGTCAACT | 1.0E-03 | 2279506 | - | 213 |
| 255 | ATATTCCCA | 1.2E-03 | 2243801 | + | -217 |
| 255 | ATGTGCACA | 6.2E-04 | 2243988 | - | -30 |
| 255 | CTCTCCACA | 1.1E-03 | 2244008 | + | -10 |
| 255 | AAATCCACA | 4.3E-04 | 2244172 | - | 154 |
| 255 | ATATCGACA | 1.3E-03 | 2244376 | + | 358 |
| 255 | TTACTCACA | 2.9E-04 | 2244409 | - | 391 |
| 256 | TTACCCCCA | 1.3E-03 | 2084384 | + | -89 |
| 256 | TTGTTCACA | 4.3E-05 | 2084517 | + | 44 |
| 256 | TGATCAACA | 4.3E-04 | 2084663 | + | 190 |
| 257 | GAATTCACA | 1.5E-03 | 2664325 | + | -216 |
| 257 | TAATAAACA | 1.5E-03 | 2664446 | + | -95 |
| 257 | TTATTCACG | 8.7E-04 | 2664602 | + | 61 |
| 257 | TTATCCACT | 1.7E-04 | 2664632 | - | 91 |
| 257 | CAATCCACA | 4.6E-04 | 2664889 | - | 348 |
| 258 | TCTTTCACA | 8.1E-04 | 2068617 | + | -337 |
| 258 | ATATTCACT | 1.4E-03 | 2068636 | + | -318 |
| 258 | TAATTAACA | 6.2E-04 | 2068852 | + | -102 |
| 258 | TTATTCACT | 4.3E-04 | 2068949 | - | -5 |
| 258 | ATATACACA | 2.9E-04 | 2069066 | - | 112 |
| 258 | TTGCACACA | 1.5E-03 | 2069213 | - | 259 |
| 259 | TCATCCACT | 9.6E-04 | 2188970 | + | -102 |
| 259 | TTAATAACA | 1.1E-03 | 2188999 | - | -73 |

| peak | sequence | p_value | start | strand | bp to peak |
|---|---|---|---|---|---|
| 259 | CCATGCACA | 1.5E-03 | 2189129 | + | 57 |
| 259 | CTATTCACT | 1.5E-03 | 2189186 | + | 114 |
| 260 | TTATGCACA | 5.9E-05 | 3701569 | + | -120 |
| 260 | TTATGCACA | 5.9E-05 | 3701689 | + | 0 |
| 260 | TTTTCCCCA | 6.2E-04 | 3701836 | + | 147 |
| 261 | TCATCCACA | 5.9E-05 | 353931 | - | -4 |
| 261 | TCACCCACA | 7.0E-04 | 354150 | - | 215 |
| 261 | TTTTCCACT | 8.7E-04 | 354221 | + | 286 |
| 262 | TTATGAACA | 1.9E-04 | 2921278 | - | -90 |
| 262 | CTATCCACA | 3.2E-05 | 2921374 | + | 6 |
| 263 | TGTTCCACA | 7.0E-04 | 2144929 | - | -345 |
| 263 | CTATGAACA | 8.7E-04 | 2144987 | - | -287 |
| 263 | TTCTTAACA | 1.5E-03 | 2145008 | + | -266 |
| 263 | TTGTTAACA | 1.7E-04 | 2145282 | - | 8 |
| 264 | CTTTCAACA | 8.1E-04 | 511026 | + | -271 |
| 264 | CTGTTCACA | 2.6E-04 | 511137 | - | -160 |
| 264 | ATATCAACA | 1.3E-04 | 511283 | - | -14 |
| 264 | ATATGCACA | 2.9E-04 | 511313 | - | 16 |
| 265 | CTGTCCCCA | 1.3E-03 | 236843 | - | -240 |
| 265 | TTGTCCACA | 9.3E-06 | 237087 | - | 4 |
| 265 | TTCTTAACA | 1.5E-03 | 237151 | + | 68 |
| 266 | TTGTAAACA | 4.6E-04 | 3377192 | + | -169 |
| 266 | CTATCCACA | 3.2E-05 | 3377362 | + | 1 |
| 266 | TTATCCAGA | 7.0E-04 | 3377375 | - | 14 |
| 267 | TTATTCATA | 1.0E-03 | 1542498 | + | -111 |
| 267 | TTCTTAACA | 1.5E-03 | 1542518 | + | -91 |
| 267 | TTGTTCACA | 4.3E-05 | 1542596 | + | -13 |
| 268 | TAATTCACA | 2.1E-04 | 2252657 | + | -301 |
| 268 | TTTTCCACA | 4.3E-05 | 2252864 | - | -94 |
| 268 | CCATCAACA | 8.7E-04 | 2252969 | - | 11 |
| 268 | TGGTCAACA | 8.7E-04 | 2253112 | + | 154 |
| 268 | CCATCCACA | 3.4E-04 | 2253194 | - | 236 |
| 269 | ACTTCCACA | 1.3E-03 | 1890706 | - | -223 |
| 269 | TAATTAACA | 6.2E-04 | 1890782 | - | -147 |
| 269 | ATATCAACA | 1.3E-04 | 1890936 | + | 7 |
