# Supplementary material for: In Vitro Whole Genome DNA Binding Analysis of the Bacterial Replication Initiator and Transcription Factor DnaA
Source: PLoS Genet. 2015 May 28;11(5):e1005258. doi: 10.1371/journal.pgen.1005258 (PMC4447404; doi:10.1371/journal.pgen.1005258)
Supplement: S5 Table — (PDF) [file pgen.1005258.s011.pdf]

Table S5. Primers used for qPCR.

genomic locus	primer name	primer sequence
nicK	clo280	5'-TGGCTACGTTGGCACGTATG
nicK	clo281	5'-AATTGACGGCAACCTTGACC
iscS	oJLS018	5'-TTTATGAAAGCGGTACAAG
iscS	oJLS019	5'-ATGACGAAACGAACAAATC
yuzB	oJLS007	5'-TTCAGGCTAAATTGTAGG
yuzB	oJLS008	5'-CCTTTAATCCGAAGAGTATG
sboA	psboA1	5'-CCTCATAAAAAGCATTTCCT
sboA	psboA2	5'-AATTGAATCCTCCCTTTTTT
yjcM	oJLS016	5'-ATGGTGACAATGATAGAC
yjcM	oJLS017	5'-AATAAGTGCTGCCAAGAG
cotH	oJLS070	5'-CCGCTTCTGTATAATGCGTAGT
cotH	oJLS071	5'-AAACACGTGGATGTCGATAAGT
ypfD	oJLS072	5'-GAGTGGGAAAGCTGTGAAATG
ypfD	oJLS073	5'-GGAAGTGTACTTGATGGCAAAG
ydiO	oJLS074	5'-TAGTGGATGTGGAGGTCTATCC
ydiO	oJLS075	5'-AGGCAGCTTCGTTAAGATCAC
yphF	oJLS076	5'- AGCCCTCCATTTGCCTTT
yphF	oJLS077	5'- GTGTGCATGCCATTCCTTATC
rplB	oJLS003	5'- TCATGTCAGGTCCTGAAG
rplB	oJLS004	5'-TACCAGCTGAACGTACAAGCTGTC
dnaA	HM126	5'-TATTTGTGTTTTAACTCTTGATTACTAATC
dnaA	HM127	5'- TTGAAAACATATAATATAGTAGATAAATAG
