# Supplementary material for: In Vitro Whole Genome DNA Binding Analysis of the Bacterial Replication Initiator and Transcription Factor DnaA
Source: PLoS Genet. 2015 May 28;11(5):e1005258. doi: 10.1371/journal.pgen.1005258 (PMC4447404; doi:10.1371/journal.pgen.1005258)
Supplement: S1 Text — (PDF) [file pgen.1005258.s012.pdf]

S1 Text. Supporting Information

Materials and Methods

Purification of hexa-histidine-tagged DnaA

B. subtilis DnaA with a C-terminal hexa-histidine tag (DnaA-his) was expressed from plasmid pBB84, a derivative of pET28b (Novagen) that adds the amino acids AAALEHHHHHH to the C-terminus of DnaA. *E. coli* BL21(DE3) pLysS (Novagen) cells containing pBB84 were grown at 30°C to an OD_{600} of 0.4, IPTG (0.4 mM) was added, and growth was continued for three hours. Cells from a 500 ml culture were harvested by centrifugation (10 min at 8300 x g), resuspended in 10 ml Talon Buffer (50 mM $NaPO_4$, pH 7.0, 5 mM imidizole, 300 mM NaCl) and frozen at -80°C. The cell suspension lysed upon thawing. Protease inhibitors (Sigma P8849 or AEBSF), $MgCl_2$ (to 10 mM), and DNase I (to 5 µg/ml) were added, and the mixture was stirred at room-temperature until the viscosity decreased (10 min). All subsequent purification steps were carried out at 4˚C. The lysate was clarified at 35,000 x g for 20 min, and loaded onto two tandem 1 ml Talon columns (Clontech). The columns were washed with 5 column volumes of Talon Buffer, followed by a 20 ml gradient to the same buffer containing 150 mM imidazole.

Fractions containing DnaA-his were mixed with 2 volumes of buffer (45 mM HEPES-KOH, pH 7.6, 0.75 mM EDTA, 15 mM magnesium acetate, 1.5 mM dithiothreitol, and 5% sucrose), and loaded onto a 5 ml HiTrap Q FF column (GE Life Sciences) preequilibrated with buffer Q (45 mM HEPES-KOH, pH 7.6, 0.5 mM EDTA, 10 mM magnesium acetate, 100 mM potassium glutamate, 1 mM DTT, 5% sucrose). The column was washed with 25 ml Buffer Q, then eluted with a 50 ml linear gradient to the

same buffer containing 1 M potassium glutamate. Fractions containing DnaA-his were concentrated using an Amicon Ultracel 50K filter unit to a volume of 2.5 ml. The buffer was exchanged to DnaA Storage Buffer (45 mM HEPES-KOH, pH 7.6, 0.5 mM EDTA, 10 mM magnesium acetate, 700 mM potassium glutamate, 1 mM DTT, 20% sucrose) using a PD10 column (GE Healthcare). The preparation was further concentrated using an Amicon Ultracel 50K filter unit (Millipore), aliquoted, and stored at -80°C. The final yield was 4.8 mg, as determined by A_{205} (31 for 1 mg/ml), or by an experimentally determined A_{280} extinction coefficient (0.627 for 1 mg/ml). The preparation was 98% pure, as assayed by quantitation of a Coomassie stained gel using an Odyssey infrared scanner (Licor).

ATPase assays

ATPase activity during binding reactions was determined using a malachite green assay, based on the method described previously [24]. A malachite green assay solution was prepared by mixing 0.0812% (w/v) malachite green, 2.23% (w/v) polyvinyl alcohol (average mol wt 30,000-70,000), 46.3 mM ammonium molybdate in 6N HCl, and water in a ratio of 2:1:1:2. The solution was rotated for at least 2 h prior to use, and was then stable for several days. Binding reactions containing 4.1 µM DnaA and 2.5 mM ATP were incubated at 21°C, as described in the Methods, except that the incubation was allowed to proceed for five hours. At 1 hour time intervals, 10 µl aliquots were removed and mixed with 40 µl malachite green assay solution, followed by 5 µl 1.16M sodium citrate. After 10-50 minutes, absorbance at 630 nm was measured using a NanoDrop. A standard curve was prepared using 0-125 µM potassium phosphate.

ATPase activity was linear for the duration of the assay period, and the rate was determined by fitting the data to a line.

Preparation of sheared genomic DNA from *B. subtilis* strain AG1839

Genomic DNA was purified from a *dnaBts* mutant of *B. subtilis*, strain AG1839 (a.k.a., KPL69; genotype: *trp, phe, dnaB134*(Ts)-*zhb83*::Tn*917*) [29, 44]. At non-permissive temperature, the *dnaB134* mutation causes a block in replication initiation, but allows completion of rounds of replication that are already underway [43], resulting in cells with uniform amounts of DNA over the whole chromosome. Cells were grown in LB at permissive temperature (30°C) to an OD_{600} of 0.8 – 1, then rapidly shifted to non-permissive temperature by addition of a one-half volume of LB preheated to 83°C. After one hour of growth at 48°C, the cells were rapidly heated to 60-65°C for 5 minutes to kill the cells without allowing them to cool. Cells were harvested by cetrifugation (5000 x g; 15 min), and the cell pellets were frozen at -80°C.

DNA was purified using Genomic-tip 500/G columns and buffers (Qiagen) as described in the accompanying literature, with the following modifications. For each Genomic-tip 500/G column, 400 OD_{600} units of cells were resuspended in 52 ml B1 buffer, 104 µl RNase A (100 mg/ml; Qiagen), 375 µl Proteinase K (>600 mAU/ml; Qiagen), and 600 µl lysozyme (100 mg/ml), and after incubation, 18.8 ml B2 buffer was added. The final DNA yield was ~500 µg.

The purified DNA was dissolved in TE to a concentration of ~0.5 mg/ml, and was sheared in 1 ml glass tubes using a Covaris S2 with the following settings: duty cycle 10%, intensity 10, cycles per burst 200, 240 sec. The resulting fragments were mostly in the 200-300 bp range. The sequencing coverage (normalized to 1) obtained with this

DNA was 1 ± 0.14 (mean ± SD), with a minimum of 0.40 and a maximum of 2.9. Most of the regions that deviated significantly from the mean corresponded to rRNA genes (ten copies in the *B. subtilis* genome), which are difficult to map with short sequence reads and thus subject to mapping artifacts. In addition, four non-ribosomal regions had ~2-fold more reads than average. The basis for additional sequence reads at these loci is not known, but did not result in artifactual peaks of DnaA binding.

Refinement of catalog of DnaA-binding regions

Regions bound by 1.4 μM ATP-DnaA-his were identified using cisGenome v 2.0 [46], with default settings (except -b 5 -w 1 -c 2 -e 250), and a false discover rate cutoff of 0.15. The preliminary catalog of 275 binding regions was further refined as follows. All peaks were inspected in the genome browser MochiView [48]. Several regions were identified that were called as a single peak, but were actually two adjacent peaks. Three of these doublet peaks were split into two separate peaks, because both daughter peaks were within the range of amplitudes present in the peak collection. Peaks were also visually compared at 1.4 and 4.1 μM, and compared to a cisGenome analysis of binding at 4.1 μM DnaA (see below). Based on this, four weak-binding regions were eliminated based on the fact that they did not show significantly more binding at 4.1 μM DnaA. Five additional peaks were eliminated because they were located within rRNA genes, which were subject to artifacts in read mapping, presumably because they are present in ten nearly identical copies in the *B. subtilis* chromosome.

The regions bound by 4.1 μM ATP-DnaA-his were also identified using cisGenome. The resulting 685 binding regions were further analyzed using PeakSplitter [47] to resolve closely spaced regions, yielding a total of 720 binding regions.

DnaA box PSSM

The DnaA box PSSM was developed using DnaA boxes that were observed to bind DnaA in our IDAP experiments. All possible DnaA boxes containing two or fewer mismatches from the TTATNCACA consensus were displayed in a genome browser [48], together with DnaA binding data for 1.4 and 4.1 μM ATP-DnaA-his. DnaA boxes were manually selected if they clearly made a significant contribution to binding (see Fig 3). A 450 kb portion (from 1 to 1.45 mb) of the genome that did not contain any of the eight strongest binding regions was used. A total of 150 DnaA box sequences were collected (S2 Table). The frequency of each base at each of the nine positions was tabulated (S3 Fig), and reformatted as a PSSM as needed for automated searches.
